# Evaluating and using existing models to map probable suitable habitat for rare plants to inform management of multiple-use public lands in the California desert

**DOI:** 10.1371/journal.pone.0214099

**Published:** 2019-04-19

**Authors:** Gordon C. Reese, Sarah K. Carter, Christina Lund, Steven Walterscheid

**Affiliations:** 1 U.S. Geological Survey, Fort Collins Science Center, Fort Collins, Colorado, United States of America; 2 California State Office, Bureau of Land Management, Sacramento, California, United States of America; Chinese Academy of Forestry, CHINA

## Abstract

Multiple-use public lands require balancing diverse resource uses and values across landscapes. In the California desert, there is strong interest in renewable energy development and important conservation concerns. The Bureau of Land Management recently completed a land-use plan for the area that provides protection for modeled suitable habitat for multiple rare plants. Three sets of habitat models were commissioned for plants of conservation concern as part of the planning effort. The Bureau of Land Management then needed to determine which model or combination of models to use to implement plan requirements. Our goals were to: 1) develop a process for evaluating the existing habitat models and 2) use the evaluation results to map probable and potential suitable habitat. We developed a method for evaluating the construction (input data and methods) and performance of existing models and applied it to 88 habitat models for 43 rare plant species. We also developed a process for mapping probable and potential suitable habitat based on the existing models; potential habitat maps are intended only to guide future field surveys. We were able to map probable suitable habitat for 26 of the 43 species and potential suitable habitat for 41 species. Forty percent of the project area contains probable suitable habitat for at least one species (43,338 km^2^), with much of that habitat (43%) occurring on lands managed by the Bureau of Land Management. Lands prioritized for renewable energy development contain 3% of the habitat modeled as suitable for at least one species. Our products can be used by agencies to review proposed projects and plan future plant surveys and by developers to target sites likely to minimize conflicts with rare plant conservation goals. Our methods can be broadly applied to understand and quantify the defensibility of models used in conservation and regulatory contexts.

## Introduction

Management of multiple-use lands, which are intended to meet the needs of current and future generations, requires balancing numerous resource uses and values within and across landscapes. Many public lands in the United States accommodate multiple uses, with some of the most prominent being those managed by the Bureau of Land Management (BLM, Federal Land Policy and Management Act of 1976 [43 USC §1701]) and U.S. Forest Service (Multiple-Use Sustained-Yield Act of 1960 [16 USC §528]). The BLM manages the largest area of public lands in the United States (1,004,358 km^2^, [1]) for diverse resource uses and values including energy production, mineral extraction, recreation, and wildlife conservation, while also protecting land health (Fundamentals of Rangeland Health [43 CFR §4180.1]) and the quality of scientific, scenic, historical, ecological, environmental, air and atmospheric, water resource, and archeological values of the lands (Federal Land Policy and Management Act of 1976 [43 USC §1701]).

In the California desert, there are important and diverse conservation concerns involving many taxa (e.g., desert tortoise (*Gopherus agassizii*), Mohave ground squirrel (*Spermophilus mohavensis*), flat tailed-horned lizard (*Phrynosoma mcallii*), rare plants [2–4]) as well as strong interests in further developing renewable energy (solar, wind, geothermal) and recreational opportunities. The BLM manages many public lands in the California desert, and they recently completed a multiyear, multimillion dollar, land-use planning effort to identify how best to accommodate these uses and values while not unduly degrading public lands. The resulting Desert Renewable Energy Conservation Plan (DRECP) was completed in 2016 [2].

Conservation and management actions in the DRECP require numerous protections for rare plants within the project area. For example, the plan requires rare plant surveys, avoidance setbacks of 0.25 miles from species occurrences, a disturbance cap of 1% of suitable rare plant habitat across the entire plan area, and disturbance caps of 0–20% of suitable habitat within specific areas prioritized for renewable energy development [2]. Protecting habitat that models indicate is suitable, regardless of current occupation status, has rarely been implemented for plants and represents a proactive approach to conservation in areas where species are rare and many areas of the landscape have not yet been surveyed.

The surface disturbance caps in the plan were based on an initial set of habitat suitability models (we use habitat suitability models and habitat models as identical umbrella terms that include all methods for modeling habitat suitability and species distributions) commissioned in 2012 by the California Energy Commission. The original set of habitat suitability models raised numerous concerns, including the selection and accuracy of input data, scale and resolution, thresholding methods, and inclusion of heavily disturbed and developed areas [5,6]. Major tenets of the DRECP are adaptive management and the use of the best available science [2]. To this end, two additional organizations were commissioned to model the habitat suitability of rare plants. The three modeling efforts differed in input data, modeling algorithms, and decision criteria. These habitat models are hypotheses that propose the location of suitable habitat based on occurrence data and environmental variables, and they thereby require testing and validation [7].

When models and the resulting maps are used for regulatory actions, there is a clear need for approaches that are science-based, transparent, and defensible [8,9]. The BLM has committed to using the best available science to inform its management decisions [10]. Sharing data publicly promotes transparency and accountability and facilitates a shared understanding of resources and habitats between managers and stakeholders, all of which can increase stakeholder involvement in management and lead to better long-term management outcomes [11]. Transparency, defensibility, and data accessibility are particularly important in high-profile decisions that affect large land areas and many stakeholders, which is often the case with public land management decisions made by the BLM [2,12,13].

Given the availability of multiple models, the BLM needed to determine which model or combination of models should be used to implement the requirements in the land-use plan. A straightforward approach would be to use the model producing the best map, e.g., the map with modeled suitable habitat overlapping the largest number of occurrences. An evaluation of results is the most common way that models are evaluated and is ideally done with occurrences independent of those used to build the model. In addition to evaluating model results, there is also interest and value in evaluating the modeling procedure. Evaluation of results alone does not prevent the incorporation of a poorly constructed and less defensible model benefitting from unusual predictive success. Such a model may reflect known occurrence data, but the lack of a strong foundation undermines its defensibility, perhaps nowhere more than where no occurrence data exist and where distributional information is therefore greatly needed.

Numerous modeling components have been shown to affect model performance including the quantity and quality of both occurrences and environmental covariates, modeling extent and grain size (resolution), modeling algorithm, and methods and metrics used to assess performance. For example, a model might be based on species occurrence locations that inadequately cover the environmental niche or on a general suite of environmental covariates that are not all relevant to the distribution of the species; the specific covariates used to model suitable habitat are crucial to model performance [14]. Implementing techniques for selecting the best combination of covariates can minimize overfitting and improve performance [15,16]. Many studies have found that these and other steps are important to the performance of habitat suitability models, but we found few that included instructive guidelines for evaluating and comparing existing models (but see [17]). Due to data limitations, model evaluation is often based on a subset of the occurrence data used to build the model which may, largely because of autocorrelation, exaggerate model performance [18]. Independent data should therefore be used for model evaluation when available [19]. Also important, when applicable, is the threshold used to convert continuous habitat suitability results to categorical (usually binary) results [20].

Our goals were to: 1) develop a process for evaluating existing habitat suitability models including both model construction (input data and methods) and the accuracy of the results and 2) use the evaluation results to map probable suitable habitat for use in land-use planning and implementation decisions. We applied our process to a case study focused on 88 models of 43 rare plant species in the California desert for which land-use planning decisions and actions are needed to inform ongoing renewable energy development. In doing so, we developed an approach for evaluating existing habitat suitability models which is broadly applicable and can be used to understand and quantify the defensibility of other models that may be used in conservation and regulatory contexts. We hope this work can contribute to a strong foundation for future efforts focused on accommodating development actions that benefit society while also protecting habitat for rare and declining species.

## Methods

### Study area and species in the California desert

The project area, bounded by 32° 37.109’ and 37° 39.972’ north and 114° 7.848’ and 118° 41.485’ west, encompasses 108,126 km^2^ in the California desert, 44,280 km^2^ of which are public lands managed by the BLM [21], and 1,734 km^2^ of which have been prioritized for renewable energy development [22]. The area includes portions of the Mojave, Colorado/Sonoran and the Great Basin deserts and is dominated by short, isolated mountain ranges within desert plains. Within this area there is a wide elevational range from 85 m below to 2,650 m above mean sea level and a variety of landforms including mountains, plateaus, alluvial fans, playas, basins, and dunes. Given the varied topography and geology of the area, there are many different biological communities within the project area [23].

The 43 plant species we examined were of primary interest in the DRECP planning effort because they were listed under the Endangered Species Act (Endangered Species Act of 1973 as amended, 16 USC §1531–1544), considered rare or sensitive by the state or the BLM, or likely to be impacted by future development within the planning area (Table 1). We present results for Harwood’s eriastrum (*Eriastrum harwoodii*) here and provide complete results for all species in S1 Supporting Information.

**Table 1:** Species for which habitat suitability models were evaluated, conservation status of each species (federally endangered [FE], federally threatened [FT], state endangered [SE], and species designated as sensitive by the Bureau of Land Management [S]), number of existing habitat models, number of those models that exceeded all exclusion criteria, number of occurrences used to evaluate models and the map of probable suitable habitat, and validation metric (capture rate, i.e., the percentage of evaluation occurrences within the modeled suitable habitat). Instances in which models exceeded the exclusion criteria and evaluation data were available, but no capture rate is provided, indicate that the suitability thresholds could not be altered to capture the desired percentage of occurrences. Species for which a disturbance cap has been identified in the Desert Renewable Energy Conservation Plan are indicated by an asterisk (*).

Scientific name	Common name	Cons- erva-tion status	Number of existing habitat models	Number of models exceeding all three exclusion criteria for occurrence data inputs	Number of occurrences (1981–2012) used to evaluate models and map probable suitable habitat	Number of recent (>2012) occurrences available for independent evaluation of probable suitable habitat map	Capture rate (1981–2012 occurrences) of final map of probable suitable habitat
*Abronia villosa var aurita*	Chaparral sand-verbena	S	3	3	14	0	0.93
*Acanthoscyphus parishii* var. *goodmaniana*	Cushenberry oxytheca	FE	2	2	3	0	1.00
*Allium shevockii*	Spanish needle onion	S	1	0	3	0	
*Astragalus bernardinus*	San Bernardino milk-vetch	S	2	2	13	2	0.92
*Astragalus douglasii* var. *perstrictus*	Jacumba milk-vetch	S	2	2	0	0	
*Astragalus lentiginosus* var. *coachellae*	Coachella Valley milk-vetch	FE	2	2	65	32	
*Astragalus nyensis*	Nye milk-vetch	S	1	1	42	0	1.00
Astragalus tricarinatus*	Triple-ribbed milkvetch	FE	3	3	39	9	0.95
*Atriplex argentea* var. *longitrichoma*	Pahrump orache	S	1	0	8	0	
*Calochortus palmeri* var. *palmeri*	Palmer’s mariposa lily	S	2	2	1	0	1.00
Calochortus striatus*	Alkali mariposa-lily	S	4	3	88	76	0.92
*Chamaesyce platysperma*	Flat-seeded spurge	S	1	0	0	0	
*Cylindropuntia munzii*	Munz cholla	S	1	0	0	0	
Cymopterus deserticola*	Desert cymopterus	S	4	3	91	6	0.93
Deinandra mohavensis*	Mojave tarplant	SE	3	2	5	1	1.00
*Echinocereus engelmannii* var. *howei*	Howe’s hedgehog cactus	S	1	0	1	0	
*Eriastrum harwoodii*	Harwood’s eriastrum	S	3	3	67	61	0.96
Erigeron parishii*	Parish’s daisy	FT	3	3	29	17	0.93
*Eriogonum bifurcatum*	Forked buckwheat	S	2	2	67	2	0.93
*Eriogonum ovalifolium* var. *vineum*	Cushenberry buckwheat	FE	2	2	12	3	0.92
Eriophyllum mohavense*	Barstow woolly sunflower	S	5	3	75	27	0.89
*Erythranthe shevockii (*now *Mimulus shevockii)*	Kelso Creek monkey flower	S	1	0	0	0	
*Eschscholzia minutiflora* ssp. *twisselmannii*	Red Rock poppy	S	3	3	29	7	0.93
*Grindelia fraxinipratensis*	Ash Meadows gum-plant	FT	1	0	4	0	
*Heuchera brevistaminea*	Laguna Mountains alumroot	S	1	0	0	0	
*Layia heterotricha*	Pale-yellow layia		1	1	3	5	1.00
Linanthus maculatus*	Little San Bernardino Mountains linanthus	S	3	3	37	27	0.97
*Lupinus excubitus* var. *medius*	Mountain springs bush lupine	S	2	2	8	2	
*Menodora spinescens* var. *mohavensis*	Mojave menodora	S	1	1	7	0	
*Mentzelia tridentata*	Creamy blazing star	S	1	1	19	2	0.89
Mimulus mohavensis*	Mojave monkeyflower	S	5	3	57	5	0.91
*Monardella linoides* ssp. *oblonga*	Tehachapi monardella	S	2	2	29	4	0.97
*Nitrophila mohavensis*	Amargosa niterwort	FE, SE	1	0	2	0	
*Pediomelum castoreum*	Beaver Dam breadroot	S	1	1	2	4	1.00
*Penstemon albomarginatus*	White-margined beardtongue	S	4	3	21	6	1.00
*Penstemon bicolor* ssp. *roseus*	Rosy two-toned beardtongue	S	1	0	7	0	
*Perityle inyoensis*	Inyo rock daisy	S	1	0	5	0	
*Phacelia nashiana*	Charlotte’s phacelia	S	3	3	60	9	0.95
*Phacelia parishii*	Parish’s phacelia	S	1	0	6	6	
*Saltugilia latimeri*	Latimer’s woodland-gilia	S	1	1	10	17	
Sidalcea covillei*	Owens Valley checkerbloom	SE	1	1	17	2	0.94
*Sphaeralcea rusbyi* var. *eremicola*	Rusby’s desert-mallow	S	3	3	48	36	0.92
*Xylorhiza orcuttii*	Orcutt’s woody aster	S	2	2	42	0	0.93
Totals:			*88*	*68*	*1036*	*368*	*26*

### Existing habitat suitability models for rare plants in the California desert

Leaders of the DRECP planning effort originally commissioned development of habitat suitability models for a suite of rare and sensitive plants to inform plan development. To address concerns with the original set of models [5,6] and broaden and update the information base used to implement plan requirements, the BLM commissioned development of additional habitat suitability models by two other contractors for rare plants occurring within the DRECP boundary.

The three sets of models included models for different species and used different inputs, algorithms, extents, resolutions, and modeling decisions [24–28]. We were working with incomplete information for each contractor; in no case did we have the complete suite of information needed to reproduce the models including a comprehensive report, electronic data (occurrences and covariates), model parameters, and full model output. One contractor (working with two subcontractors) produced as many as three models for some species; when multiple models were available, we only used the model recommended for use in the DRECP planning effort. We purposefully do not refer to the individual organizations that developed specific models, as this information is not needed to understand the methods or interpret the results of our study.

### Method for evaluating existing models and mapping suitable habitat

**Fig 1:**
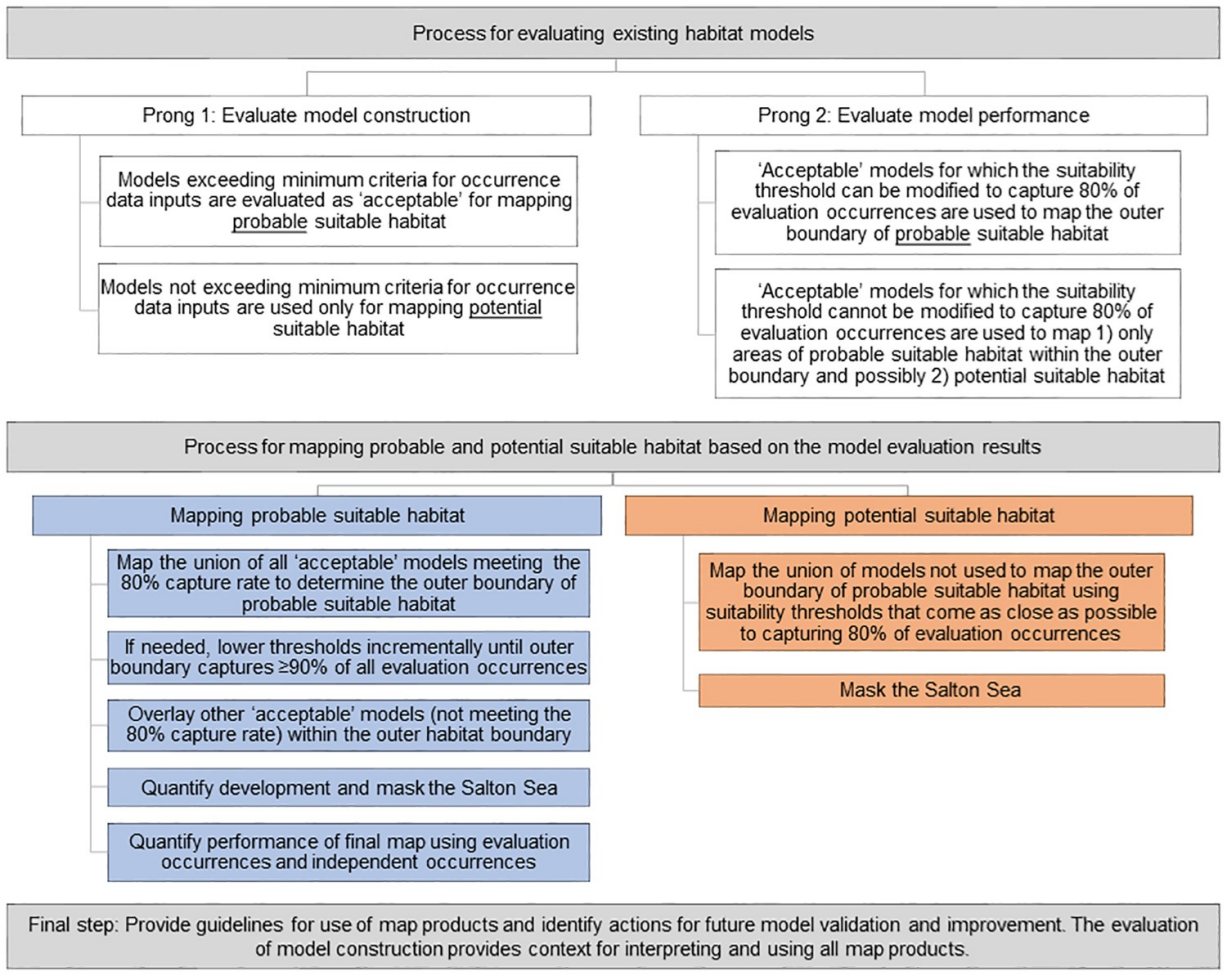
Process for evaluating existing habitat models and mapping probable and potential suitable habitat for rare plants in the California desert.

We developed a two-pronged approach for evaluating the existing habitat models that consisted of: 1) an evaluation of model construction (input data and methods), and 2) a post-hoc quantitative evaluation of model performance (Fig 1). In each prong, we established specific requirements for determining if and how model results would be used to map suitable habitat. We evaluated as acceptable those models meeting minimum criteria for occurrence input data (number, age, and spatial accuracy) and used them to map probable suitable habitat (for implementing land-use plan actions). To map potential suitable habitat (for guiding future plant surveys only), we used models not used to map the outer boundary of probable suitable habitat, as described below (Fig 1).

#### Evaluating model construction (input data and methods)

We developed a list of categories and topics for evaluating model construction (input data and methods) for existing models (Table 2) by expanding and refining the work of Sofaer et al. [17] for application to our case study. We also developed specific criteria, based on the qualitative work of Sofaer et al. [17], to assign ranks of ideal, acceptable, or interpret with caution to each topic (Table 2). Importantly, our criteria had to address the specific context and purpose of this study, be practical to apply, and generate clear evaluation results based on the available information (primarily the reports and data submitted by each contractor).

**Table 2:** Categories, topics, and criteria for evaluating the model inputs and modeling methods used in existing habitat models for rare plants in the California desert. Models were assigned qualitative rankings of interpret with caution (red), acceptable (yellow), or ideal (green) based on the criteria described below. The first three occurrence data topics (number, age, and spatial accuracy of occurrences used to develop the model) also include exclusion criteria (in bold); models exceeding all three exclusion criteria were evaluated as acceptable for use in mapping probable suitable habitat. Models failing any of these exclusion criteria were considered for use in mapping potential suitable habitat for use only in targeting future field surveys (see Methods).

*Category*	*Topic*	*Interpret with caution*	*Acceptable*	*Ideal*	*References*
*Occur- rence data used to develop model*	Number of occur- rences	<25 occurrences, unless the species has a very small range for which ecological characteristics are well known. **Exclusion criteria: <10 occurrences**	25–50 occurrences	>50 occurrences	[29–31]
	Age of occur- rences	Substantial portion (e.g., >20%) of records are from 1980 or earlier, and thus may be less reliable and represent landcover and climate conditions that are no longer accurate. **Exclusion criteria: majority ≤1950**	Most (e.g., ≥80%) records are from 1981 to present	All records are from 2000 to present	[32], K. Lazar, *pers*. *comm*. May 2018
	Spatial accuracy of occur- rences	Substantial portion of records (e.g., >20%) have imprecise locational information (i.e., California Natural Diversity Database [CNDDB] accuracy classes 6–10 [33]). **Exclusion criteria: majority have CNDDB accuracy classes of 6–10.**	Most (e.g., ≥80%) records have precise locational information (e.g., mapped to within 150 m, CNDDB accuracy classes 1, 2, 4 [33])	All records have precise locational information (e.g., mapped to within 150 m, CNDDB accuracy classes 1, 2, 4 [33])	[34], K. Lazar, *pers*. *comm*. May 2018
	Status of occur- rences	Substantial portion of records (e.g., >20%) have an occurrence rank of Fair, Poor, or X ([possibly] extirpated, [33])	Small proportion (e.g., ≤20%) of occurrences have an occurrence rank of Fair, Poor, or X ([possibly] extirpated, [33]) or notes that indicate plants were unhealthy or non-reproductive	Majority of occurrences have an occurrence rank of Excellent or Good [33]	[18], K. Lazar, *pers*. *comm*. May 2018
	Species identifi- cation of occur- rences	Substantial portion of records (e.g., >20%) are from a source for which the reliability of species identification is unknown, or are of a nature that suggests concern with species identification (e.g., historic records with no notes)	Small proportion of records (e.g., 20%) are from a source for which the reliability of species identification is unclear or unknown, or are of a nature that suggests concern (e.g., historic records with no notes)	All records are from a source where data are reviewed, vetted, and quality- checked for accuracy (e.g., CNDDB)	[7,35]
	Spatial bias of occur- rences	Spatial bias is apparent in records (e.g., clumped in space) and records were not thinned to a minimum separation distance	Records are likely to have been collected opportunistically and/or are clumped, but exhibit (or have been thinned to) an acceptable separation distance	Records are well-spaced out or a majority appear to have been collected through systematic surveys	[36]
	Spatial distri- bution of occur- rences	Records are available from a limited portion of the known species’ range	Records are available across a substantial portion of the known species’ range	Records are from systematic surveys conducted across most of the species’ known range	[18]
	Absence data	No absence data are available, and the modeling method does not account for this	No absence data are available, but the modeling method either doesn’t require absence data or accounts for the lack of absence data using appropriate selection of background points	Both presence and absence records are available from systematic surveys	[37]
*Environ-mental covariates*	Ecolog- ical rele- vance	Covariates not selected or justified with reference to the species’ biology	Selection of covariates justified based on general biological and ecological characteristics of this or similar species	Covariate selection justified based on specific biological and ecological characteristics of the species	[14]
	Compre-hensive	Covariates reflect few of the known aspects of the biology of this or similar species	Covariates reflect some major known aspects of the biology of this or similar species	Covariates encompass all major known aspects of the biology of the species	[7]
	Resolu- tion and scale	Spatial, temporal, and thematic resolution and analysis scale of the covariate data not considered or justified in relation to the species’ biology, and/or appear to be a poor match	Spatial, temporal, and thematic resolution and analysis scale of the covariate data considered/ justified in relation to the species’ biology, with the majority appearing to be a reasonable match	Spatial, temporal, and thematic resolution and analysis scale of the covariate data considered/ justified in relation to the species’ biology, and all appear to be a good match	[14]
	Accur- acy	There is no evidence that the accuracy of the covariate data was considered	There is evidence that the accuracy of the covariate data was considered and generally deemed acceptable by the modelers for modeling rare plant habitat in the region	The accuracy of each covariate was considered by the modelers and found to be acceptable for this species	[38]
	Number of covar- iates	The number of covariates meets or exceeds the number of occurrence locations used to develop the model	The number of covariates is reasonable considering the number of occurrence locations used to develop the model	The number of covariates is small (e.g., 1 covariate per 10 occurrence locations) compared to the number of occurrence locations used to develop the model	[39–40]
	Current covariate data	Majority of covariate data reflect a time period from 1980 or earlier	Most (e.g., > 80%) covariate data reflect current conditions (1981-present)	All covariate data reflect current conditions (1981-present)	[41]
	Covar- iate selection	All covariates appear to have been used without regard to their relation to the occurrence locations or ability to explain variation in the data	There is evidence that the relation of covariates to the occurrence locations/ability to explain variation in the data was considered, but it is unclear how covariate selection decisions were made	Covariates were selected using a clear and repeatable process based on their relation to the occurrence locations/ ability to explain variation in the data	[42]
	Correla- tion	All covariates appear to have been retained without consideration of correlations with other covariates	There is evidence that possible correlations between covariates were considered and/or the modeling method accommodates the use of correlated covariates	There is evidence of a priori consideration and analysis of potential correlations between covariates, with testing to assess the sensitivity of model outputs to correlations	[43]
*Model- ing algor- ithm*	Use in the literature	Method is not commonly used in the recent peer-reviewed literature and not enough details are provided to be able to clearly understand or repeat the process	Method is commonly used in the recent peer-reviewed literature, but not enough details are provided to be able to repeat the process	Modeling method is commonly used, well-accepted, repeatable, and clear	[7,8]
	Inter- actions	Modeling method does not consider or accommodate interactions between covariates	Modeling method accommodates pre-defined and/or limited interactions between covariates	Modeling method explicitly allows for undefined interactions between covariates	[44]
	Non- linearity	Modeling method does not consider or accommodate non-linear relationships	Modeling method accommodates pre-defined and/or limited non-linear relationships	Modeling method accommo-dates unspecified non-linear relationships	[36,45]
*Model- ing extent and resolu- tion*	Model extent	Model extent excludes a significant portion of the known species’ range in California	Model extent encompasses most or all of the known species’ range in California	Model extent includes the known species’ range in California and a buffer area outside of it	[46]
	Resolu- tion of model output	Resolution of the model output is substantially finer than that of multiple model covariates and appropriate recommendations for spatial scale of use are not provided, potentially encouraging misuse of the results	Resolution of the model output is finer than that of multiple model covariates, but appropriate recommendations for spatial scale of use are provided	Resolution of the model output is similar to or coarser than model covariates	[39]
*Model selection and thresh- olds*	Model selection	Method of model selection is not commonly used in the peer-reviewed literature, and/or not enough details were provided to be able to clearly understand (or repeat) the process	Method of model selection is commonly used in the peer-reviewed literature or clearly described, but not enough details were provided to be able to repeat the process	The final model was selected using a clear, objective, well- accepted, well- documented, and repeatable process	[47]
	Selection of threshold for mapping suitable habitat	Method of threshold selection is not commonly used in the peer-reviewed literature and/or not enough details were provided to be able to clearly understand (or repeat) the process	Method of threshold selection is commonly used in the peer-reviewed literature or clearly described, but not enough details were provided to be able to repeat the process	The threshold was selected using a clear, objective, well- accepted, and repeatable process	[8,48]

Occurrence data from 1981–2012 in the California Natural Diversity Database (CNDDB) [49] were likely used by all of the contractors, and we therefore used these data to evaluate the occurrence data topics (hereafter referred to as evaluation occurrences). Models failing to exceed exclusion criteria for the number, age, or spatial accuracy of occurrence data (see text in bold in Table 2) were not considered acceptable for mapping probable suitable habitat; they were instead considered for use in mapping potential suitable habitat for the purpose of guiding future plant surveys.

The evaluation results for each model include a brief description of the basis for each topic rank. Topics for which the contractor provided no details were assigned a rank of interpret with caution because of the lack of defensibility of that component of the model.

#### Evaluating the performance of the existing habitat models

The second prong in our evaluation (see Fig 1) utilized the capture rate, i.e., the percentage of occurrences within the modeled suitable habitat, of the evaluation occurrences to evaluate the performance of model results. These evaluation occurrences were likely used by all of the contractors according to both the reports from each organization and to the lead CNDDB botanist (K. Lazar, *pers*. *comm*. May 2018). We used only those occurrence polygons with high spatial accuracy (i.e., accuracy classes 1, 2, 3 [limited to those polygons smaller than or equal in size to a circle with a radius of 150 m], and 4) [33]. We represented the location of each polygon by its internal centroid and randomly sampled the largest possible number of occurrence centroids that were separated by a minimum of 430 m from any other occurrence centroid. This last step ensured that no two evaluation points could have fallen within the same pixel during model construction for nearly all of the models (two of the 88 existing habitat models had pixel resolutions of approximately 355 m).

### Mapping probable suitable habitat based on the existing models

We used only those models that met two conditions to delineate the outer boundary of probable suitable habitat. First, the model had to exceed the three exclusion criteria that we identified for the number, age, and spatial accuracy of occurrences used to develop the model (Table 2). We refer to these models as ‘acceptable’ models. Second, the model results had to capture, or be able to be modified to capture, a specified percentage of the evaluation occurrences. We reviewed the literature, explored multiple possible occurrence capture rates (70%, 80%, 90%, 100%), and worked with botany, wildlife, geographic information system (GIS), policy, and planning staff at the BLM California State Office to select 80% as the required minimum capture rate for mapping the outer boundary of probable suitable habitat for this project. Thus, models evaluated as acceptable for which the suitability threshold selected by the contractor met this capture rate, or models for which the threshold could be altered to meet this capture rate, were used to map the outer boundary of probable suitable habitat (see next section). In some cases, model thresholds were increased to reduce the capture rate to approximately 80%. One organization (Contractor B) used modeling methods that often made it difficult to closely match specific capture rates, and we therefore reduced the acceptable capture rate requirement to 78% for their models.

We used the union of models meeting both criteria, i.e., those exceeding all three exclusion criteria and capturing 80% of the evaluation occurrences, to delineate the outer boundary of probable suitable habitat for a species. If this boundary did not capture at least 90% of the evaluation occurrences, we decreased suitability thresholds of the base models until the final (union) map met the 90% capture rate requirement. The methodology of one organization (Contractor B) made it difficult to closely match the 90% capture rate and we therefore reduced the acceptable capture rate requirement to 89% when their model was included. When only one model was deemed acceptable for mapping probable suitable habitat, the threshold of that model was adjusted to capture as close to 90% of the evaluation occurrences as possible.

Within the outer boundary of probable suitable habitat, we mapped all models evaluated as acceptable, i.e., those models that exceeded the exclusion criteria regardless of whether or not they met the 80% minimum capture rate. For each pixel of probable suitable habitat, we quantified both the number of models that predicted suitable habitat and the average habitat suitability score [50].

We finalized each map by masking the Salton Sea [51]. Additionally, we calculated the area of probable suitable habitat classified as developed (i.e., ‘LF20: Developed’) by the Landscape Fire and Resource Management Planning Tools Project (LANDFIRE) existing vegetation type (EVT) dataset [52]. We considered masking developed areas in both the maps and electronic datasets; however, in a visual examination of National Agriculture Imagery Program (NAIP) imagery ([53], 60 cm resolution), we found multiple occurrence locations for multiple species that were classified as developed by LANDFIRE EVT. Thus, we chose not to mask developed areas and instead present the full model results.

In addition to the evaluation occurrences (1981–2012 CNDDB), we also computed capture rates of more recent CNDDB occurrences (2013–2018), representing an independent evaluation. We processed these recent occurrences in the same way as the 1981–2012 occurrences. While there were too few of these more recent occurrences for use in the baseline assessment for all species (see Table 1), the capture rates of these independent occurrences, when available, provide valuable additional information to map users.

### Mapping potential suitable habitat for use in guiding future plant surveys

We mapped potential suitable habitat (for use in targeting future plant surveys) with models that were not used to map the outer boundary of probable suitable habitat. In general, we mapped potential suitable habitat using: 1) models that failed one or more exclusion criteria for occurrence data, and 2) models that could not be modified to meet our 80% minimum occurrence capture rate criteria. One contractor masked their model results such that their recommended map included only those watersheds with one or more known occurrences. We considered the masked results for mapping probable suitable habitat; we used the unmasked results for mapping potential suitable habitat regardless of whether the masked version was used in our map of probable suitable habitat. Thresholds for the individual models used to map potential suitable habitat were altered, when possible, to capture as close to 80% of the evaluation occurrences as possible.

### Individual and multispecies products

Results were packaged in multiple ways to facilitate use by the BLM and others with an interest in resource management in the California desert. For each species, we provided: 1) an evaluation of the model construction (input data and methods), 2) a map of the existing habitat suitability models, 3) a map and performance evaluation of probable suitable habitat, and 4) a map of potential suitable habitat for guiding future plant surveys. We also developed a multi-species product representing the number of species for which each pixel was modeled as probable suitable habitat, to assist in initial screening of proposed development projects. Probable and potential suitable habitat maps are provided as: 1) hard copy maps with relevant boundaries and land-use designations (S1 Supporting Information), and 2) electronic, open-access raster datasets [50].

## Results

### Evaluating model construction (input data and methods) of the existing models

The three existing habitat models for Harwood’s eriastrum all exceeded the exclusion criteria for number, age, and spatial accuracy of occurrence data. Thus, we considered all for mapping probable suitable habitat (Table 3). There were topics evaluated as ‘interpret with caution’ for each model, particularly models from contractors B and C in the categories of occurrence data (contractor C), environmental covariates (both), and modeling algorithm (both). This same general pattern held true for the remaining 42 species (see S1 Supporting Information Tables B1-B43), as many of the interpret with caution rankings were related to unclear or unspecified methods that applied to every species modeled by that organization.

**Table 3:** Evaluation of model construction (input data and methods) for the three existing habitat models for Harwood’s eriastrum (*Eriastrum harwoodii*). Please see Table 2 for a description of the criteria used to rate each topic as interpret with caution (red), acceptable (yellow), or ideal (green). Evidence in the organization’s submitted report, data, and metadata was used as the basis for determining topic ratings. For most occurrence data topics (indicated by an asterisk [*]), minimum ratings were based on occurrence data through 2012 currently available in the California Natural Diversity Database [49], as all contractors used CNDDB data in model development (see Methods).

Category	Topic	Contractor A	Contractor B	Contractor C
Occurrence data used to develop the model	Number of occurrences*	Report/data indicate that model was built from 255 occurrences. Currently available CNDDB data indicate 55 occurrences were likely used by this contractor for model development.	Report/data indicate that model was built from 49 occurrences. Currently available CNDDB data indicate 56 occurrences were available for use by this contractor for model development.	Report/data indicate that model was built from 55 occurrences. Currently available CNDDB data indicate 56 occurrences were available for use by this contractor for model development.
	Age of occurrences*	Report indicates use of occurrence data from 1981–2012.	1 of 56 currently available CNDDB occurrences is from before 1981.	1 of 56 currently available CNDDB occurrences is from before 1981.
	Spatial accuracy of occurrences*	Report/data indicate occurrences with uncertainty >250–500 m were excluded.	10 of 56 (18%) currently available CNDDB occurrences have imprecise spatial accuracy.	10 of 56 (18%) currently available CNDDB occurrences have imprecise spatial accuracy.
	Status of occurrences*	11 of 56 (20%) currently available CNDDB occurrences have Fair or Poor occurrence ranks.	11 of 56 (20%) currently available CNDDB occurrences have Fair or Poor occurrence ranks.	11 of 56 (20%) currently available CNDDB occurrences have Fair or Poor occurrence ranks.
	Species identification of occurrences*	All records are from CNDDB, for which species identification is reliable.	All records are from CNDDB, for which species identification is reliable.	All records appear to be from CNDDB, for which species identification is reliable.
	Spatial bias of occurrences*	Report indicates that records were thinned to 1 presence in each 270 m cell.	CNDDB records are separated by 250 m or more [33]. Report does not indicate any post processing of occurrence data.	CNDDB records are separated by 250 m or more [33]. Report indicates that habitat was not modeled for species with occurrence records limited to “a very small area under highly constrained habitat conditions”.
	Spatial distribution of occurrences*	Currently available CNDDB records appear to be from a substantial portion of the occupied geographic subdivisions for the species in California [54].	Currently available CNDDB records appear to be from a substantial portion of the occupied geographic subdivisions for the species in California [54].	Currently available CNDDB records appear to be from a substantial portion of the occupied geographic subdivisions for the species in California [54].
	Absence data	MaxEnt randomly samples background locations.	Modeling method does not require absence data.	Modeling method does not require absence data.
Environ- mental covariates	Ecological relevance	The report states that the following criteria were used for selecting covariates: “Significant habitat factor for ≥1 of the species; adequate resolution; available for entire study area; best available (accuracy and currency); based on ecological studies”. *A team of BLM botanists determined that the selected covariate set was relevant to this species and more generally to the suite of rare plants considered in this study*.	The report states that habitat factors used were based on “life histories, specific habitat requirements, and geographic distribution of each species”. *A team of BLM botanists determined that the selected covariate set was relevant to this species and more generally to the suite of rare plants considered in this study*.	The report states that there was collaboration in identifying the covariates with “BLM and team members at UC Riverside Center for Conservation Biology”. *A team of BLM botanists determined that the selected covariate set was relevant to this species and more generally to the suite of rare plants considered in this study*.
	Comprehensive	The report states that covariates are “based on available ecological studies and life history accounts for that species”. *A team of BLM botanists determined that this was a generally comprehensive set of covariates*, *but that surficial geology is important to some species*. *The effect of this omission was unknown but of concern*.	It appears from the report that the same covariates were used for all species: “spatial data for eight habitat variables, including associated vegetation types, soil texture, parent material (rock type), ecological subregion, watershed boundaries, elevation, and slope”. *A team of BLM botanists determined that this was a generally comprehensive set of covariates*, *but that surficial geology and climate variables capturing seasonality could be strong predictors for some species*. *The effects of these omissions were unknown but of concern*.	The report states that there was collaboration in identifying the covariates with “BLM and team members at UC Riverside Center for Conservation Biology”. Because of this collaboration, we assumed that the covariate data used are reasonably comprehensive. *A team of BLM botanists agreed with this conclusion*.
	Resolution and scale	Covariates probably match temporally and thematically, but spatial resolution (270–360 m) was not justified/ explained. *A team of BLM botanists agreed that a stated rationale would have been preferable but deferred to the modeling group*.	Covariates probably match temporally and thematically, but 30 m spatial resolution was not justified/explained. *A team of BLM botanists agreed that a stated rationale would have been preferable but deferred to the modeling group*.	Covariates probably match temporally and thematically, but spatial resolution (180–250 m) was not justified/ explained. *A team of BLM botanists agreed that a stated rationale would have been preferable but deferred to the modeling group*.
	Accuracy	Most covariate datasets are from known and commonly used sources, and report indicates the accuracy of the covariate data was considered.	Although most covariate datasets are from known and commonly used sources, the report did not document any consideration of covariate data accuracy within the project boundary.	Although most covariate datasets are from known and commonly used sources, the report did not document any consideration of covariate data accuracy within the project boundary.
	Number of covariates	Model includes 12 covariates and 255 occurrences (though from ca. 55 original occurrence locations)	Model includes 19 covariates and 49 occurrences	Model includes 5 covariates and 55 occurrences; Report stated that no more than one variable per 10 occurrences was allowed.
	Current covariate data	Covariates used represent contemporary conditions (or resources such as soils and geology that change very little over time).	Covariates used represent contemporary conditions (or resources such as soils and geology that change very little over time).	Covariates used represent contemporary conditions (or resources such as soils and geology that change very little over time).
	Covariate selection	MaxEnt weights covariates based on relationships with occurrences.	No covariate selection process–all covariates used for all species.	A covariate selection process is implied in the report, but unclear.
	Correlation	MaxEnt accommodates the use of correlated covariates [55]	Report provides no indication that correlations among covariates were considered.	Report provides no indication that correlations among covariates were considered.
Modeling algorithm	Use in the literature	MaxEnt is commonly used and accepted for species distribution modeling [56,57]	Method of using occurrence locations to inform a GIS-based overlay model is uncommon in the recent peer-reviewed literature, and the report did not provide sufficient detail to fully understand the modeling process.	Modeling method (based on Mahalanobis distance, implemented in the R package aster) is uncommon in the field of species distribution modeling, and the report did not provide sufficient detail to fully understand the modeling process.
	Interactions	MaxEnt accommodates covariate interactions.	The method is non-statistical, and thus ignores covariate interactions.	Not enough information was provided in the report to address this topic, but consideration of covariate interactions is apparently not automatic [58].
	Non-linear	MaxEnt accommodates non-linear relationships between covariates and occurrence data.	Not enough information was provided in the report to address this topic. The modeling method could accommodate non-linear relationships, but it is unclear if it did so.	Not enough information was provided in the report to address this topic, but incorporation of non-linear relationships between covariates and occurrence data is apparently not automatic [58].
Modeling extent and resolution	Model extent	Project area included most of the area of occupied geographic subdivisions for the species in California [54].	Project area included most of the area of occupied geographic subdivisions for the species in California [54].	Project area included most of the area of occupied geographic subdivisions for the species in California [54].
	Resolution of model output	Output resolution (270–360 m) is finer than resolution of multiple covariates, and appropriate recommendations for spatial scale of use are not provided.	Output resolution (30 m) is finer than multiple covariates, and appropriate recommendations for spatial scale of use are not provided.	Output resolution (180–250 m) is finer than resolution of multiple covariates, and appropriate recommendations for spatial scale of use are not provided.
Model selection and thresholds	Model selection	MaxEnt implements a machine-learning algorithm to determine covariate weights, but some details needed to repeat the process were not provided in the report.	A single model was built by weighting covariate values by occurrence locations. Subsequently, watersheds without documented occurrences along with developed areas and permanent water were removed. Report did not provide sufficient detail to be able to repeat the process.	Several combinations of the covariates were compared, but specific details are not provided in the report.
	Selection of threshold for mapping suitable habitat	Threshold selected based on maximum training specificity plus sensitivity [59].	Not enough information was provided in the report to address this topic.	Report indicates that they used the suitability value that “best encompassed the species known location points”. The percentage of occurrences captured varied across species, so the details of this step are unclear.

### Evaluating performance of the existing models

**Fig 2:**
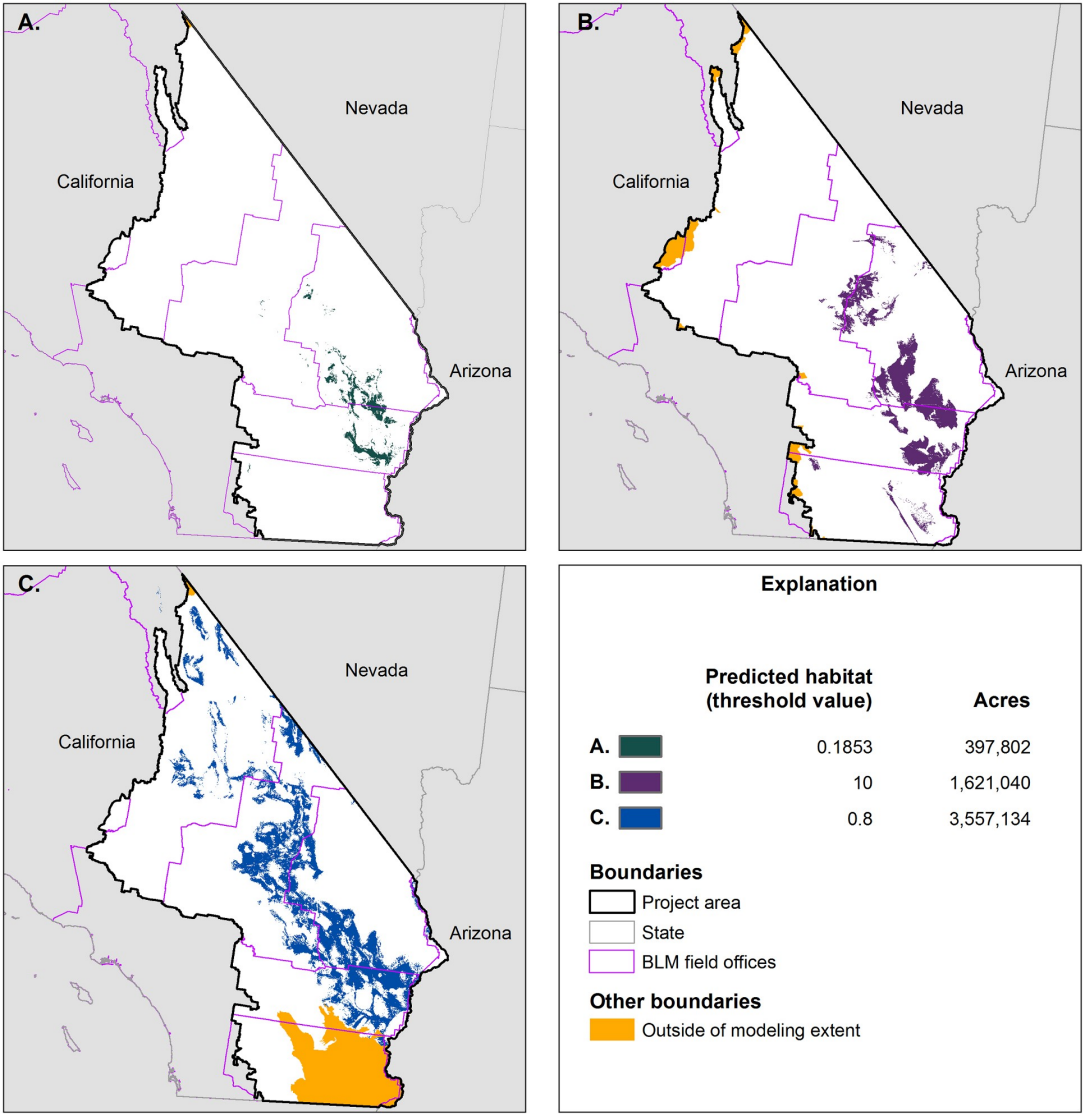
Existing habitat models for Harwood’s eriastrum developed by a) Contractor A, b) Contractor B, and c) Contractor C. The legend includes the threshold value used by each contractor to define suitable habitat as well as the resulting number of acres of suitable habitat within the project area.

The Harwood’s eriastrum models, as delivered by the individual organizations, captured between 66 and 81% of the 67 evaluation occurrences. The area of modeled suitable habitat also varied, from 397,802 to 3,557,133 acres (Fig 2). Two of the three models (contractors A, B) captured, or were modified to capture, 80% of the evaluation occurrences. Contractor C provided only model results above their selected suitability threshold, and thus we could not decrease the threshold to meet the 80% capture rate criteria for Harwood’s eriastrum. We therefore used the results of only two of the three models to map the outer boundary of probable suitable habitat. Two models were similarly used to map the outer boundary of probable suitable habitat for many other species (S1 Supporting Information). The number of acres of modeled suitable habitat also varied widely across contractors for some of the other rare plant species (S1 Supporting Information).

### Mapping probable suitable habitat for Harwood’s eriastrum

**Fig 3:**
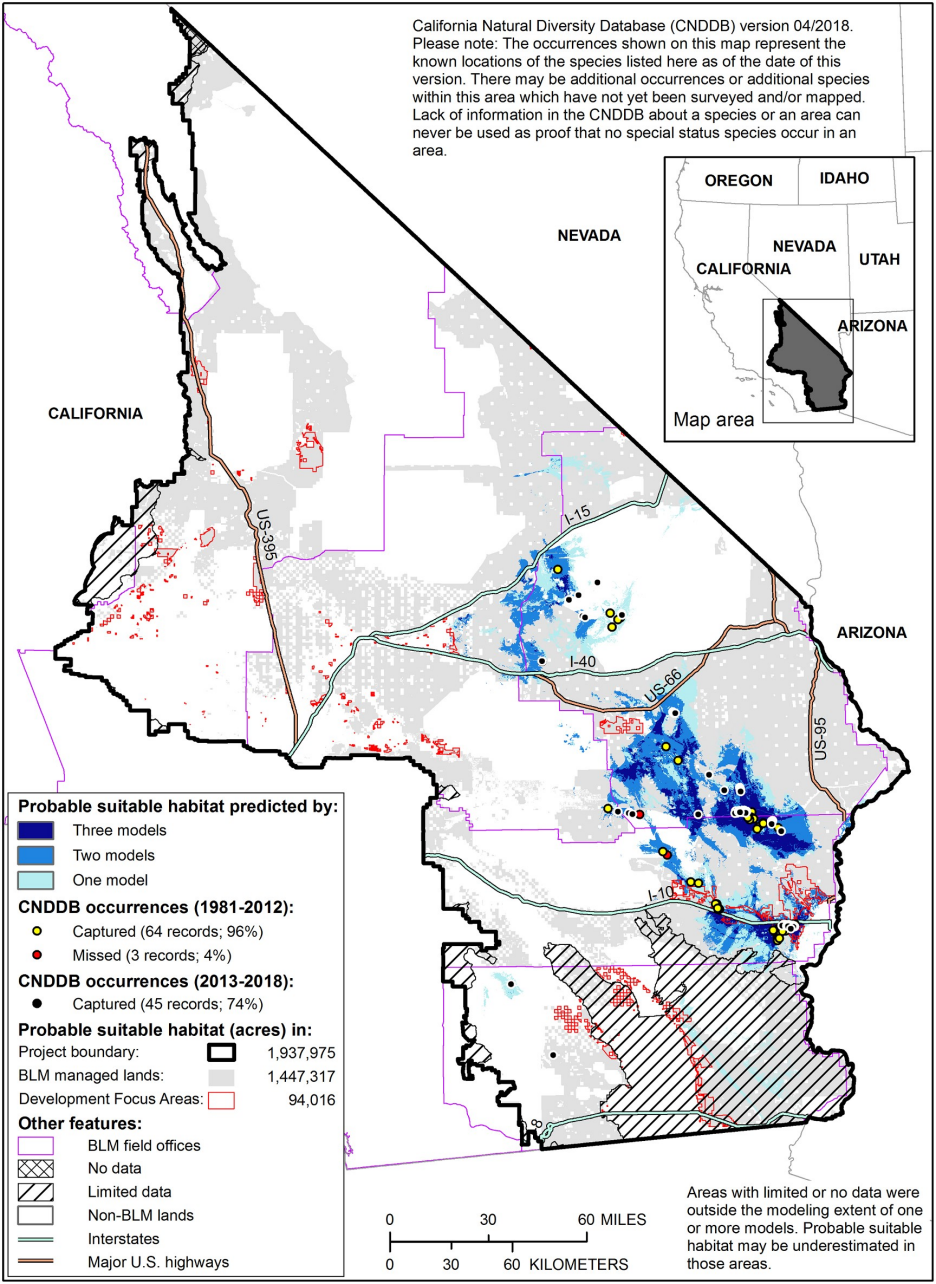
Probable suitable habitat for Harwood’s eriastrum. Shades of blue indicate the number of models predicting suitable habitat for the species. Results of the post-hoc performance evaluation using two sets of occurrences (evaluation occurrences from 1981–2012 and independent occurrences from 2013–2018, both from CNDDB) are shown on the map and in the legend, along with the area of probable suitable habitat within three boundaries: the project area, public lands managed by the Bureau of Land Management (BLM), and areas prioritized for development (Development Focus Areas) as identified in the Desert Renewable Energy Conservation Plan [2]. Note that some areas mapped as probable suitable habitat are currently classified as developed [52].

The (spatial) union of the two Harwood’s eriastrum models meeting the 80% desired capture rate formed the outer boundary of probable suitable habitat, encompassing 1,937,975 acres within the project area (Fig 3). The third model, which exceeded the three exclusion criteria but could not be altered to capture 80% of the evaluation occurrences, was only mapped within the already established outer boundary. As a result, 64% of the probable suitable habitat was mapped by multiple models (i.e., 2 or 3 models), and average habitat suitability scores ranged from 10 to 89 (scores were standardized from 1–100, [50]). Less than 1% (14,836 acres) of the mapped probable suitable habitat is currently classified as developed by LANDFIRE EVT. The final map of probable suitable habitat captured 96% of the evaluation occurrences (*n* = 67) and 100% of the 2013–2018 (i.e., independent) occurrences (*n* = 14).

Overall, we were able to map probable suitable habitat for 26 of the 43 species of interest (Table 1), with areas of probable suitable habitat ranging from 22,453 to 2,632,407 acres (S1 Supporting Information Table A1). On average, 45% of species’ probable suitable habitat occurred on public lands managed by the BLM (range 15–85%) and 2% of that habitat was within areas prioritized for renewable energy development in the DRECP (range 0–6%, S1 Supporting Information Table A1). On average, 2% of probable suitable habitat for each species was mapped by LANDFIRE EVT as developed (range 0–20%, S1 Supporting Information Table A1).

### Mapping potential suitable habitat for use in guiding future plant surveys

**Fig 4:**
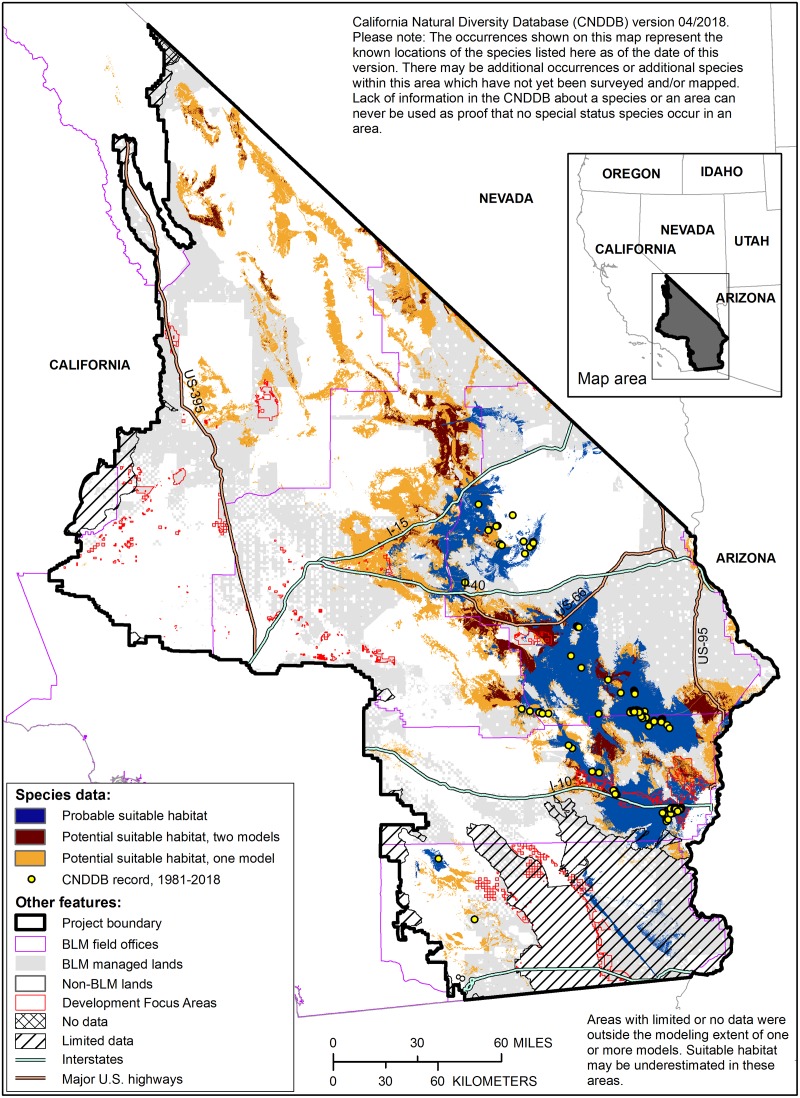
Potential suitable habitat for Harwood’s eriastrum for guiding future plant surveys. Shades of orange indicate the number of overlapping models predicting potential suitable habitat outside of the probable suitable habitat boundary (shown in blue). Note that some areas mapped as potential suitable habitat are currently classified as developed [52].

As all three existing habitat models for Harwood’s eriastrum were used to map probable suitable habitat, the only additional areas mapped as potential suitable habitat (i.e., outside of the probable suitable habitat boundary) were: 1) the additional area mapped as suitable by Contractor C, since the threshold for their model could not be decreased to capture 80% of the evaluation points, and 2) the area mapped as suitable by Contractor B that was outside of watersheds with known occurrence locations, i.e., the unmasked version of their model (Fig 4). Within the area mapped as potential suitable habitat, 32% of the area was mapped by more than one model and the average of the standardized habitat suitability scores ranged from 35 to 89 [50].

### Multispecies product

**Fig 5:**
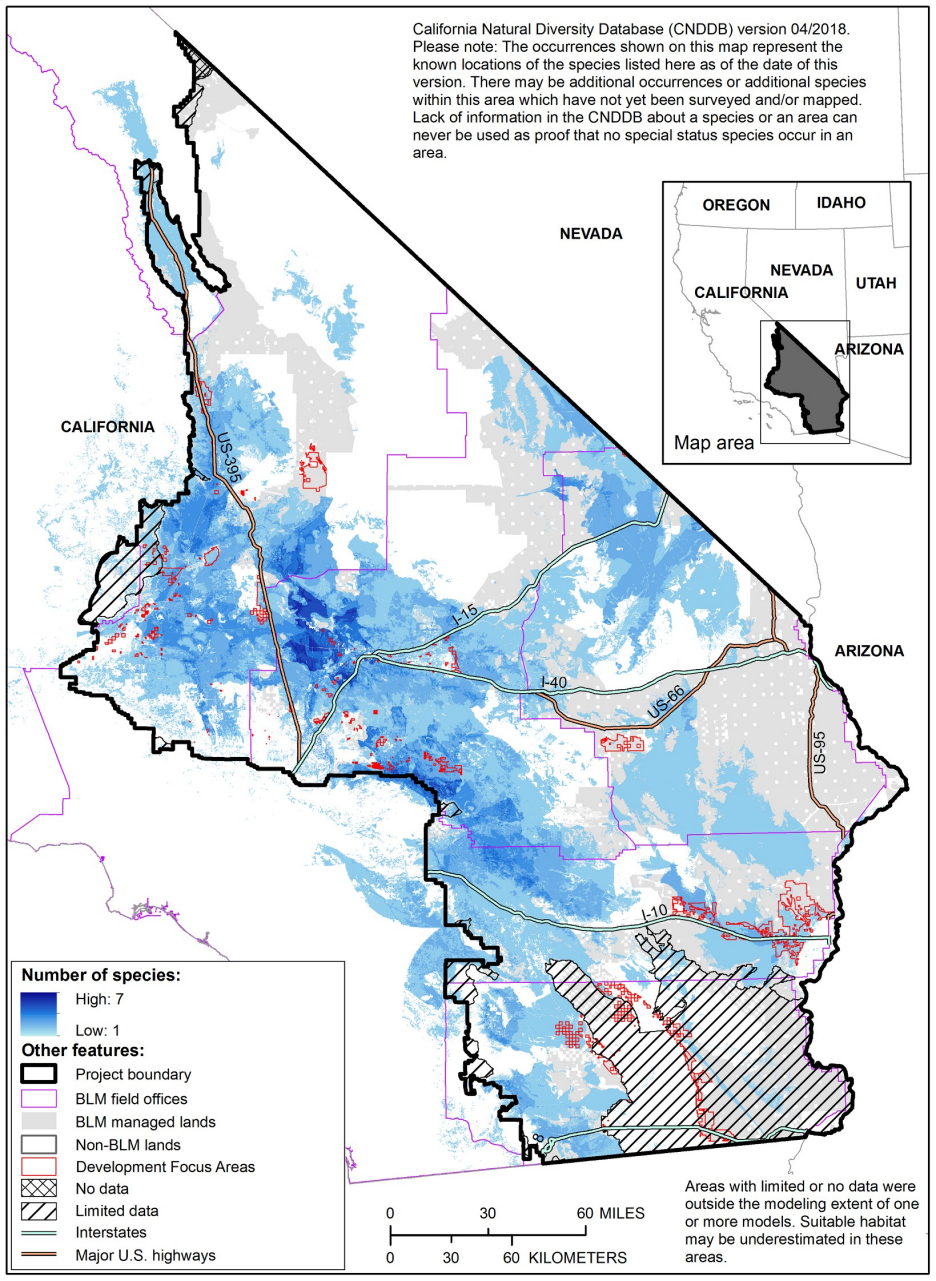
Multispecies map of probable suitable habitat. Shades of blue indicate the number of species for which probable suitable habitat is predicted. Note that some areas mapped as probable suitable habitat are currently classified as developed by LANDFIRE [52].

We identified 10,708,975 acres within the project boundary (40% of the total project area) that are mapped as probable suitable habitat for at least one rare plant species (Fig 5). Nearly 45% (4,554,576 acres) of that habitat occurs on public lands managed by the BLM, including 193,659 acres that occur within areas prioritized for renewable energy development identified in the DRECP [2].

## Discussion

Effective management of multiple-use lands requires openly acknowledging tradeoffs between potentially conflicting resource uses such as development, recreation, and conservation [60]. Pressure is increasing to allow more intensive uses of public lands [61], which also provide critical habitat for sustaining many rare and declining species [62]. Renewable energy development in the California desert can move California closer to its sustainability goals for energy acquisition (California Senate Bill 350: Clean Energy and Pollution Reduction Act [2015]), but can involve tradeoffs when areas prioritized for development coincide with areas supporting rare plants and animals [63,64]. Clear, defensible, science-based information can help managers implement measures in land-use plans that help to balance such tradeoffs.

The areas prioritized for renewable energy development within the DRECP were designed to both maximize renewable energy potential and minimize environmental conflicts [65]. Our results indicate that while the vast majority of rare plant habitat occurs outside of these development focus areas, approximately 3% of the habitat suitable for Harwood’s eriastrum and 2% (range 0–6%) of the habitat suitable for other rare plant species occurs within the development focus areas. Project proponents and resource managers working in the DRECP can use our maps and other spatial data to help guide the selection of project locations to avoid or minimize loss of rare plant habitat in these areas. Project proponents are also required to conduct plant surveys during project evaluation, and results of these surveys can inform both the design of development projects and future habitat modeling and validation efforts.

The method we developed builds on existing science that highlights key topics and concerns in developing species distribution models [7,17,44,55]. We addressed common problems that can occur when contractors deliver to management agencies model outputs without the full suite of associated occurrence and covariate data [7]. A goal of the original modeling efforts was identification of both the location and degree of potential conflict between rare plant habitat and renewable energy development. Our project evaluated the construction and results (performance) of competing habitat models and provided results that can be used by land managers. Our evaluation process, particularly with respect to model construction (input data and methods), highlights numerous modeling steps that still require science-based recommendations and imposes clear criteria for use of information that may be used in a regulatory capacity. Additionally, our method is transferable and incorporates three actions to facilitate the evaluation and use of existing models for conservation and management decision-making: 1) evaluation based both on information provided by the model developer and on high-quality evaluation occurrences from a reliable source (here, one of the more than 80 natural heritage programs that NatureServe oversees), 2) close collaboration with the agency to identify who will use products and for what purposes (here, probable suitable habitat maps for informing land-use decisions and implementation of surface disturbance caps in the DRECP and potential suitable habitat maps for informing future plant surveys), and 3) provision of products in multiple formats through peer-reviewed outlets to increase transparency, defensibility, and data accessibility.

### Using probable suitable habitat maps to inform development and implement the land-use plan

Our study provides clear and relevant products as well as guidelines for their interpretation and use by stakeholders with an interest in public land management in the California desert. The final maps of probable suitable habitat represent our goal of mapping the best available representation of probable suitable habitat based on the existing models and data for the species in the project boundary. Populations of these rare and sensitive plants are likely to have a better chance of persisting into the future if probable suitable habitat is protected from loss and degradation. Areas where multiple models overlap and where average habitat suitability scores are high provide additional indications that mapped areas are suitable for the species. The evaluation of model inputs and methods, which is less common than evaluations of model results [17], provides context for map use—users can have more confidence in maps for which most evaluation topics were rated as ideal or acceptable (Table 3, S1 Supporting Information Tables B1-B43). It is also important to test model results with evaluation data, ideally independent of those used to build the model (Fig 3, S1 Supporting Information); high capture rates provide users with greater confidence in map results, particularly when sample sizes are larger (e.g., ≥10 occurrence locations).

A primary envisioned use for the maps of probable suitable habitat is for agency screening of areas proposed for future renewable energy development. Our multispecies map could be used in the first of four screening steps to identify whether project areas may contain suitable habitat for rare plants. When used together, the multispecies data product and probable suitable habitat data for the individual species provide information about both how many species and which individual species may have suitable habitat at a given location. The presence of probable suitable habitat near a proposed development area is an indication that probable suitable habitat may be present on the site. The electronic data [50] are presented at a spatial resolution of 10 m pixels, which was used so that none of the contractors’ model results would be lost. Using the maps of probable suitable habitat at a resolution no smaller than 360 m (i.e., 36 pixels x 36 pixels) ensures that the maps are not used at a resolution finer than that of the coarsest input model.

A second step in the screening process is examination of the relevant probable suitable habitat species maps and recorded occurrence locations (occurrence data may be requested from CNDDB, https://www.wildlife.ca.gov/Data/CNDDB). Investigating average habitat suitability scores as well as the number of models predicting suitable habitat for a species will provide additional information, with larger scores and more models suggesting greater confidence in the suitability of a site for the species.

A third step in using our products for project screening relates to informing siting decisions (e.g., siting of a project within a Development Focus Area). Consulting information on habitat characteristics of species that may be present together with other relevant spatial data (e.g., the most recently available soils, surficial geology, land cover, and species occurrence data) will provide additional insight into the suitability of the area for the species in question. This step may provide crucial new information for areas that are currently data poor or not yet surveyed.

A final step in using our products to help inform land-use decisions, including design of the surface disturbance footprint of a proposed development, is to conduct on-the-ground plant and habitat surveys whenever our products indicate that probable suitable habitat may be present on or near the proposed disturbance area. Newly collected occurrence data from these surveys can be used to further test existing models.

Some areas of modeled probable suitable habitat might not be occupied at any given time because of fluctuations in population numbers and distribution (e.g., due to unfavorable weather conditions, competition with other native or invasive species, herbivory [66]). The distributions of rare species are particularly difficult to predict [67]; the limited knowledge of and data for most rare desert plants means that some areas predicted to be suitable may not in fact be and vice versa. For example, some locations mapped as suitable habitat may have been recently developed, or there may be small inclusions (too small to be mapped in land-cover products) of a land-cover type that is likely to support the species within larger areas of a different (less suitable) land-cover type. As we gather more plant survey data and more information about environmental factors (e.g., soils, weather and climate, physiological limits) driving the distribution of these species, we can refine habitat suitability models.

### Using the maps of suitable habitat to target future plant surveys

Maps of potential suitable habitat are intended only as a tool for maximizing the benefits of on-the-ground plant surveys for collecting new data that can be used to develop and update habitat suitability models [68]. Prioritizing future survey effort in areas that are mapped as *probable* suitable habitat by multiple models, have relatively high habitat suitability values, and are distant from known occurrence locations may provide the greatest return on investment. Using the maps of *potential* suitable habitat to select future survey locations in a similar manner may further expand our information about and understanding of species’ environmental tolerances.

### Improving models and data for public land management applications

Our case study application of these methods for evaluating existing models and using the results to map probable suitable habitat revealed five common areas in which available information limited our ability to confidently map probable suitable. First, for some of the species examined, existing models were based on a very small number of occurrences (<10 locations, see S1 Supporting Information). In other cases, there were no recent (>1980) occurrences in CNDDB with which to evaluate the performance of the existing model (Table 1). Targeting future survey efforts first toward these two categories of species may improve both circumstances and contribute to defensible and accurate mapping of probable suitable habitat. A second priority for additional surveys is those species for which there are fewer than 50 recent, spatially accurate occurrences, or for which the locations are highly clustered. Studies have indicated that 50 or more occurrences may be needed to stabilize performance with some modeling approaches [30], and clustered occurrence data covering a small proportion of a species’ range are less likely to represent the breadth of environmental conditions that are suitable for the species [18]. Additional considerations in prioritizing future plant surveys include the conservation status of the species at federal and state levels, the overlap of the species’ habitat with locations prioritized for energy or other types of development (e.g., recreation areas), the sensitivity of the species to projected climate change in the region, and the life history characteristics of the species.

Defensibility of model results is greatest when the organizations developing models for informing regulatory activities use methods and algorithms that are tested and well established in the scientific literature. This was not the case for two of the three sets of models examined here. Also, agencies find it difficult to use information that is only available for part of the project area. Expanding current project areas, in consultation with land managers, can accommodate future planning efforts that use slightly altered boundaries, as was the case here (J. Karuzas, *pers*. *comm*.). Delivering complete and continuous model outputs to agencies facilitates their use should different habitat suitability thresholds be relevant in the future. The multiple-use mandate of the BLM and other public land managers means that the tradeoff between using lower thresholds that identify larger areas of habitat, but may leave fewer development opportunities across the landscape, is a key consideration; such decisions may change over time and with different administrations. For example, 24 of the existing models were difficult to use for mapping probable suitable habitat because only those results above the contractor’s selected threshold were delivered to the agency.

Environmental covariates used in the models examined here have been criticized for being a ‘kitchen sink’ approach to modeling habitat without adequate consideration of spatial resolution or accuracy [5]—criticisms that are by no means unique to these models [69]. Soils data for much of the project area are spatially coarse, which is not ideal for modeling rare plant habitat, but new soil mapping efforts are underway (Jim Weigand, *pers*. *comm*.) and may improve future models. New modeling efforts may also benefit from use of the most recently available spatial data on surficial geology and seasonal temperature and precipitation, all of which were only included in some of the models examined here, despite relevancy to the distribution of many desert plants. Agencies may also want to consider using contract language that requires that new modeling efforts publish clear and comprehensive documentation and full results in a reputable, peer-reviewed outlet (e.g., scientific journal, government technical report). Finally, areas that provide suitable habitat today may no longer provide conditions that are optimal for the species in 10, 20, or 50 years. Long term habitat management and conservation efforts on public lands can benefit from considering how and over what time period climate and land-cover conditions are likely to change, revising models as needed based on more recent data, and planning for protection of habitat areas and corridors that will allow species to respond and move, over time, to new locations that may provide suitable habitat in the future.

## Supporting information

S1 Supporting Information Complete materials for all 43 rare plant species evaluated in the study. Materials consist of a summary table, evaluations of the model construction for all species, maps of existing habitat models for all species, maps of probable suitable habitat for 26 species, and maps of potential suitable habitat for targeting future plant surveys for 41 species.
(PDF)
