# Supplementary material for: Evaluating and using existing models to map probable suitable habitat for rare plants to inform management of multiple-use public lands in the California desert
Source: PLoS One. 2019 Apr 19;14(4):e0214099. doi: 10.1371/journal.pone.0214099 (PMC6474587; doi:10.1371/journal.pone.0214099)

S1 Supporting Information

In this supporting information we provide complete materials for all 43 rare plant species evaluated in the study, consisting of a summary table, evaluations of the model construction for all species, maps of existing habitat models for all species, maps of probable suitable habitat for 26 species, and maps of potential suitable habitat for targeting future plant surveys for 41 species.

For simplicity, we include below general captions for all tables and figures for individual species in this supporting information, so that the captions do not have to be repeated many times. Figures and tables for Harwood's eriastrum (*Eriastrum harwoodii*) are presented first, as they comprise the case study in the article and contain full details for all cells in the evaluation of the model construction (Table B1_*Eriastrum harwoodii*).

Reference not contained in main text:

70. US Department of Agriculture Natural Resources Conservation Service. The PLANTS Database. 2018 [cited 2018 Jul 11]. Available from http://plants.usda.gov.

Table A1. Rare and sensitive plant species of primary interest in the Desert Renewable Energy Conservation Plan (DRECP, [2]) planning and implementation effort and evaluated for this project (n=43). For those 26 species for which we were able to map probable suitable habitat, we provide the acres of probable suitable habitat 1) in the project boundary, 2) on public lands managed by the Bureau of Land Management, 3) within areas prioritized for energy development in the DRECP, and 4) currently classified as developed by the Landscape Fire and Resource Management Planning Tools Program [52]. We also provide the page number that information on this species begins in the supplementary material.

Table and figure num-ber	Scientific name	Common name	Acres of probable suitable habitat in project boundary	Acres of probable suitable habitat on BLM-managed lands	Acres of probable suitable habitat in areas prior-itized for develop-ment	Acres of proba-ble suitable habitat classi-fied as devel-oped	Page num-ber
2	*Abronia villosa var aurita*	Chaparral sand-verbena	878,917	257,919	27,463	125,547	17
3	*Acanthoscyphus parishii* var. *goodmaniana*	Cushenberry oxytheca	47,863	40,759	222	68	24
4	*Allium shevockii*	Spanish needle onion					30

Table and figure num-ber	Scientific name	Common name	Acres of probable suitable habitat in project boundary	Acres of probable suitable habitat on BLM-managed lands	Acres of probable suitable habitat in areas prior-itized for develop-ment	Acres of proba-ble suitable habitat classi-fied as devel-oped	Page num -ber
5	*Astragalus bernardinus*	San Bernardino milk-vetch	1,426,979	284,903	1,014	3,362	35
6	*Astragalus douglasii* var. *perstrictus*	Jacumba milk-vetch					41
7	*Astragalus lentiginosus* var. *coachellae*	Coachella Valley milk-vetch					46
8	*Astragalus nyensis*	Nye milk-vetch	57,795	40,665	-	34	51
9	*Astragalus tricarinatus*	Triple-ribbed milkvetch	250,892	87,545	-	1,183	57
10	*Atriplex argentea* var. *longitrichoma*	Pahrump orache					63
11	*Calochortus palmeri* var. *palmeri*	Palmer’s mariposa lily	51,546	7,983	442	10,375	68
12	*Calochortus striatus*	Alkali mariposa-lily	1,375,731	306,274	12,432	61,325	74
13	*Chamaesyce platysperma*	Flat-seeded spurge					80
14	*Cylindropuntia munzii*	Munz cholla					85
15	*Cymopterus deserticola*	Desert cymopterus	957,046	224,649	6,825	18,108	90
16	*Deinandra mohavensis*	Mojave tarplant	263,615	135,037	14,709	3,283	96
17	*Echinocereus engelmannii* var. *howei*	Howe's hedgehog cactus					102
1	*Eriastrum harwoodii*	Harwood's eriastrum	1,937,975	1,447,317	94,016	14,836	6
18	*Erigeron parishii*	Parish’s daisy	323,265	132,488	3,031	2,420	107

Table and figure num-ber	Scientific name	Common name	Acres of probable suitable habitat in project boundary	Acres of probable suitable habitat on BLM-managed lands	Acres of probable suitable habitat in areas prior-itized for develop-ment	Acres of proba-ble suitable habitat classi-fied as devel-oped	Page num -ber
19	*Eriogonum bifurcatum*	Forked buckwheat	390,926	323,041	1,007	636	113
20	*Eriogonum ovalifolium* var. *vineum*	Cushenberry buckwheat	46,194	37,041	634	118	119
21	*Eriophyllum mohavense*	Barstow woolly sunflower	511,175	170,661	11,544	5,693	125
22	*Erythranthe shevockii (*now *Mimulus shevockii)*	Kelso Creek monkey flower					131
23	*Eschscholzia minutiflora* ssp *twisselmannii*	Red Rock poppy	1,079,479	519,061	7,370	3,435	136
24	*Grindelia fraxinipratensis*	Ash Meadows gum-plant					142
25	*Heuchera brevistaminea*	Laguna Mountains alumroot					147
26	*Layia heterotricha*	Pale-yellow layia	22,453	3,446	1,143	502	152
27	*Linanthus maculatus*	Little San Bernardino Mountains linanthus	1,320,822	323,465	10,493	38,238	158
28	*Lupinus excubitus* var. *medius*	Mountain springs bush lupine					164
29	*Menodora spinescens* var. *mohavensis*	Mojave menodora					169
30	*Mentzelia tridentata*	Creamy blazing star	2,632,407	1,242,932	36,882	6,884	174

Table and figure num-ber	Scientific name	Common name	Acres of probable suitable habitat in project boundary	Acres of probable suitable habitat on BLM-managed lands	Acres of probable suitable habitat in areas prior-itized for develop-ment	Acres of proba-ble suitable habitat classi-fied as devel-oped	Page num -ber
31	*Mimulus mohavensis*	Mojave monkeyflower	357,907	194,086	4,085	6,500	180
32	*Monardella linoides* ssp. *oblonga*	Tehachapi monardella	26,905	7,885	447	5	186
33	*Nitrophila mohavensis*	Amargosa niterwort					192
34	*Pediomelum castoreum*	Beaver Dam breadroot	408,750	173,306	2,394	9,167	197
35	*Penstemon albomarginatus*	White-margined beardtongue	239,084	188,647	2,072	542	203
36	*Penstemon bicolor* ssp. *roseus*	Rosy two-toned beardtongue					209
37	*Perityle inyoensis*	Inyo rock daisy					214
38	*Phacelia nashiana*	Charlotte's phacelia	518,903	335,114	18,992	2,823	219
39	*Phacelia parishii*	Parish’s phacelia					225
40	*Saltugilia latimeri*	Latimer’s woodland-gilia					230
41	*Sidalcea covillei*	Owens Valley checkerbloom	225,022	88,860	3,776	7,549	235
42	*Sphaeralcea rusbyi* var. *eremicola*	Rusby's desert-mallow	749,192	212,818	-	1,058	241
43	*Xylorhiza orcuttii*	Orcutt’s woody aster	523,275	151,389	13,107	2,237	247

Tables B(1-43)_(*Species Name*). Evaluation of the model construction for existing habitat model(s) for each species. Please see Table 2 for a description of the criteria used to rate each topic as interpret with caution (red), acceptable (yellow), or ideal (green). Tables cells for which the explanation is the same as that provided for the Harwood's eriastrum case study in the body of the article have only an assigned color: red (interpret with caution), yellow (acceptable), or green (ideal). Very dark red colors in the first three occurrence topics (number, age, and spatial accuracy of occurrence data used to develop the model) indicate the model did not meet the exclusion criteria and thus is not suitable for mapping probable suitable habitat. Evidence in the organization's submitted technical report, data, and metadata was used as the basis for determining topic rankings. For most occurrence data topics (indicated by an asterisk [*]), minimum ratings were based on occurrence data through 2012 currently available in the California Natural Diversity Database [49], as all contractors used CNDDB data in model development (see Methods). Contractor C may have used additional occurrence data for some species for which we do not have detailed information that would allow evaluation. We have attempted to accommodate this uncertainty in our ratings for Contractor C in these categories.

Figs A(1-43)_(*Species Name*). Existing habitat models for each species. The legend indicates the threshold habitat suitability value that each contractor used to determine suitable habitat and the resulting number of acres of suitable habitat within the project boundary. Models produced by Contractors A, B, and C are shown in dark green, purple and blue, respectively. A model from every contractor was not available for every species.

Figs B(1-43)_(*Species Name*). Probable suitable habitat for each of the 26 species for which we were able to map probable suitable habitat. Shades of blue indicate the number of existing models predicting suitable habitat for the species. Results of the post-hoc performance evaluation using two sets of evaluation points (CNDDB occurrences from 1981-2012 and from 2013-2018) are shown on the map and in the legend, along with the area of probable suitable habitat within three boundaries: the project boundary, public lands managed by the Bureau of Land Management (BLM), and areas prioritized for development (Development Focus Areas) identified in the Desert Renewable Energy Conservation Plan [2]. Note that some areas mapped as probable suitable habitat are currently classified as developed (see Table A1).

Figs C(1-43)_(*Species Name*). Potential suitable habitat for each of the 41 species for which we were able to map potential suitable habitat to guide future plant surveys. Shades of orange indicate the number of overlapping models predicting potential suitable habitat outside of the probable suitable habitat boundary (shown in blue).

Table B1. Evaluation of the model construction for the three existing habitat models for Harwood's eriastrum (*Eriastrum harwoodii*). Please see Table 2 for a description of the criteria used to rate each topic as interpret with caution (red), acceptable (yellow), or ideal (green). Evidence in the organization's submitted report, data, and metadata was used as the basis for determining topic ratings. For most occurrence data topics (indicated by an asterisk [*]), minimum ratings were based on occurrence data through 2012 currently available in the California Natural Diversity Database [49], as all contractors used CNDDB data in model development (see Methods).

Category	Topic	Contractor A	Contractor B	Contractor C
Occurrence data used to develop the model	Number of occurrences*	Report/data indicate that model was built from 255 occurrences. Currently available CNDDB data indicate 55 occurrences were likely used by this contractor for model development.	Report/data indicate that model was built from 49 occurrences. Currently available CNDDB data indicate 56 occurrences were available for use by this contractor for model development.	Report/data indicate that model was built from 55 occurrences. Currently available CNDDB data indicate 56 occurrences were available for use by this contractor for model development.
	Age of occurrences*	Report indicates use of occurrence data from 1981-2012.	1 of 56 currently available CNDDB occurrences is from before 1981.	1 of 56 currently available CNDDB occurrences is from before 1981.
	Spatial accuracy of occurrences*	Report/data indicate occurrences with uncertainty >250-500 m were excluded.	10 of 56 (18%) currently available CNDDB occurrences have imprecise spatial accuracy.	10 of 56 (18%) currently available CNDDB occurrences have imprecise spatial accuracy.
	Status of occurrences*	11 of 56 (20%) currently available CNDDB occurrences have Fair or Poor occurrence ranks.	11 of 56 (20%) currently available CNDDB occurrences have Fair or Poor occurrence ranks.	11 of 56 (20%) currently available CNDDB occurrences have Fair or Poor occurrence ranks.
	Species identification of occurrences*	All records are from CNDDB, for which species	All records are from CNDDB, for which species	All records appear to be from CNDDB, for which species

Category	Topic	Contractor A	Contractor B	Contractor C
		identification is reliable.	identification is reliable.	identification is reliable.
	Spatial bias of occurrences*	Report indicates that records were thinned to 1 presence in each 270 m cell.	CNDDB records are separated by 250 m or more [33]. Report does not indicate any post processing of occurrence data.	CNDDB records are separated by 250 m or more [33]. Report indicates that habitat was not modeled for species with occurrence records limited to "a very small area under highly constrained habitat conditions".
	Spatial distribution of occurrences*	Currently available CNDDB records appear to be from a substantial portion of the occupied geographic subdivisions for the species in California [54].	Currently available CNDDB records appear to be from a substantial portion of the occupied geographic subdivisions for the species in California [54].	Currently available CNDDB records appear to be from a substantial portion of the occupied geographic subdivisions for the species in California [54].
	Absence data	MaxEnt randomly samples background locations.	Modeling method does not require absence data.	Modeling method does not require absence data.

Category	Topic	Contractor A	Contractor B	Contractor C
Environ-mental covariates	Ecological relevance	The report states that the following criteria were used for selecting covariates: "Significant habitat factor for ≥1 of the species; adequate resolution; available for entire study area; best available (accuracy and currency); based on ecological studies". *A team of BLM botanists determined that the selected covariate set was relevant to this species and more generally to the suite of rare plants considered in this study.*	The report states that habitat factors used were based on "life histories, specific habitat requirements, and geographic distribution of each species". *A team of BLM botanists determined that the selected covariate set was relevant to this species and more generally to the suite of rare plants considered in this study.*	The report states that there was collaboration in identifying the covariates with "BLM and team members at UC Riverside Center for Conservation Biology". *A team of BLM botanists determined that the selected covariate set was relevant to this species and more generally to the suite of rare plants considered in this study.*

Category	Topic	Contractor A	Contractor B	Contractor C
	Comprehensive	The report states that covariates are “based on available ecological studies and life history accounts for that species”. *A team of BLM botanists determined that this was a generally comprehensive set of covariates, but that surficial geology is important to some species. The effect of this omission was unknown but of concern.*	It appears from the report that the same covariates were used for all species: “spatial data for eight habitat variables, including associated vegetation types, soil texture, parent material (rock type), ecological subregion, watershed boundaries, elevation, and slope”. *A team of BLM botanists determined that this was a generally comprehensive set of covariates, but that surficial geology and climate variables capturing seasonality could be strong predictors for some species. The effects of these omissions were unknown but of concern.*	The report states that there was collaboration in identifying the covariates with “BLM and team members at UC Riverside Center for Conservation Biology”. Because of this collaboration, we assumed that the covariate data used are reasonably comprehensive. *A team of BLM botanists agreed with this conclusion.*

Category	Topic	Contractor A	Contractor B	Contractor C
	Resolution and scale	Covariates probably match temporally and thematically, but spatial resolution (270-360 m) was not justified/ explained. *A team of BLM botanists agreed that a stated rationale would have been preferable but deferred to the modeling group.*	Covariates probably match temporally and thematically, but 30 m spatial resolution was not justified/explained. *A team of BLM botanists agreed that a stated rationale would have been preferable but deferred to the modeling group.*	Covariates probably match temporally and thematically, but spatial resolution (180-250 m) was not justified/ explained. *A team of BLM botanists agreed that a stated rationale would have been preferable but deferred to the modeling group.*
	Accuracy	Most covariate datasets are from known and commonly used sources, and report indicates the accuracy of the covariate data was considered.	Although most covariate datasets are from known and commonly used sources, the report did not document any consideration of covariate data accuracy within the project boundary.	Although most covariate datasets are from known and commonly used sources, the report did not document any consideration of covariate data accuracy within the project boundary.
	Number of covariates	Model includes 12 covariates and 255 occurrences (though from ca. 55 original occurrence locations)	Model includes 19 covariates and 49 occurrences	Model includes 5 covariates and 55 occurrences; Report stated that no more than one variable per 10 occurrences was allowed.
	Current covariate data	Covariates used represent contemporary conditions (or resources such as soils and geology	Covariates used represent contemporary conditions (or resources such as soils and geology	Covariates used represent contemporary conditions (or resources such as soils and geology

Category	Topic	Contractor A	Contractor B	Contractor C
		that change very little over time).	that change very little over time).	that change very little over time).
	Covariate selection	MaxEnt weights covariates based on relationships with occurrences.	No covariate selection process – all covariates used for all species.	A covariate selection process is implied in the report, but unclear.
	Correlation	MaxEnt accommodates the use of correlated covariates [55]	Report provides no indication that correlations among covariates were considered.	Report provides no indication that correlations among covariates were considered.
Modeling algorithm	Use in the literature	MaxEnt is commonly used and accepted for species distribution modeling [55,56]	Method of using occurrence locations to inform a GIS-based overlay model is uncommon in the recent peer-reviewed literature, and the report did not provide sufficient detail to fully understand the modeling process.	Modeling method (based on Mahalanobis distance, implemented in the R package aster) is uncommon in the field of species distribution modeling, and the report did not provide sufficient detail to fully understand the modeling process.
	Interactions	MaxEnt accommodates covariate interactions.	The method is non-statistical, and thus ignores covariate interactions.	Not enough information was provided in the report to address this topic, but consideration of covariate interactions is apparently not automatic [58].

Category	Topic	Contractor A	Contractor B	Contractor C
	Non-linear	MaxEnt accommodates non-linear relationships between covariates and occurrence data.	Not enough information was provided in the report to address this topic. The modeling method could accommodate non-linear relationships, but it is unclear if it did so.	Not enough information was provided in the report to address this topic, but incorporation of non-linear relationships between covariates and occurrence data is apparently not automatic [58].
Modeling extent and resolution	Model extent	Project area included most of the area of occupied geographic subdivisions for the species in California [54].	Project area included most of the area of occupied geographic subdivisions for the species in California [54].	Project area included most of the area of occupied geographic subdivisions for the species in California [54].
	Resolution of model output	Output resolution (270-360 m) is finer than resolution of multiple covariates, and appropriate recommendations for spatial scale of use are not provided.	Output resolution (30 m) is finer than multiple covariates, and appropriate recommendations for spatial scale of use are not provided.	Output resolution (180-250 m) is finer than resolution of multiple covariates, and appropriate recommendations for spatial scale of use are not provided.

Category	Topic	Contractor A	Contractor B	Contractor C
Model selection and thresholds	Model selection	MaxEnt implements a machine-learning algorithm to determine covariate weights, but some details needed to repeat the process were not provided in the report.	A single model was built by weighting covariate values by occurrence locations. Subsequently, watersheds without documented occurrences along with developed areas and permanent water were removed. Report did not provide sufficient detail to be able to repeat the process.	Several combinations of the covariates were compared, but specific details are not provided in the report.
	Selection of threshold for mapping suitable habitat	Threshold selected based on maximum training specificity plus sensitivity [59].	Not enough information was provided in the report to address this topic.	Report indicates that they used the suitability value that "best encompassed the species known location points". The percentage of occurrences captured varied across species, so the details of this step are unclear.

Fig A1_*Eriastrum harwoodii*.

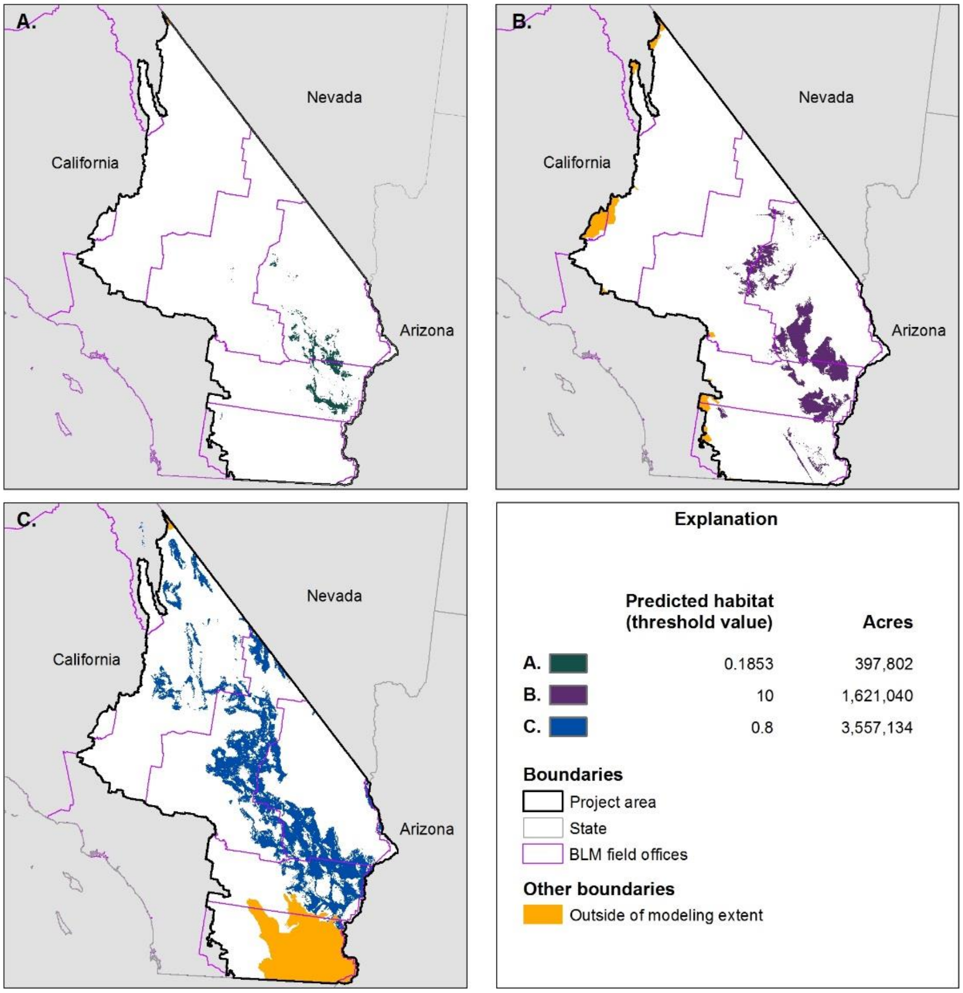

Fig B1_*Eriastrum harwoodii*.

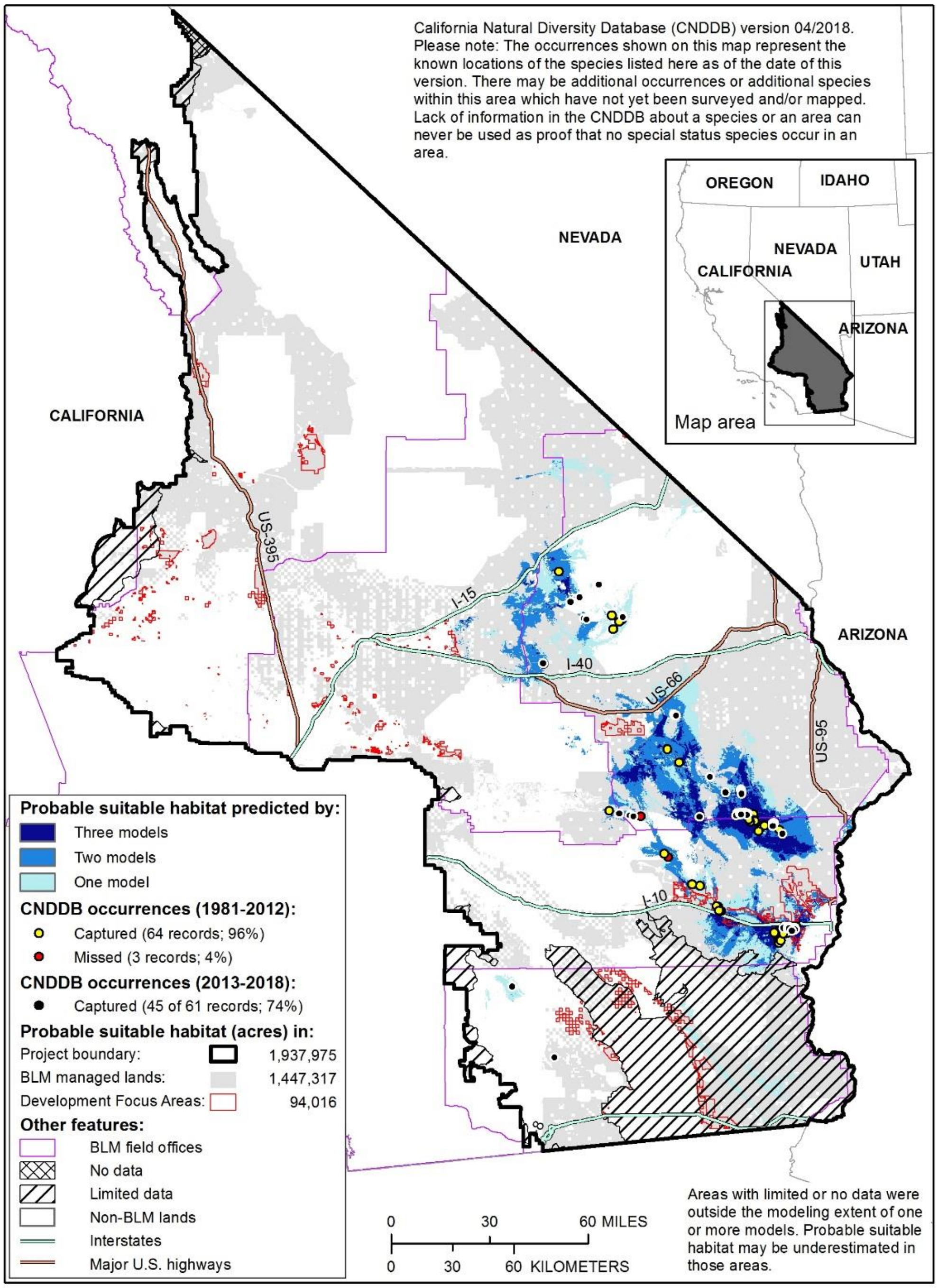

Fig C1_*Eriastrum harwoodii*.

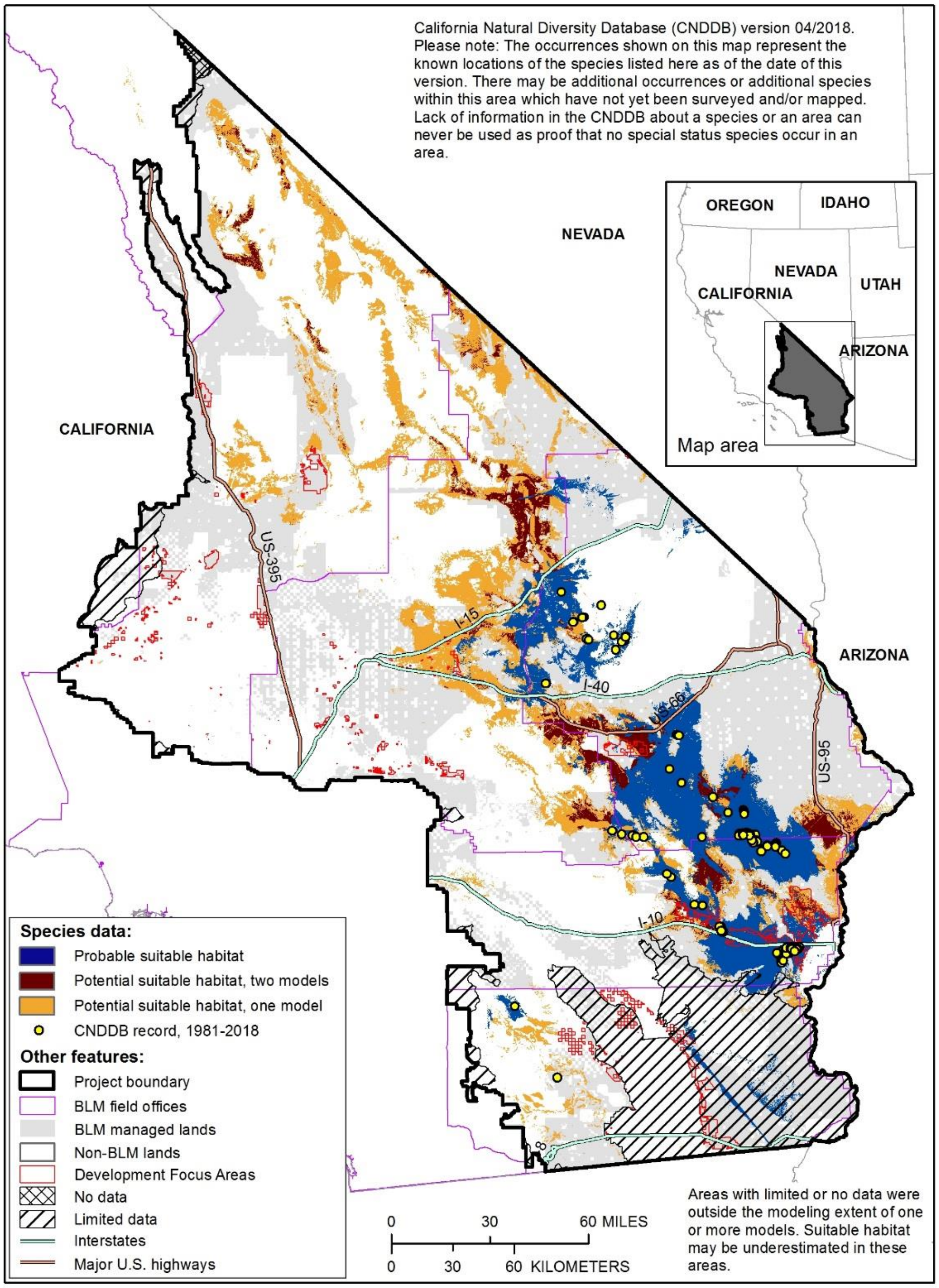

Table B2_*Abronia villosa* var. *aurita*.

Category	Topic	Contractor A	Contractor B	Contractor C
Occurrence data used to develop the model	Number of occurrences*	Report/data indicate that model was built from 32 occurrences. Currently available CNDDB data indicate 58 occurrences were likely available for use by this contractor for model development.	Report/data indicate that model was built from 24 occurrences. Currently available CNDDB data indicate 87 occurrences were likely available for use by this contractor for model development.	Report/data indicate that two regional models were built from 106 and 139 occurrences. Currently available CNDDB data indicate 87 occurrences were likely available for use by this contractor for model development.
	Age of occurrences*		29 of 87 (33%) currently available CNDDB occurrences are from prior to 1981. However, the small number of points stated in the report suggests that not all of these older locations may have been used.	29 of 87 (33%) currently available CNDDB occurrences are from prior to 1981. The large number of points stated in the report suggests that many of these older data, or other data for which dates are unknown, were used.
	Spatial accuracy of occurrences*	Report/ data indicate occurrences with uncertainty >250-500 m were excluded.	39 of 87 (45%) currently available CNDDB occurrences have imprecise spatial accuracy. However, the small number of points stated in the report suggests that not all of these records may have used.	39 of 87 (45%) currently available CNDDB occurrences have imprecise spatial accuracy. The large number of points stated in the report suggests that many of these data may have been used.

Category	Topic	Contractor A	Contractor B	Contractor C
	Status of occurrences*	12 of 58 (21%) currently available CNDDB occurrences have imprecise spatial accuracy. However, the small number of points stated in the report suggests that all of these records may not have been used.	12 of 87 (14%) currently available CNDDB occurrences have Fair or Poor occurrence ranks.	12 of 87 (14%) currently available CNDDB occurrences have Fair or Poor occurrence ranks.
	Species identification of occurrences*			18-32% of the records may have been from a source other than CNDDB, for which species identification is reliable.
	Spatial bias of occurrences*			
	Spatial distribution of occurrences*	Currently available CNDDB records within the contractor's project boundary appear to be from a substantial portion of the occupied geographic subdivisions for the species in California [54].	Currently available CNDDB records within the contractor's project boundary appear to be from a substantial portion of the occupied geographic subdivisions for the species in California [54].	Currently available CNDDB records within the contractor's project boundary appear to be from a limited portion of the occupied geographic subdivisions for the species in California [54].
	Absence data			
Environ-mental covariates	Ecological relevance			
	Comprehensive			

Category	Topic	Contractor A	Contractor B	Contractor C
	Resolution and scale			
	Accuracy			
	Number of covariates	Model includes 11 covariates and 32 occurrences	Model includes 17 covariates and 24 occurrences	Models include 6-10 covariates and 106-139 occurrences; Report stated that no more than one variable per 10 occurrences was allowed.
	Current covariate data			
	Covariate selection			
	Correlation			
Modeling algorithm	Use in the literature			
	Interactions			
	Non-linear			
Modeling extent and resolution	Model extent	Contractor's project boundary included a substantial portion of the area of occupied geographic subdivisions for the species in California [54].	Contractor's project boundary included a substantial portion of the area of occupied geographic subdivisions for the species in California [54].	Contractor's project boundary excluded a substantial portion of the area of occupied geographic subdivisions for the species in California [54].
	Resolution of model output			
Model selection	Model selection			

Category	Topic	Contractor A	Contractor B	Contractor C
and thresholds	Selection of threshold for mapping suitable habitat			

Fig A2_*Abronia villosa* var. *aurita*.

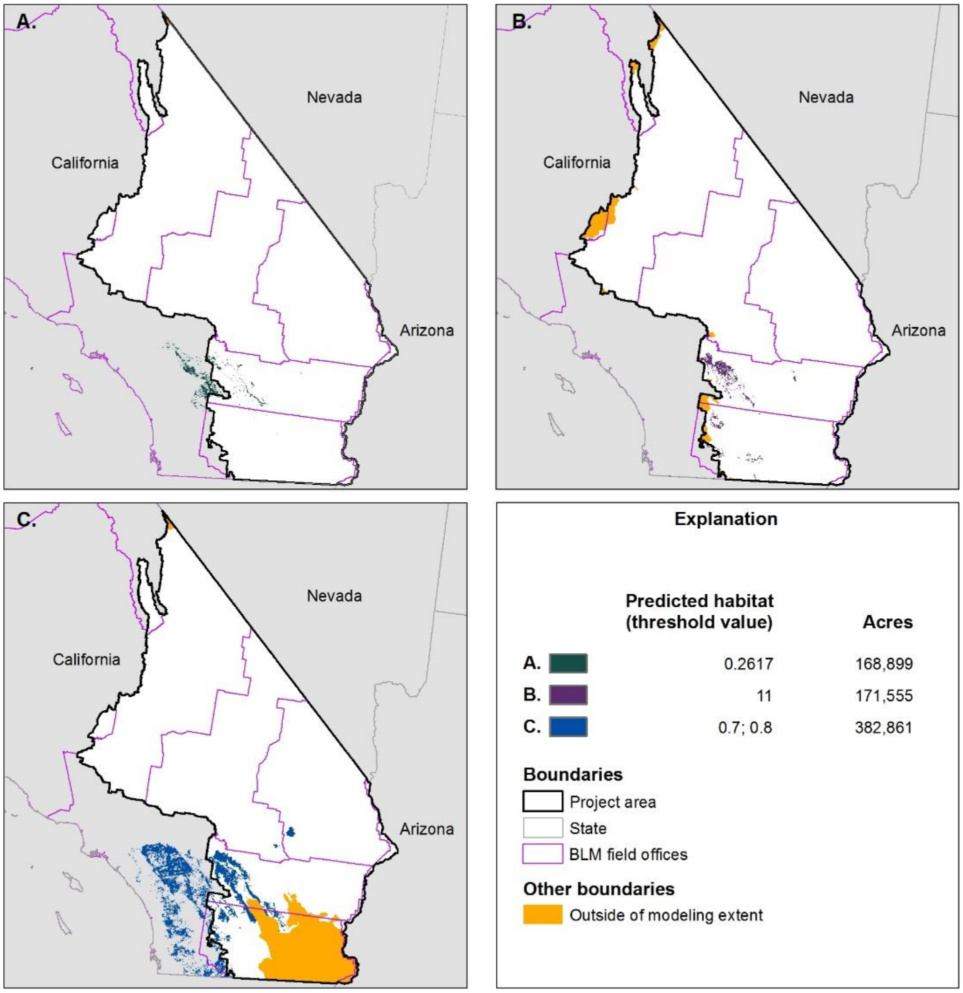

Fig B2_*Abronia villosa* var. *aurita.*

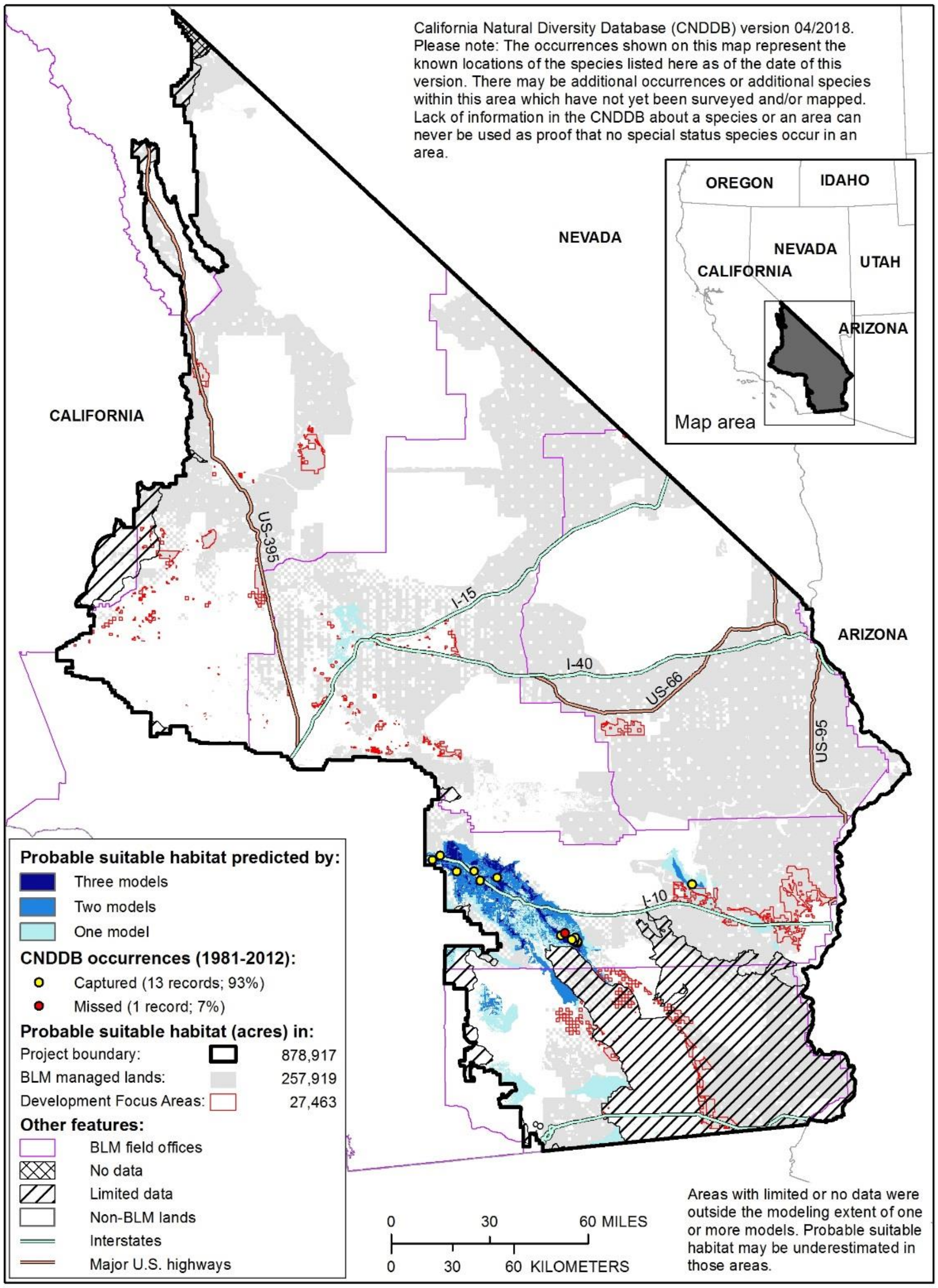

Fig C2_*Abronia villosa* var. *aurita*.

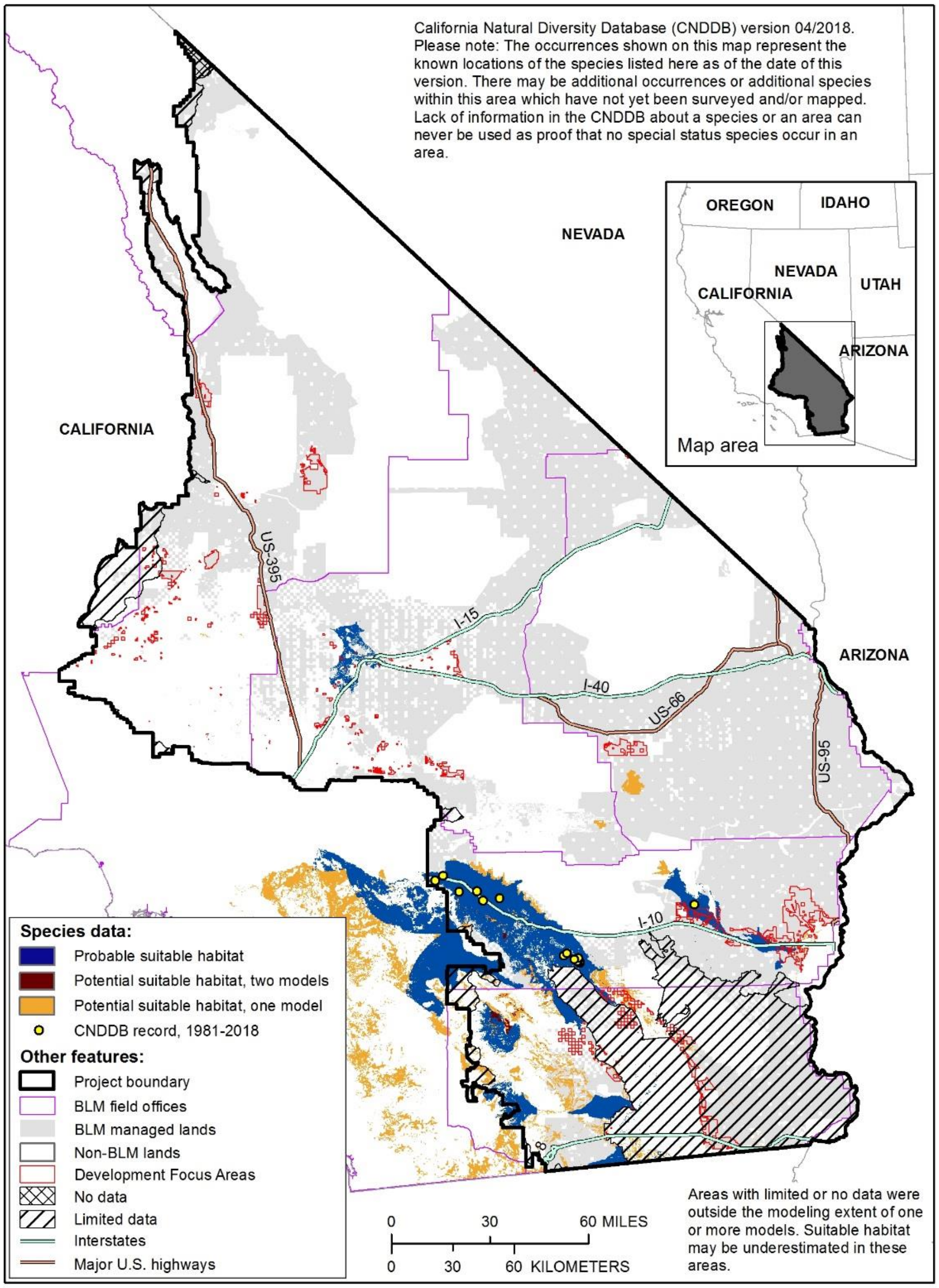

Table B3_ *Acanthoscyphus parishii* var. *goodmaniana*.

Category	Topic		Contractor B	Contractor C
Occurrence data used to develop the model	Number of occurrences*		Report/data indicate that model was built from 26 occurrences. Currently available CNDDB data indicate 22 occurrences were likely available for use by this contractor for model development.	Report/data indicate that model was built from 75 occurrences. Currently available CNDDB data indicate 22 occurrences were likely available for use by this contractor for model development.
	Age of occurrences*		0 of 22 (0%) currently available CNDDB occurrences are from prior to 1981.	0 of 22 (0%) currently available CNDDB occurrences are from prior to 1981. The larger number of points stated in the report suggests that additional data for which dates are unknown, were used.
	Spatial accuracy of occurrences*		0 of 22 (0%) currently available CNDDB occurrences have imprecise spatial accuracy.	0 of 22 (0%) currently available CNDDB occurrences have imprecise spatial accuracy. The large number of points stated in the report suggests that additional data for which accuracy is unknown, were used.

Category	Topic		Contractor B	Contractor C
	Status of occurrences*		5 of 22 (23%) currently available CNDDB occurrences have Fair or Poor occurrence ranks.	5 of 22 (23%) currently available CNDDB occurrences have Fair or Poor occurrence ranks.
	Species identification of occurrences*			53 of 75 (72%) records may have been from a source other than CNDDB, for which the reliability of species identification is unknown.
	Spatial bias of occurrences*			
	Spatial distribution of occurrences*		Currently available CNDDB records within the contractor's project boundary appear to be from a substantial portion of the occupied geographic subdivision for the species in California [54].	Currently available CNDDB records within the contractor's project boundary appear to be from a limited portion of the occupied geographic subdivision for the species in California [54].
	Absence data			
Environ-mental covariates	Ecological relevance			
	Comprehensive			
	Resolution and scale			
	Accuracy			

Category	Topic		Contractor B	Contractor C
	Number of covariates		Model includes 16 covariates and 26 occurrences	Model includes 7 covariates and 75 occurrences; Report stated that no more than one variable per 10 occurrences was allowed.
	Current covariate data			
	Covariate selection			
	Correlation			
Modeling algorithm	Use in the literature			
	Interactions			
	Non-linear			
Modeling extent and resolution	Model extent		Contractor's project boundary included most of the area of the occupied geographic subdivision for the species in California [54].	Contractor's project boundary excluded a substantial portion of the area of the occupied geographic subdivision for the species in California [54].
	Resolution of model output			
Model selection and thresholds	Model selection			
	Selection of threshold for mapping suitable habitat			

Fig A3_ *Acanthoscyphus parishii* var. *goodmaniana*.

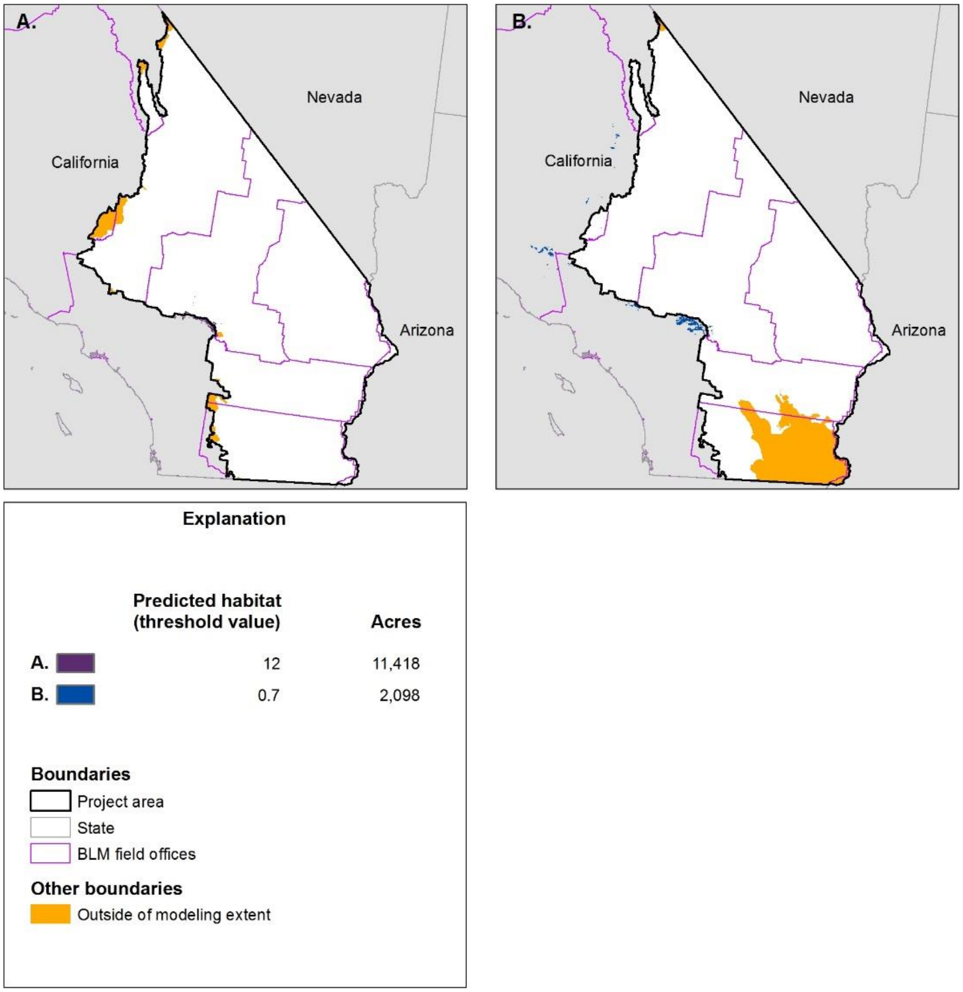

Fig B3_ *Acanthoscyphus parishii* var. *goodmaniana*.

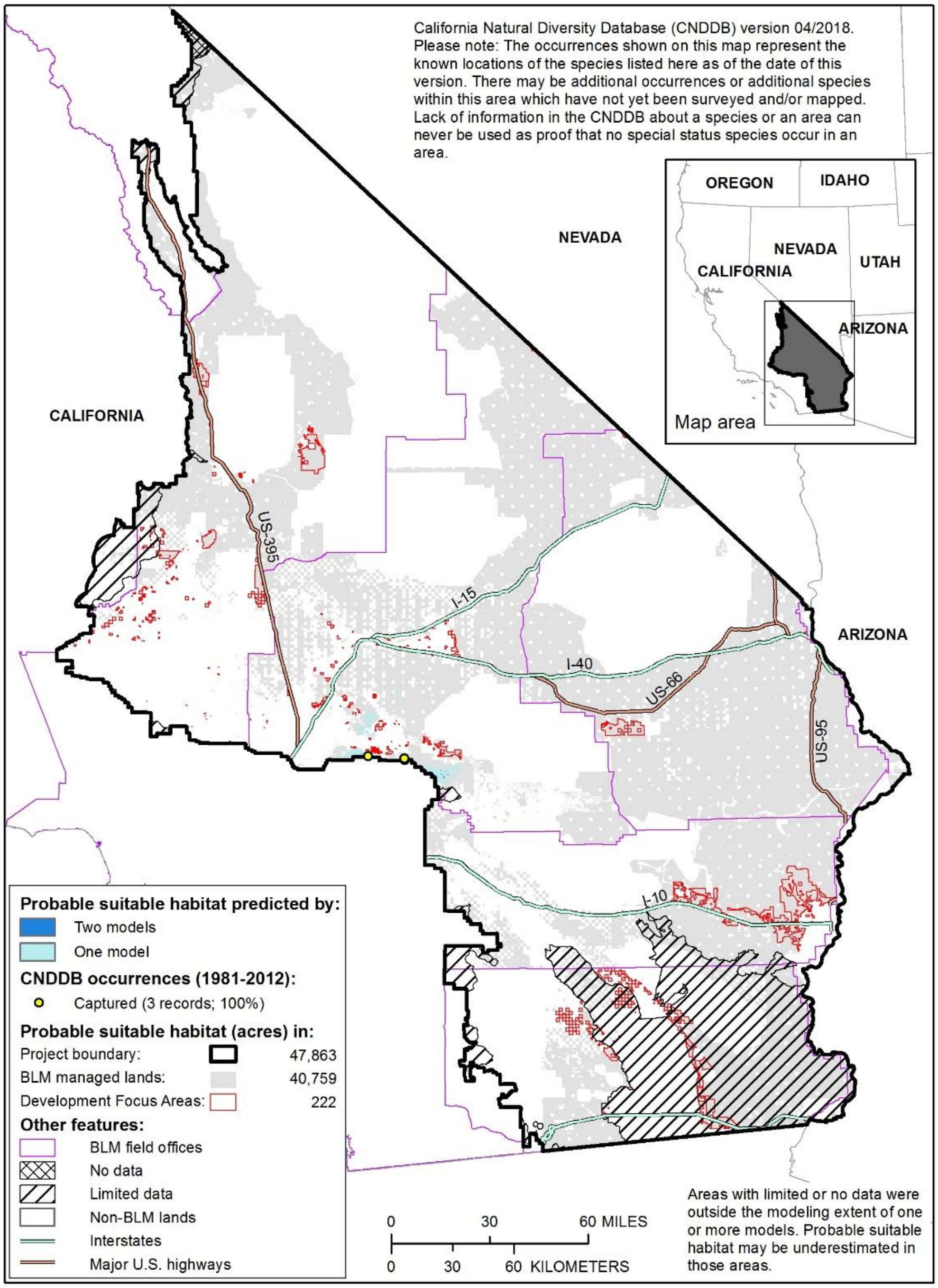

Fig C3_ *Acanthoscyphus parishii* var. *goodmaniana*.

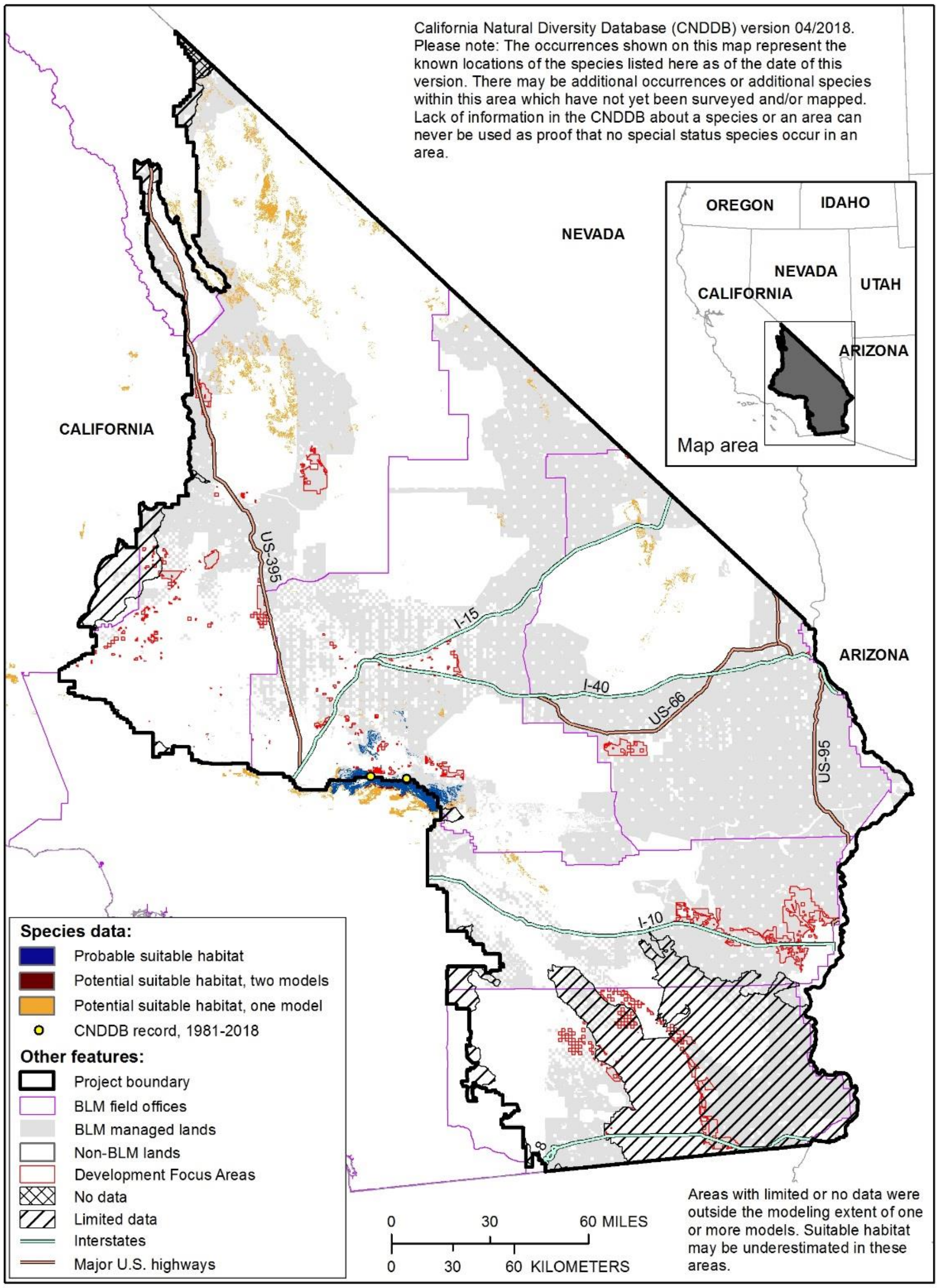

Table B4_*Allium shevockii*.

Category	Topic		Contractor B	
Occurrence data used to develop the model	Number of occurrences*		Report/data indicate that model was built from 3 occurrences. Currently available CNDDB data indicate 10 occurrences were available for use by this contractor for model development, but most are outside of the contractor's project boundary.	
	Age of occurrences*		0 of 10 (0%) currently available CNDDB occurrences is from prior to 1981.	
	Spatial accuracy of occurrences*		0 of 10 (0%) currently available CNDDB occurrences have imprecise spatial accuracy.	
	Status of occurrences*		0 of 10 (0%) currently available CNDDB occurrences have Fair or Poor occurrence ranks.	
	Species identification of occurrences*			
	Spatial bias of occurrences*			

Category	Topic		Contractor B	
	Spatial distribution of occurrences*		Currently available CNDDB records in the contractor's boundary are from a very limited portion of the area of the occupied geographic subdivisions for the species in California [54].	
	Absence data			
Environ-mental covariates	Ecological relevance			
	Comprehensive			
	Resolution and scale			
	Accuracy			
	Number of covariates		Model includes 11 covariates and 3 occurrences.	
	Current covariate data			
	Covariate selection			
	Correlation			
Modeling algorithm	Use in the literature			
	Interactions			
	Non-linear			

Category	Topic		Contractor B	
Modeling extent and resolution	Model extent		Contractor's project boundary includes very little of the area of the occupied geographic subdivisions for the species in California [54].	
	Resolution of model output			
Model selection and thresholds	Model selection			
	Selection of threshold for mapping suitable habitat			

Fig A4_*Allium shevockii*.

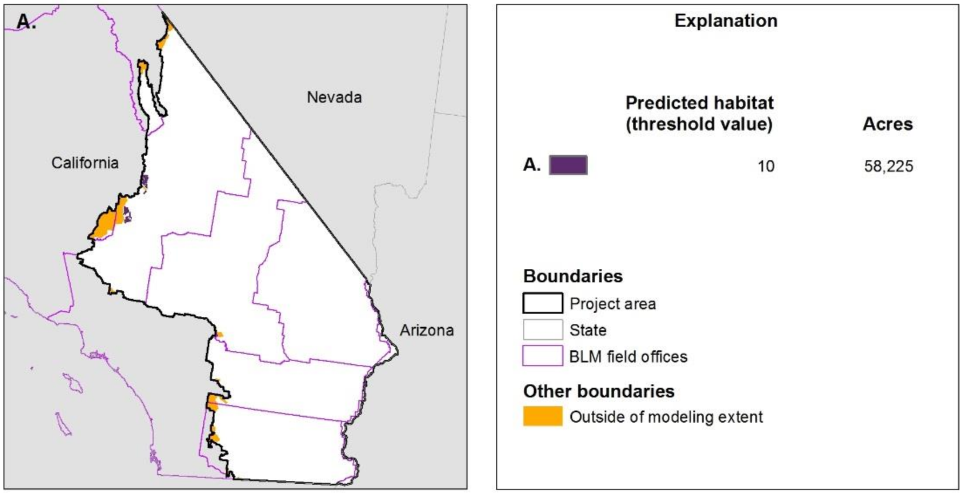

Fig C4_*Allium shevockii*.

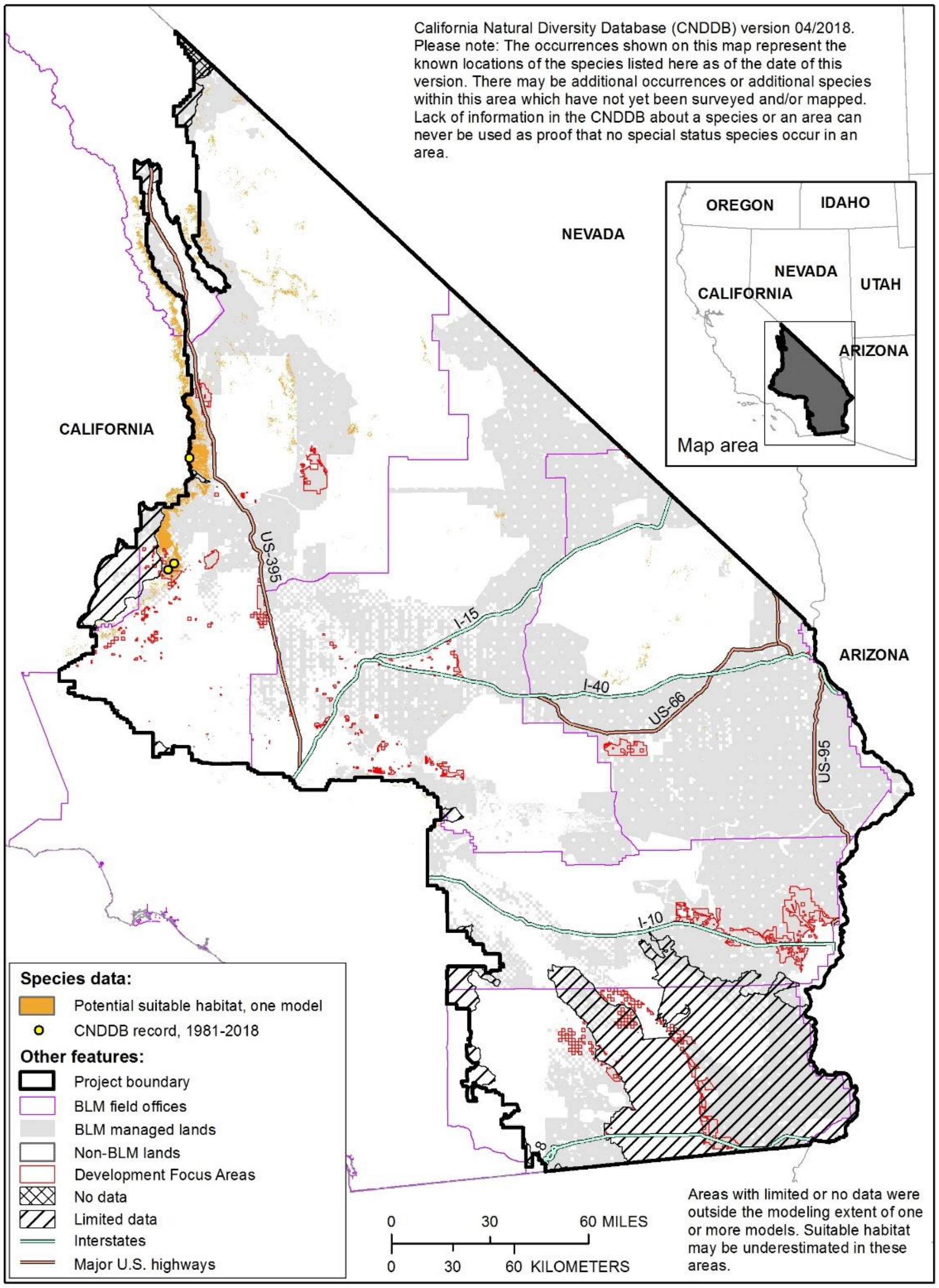

Table B5_*Astragalus bernardinus*.

Category	Topic		Contractor B	Contractor C
Occurrence data used to develop the model	Number of occurrences*		Report/data indicate that model was built from 33 occurrences. Currently available CNDDB data indicate 40 occurrences were available for use by this contractor for model development.	Report/data indicate that model was built from 47 occurrences. Currently available CNDDB data indicate 40 occurrences were available for use by this contractor for model development.
	Age of occurrences*		20 of 40 (50%) currently available CNDDB occurrences are from prior to 1981. While not all of these records may have been used, it seems likely a substantial portion of records may have been older	20 of 40 (50%) currently available CNDDB occurrences are from prior to 1981. It seems likely that many of these records were used.
	Spatial accuracy of occurrences*		27 of 40 (68%) currently available CNDDB occurrences have imprecise spatial accuracy.	27 of 40 (68%) currently available CNDDB occurrences have imprecise spatial accuracy.
	Status of occurrences*		0 of 40 (0%) currently available CNDDB occurrences have Fair or Poor occurrence ranks.	0 of 40 (0%) currently available CNDDB occurrences have Fair or Poor occurrence ranks, but none have Good or Excellent ranks.

Category	Topic		Contractor B	Contractor C
	Species identification of occurrences*			Most records appear to be from CNDDB, for which species identification is reliable.
	Spatial bias of occurrences*			
	Spatial distribution of occurrences*		Currently available CNDDB records within the contractor's project boundary appear to be from a substantial portion of the occupied geographic subdivisions for the species in California [54].	Currently available CNDDB records within the contractor's project boundary appear to be from a substantial portion of the occupied geographic subdivisions for the species in California [54].
	Absence data			
Environ-mental covariates	Ecological relevance			
	Comprehensive			
	Resolution and scale			
	Accuracy			
	Number of covariates		Model includes 16 covariates and 33 occurrences	Model includes 5 covariates and 47 occurrences; Report stated that no more than one variable per 10 occurrences was allowed.
	Current covariate data			

Category	Topic		Contractor B	Contractor C
	Covariate selection			
	Correlation			
Modeling algorithm	Use in the literature			
	Interactions			
	Non-linear			
Modeling extent and resolution	Model extent		Contractor’s project boundary included most of the area of occupied geographic subdivisions for the species in California [54].	Contractor’s project boundary included most of the area of occupied geographic subdivisions for the species in California [54].
	Resolution of model output			
Model selection and thresholds	Model selection			
	Selection of threshold for mapping suitable habitat			

Fig A5_*Astragalus bernardinus*.

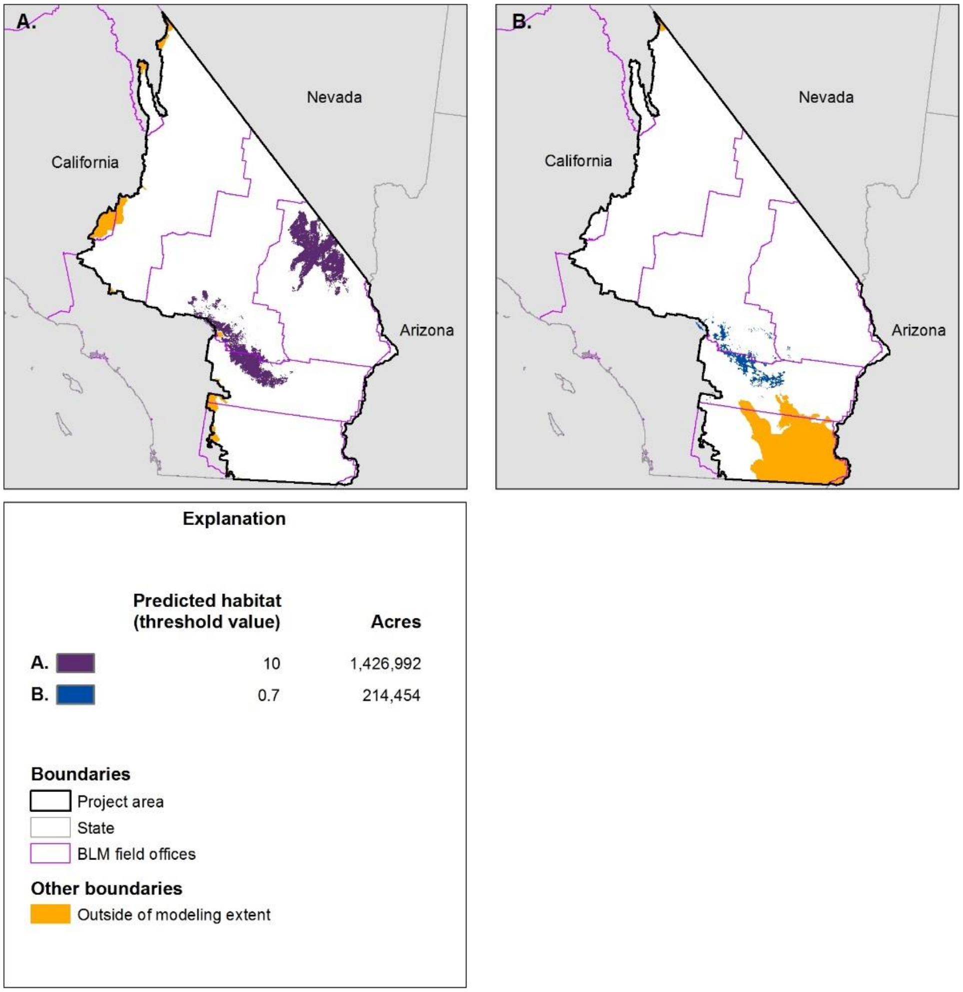

Fig B5_*Astragalus bernardinus*.

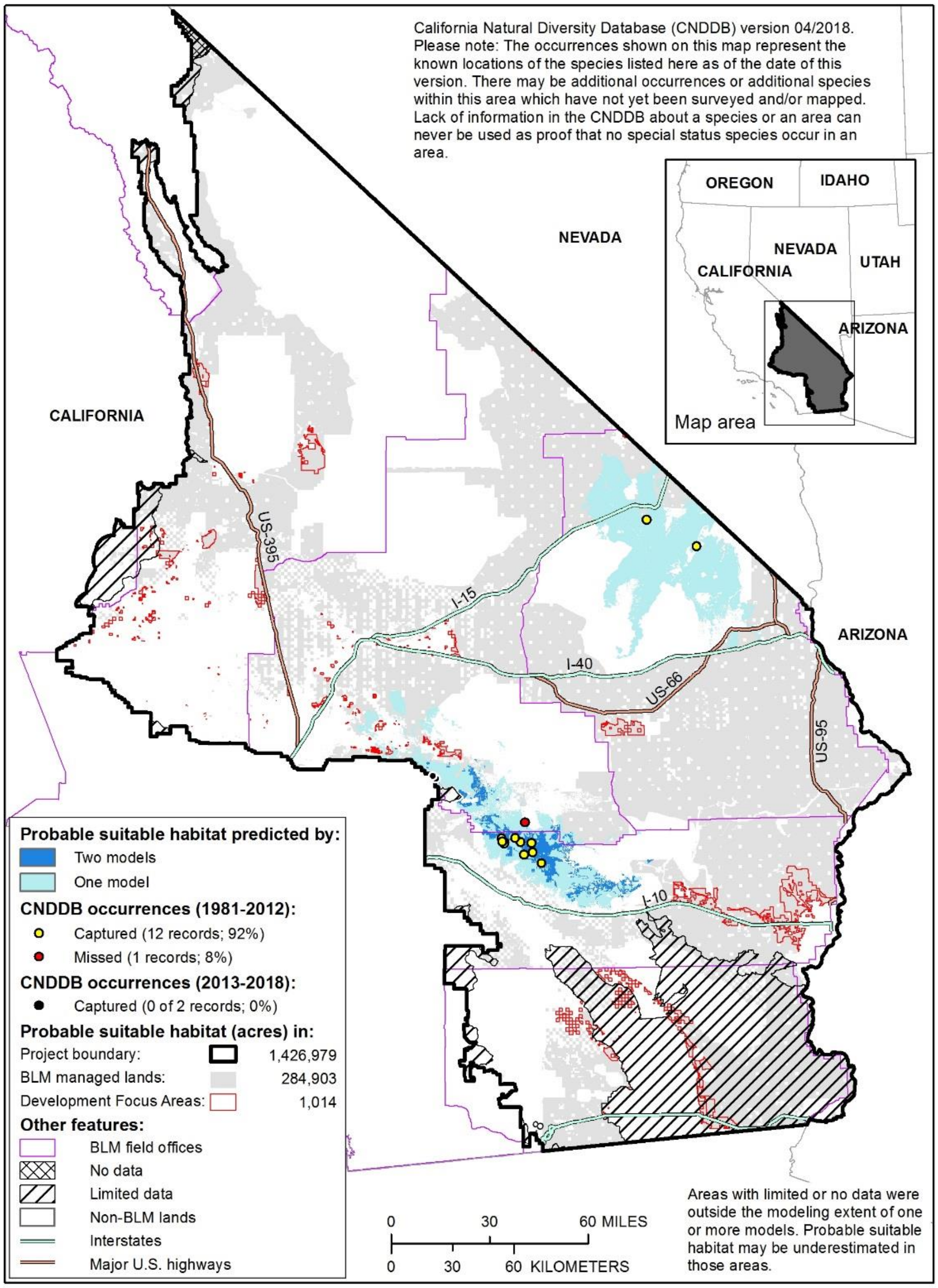

Fig C5_*Astragalus bernardinus*.

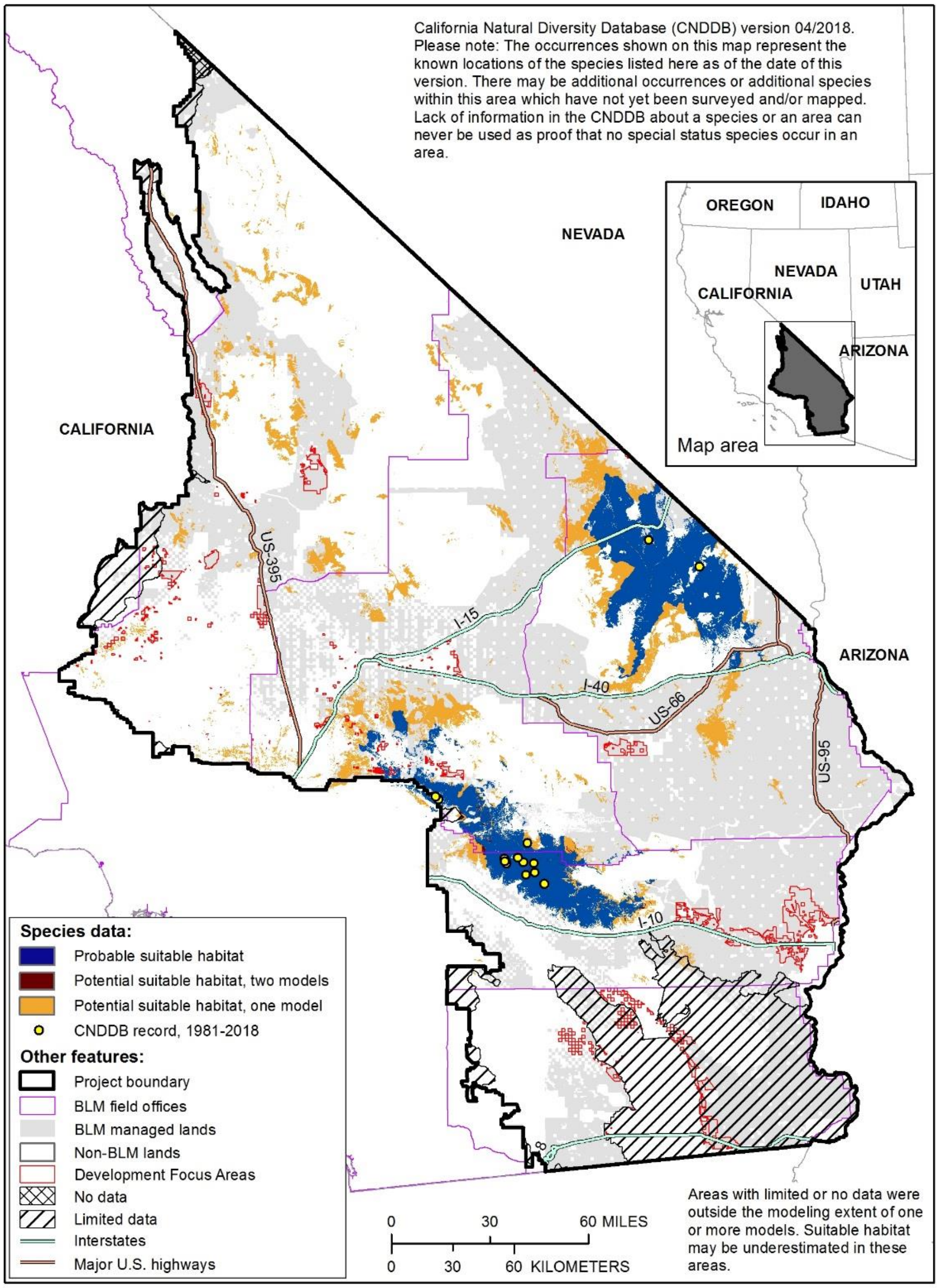

Table B6_ *Astragalus douglasii* var. *perstrictus.*

Category	Topic		Contractor B	Contractor C
Occurrence data used to develop the model	Number of occurrences*		Report/data indicate that model was built from 12 occurrences. Currently available CNDDB data indicate 52 occurrences were available for use by this contractor for model development, but nearly all are outside of the contractor's project boundary.	Report/data indicate that model was built from 53 occurrences. Currently available CNDDB data indicate 52 occurrences were available for use by this contractor for model development.
	Age of occurrences*		20 of 52 (38%) currently available CNDDB occurrences is from prior to 1981.	20 of 52 (38%) currently available CNDDB occurrences is from prior to 1981.
	Spatial accuracy of occurrences*		24 of 52 (46%) currently available CNDDB occurrences have imprecise spatial accuracy.	24 of 52 (46%) currently available CNDDB occurrences have imprecise spatial accuracy.
	Status of occurrences*		5 of 52 (10%) currently available CNDDB occurrences have Fair or Poor occurrence ranks.	5 of 52 (10%) currently available CNDDB occurrences have Fair or Poor occurrence ranks.
	Species identification of occurrences*			Nearly all records appear to be from CNDDB, for which species identification is reliable.

Category	Topic		Contractor B	Contractor C
	Spatial bias of occurrences*			
	Spatial distribution of occurrences*		Currently available CNDDB records in the contractor's boundary are from a very limited portion of the area of the occupied geographic subdivisions for the species in California [54].	Currently available CNDDB records in the contractor's boundary are from a substantial portion of the area of the occupied geographic subdivisions for the species in California [54].
	Absence data			
Environ-mental covariates	Ecological relevance			
	Comprehensive			
	Resolution and scale			
	Accuracy			
	Number of covariates		Model includes 12 covariates and 12 occurrences.	Model includes 6 covariates and 53 occurrences; report stated that no more than one variable per 10 occurrences was allowed.
	Current covariate data			
	Covariate selection			
	Correlation			
Modeling algorithm	Use in the literature			
	Interactions			

Category	Topic		Contractor B	Contractor C
	Non-linear			
Modeling extent and resolution	Model extent		Contractor's project boundary includes only a very small portion of the area of the occupied geographic subdivisions for the species in California [54].	Contractor's project boundary includes most or all of the area of the occupied geographic subdivisions for the species in California [54], but not a complete buffer around them.
	Resolution of model output			
Model selection and thresholds	Model selection			
	Selection of threshold for mapping suitable habitat			

Fig A6_ *Astragalus douglasii* var. *perstrictus.*

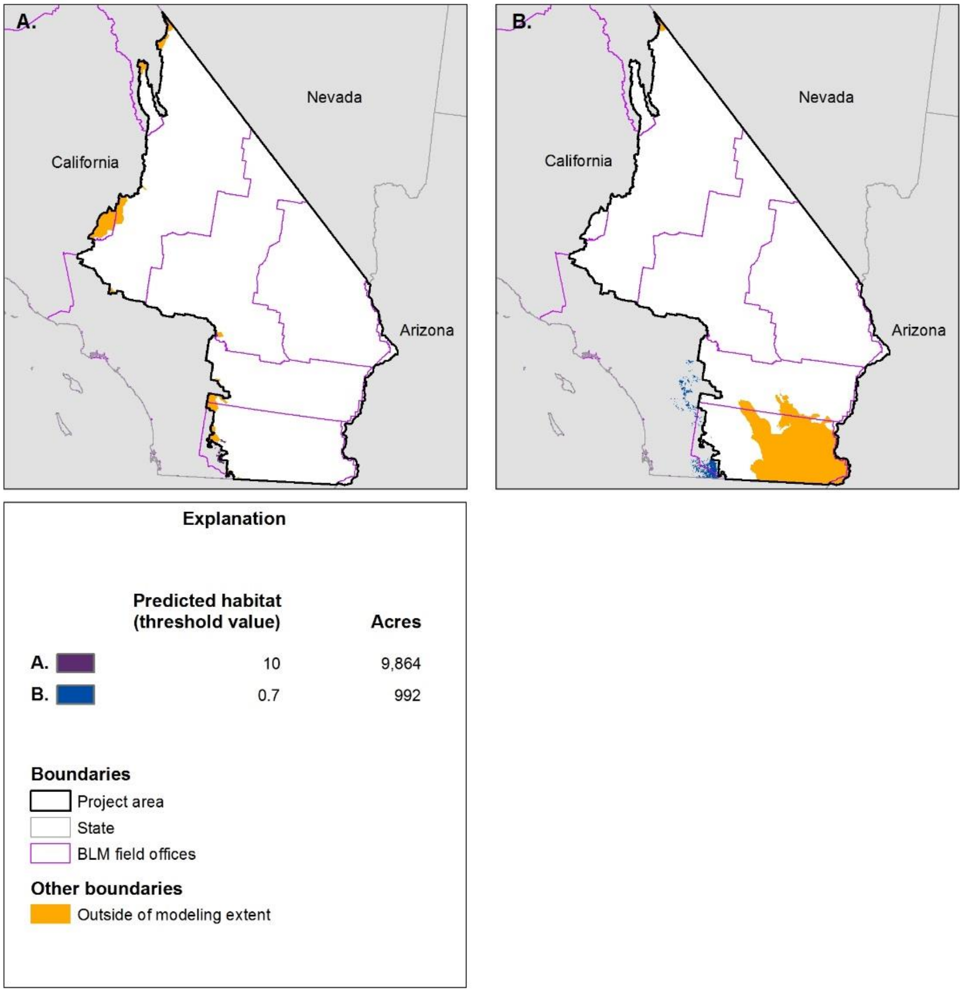

Fig C6_ *Astragalus douglasii* var. *perstrictus.*

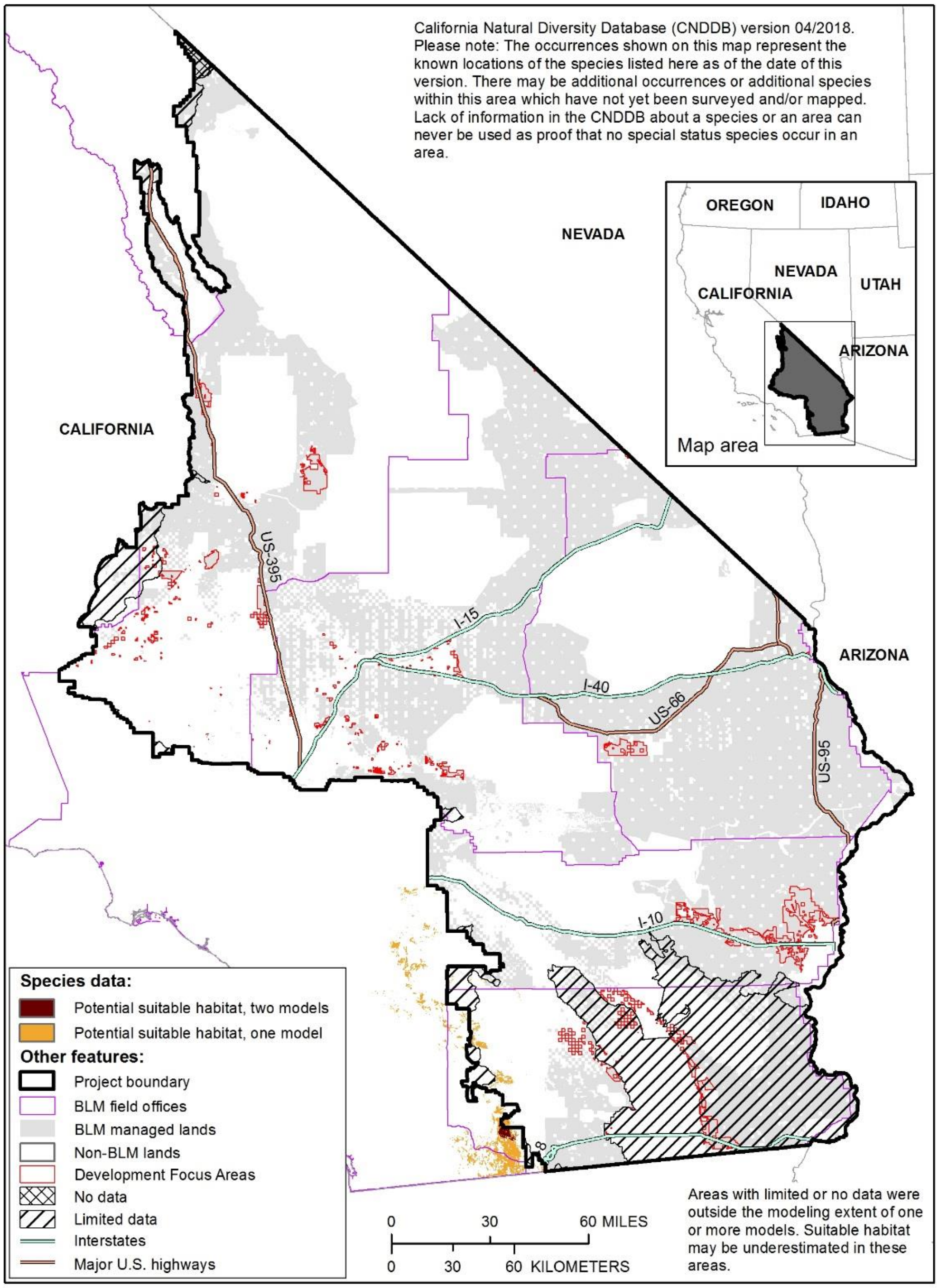

Table B7_*Astragalus lentiginosus* var. *coachellae.*

Category	Topic		Contractor B	Contractor C
Occurrence data used to develop the model	Number of occurrences*		Report/data indicate that model was built from 52 occurrences. Currently available CNDDB data indicate 44 occurrences were available for use by this contractor for model development.	Report/data indicate that model was built from 159 occurrences. Currently available CNDDB data indicate 44 occurrences were available for use by this contractor for model development.
	Age of occurrences*		5 of 44 (11%) currently available CNDDB occurrences is from prior to 1981.	5 of 44 (11%) currently available CNDDB occurrences is from prior to 1981.
	Spatial accuracy of occurrences*		19 of 44 (43%) currently available CNDDB occurrences have imprecise spatial accuracy.	19 of 44 (43%) currently available CNDDB occurrences have imprecise spatial accuracy.
	Status of occurrences*		18 of 44 (41%) currently available CNDDB occurrences have Fair or Poor occurrence ranks.	18 of 44 (41%) currently available CNDDB occurrences have Fair or Poor occurrence ranks.
	Species identification of occurrences*			A substantial portion of records appear to be from a source other than CNDDB, for which the reliability of species identification is unknown.

Category	Topic		Contractor B	Contractor C
	Spatial bias of occurrences*			
	Spatial distribution of occurrences*		Currently available CNDDB records in the contractor's boundary are from a limited portion of the area of the occupied geographic subdivision for the species in California [54].	Currently available CNDDB records in the contractor's boundary are from a limited portion of the area of the occupied geographic subdivision for the species in California [54].
	Absence data			
Environ-mental covariates	Ecological relevance			
	Comprehensive			
	Resolution and scale			
	Accuracy			
	Number of covariates		Model includes 17 covariates and 52 occurrences.	Model includes 10 covariates and 159 occurrences; report stated that no more than one variable per 10 occurrences was allowed.
	Current covariate data			
	Covariate selection			
	Correlation			
Modeling algorithm	Use in the literature			
	Interactions			

Category	Topic		Contractor B	Contractor C
	Non-linear			
Modeling extent and resolution	Model extent		Contractor’s project boundary includes most of the area of the occupied geographic subdivisions for the species in California [54], but not a complete buffer around them.	Contractor’s project boundary includes nearly all of the area of the occupied geographic subdivisions for the species in California [54], but not a complete buffer around them.
	Resolution of model output			
Model selection and thresholds	Model selection			
	Selection of threshold for mapping suitable habitat			

Fig A7_ *Astragalus lentiginosus* var. *coachellae.*

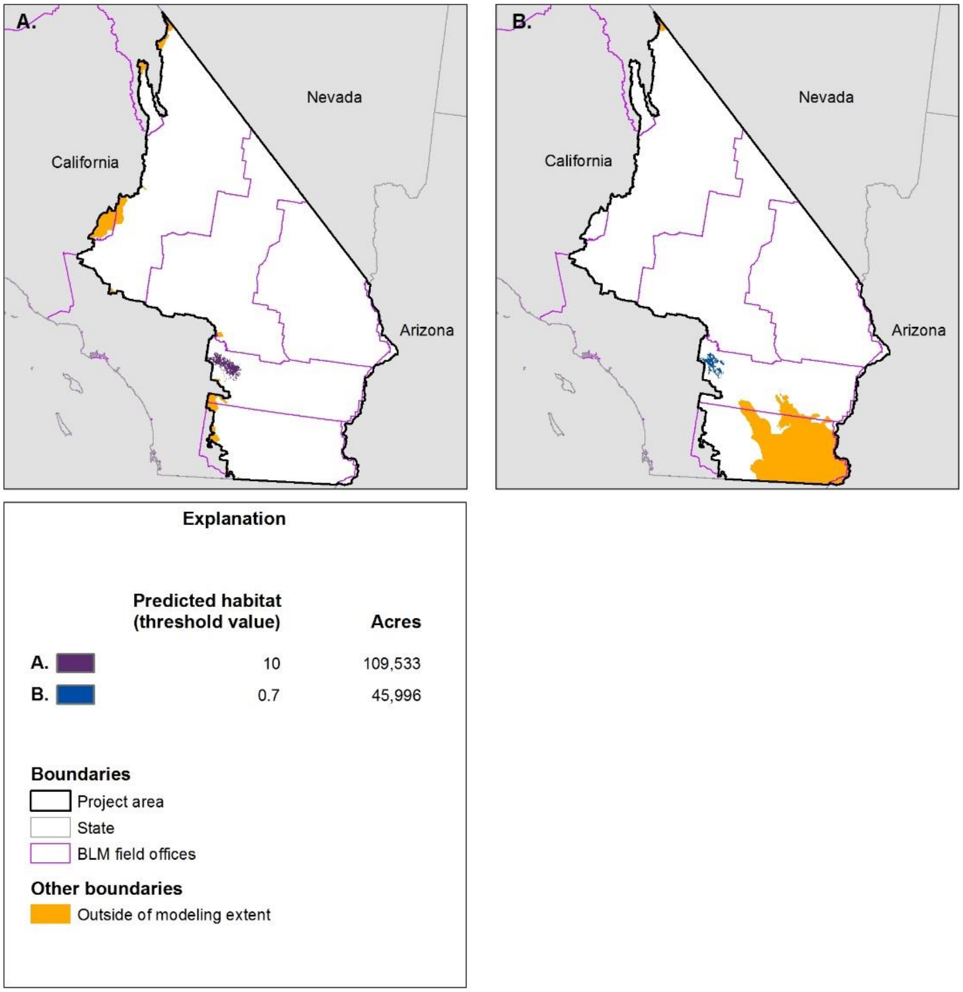

Fig C7_ *Astragalus lentiginosus* var. *coachellae.*

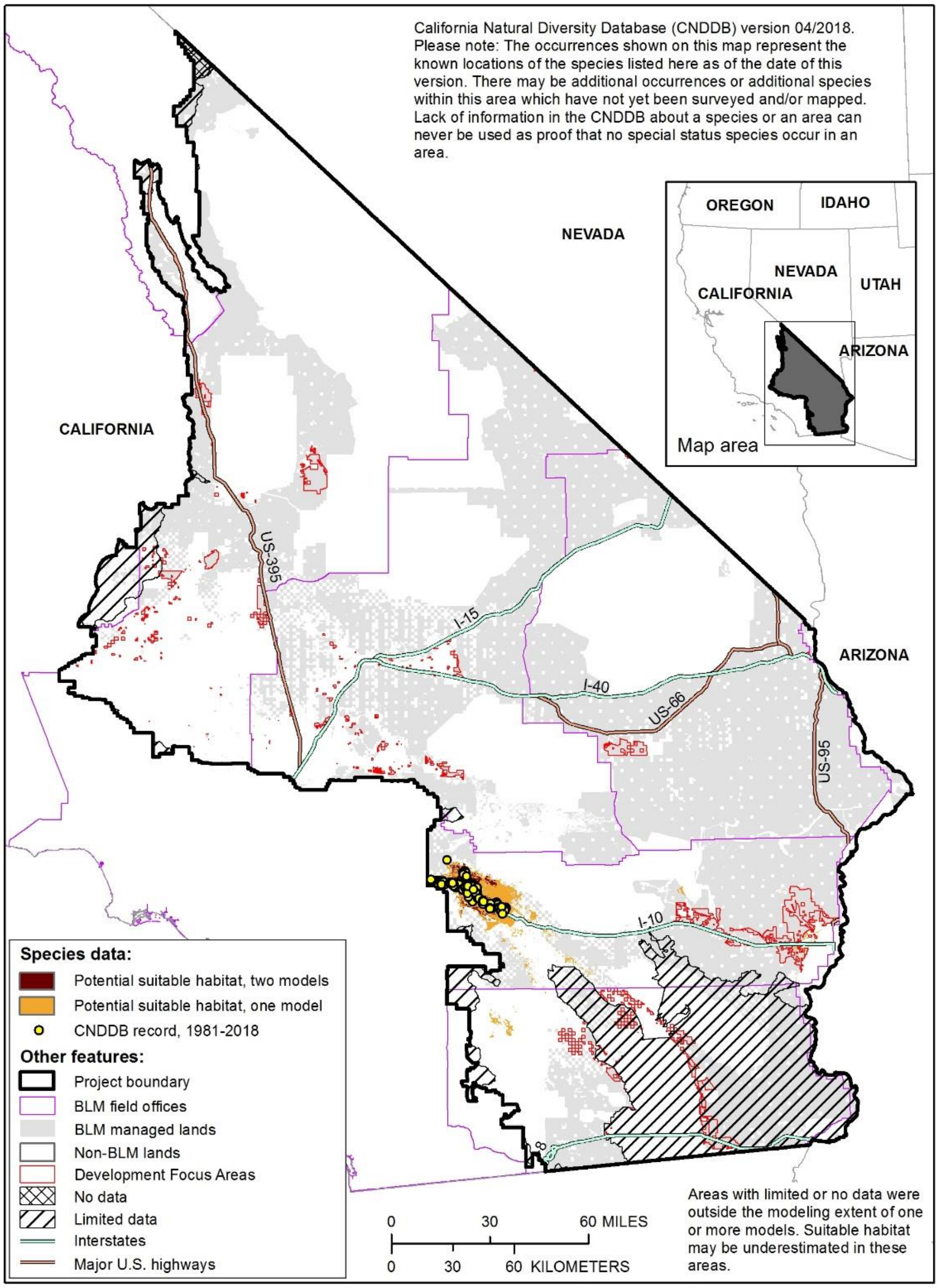

Table B8_*Astragalus nyensis*.

Category	Topic		Contractor B	
Occurrence data used to develop the model	Number of occurrences*		Report/data indicate that model was built from 19 occurrences. Currently available CNDDB data also indicate 19 occurrences were likely available for use by this contractor for model development.	
	Age of occurrences*		0 of 19 (0%) currently available CNDDB occurrences are from prior to 1981.	
	Spatial accuracy of occurrences*		0 of 19 (0%) currently available CNDDB occurrences have imprecise spatial accuracy.	
	Status of occurrences*		11 of 19 (58%) currently available CNDDB occurrences have Fair or Poor occurrence ranks.	
	Species identification of occurrences*			
	Spatial bias of occurrences*			

Category	Topic		Contractor B	
	Spatial distribution of occurrences*		This species is not listed as occurring in California [54]; most habitat is in Nevada with a small area in Utah [70]. As such, currently available CNDDB records within the contractor's project boundary are from, at most, a very limited and small area of the species' range [54].	
	Absence data			
Environ-mental covariates	Ecological relevance			
	Comprehensive			
	Resolution and scale			
	Accuracy			
	Number of covariates		Model includes 10 covariates and 19 occurrences	
	Current covariate data			
	Covariate selection			
	Correlation			
Modeling algorithm	Use in the literature			

Category	Topic		Contractor B	
	Interactions			
	Non-linear			
Modeling extent and resolution	Model extent		This species is not listed as occurring in California [54]; most habitat is in Nevada with a small area in Utah [70]. As such, the model extent included at most the fringe of the range for this species which is problematic.	
	Resolution of model output			
Model selection and thresholds	Model selection			
	Selection of threshold for mapping suitable habitat			

Fig A8_ *Astragalus nyensis*.

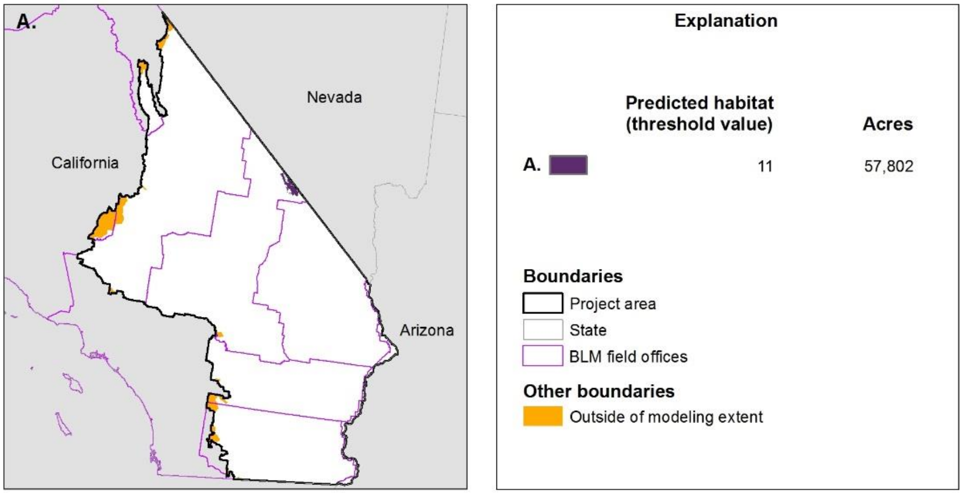

Fig B8_ *Astragalus nyensis*.

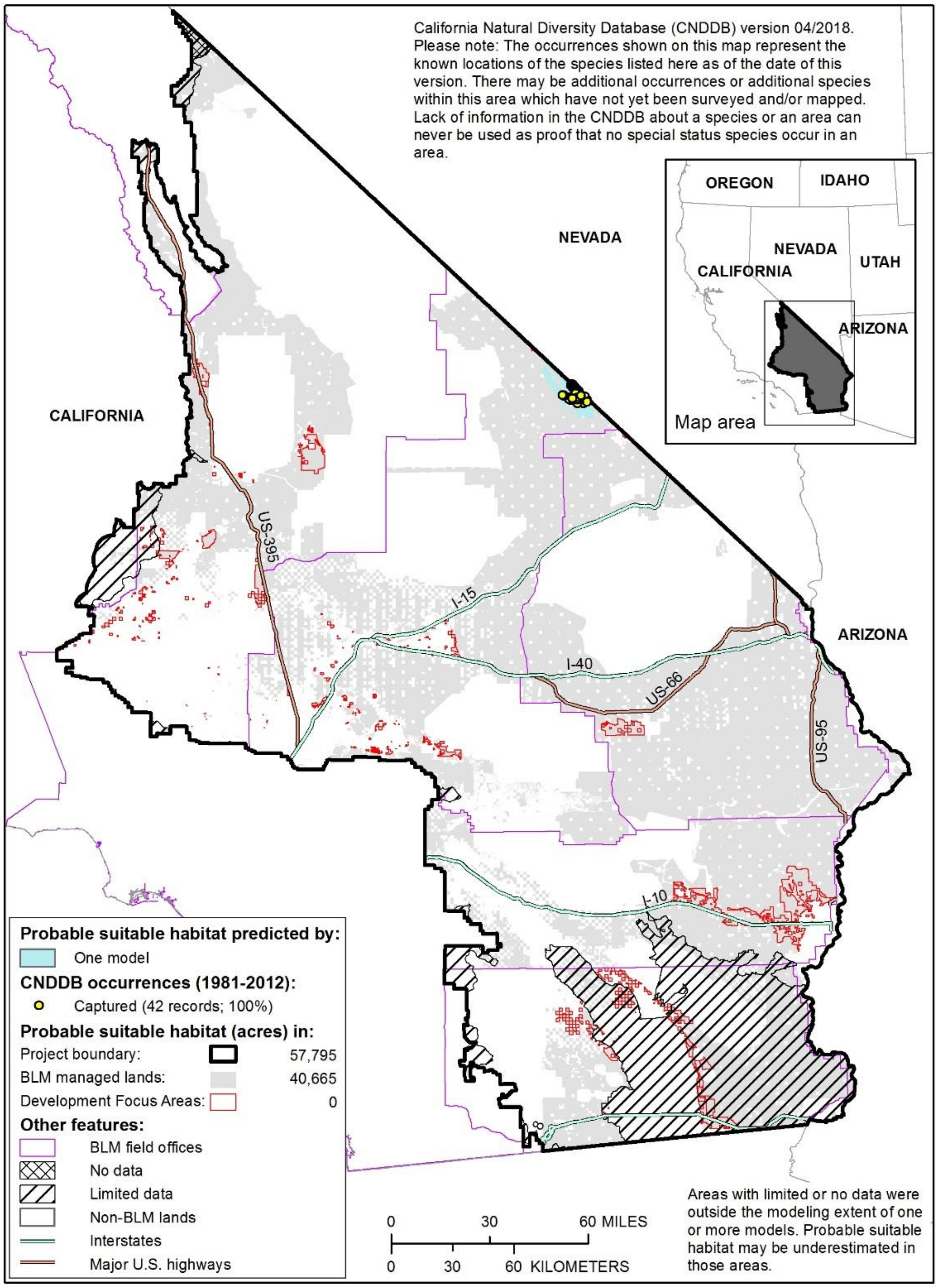

Fig C8_ *Astragalus nyensis*.

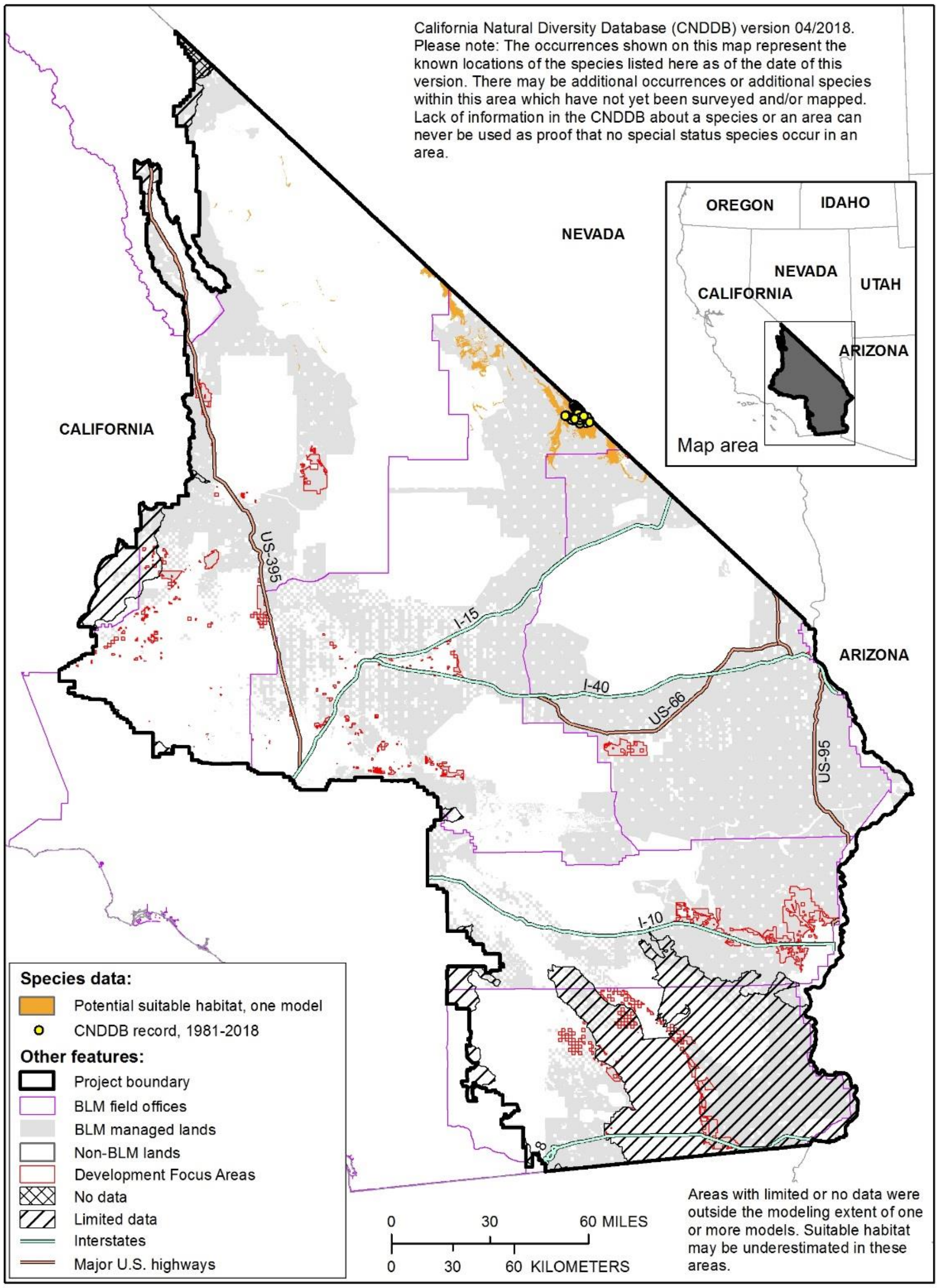

Table B9_ *Astragalus tricarinatus*.

Category	Topic	Contractor A	Contractor B	Contractor C
Occurrence data used to develop the model	Number of occurrences*	Report/data indicate that model was built from 90 occurrences. Currently available CNDDB data indicate 34 occurrences were likely used by this contractor for model development.	Report/data indicate that model was built from 37 occurrences. Currently available CNDDB data indicate 36 occurrences were available for use by this contractor for model development.	Report/data indicate that model was built from 82 occurrences. Currently available CNDDB data indicate 36 occurrences were available for use by this contractor for model development.
	Age of occurrences*	Report indicates use of occurrence data from 1981-2012. 3 records are from prior to 2000.	2 of 36 (6%) currently available CNDDB occurrences are from prior to 1981.	2 of 36 (6%) currently available CNDDB occurrences are from prior to 1981.
	Spatial accuracy of occurrences*	Report/data indicate occurrences with uncertainty >250-500 m were excluded.	5 of 36 (14%) currently available CNDDB occurrences have imprecise spatial accuracy.	5 of 36 (14%) currently available CNDDB occurrences have imprecise spatial accuracy.
	Status of occurrences*	16 of 34 (47%) currently available CNDDB occurrences have Fair or Poor occurrence ranks.	16 of 36 (44%) currently available CNDDB occurrences have Fair or Poor occurrence ranks.	16 of 36 (44%) currently available CNDDB occurrences have Fair or Poor occurrence ranks.
	Species identification of occurrences*			A substantial portion of records appear to be from a sources other than CNDDB, for which the reliability of species identification is unknown.

Category	Topic	Contractor A	Contractor B	Contractor C
	Spatial bias of occurrences*			
	Spatial distribution of occurrences*	Currently available CNDDB records appear to be from a limited portion of the occupied geographic subdivisions for the species in California [54].	Currently available CNDDB records appear to be from a limited portion of the occupied geographic subdivisions for the species in California [54].	Currently available CNDDB records appear to be from a limited portion of the occupied geographic subdivisions for the species in California [54].
	Absence data			
Environ-mental covariates	Ecological relevance			
	Comprehensive			
	Resolution and scale			
	Accuracy			
	Number of covariates	Model includes 7 covariates and 90 occurrences.	Model includes 17 covariates and 37 occurrences.	Model includes 7 covariates and 82 occurrences; report stated that no more than one variable per 10 occurrences was allowed.
	Current covariate data			
	Covariate selection			
	Correlation			
Modeling algorithm	Use in the literature			
	Interactions			
	Non-linear			

Category	Topic	Contractor A	Contractor B	Contractor C
Modeling extent and resolution	Model extent	Contractor’s project boundary included all of the area of occupied geographic subdivisions for the species in California [54], but the buffer did not extent to all sides.	Contractor’s project boundary included most of the area of occupied geographic subdivisions for the species in California [54], but not a buffer.	Contractor’s project boundary included all of the area of occupied geographic subdivisions for the species in California [54], but the buffer did not extent to all sides.
	Resolution of model output			
Model selection and thresholds	Model selection			
	Selection of threshold for mapping suitable habitat			

Fig A9_ *Astragalus tricarinatus*.

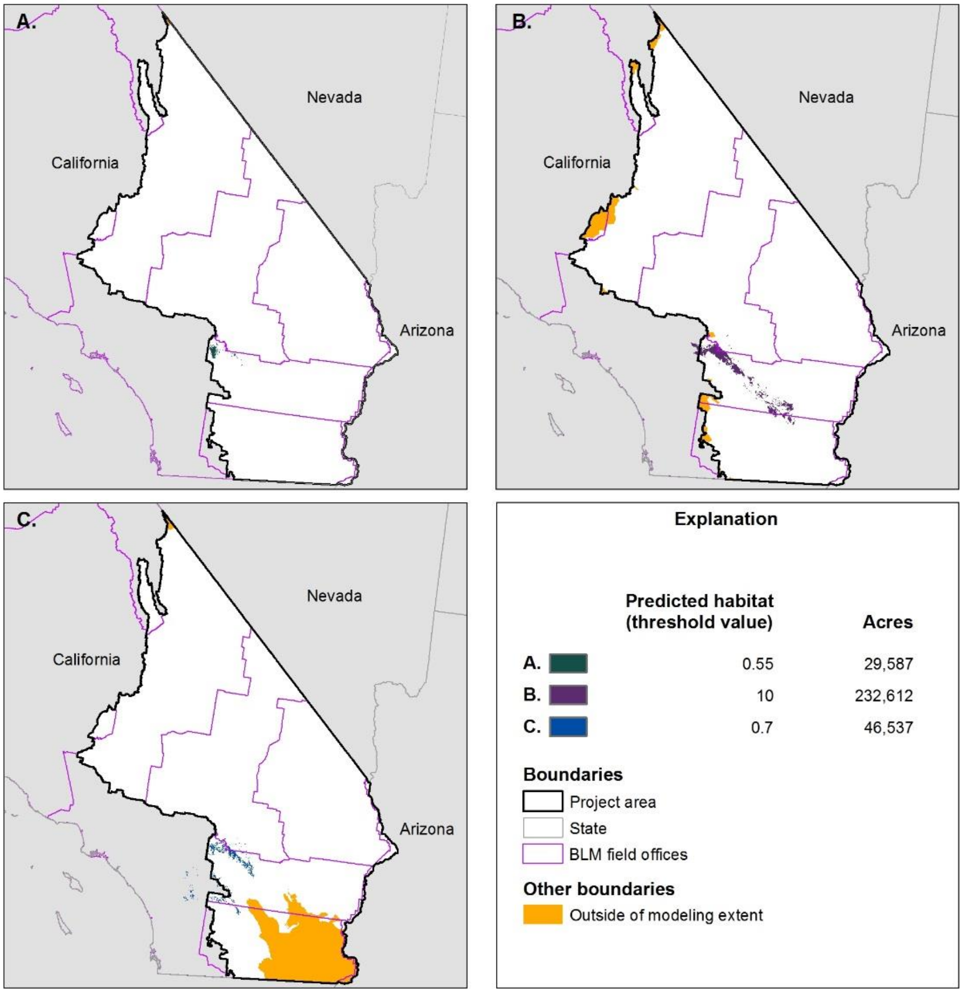

Fig B9_ *Astragalus tricarinatus*.

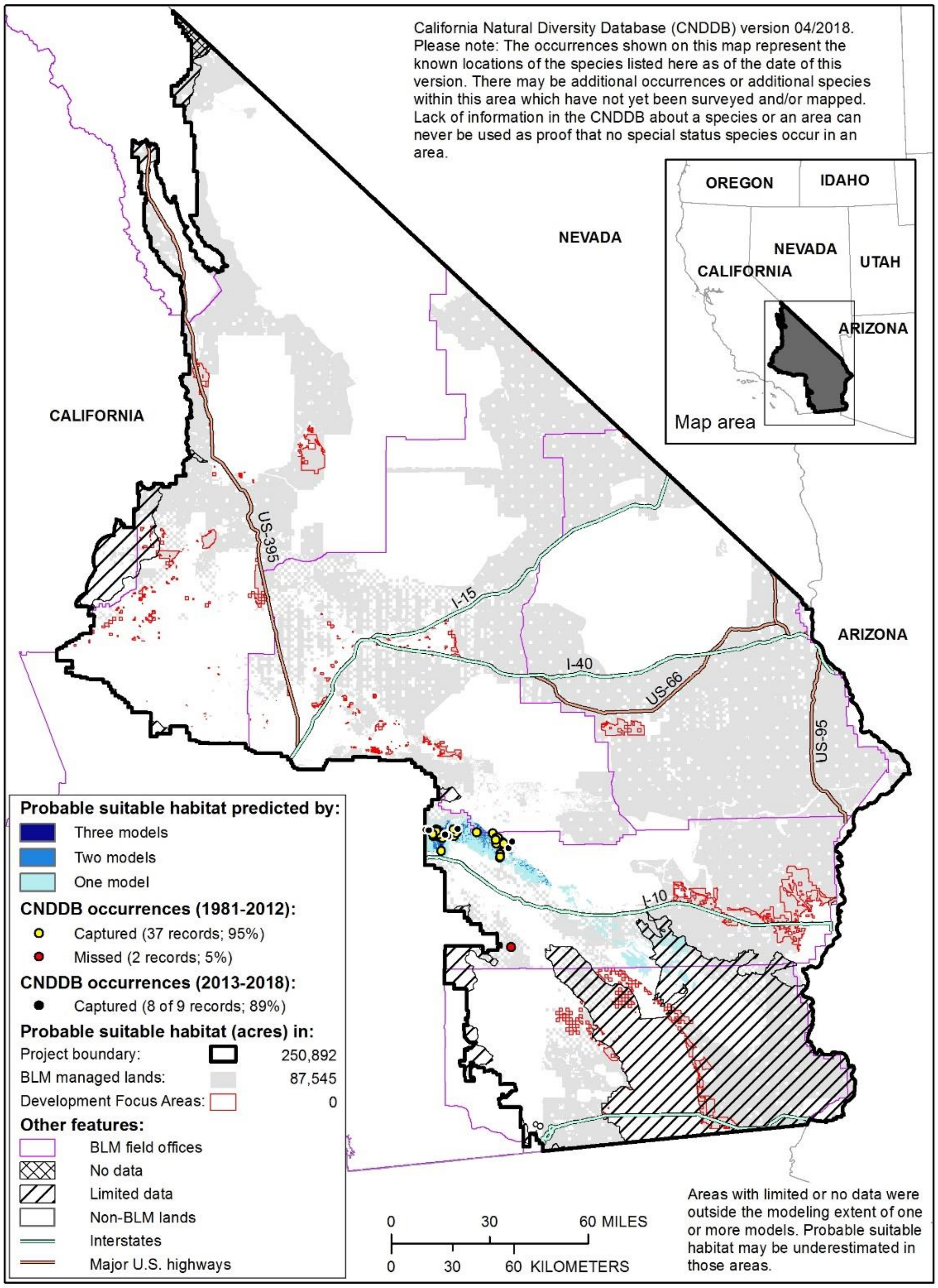

Fig C9_ *Astragalus tricarinatus*.

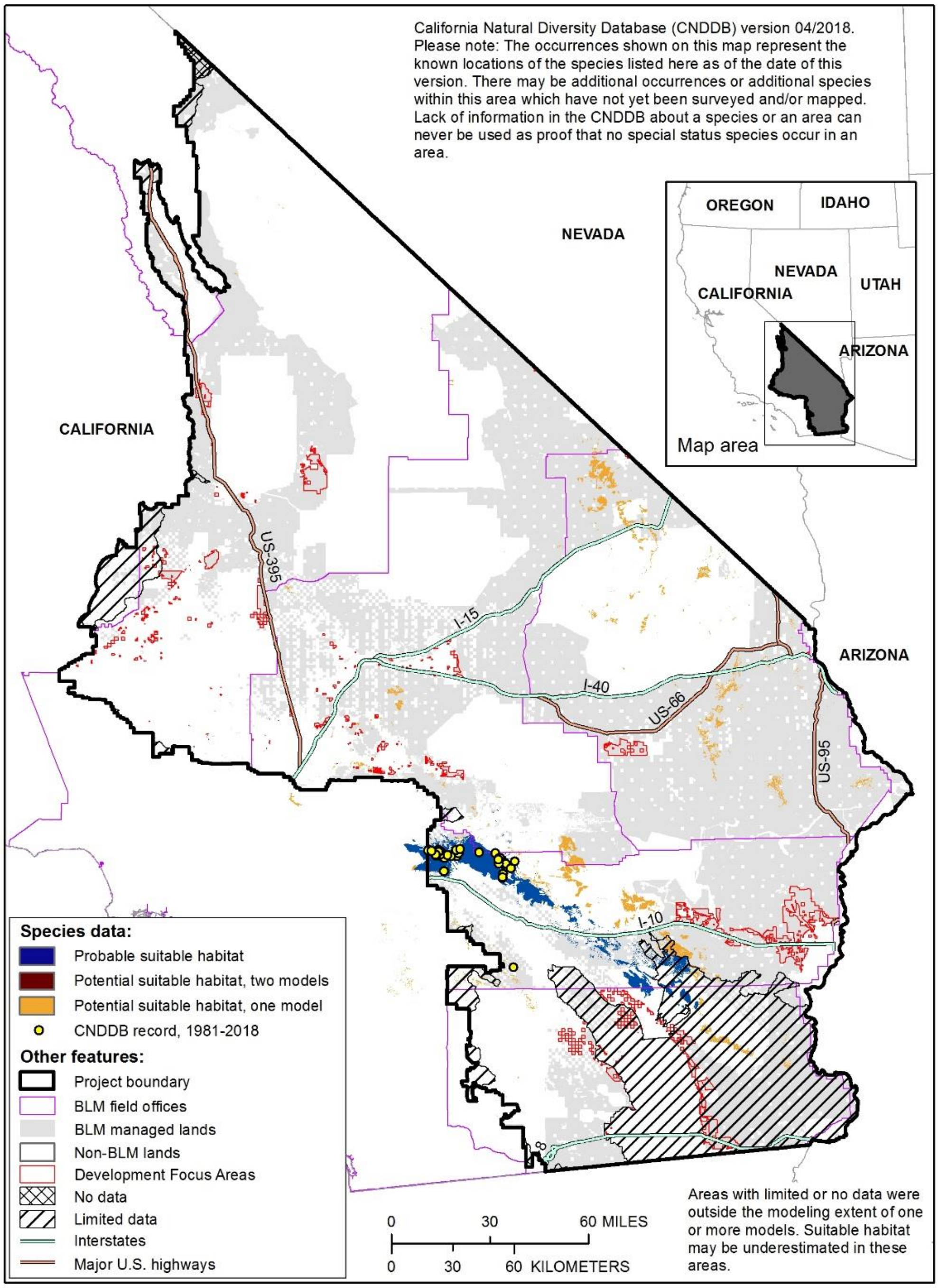

Table B10_*Atriplex argentea* var. *longitrichoma*.

Category	Topic		Contractor B	
Occurrence data used to develop the model	Number of occurrences*		Report/data indicate that model was built from 7 occurrences. Currently available CNDDB data indicate 7 occurrences were available for use by this contractor for model development.	
	Age of occurrences*		0 of 7 (0%) currently available CNDDB occurrences is from prior to 1981.	
	Spatial accuracy of occurrences*		0 of 7 (0%) currently available CNDDB occurrences have imprecise spatial accuracy.	
	Status of occurrences*		0 of 7 (0%) currently available CNDDB occurrences have Fair or Poor occurrence ranks.	
	Species identification of occurrences*			
	Spatial bias of occurrences*			

Category	Topic		Contractor B	
	Spatial distribution of occurrences*		Currently available CNDDB records in the contractor’s boundary are from a very limited portion of the area of the occupied geographic subdivision for the species in California [54].	
	Absence data			
Environ-mental covariates	Ecological relevance			
	Comprehensive			
	Resolution and scale			
	Accuracy			
	Number of covariates		Model includes 10 covariates and 7 occurrences.	
	Current covariate data			
	Covariate selection			
	Correlation			
Modeling algorithm	Use in the literature			
	Interactions			
	Non-linear			

Category	Topic		Contractor B	
Modeling extent and resolution	Model extent		Contractor’s project boundary includes most of the area of the occupied geographic subdivisions for the species in California [54], but not a complete buffer around them.	
	Resolution of model output			
Model selection and thresholds	Model selection			
	Selection of threshold for mapping suitable habitat			

Fig A10_*Atriplex argentea* var. *longitrichoma*.

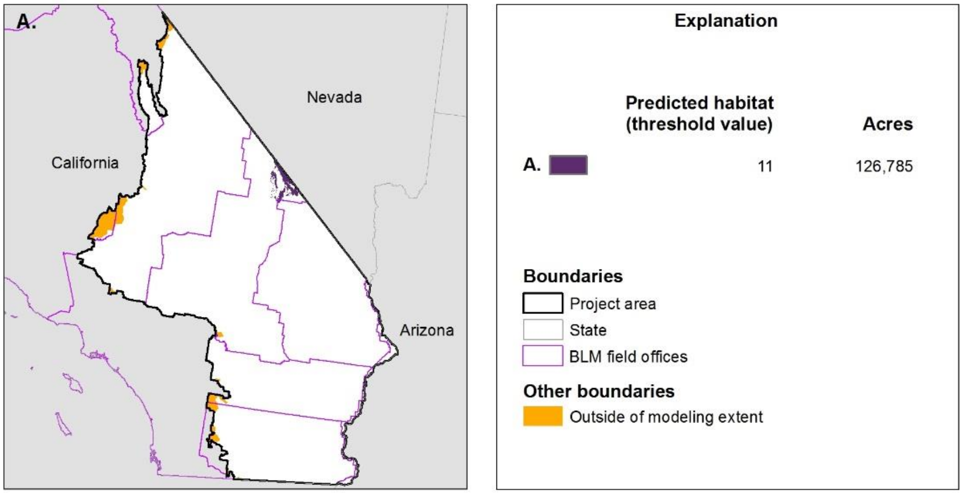

Fig C10_*Atriplex argentea* var. *longitrichoma*.

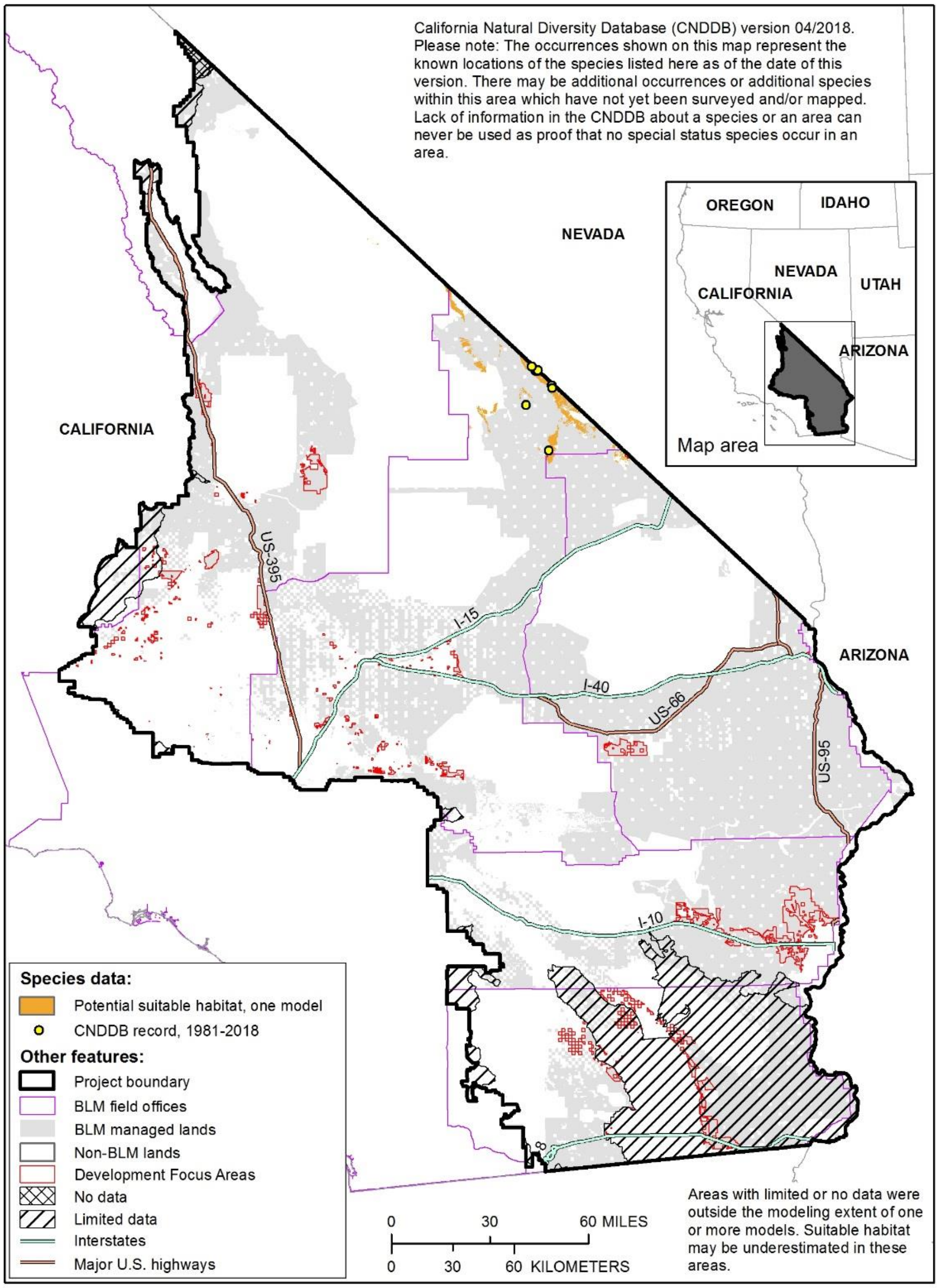

Table B11_*Calochortus palmeri* var. *palmeri*.

Category	Topic		Contractor B	Contractor C
Occurrence data used to develop the model	Number of occurrences*		Report/data indicate that model was built from 19 occurrences. Currently available CNDDB data indicate 111 occurrences were available for use by this contractor for model development, but many were outside of the contractor's project boundary.	Report/data indicate that model was built from 102 occurrences. Currently available CNDDB data indicate 111 occurrences were available for use by this contractor for model development.
	Age of occurrences*		12 of 111 (11%) currently available CNDDB occurrences are from prior to 1981.	12 of 111 (11%) currently available CNDDB occurrences are from prior to 1981.
	Spatial accuracy of occurrences*		24 of 111 (22%) currently available CNDDB occurrences have imprecise spatial accuracy, but many of these are outside of the contractor's boundary.	24 of 111 (22%) currently available CNDDB occurrences have imprecise spatial accuracy.
	Status of occurrences*		12 of 111 (11%) currently available CNDDB occurrences have Fair or Poor occurrence ranks.	12 of 111 (11%) currently available CNDDB occurrences have Fair or Poor occurrence ranks.
	Species identification of occurrences*			Most but not all records appear to be from CNDDB, for which species

Category	Topic		Contractor B	Contractor C
				identification is reliable.
	Spatial bias of occurrences*			
	Spatial distribution of occurrences*		Currently available CNDDB records within the contractor's project boundary are from a limited portion of the occupied geographic subdivisions for the species in California [54].	Currently available CNDDB records within the contractor's project boundary are from a substantial portion of the occupied geographic subdivisions for the species in California [54].
	Absence data			
Environ-mental covariates	Ecological relevance			
	Comprehensive			
	Resolution and scale			
	Accuracy			
	Number of covariates		Model includes 16 covariates and 19 occurrences.	Model includes 5 covariates and 102 occurrences; report stated that no more than one variable per 10 occurrences was allowed.
	Current covariate data			
	Covariate selection			
	Correlation			

Category	Topic		Contractor B	Contractor C
Modeling algorithm	Use in the literature			
	Interactions			
	Non-linear			
Modeling extent and resolution	Model extent		Contractor's project boundary excludes a significant portion of the area of occupied geographic subdivisions for the species in California [54].	Contractor's project boundary excludes a significant portion of the area of occupied geographic subdivisions for the species in California [54].
	Resolution of model output			
Model selection and thresholds	Model selection			
	Selection of threshold for mapping suitable habitat			

Fig A11_*Calochortus palmeri* var. *palmeri*.

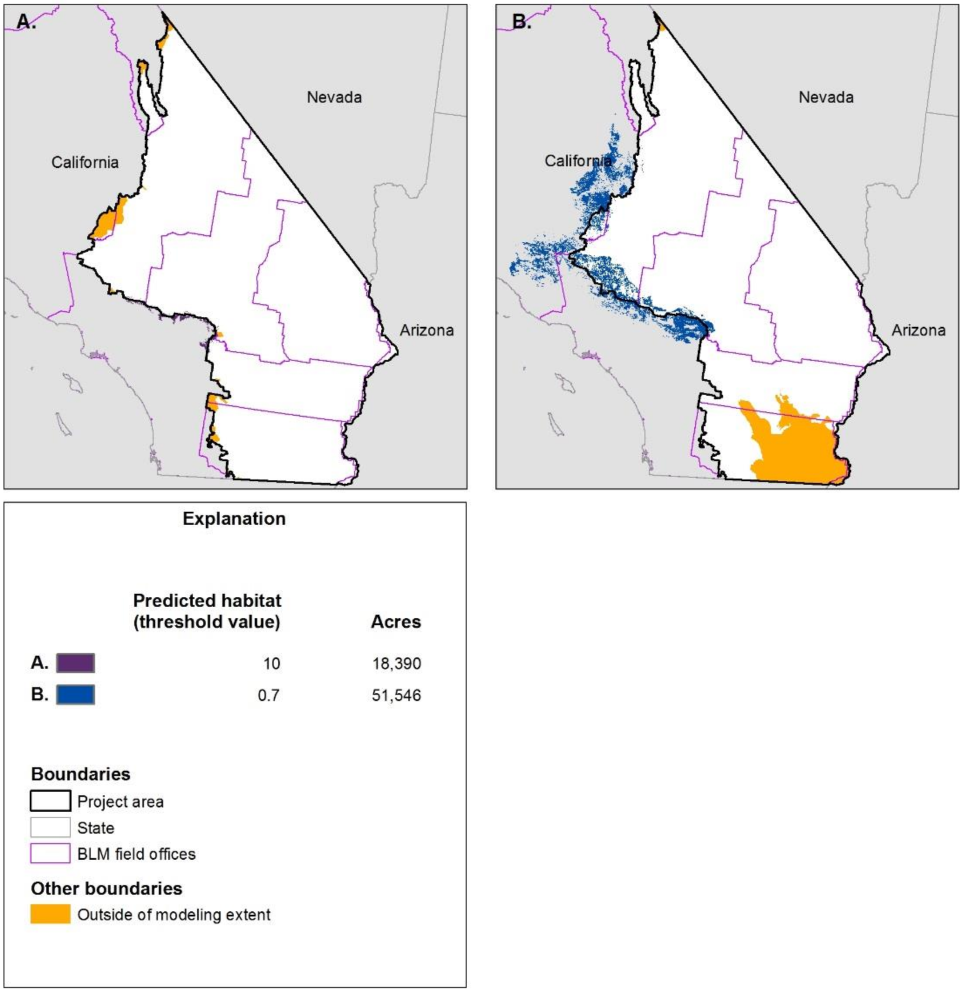

Fig B11_*Calochortus palmeri* var. *palmeri*.

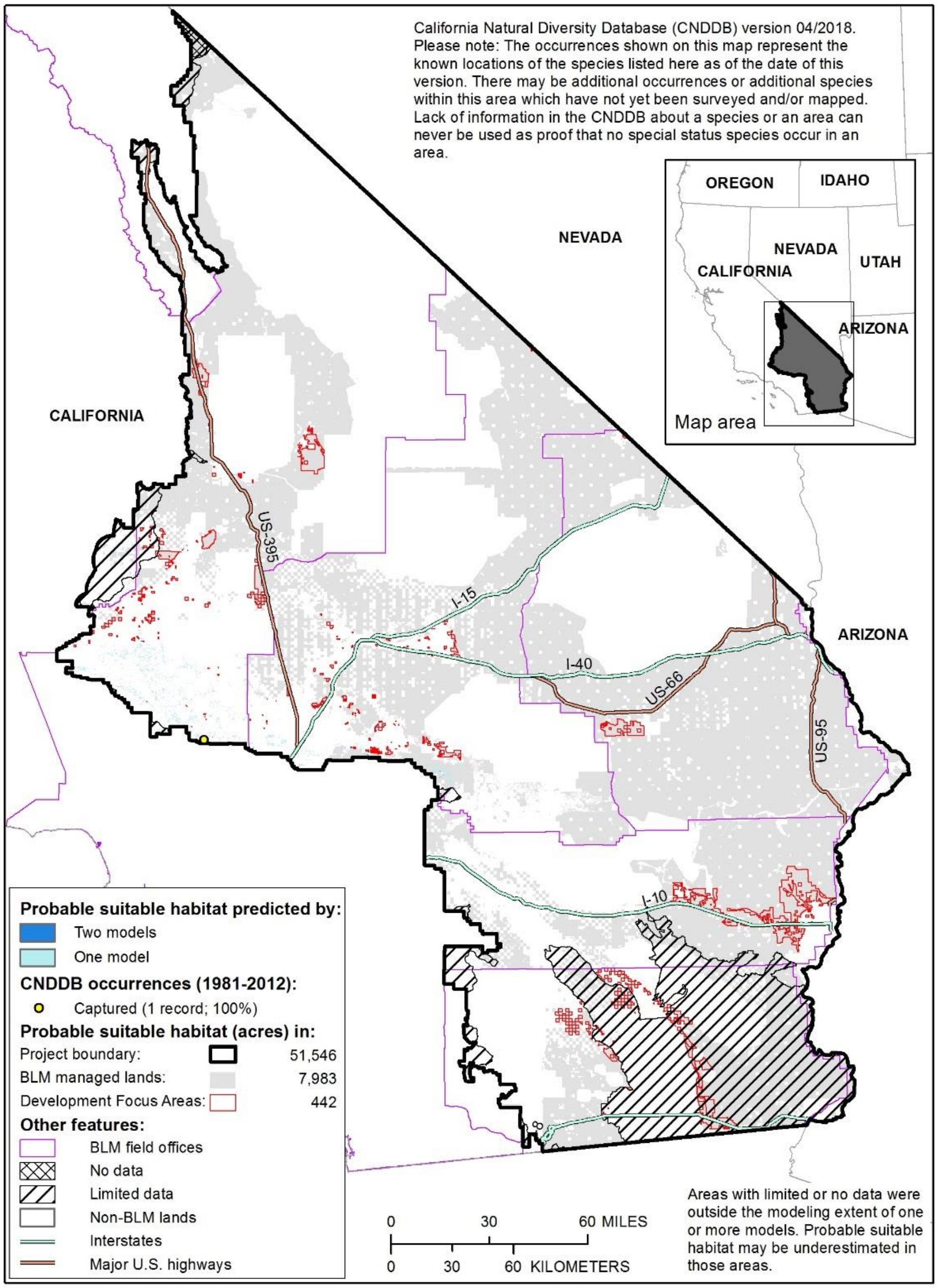

Fig C11_*Calochortus palmeri* var. *palmeri*.

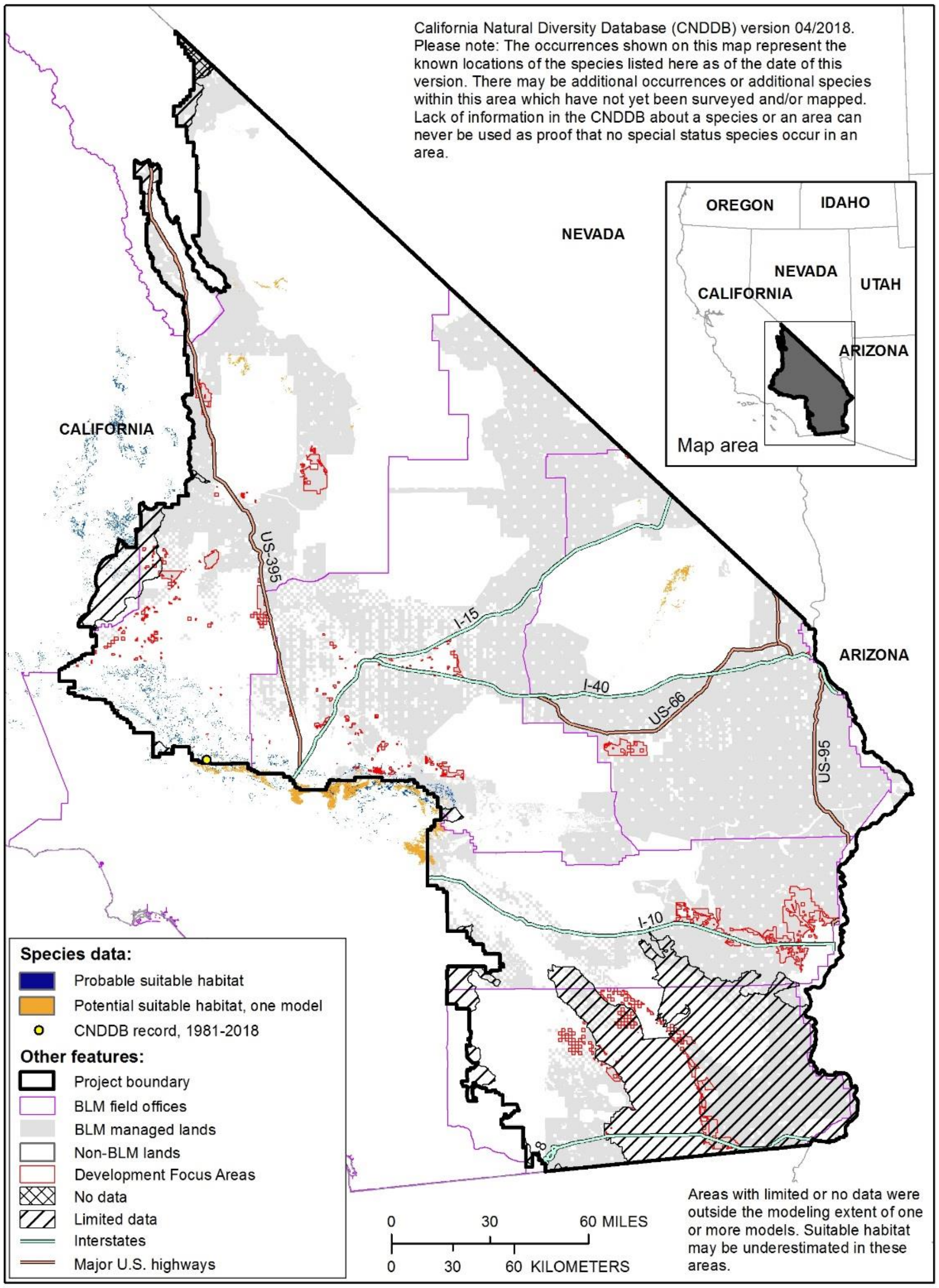

Table B12_*Calochortus striatus*.

Category	Topic	Contractor A	Contractor B	Contractor C
Occurrence data used to develop the model	Number of occurrences*	Report/data indicate that model was built from 479 occurrences. Currently available CNDDB data indicate 81 occurrences were likely used by this contractor for model development.	Report/data indicate that model was built from 89 occurrences. Currently available CNDDB data indicate 89 occurrences were available for use by this contractor for model development.	Report/data indicate that model was built from 108 occurrences. Currently available CNDDB data indicate 89 occurrences were available for use by this contractor for model development.
	Age of occurrences*	Report indicates use of occurrence data from 1981-2012. Many records are from prior to 2000.	5 of 89 (6%) currently available CNDDB occurrences are from prior to 1981.	5 of 89 (6%) currently available CNDDB occurrences are from prior to 1981.
	Spatial accuracy of occurrences*	Report/data indicate occurrences with uncertainty >250-500 m were excluded.	20 of 89 (22%) currently available CNDDB occurrences have imprecise spatial accuracy.	20 of 89 (22%) currently available CNDDB occurrences have imprecise spatial accuracy.
	Status of occurrences*	13 of 81 (16%) currently available CNDDB occurrences have Fair or Poor occurrence ranks.	13 of 89 (15%) currently available CNDDB occurrences have Fair or Poor occurrence ranks.	13 of 89 (15%) currently available CNDDB occurrences have Fair or Poor occurrence ranks.
	Species identification of occurrences*			A small portion of records (ca. 18%) appear to be from a sources other than CNDDB, for which the reliability of species

Category	Topic	Contractor A	Contractor B	Contractor C
				identification is unknown.
	Spatial bias of occurrences*			
	Spatial distribution of occurrences*	Currently available CNDDB records are from a substantial portion of the occupied geographic subdivisions for the species in California [54].	Currently available CNDDB records are from a limited portion of the occupied geographic subdivisions for the species in California [54].	Currently available CNDDB records are from a substantial portion of the occupied geographic subdivisions for the species in California [54].
	Absence data			
Environ-mental covariates	Ecological relevance			
	Comprehensive			
	Resolution and scale			
	Accuracy			
	Number of covariates	Model includes not more than 22 covariates and 479 occurrences.	Model includes 17 covariates and 89 occurrences.	Model includes 7 covariates and 108 occurrences; report stated that no more than one variable per 10 occurrences was allowed.
	Current covariate data			
	Covariate selection			
	Correlation			
Modeling algorithm	Use in the literature			

Category	Topic	Contractor A	Contractor B	Contractor C
	Interactions			
	Non-linear			
Modeling extent and resolution	Model extent	Contractor's project boundary included most of the area of occupied geographic subdivisions for the species in California [54].	Contractor's project boundary included most of the area of occupied geographic subdivisions for the species in California [54].	Contractor's project boundary included most of the area of occupied geographic subdivisions for the species in California [54].
	Resolution of model output			
Model selection and thresholds	Model selection			
	Selection of threshold for mapping suitable habitat			

Fig A12_*Calochortus striatus*.

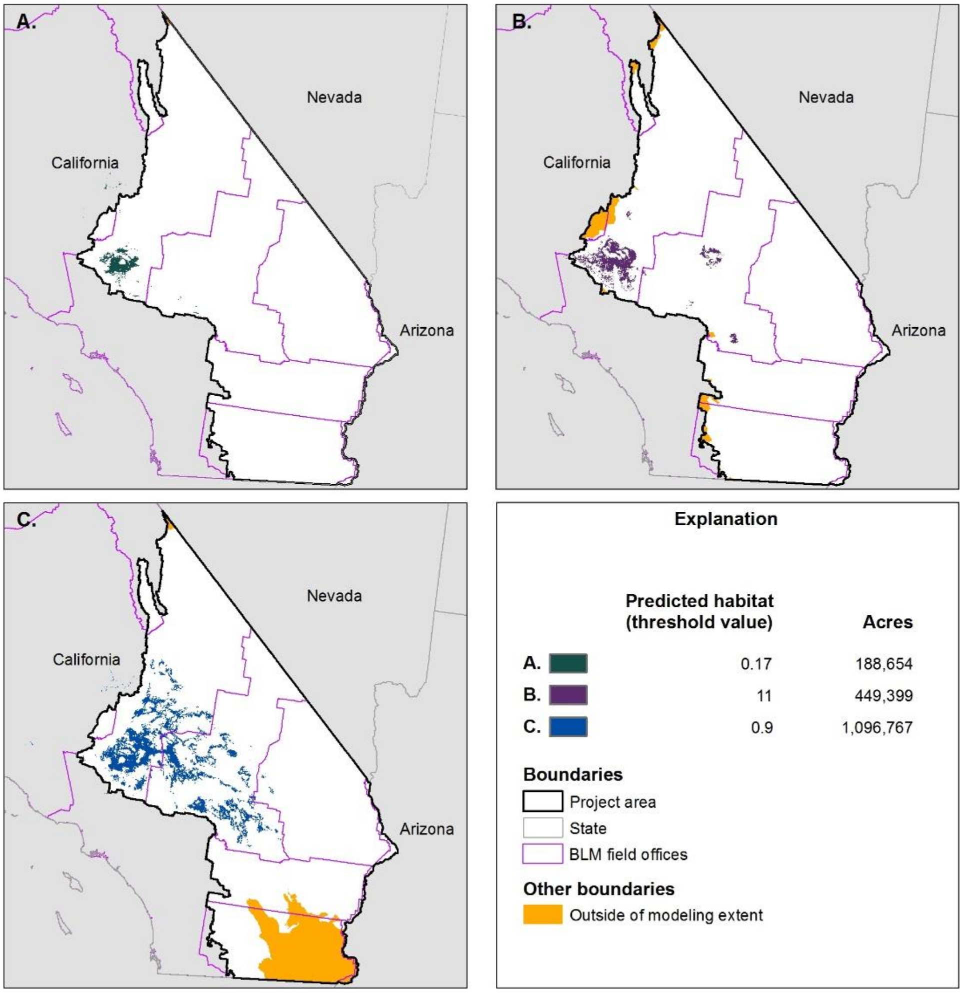

Fig B12_*Calochortus striatus*.

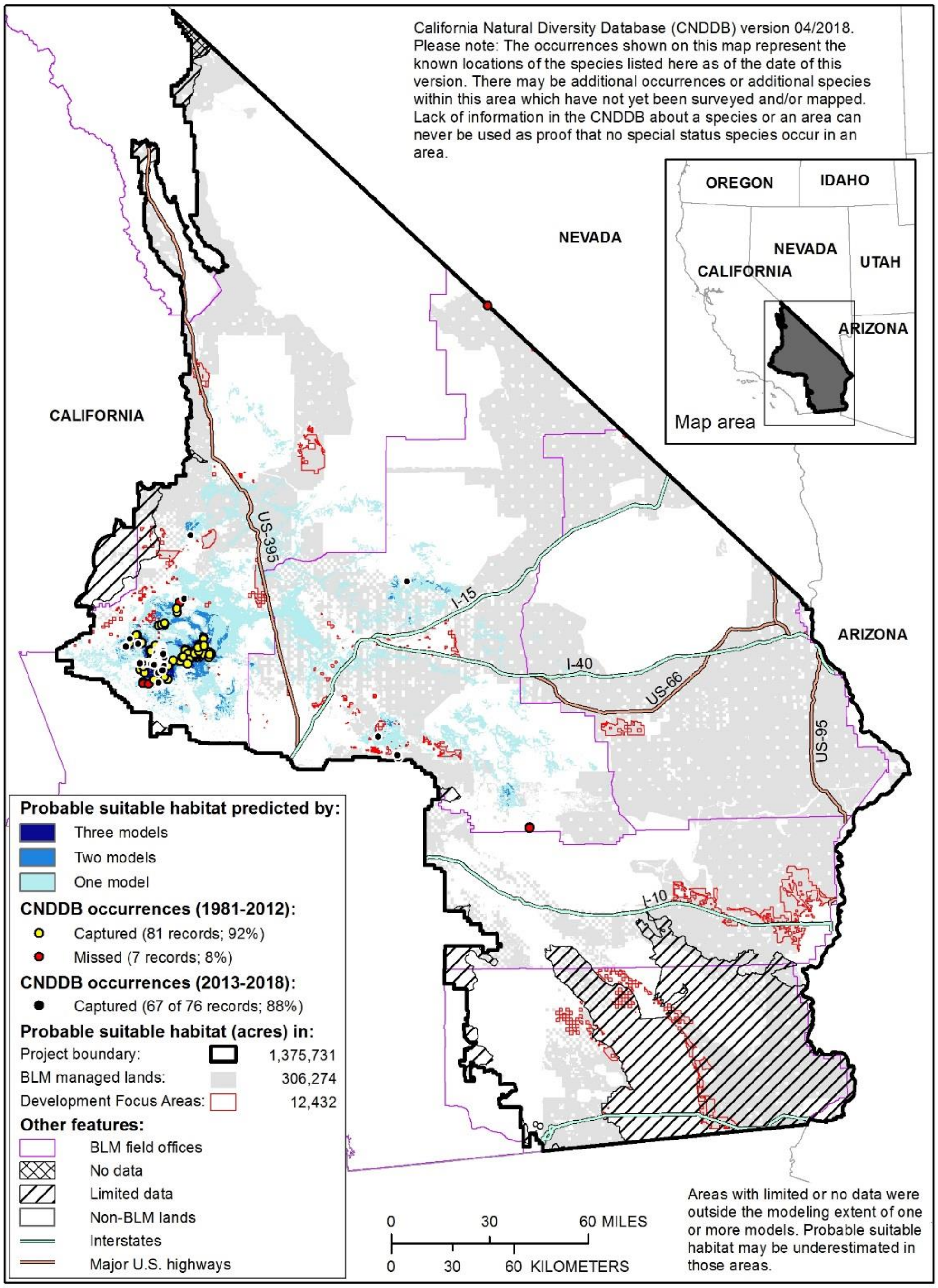

Fig C12_*Calochortus striatus*.

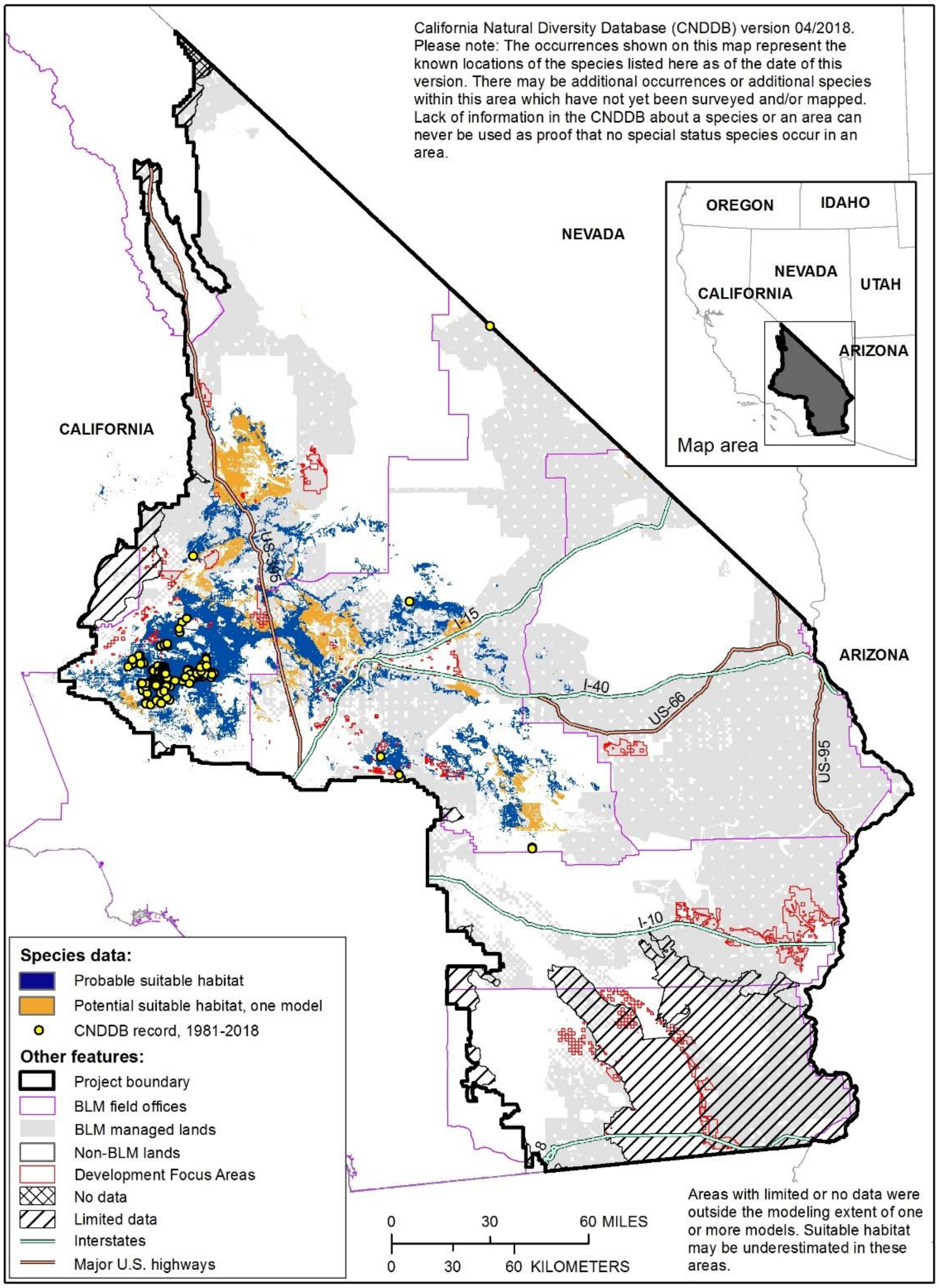

Table B13_*Chamaesyce platysperma*.

Category	Topic		Contractor B	
Occurrence data used to develop the model	Number of occurrences*		Report/data indicate that model was built from 3 occurrences. Currently available CNDDB data indicate 4 occurrences were available for use by this contractor for model development.	
	Age of occurrences*		3 of 4 (75%) currently available CNDDB occurrences is from prior to 1981.	
	Spatial accuracy of occurrences*		3 of 4 (75%) currently available CNDDB occurrences have imprecise spatial accuracy.	
	Status of occurrences*		0 of 4 (0%) currently available CNDDB occurrences have Fair or Poor occurrence ranks.	
	Species identification of occurrences*			
	Spatial bias of occurrences*			

Category	Topic		Contractor B	
	Spatial distribution of occurrences*		Currently available CNDDB records in the contractor's boundary are from a substantial portion of the area of the occupied geographic subdivision for the species in California [54].	
	Absence data			
Environ-mental covariates	Ecological relevance			
	Comprehensive			
	Resolution and scale			
	Accuracy			
	Number of covariates		Model includes 19 covariates and 3 occurrences.	
	Current covariate data			
	Covariate selection			
	Correlation			
Modeling algorithm	Use in the literature			
	Interactions			
	Non-linear			

Category	Topic		Contractor B	
Modeling extent and resolution	Model extent		Contractor's project boundary includes most of the area of the occupied geographic subdivisions for the species in California [54], but not a complete buffer around them.	
	Resolution of model output			
Model selection and thresholds	Model selection			
	Selection of threshold for mapping suitable habitat			

Fig A13_*Chamaesyce platysperma*.

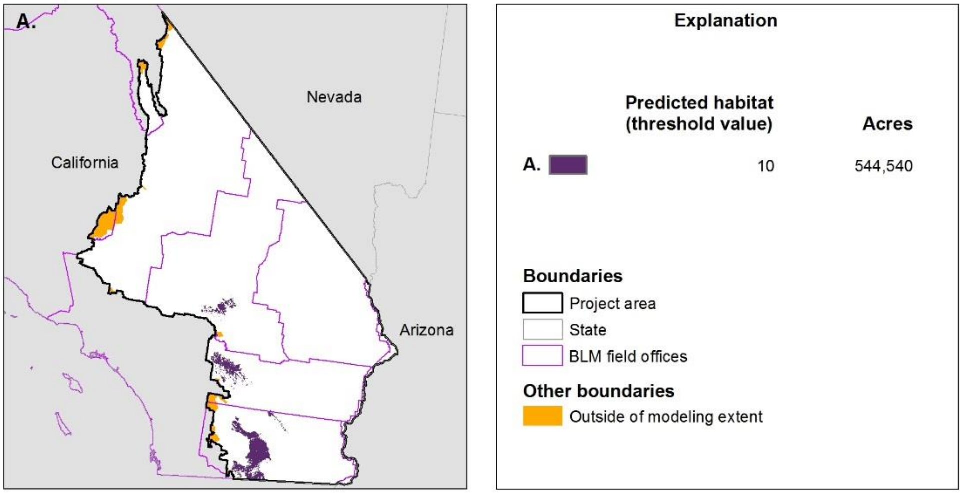

Fig C13_*Chamaesyce platysperma*.

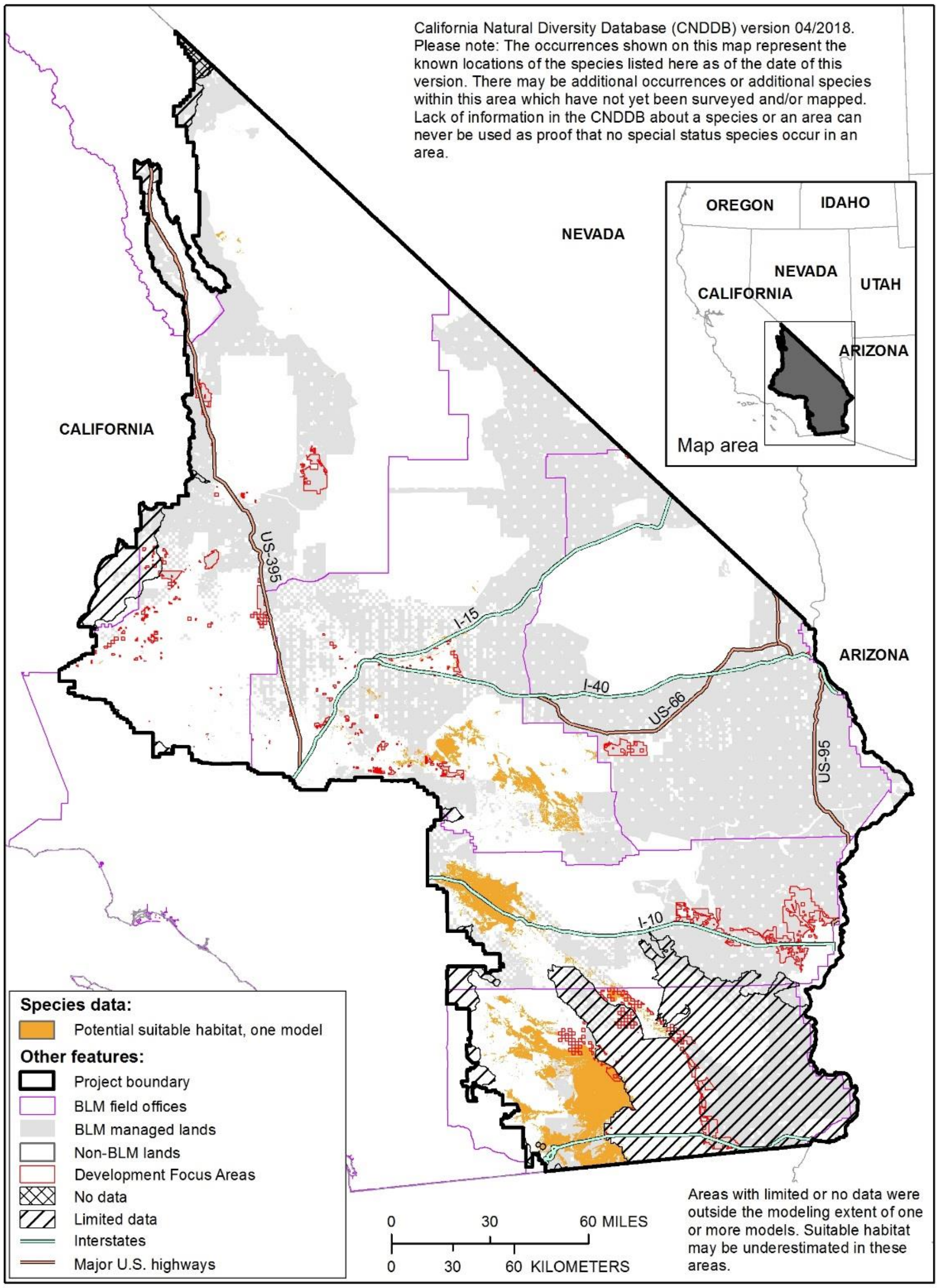

Table B14_*Cylindropuntia munzii*.

Category	Topic		Contractor B	
Occurrence data used to develop the model	Number of occurrences*		Report/data indicate that model was built from 2 occurrences. Currently available CNDDB data indicate 2 occurrences were available for use by this contractor for model development.	
	Age of occurrences*		0 of 2 (0%) currently available CNDDB occurrences is from prior to 1981.	
	Spatial accuracy of occurrences*		2 of 2 (100%) currently available CNDDB occurrences have imprecise spatial accuracy.	
	Status of occurrences*		0 of 2 (0%) currently available CNDDB occurrences have Fair or Poor occurrence ranks.	
	Species identification of occurrences*			
	Spatial bias of occurrences*			

Category	Topic		Contractor B	
	Spatial distribution of occurrences*		Currently available CNDDB records in the contractor’s boundary are from a limited portion of the area of the occupied geographic subdivision for the species in California [54].	
	Absence data			
Environ-mental covariates	Ecological relevance			
	Comprehensive			
	Resolution and scale			
	Accuracy			
	Number of covariates		Model includes 13 covariates and 2 occurrences.	
	Current covariate data			
	Covariate selection			
	Correlation			
Modeling algorithm	Use in the literature			
	Interactions			
	Non-linear			

Category	Topic		Contractor B	
Modeling extent and resolution	Model extent		Contractor's project boundary includes most of the area of the occupied geographic subdivision for the species in California [54], but not a complete buffer around it.	
	Resolution of model output			
Model selection and thresholds	Model selection			
	Selection of threshold for mapping suitable habitat			

Fig A14_*Cylindropuntia munzii*.

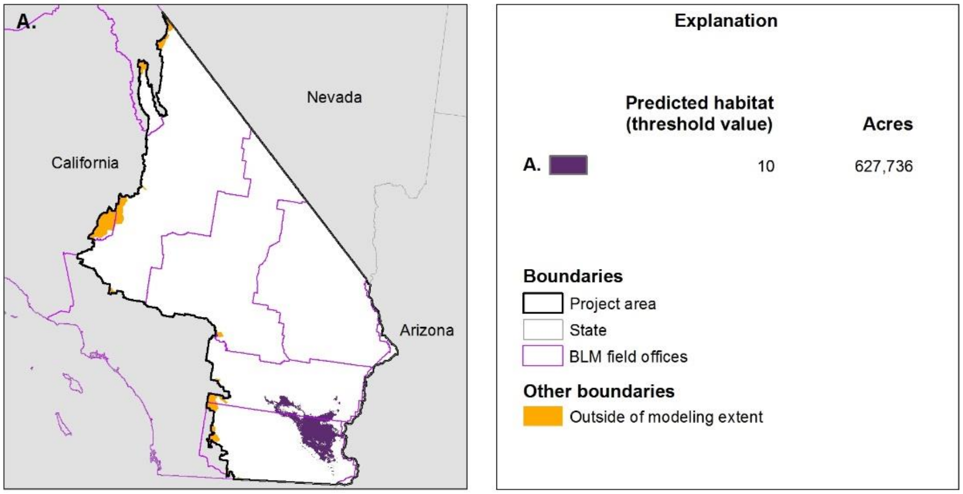

Fig C14_*Cylindropuntia munzii*.

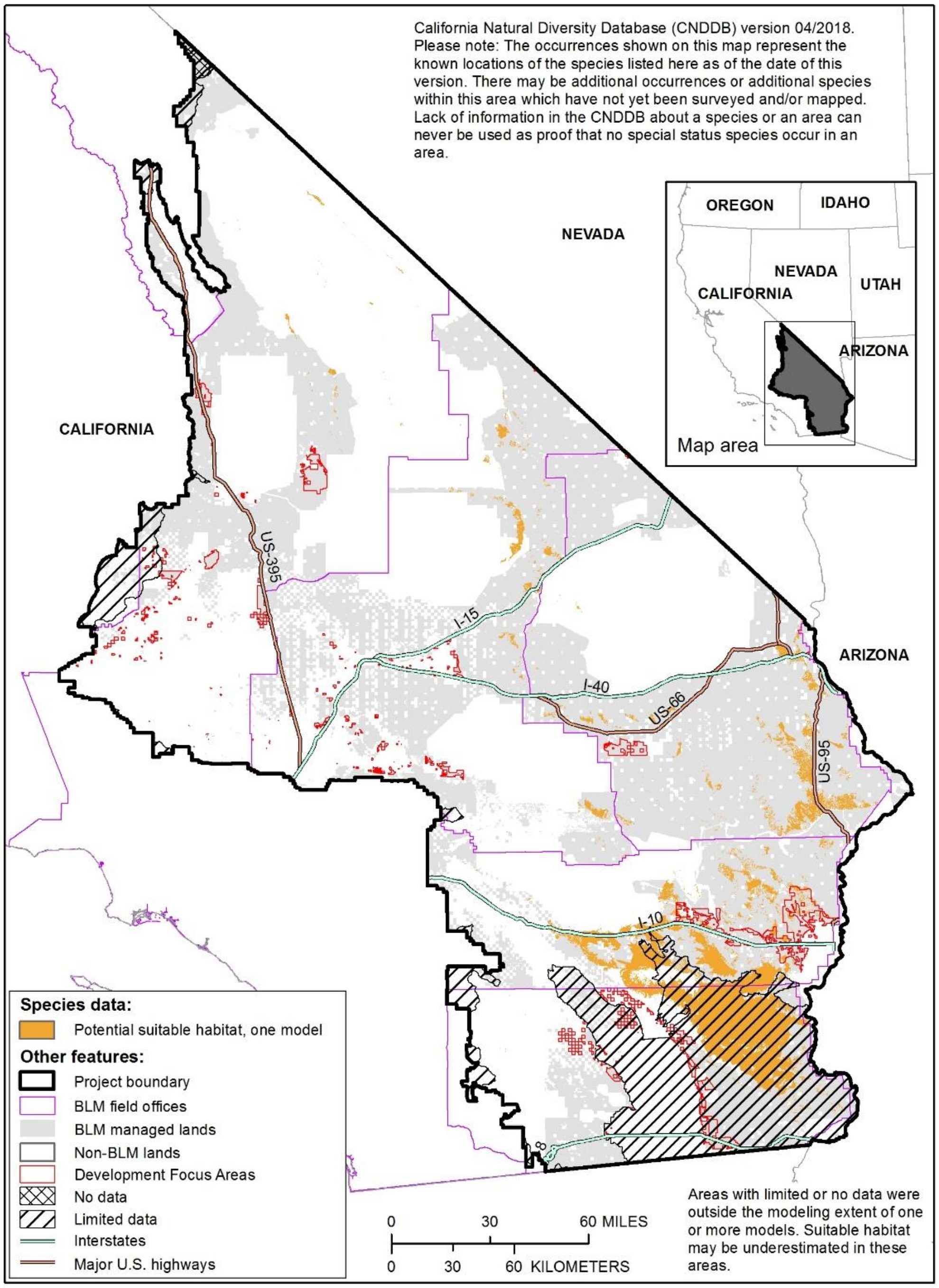

Table B15_*Cymopterus deserticola*.

Category	Topic	Contractor A	Contractor B	Contractor C
Occurrence data used to develop the model	Number of occurrences*	Report/data indicate that model was built from 319 occurrences. Currently available CNDDB data indicate 76 occurrences were likely used by this contractor for model development.	Report/data indicate that model was built from 79 occurrences. Currently available CNDDB data indicate 78 occurrences were available for use by this contractor for model development.	Report/data indicate that model was built from 79 occurrences. Currently available CNDDB data indicate 78 occurrences were available for use by this contractor for model development.
	Age of occurrences*	Report indicates use of occurrence data from 1981-2012. Many records are from prior to 2000.	2 of 78 (2%) currently available CNDDB occurrences are from prior to 1981.	2 of 78 (2%) currently available CNDDB occurrences are from prior to 1981.
	Spatial accuracy of occurrences*	Report/data indicate occurrences with uncertainty >250-500 m were excluded.	9 of 78 (12%) currently available CNDDB occurrences have imprecise spatial accuracy.	9 of 78 (12%) currently available CNDDB occurrences have imprecise spatial accuracy.
	Status of occurrences*	49 of 76 (64%) currently available CNDDB occurrences have Fair or Poor occurrence ranks.	50 of 78 (64%) currently available CNDDB occurrences have Fair or Poor occurrence ranks.	50 of 78 (64%) currently available CNDDB occurrences have Fair or Poor occurrence ranks.
	Species identification of occurrences*			Nearly all records appear to be from CNDDB, for which species identification is reliable.
	Spatial bias of occurrences*			

Category	Topic	Contractor A	Contractor B	Contractor C
	Spatial distribution of occurrences*	Currently available CNDDB records are from a substantial portion of the occupied geographic subdivisions for the species in California [54].	Currently available CNDDB records are from a substantial portion of the occupied geographic subdivisions for the species in California [54].	Currently available CNDDB records are from a substantial portion of the occupied geographic subdivisions for the species in California [54].
	Absence data			
Environ-mental covariates	Ecological relevance			
	Comprehensive			
	Resolution and scale			
	Accuracy			
	Number of covariates	We could not determine the number of covariates used based on the available data, but with 319 occurrences the ratio is likely at least acceptable.	Model includes 17 covariates and 79 occurrences.	Model includes 7 covariates and 79 occurrences; report stated that no more than one variable per 10 occurrences was allowed.
	Current covariate data			
	Covariate selection			
	Correlation			
Modeling algorithm	Use in the literature			
	Interactions			
	Non-linear			

Category	Topic	Contractor A	Contractor B	Contractor C
Modeling extent and resolution	Model extent	Contractor's project boundary included nearly all of the area of occupied geographic subdivisions for the species in California [54], but not a buffer that extends all the way around it.	Contractor's project boundary included nearly all of the area of occupied geographic subdivisions for the species in California [54], but not a buffer that extends all the way around it.	Contractor's project boundary included nearly all of the area of occupied geographic subdivisions for the species in California [54], but not a buffer that extends all the way around it.
	Resolution of model output			
Model selection and thresholds	Model selection			
	Selection of threshold for mapping suitable habitat			

Fig A15_*Cymopterus deserticola*.

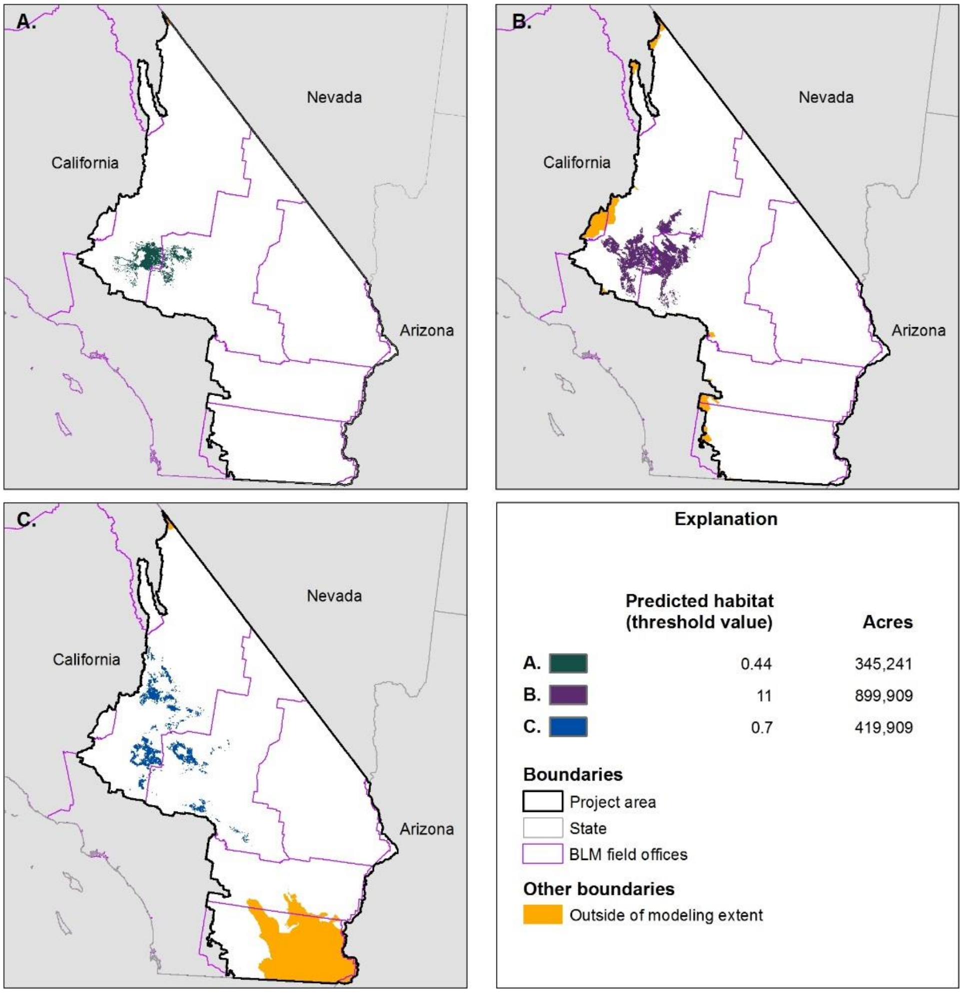

Fig B15_*Cymopterus deserticola*.

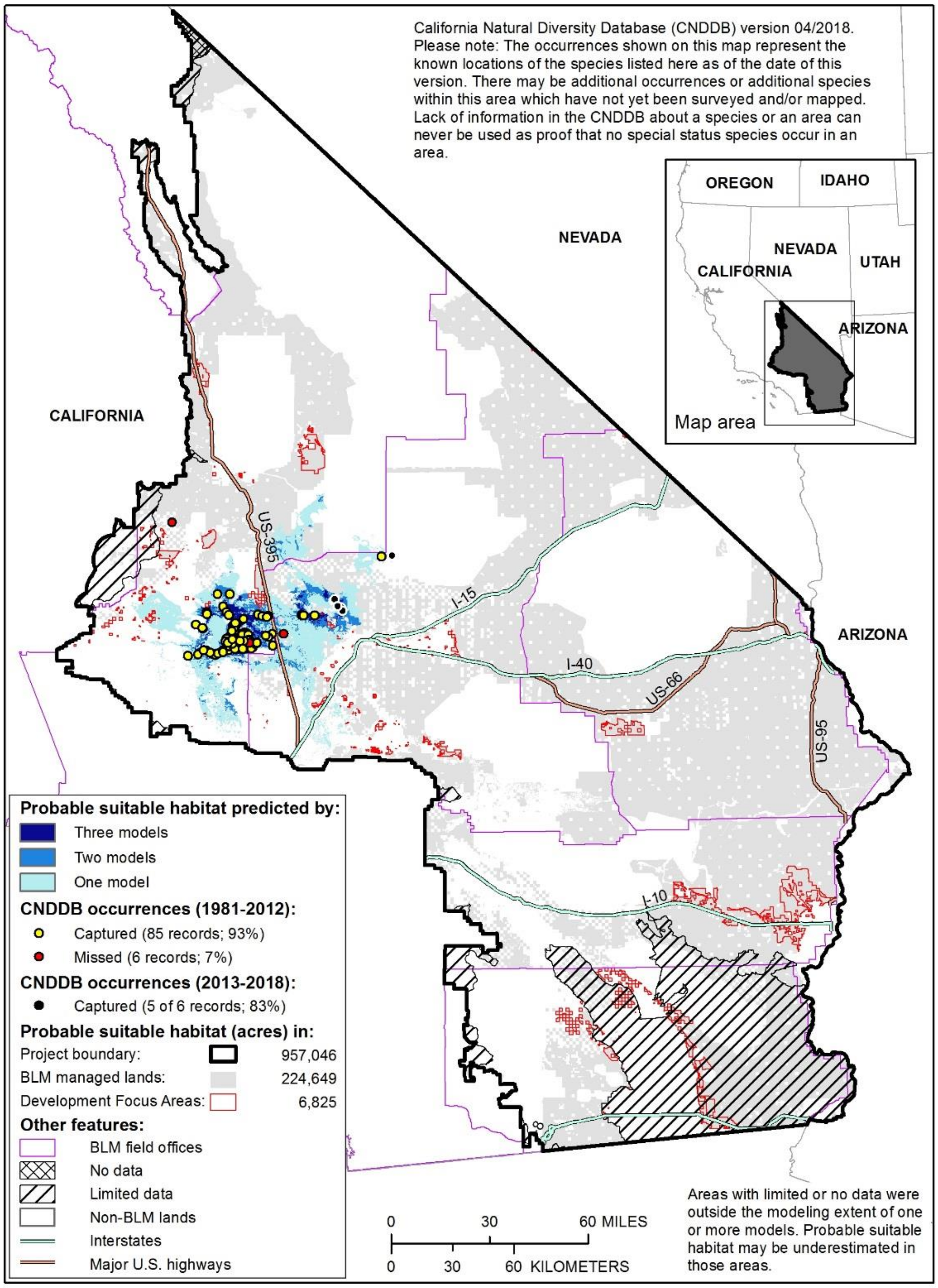

Fig C15_*Cymopterus deserticola*.

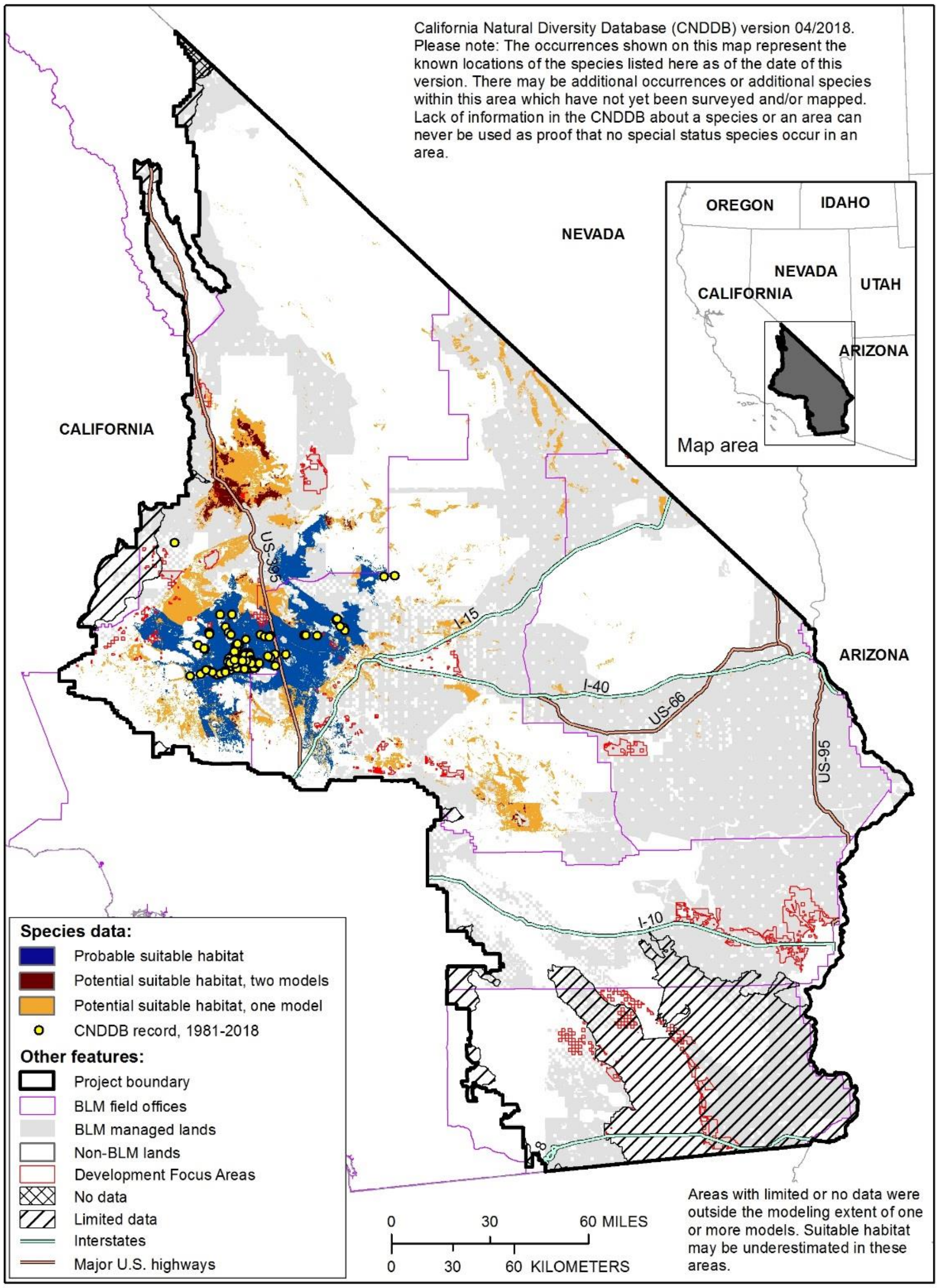

Table B16_*Deinandra mohavensis*.

Category	Topic	Contractor A	Contractor B	Contractor C
Occurrence data used to develop the model	Number of occurrences*	Report/data indicate that two separate (regional) models were built from 23 and 131 occurrences. Currently available CNDDB data indicate 71 occurrences were likely used by this contractor for model development.	Report/data indicate that model was built from 7 occurrences. Currently available CNDDB data indicate 74 occurrences were available for use by this contractor for model development, but many were outside their project boundary.	Report/data indicate that model was built from 114 occurrences. Currently available CNDDB data indicate 74 occurrences were available for use by this contractor for model development.
	Age of occurrences*	Report indicates use of occurrence data from 1981-2012. Numerous records are from prior to 2000.	3 of 74 (4%) currently available CNDDB occurrences are from prior to 1981.	3 of 74 (4%) currently available CNDDB occurrences are from prior to 1981.
	Spatial accuracy of occurrences*	Report/data indicate occurrences with uncertainty >250-500 m were excluded.	19 of 74 (26%) currently available CNDDB occurrences have imprecise spatial accuracy.	19 of 74 (26%) currently available CNDDB occurrences have imprecise spatial accuracy.
	Status of occurrences*	9 of 71 (13%) currently available CNDDB occurrences have Fair or Poor occurrence ranks.	9 of 74 (12%) currently available CNDDB occurrences have Fair or Poor occurrence ranks.	9 of 74 (12%) currently available CNDDB occurrences have Fair or Poor occurrence ranks.
	Species identification of occurrences*			A substantial portion of records appear to be from sources other than CNDDB, for which the

Category	Topic	Contractor A	Contractor B	Contractor C
				reliability of species identification is unknown.
	Spatial bias of occurrences*			
	Spatial distribution of occurrences*	Currently available CNDDB records in the contractor's boundary are from a substantial portion of the occupied geographic subdivisions for the species in California [54].	Currently available CNDDB records in the contractor's boundary are from a limited portion of the occupied geographic subdivisions for the species in California [54].	Currently available CNDDB records in the contractor's boundary are from a substantial portion of the occupied geographic subdivisions for the species in California [54].
	Absence data			
Environ-mental covariates	Ecological relevance			
	Comprehensive			
	Resolution and scale			
	Accuracy			
	Number of covariates	Models include 9 covariates and 23 or 131 occurrences, which is at a minimum acceptable.	Model includes 16 covariates and 7 occurrences.	Model includes 9 covariates and 114 occurrences; report stated that no more than one variable per 10 occurrences was allowed.
	Current covariate data			
	Covariate selection			
	Correlation			

Category	Topic	Contractor A	Contractor B	Contractor C
Modeling algorithm	Use in the literature			
	Interactions			
	Non-linear			
Modeling extent and resolution	Model extent	Contractor's project boundary included much of the area of occupied geographic subdivisions for the species in California [54].	Contractor's project boundary excludes a significant portion of the area of occupied geographic subdivisions for the species in California [54].	Contractor's project boundary included much of the area of occupied geographic subdivisions for the species in California [54].
	Resolution of model output			
Model selection and thresholds	Model selection			
	Selection of threshold for mapping suitable habitat			

Fig A16_*Deinandra mohavensis*.

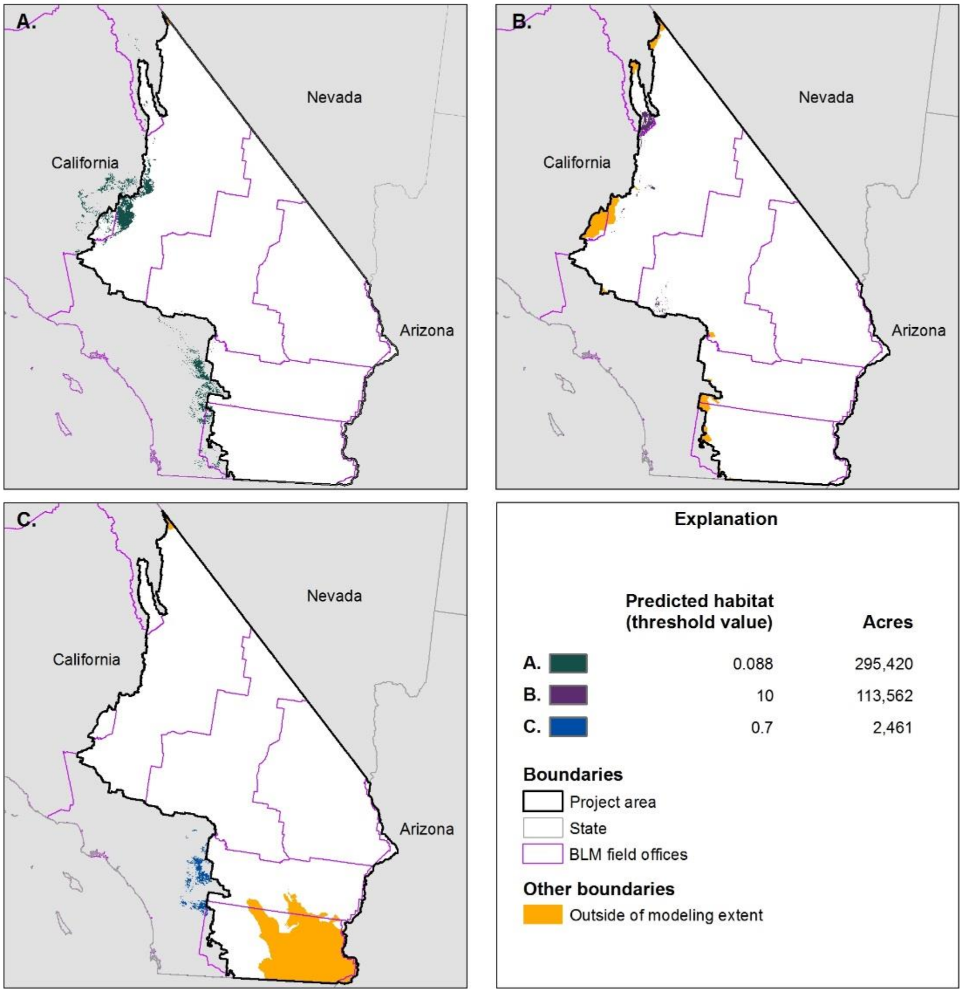

Fig B16_*Deinandra mohavensis*.

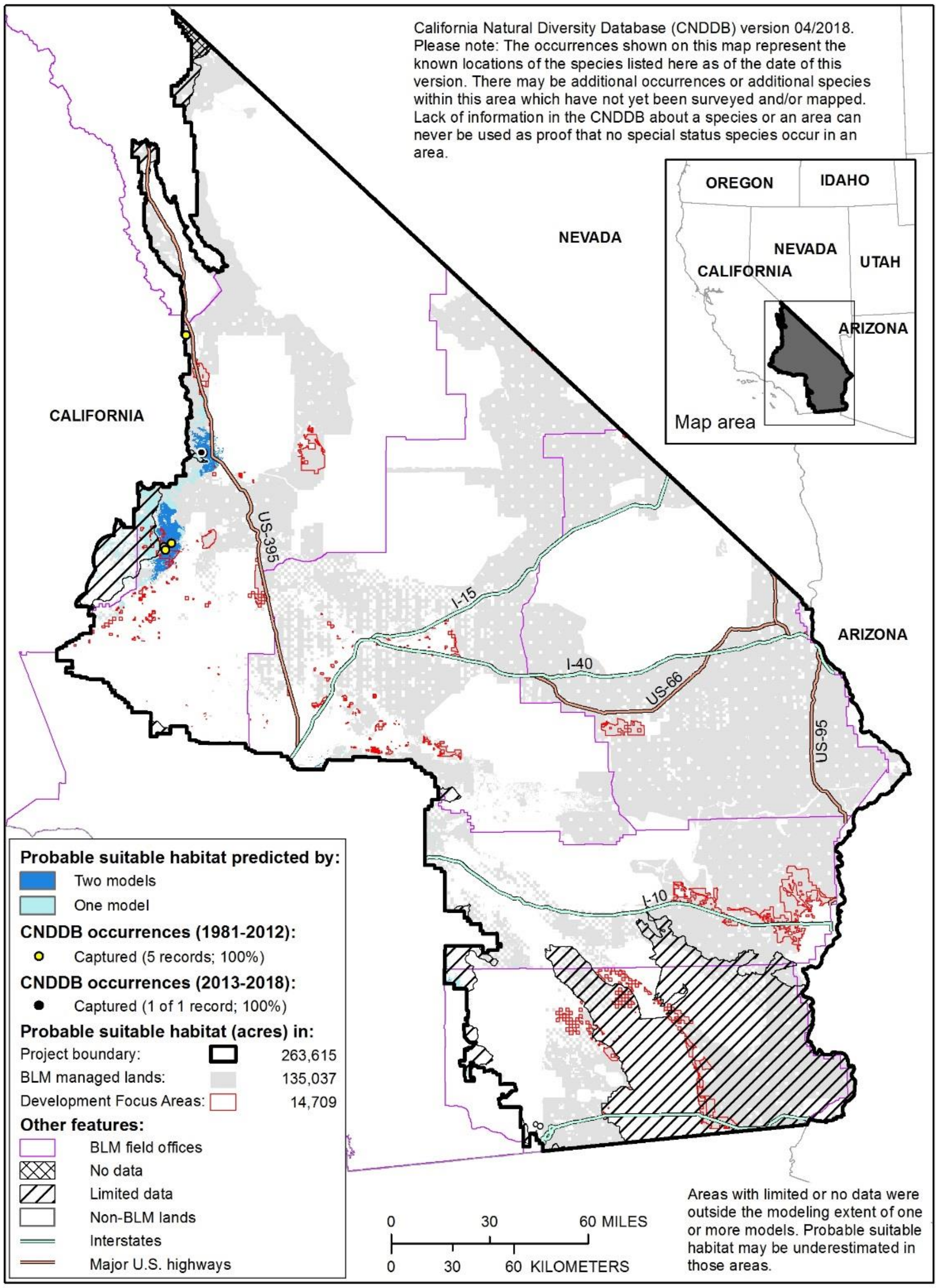

Fig C16_*Deinandra mohavensis*.

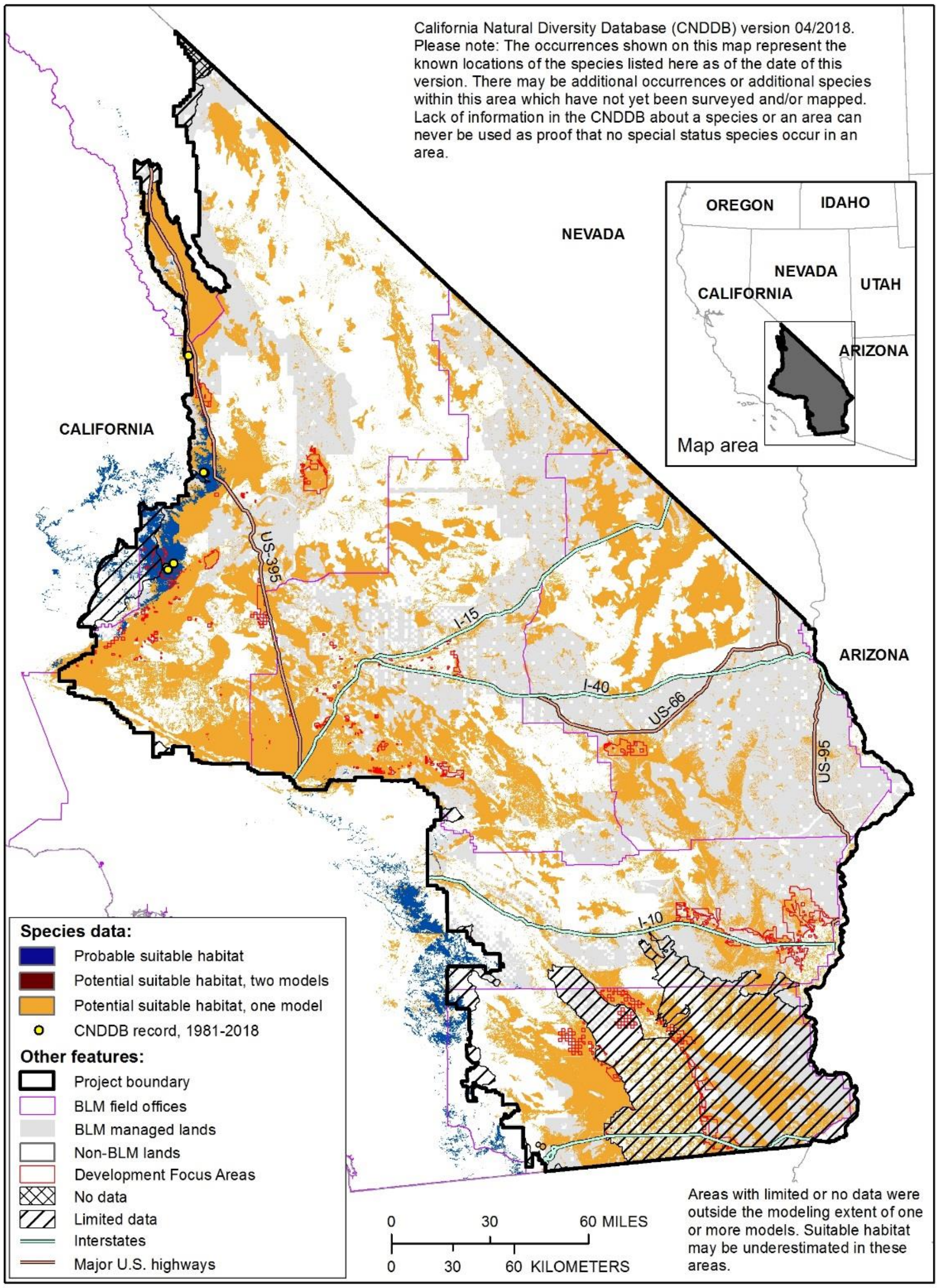

Table B17_*Echinocereus engelmannii* var. *howei.*

Category	Topic		Contractor B	
Occurrence data used to develop the model	Number of occurrences*		Report/data indicate that model was built from 3 occurrences. Currently available CNDDB data indicate 3 occurrences were available for use by this contractor for model development.	
	Age of occurrences*		1 of 3 (33%) currently available CNDDB occurrences is from prior to 1981.	
	Spatial accuracy of occurrences*		2 of 3 (67%) currently available CNDDB occurrences have imprecise spatial accuracy.	
	Status of occurrences*		0 of 3 (0%) currently available CNDDB occurrences have Fair or Poor occurrence ranks.	
	Species identification of occurrences*			
	Spatial bias of occurrences*			

Category	Topic		Contractor B	
	Spatial distribution of occurrences*		Currently available CNDDB records in the contractor's boundary are from a very limited portion of the area of the area shown for the species in the USDA PLANTS Database [70].	
	Absence data			
Environ-mental covariates	Ecological relevance			
	Comprehensive			
	Resolution and scale			
	Accuracy			
	Number of covariates		Model includes 13 covariates and 3 occurrences.	
	Current covariate data			
	Covariate selection			
	Correlation			
Modeling algorithm	Use in the literature			
	Interactions			
	Non-linear			

Category	Topic		Contractor B	
Modeling extent and resolution	Model extent		This subspecies is not listed in California [54]. The contractor's project boundary includes much of the area shown for the species in the USDA PLANTS Database [70], but not a complete buffer around it.	
	Resolution of model output			
Model selection and thresholds	Model selection			
	Selection of threshold for mapping suitable habitat			

Fig A17_*Echinocereus engelmannii* var. *howei*.

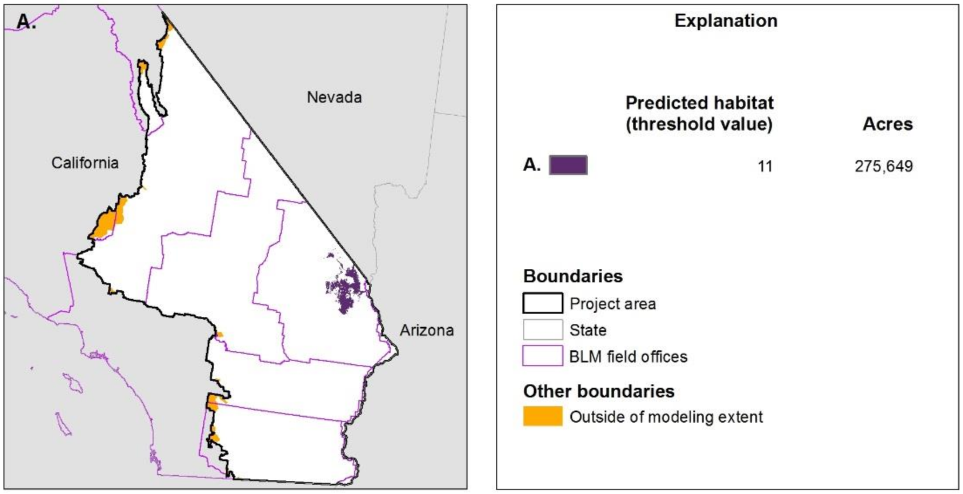

Fig C17_*Echinocereus engelmannii* var. *howei*.

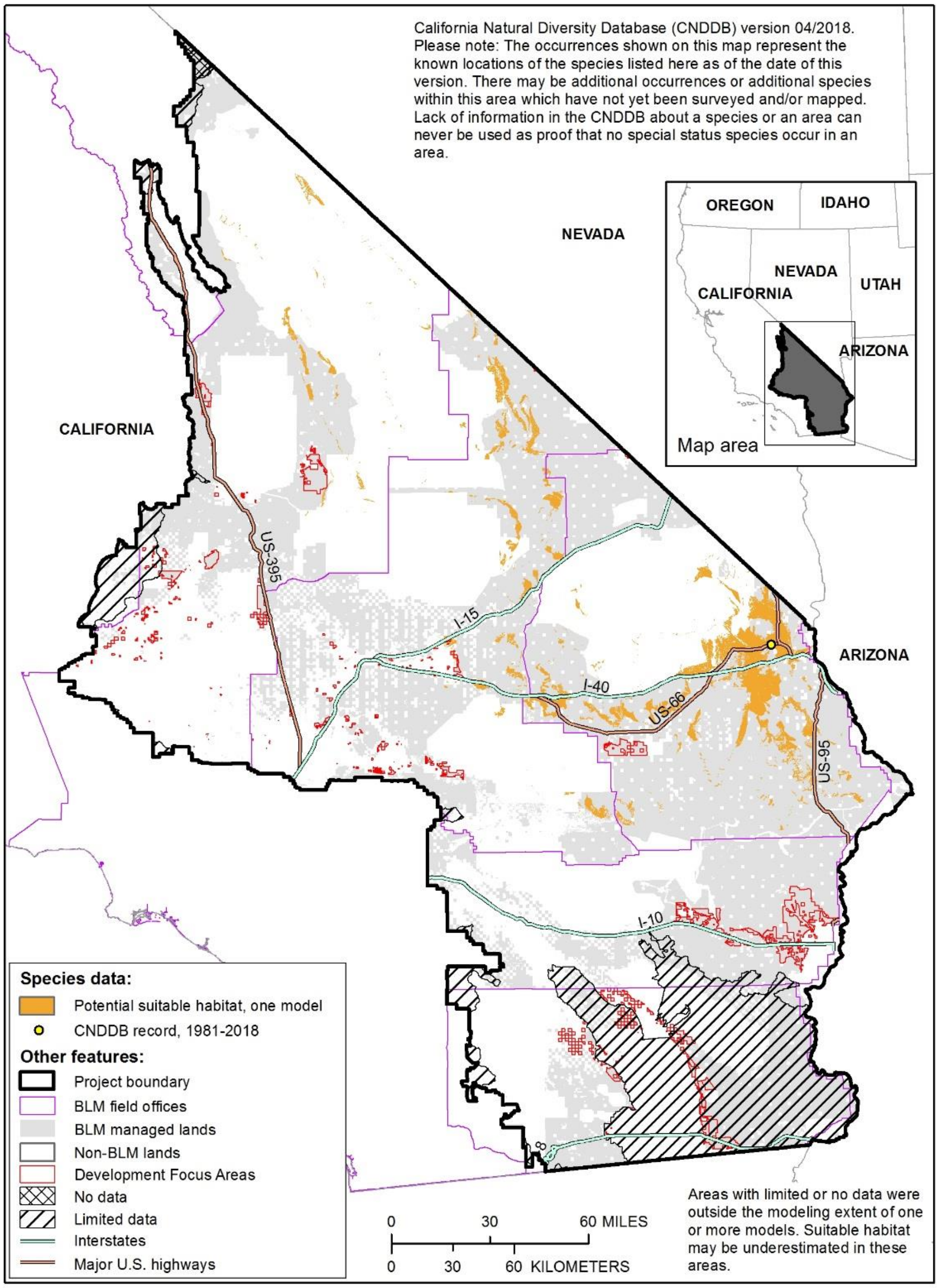

Table B18_*Erigeron parishii*.

Category	Topic	Contractor A	Contractor B	Contractor C
Occurrence data used to develop the model	Number of occurrences*	Report/data indicate that model were built from 151 occurrences. Currently available CNDDB data indicate 32 occurrences were likely used by this contractor for model development.	Report/data indicate that model was built from 35 occurrences. Currently available CNDDB data indicate 32 occurrences were available for use by this contractor for model development, but many were outside their project boundary.	Report/data indicate that model was built from 85 occurrences. Currently available CNDDB data indicate 32 occurrences were available for use by this contractor for model development.
	Age of occurrences*	Report indicates use of occurrence data from 1981-2012. Numerous records are from prior to 2000.	0 of 32 (0%) currently available CNDDB occurrences are from prior to 1981.	0 of 32 (0%) currently available CNDDB occurrences are from prior to 1981.
	Spatial accuracy of occurrences*	Report/data indicate occurrences with uncertainty >250-500 m were excluded.	2 of 32 (6%) currently available CNDDB occurrences have imprecise spatial accuracy.	2 of 32 (6%) currently available CNDDB occurrences have imprecise spatial accuracy.
	Status of occurrences*	4 of 32 (12%) currently available CNDDB occurrences have Fair or Poor occurrence ranks.	4 of 32 (12%) currently available CNDDB occurrences have Fair or Poor occurrence ranks.	4 of 32 (12%) currently available CNDDB occurrences have Fair or Poor occurrence ranks.
	Species identification of occurrences*			A substantial portion of records appear to be from sources other than CNDDB, for which the

Category	Topic	Contractor A	Contractor B	Contractor C
				reliability of species identification is unknown.
	Spatial bias of occurrences*			
	Spatial distribution of occurrences*	Currently available CNDDB records in the contractor's boundary are from a substantial portion of the occupied geographic subdivisions for the species in California [54].	Currently available CNDDB records in the contractor's boundary are from a limited portion of the occupied geographic subdivisions for the species in California [54].	Currently available CNDDB records in the contractor's boundary are from a substantial portion of the occupied geographic subdivisions for the species in California [54].
	Absence data			
Environ-mental covariates	Ecological relevance			
	Comprehensive			
	Resolution and scale			
	Accuracy			
	Number of covariates	Model includes 6 covariates and 151 occurrences.	Model includes 15 covariates and 35 occurrences.	Model includes 8 covariates and 85 occurrences; report stated that no more than one variable per 10 occurrences was allowed.
	Current covariate data			
	Covariate selection			
	Correlation			

Category	Topic	Contractor A	Contractor B	Contractor C
Modeling algorithm	Use in the literature			
	Interactions			
	Non-linear			
Modeling extent and resolution	Model extent	Contractor's project boundary appears to include most or all of the area of occupied geographic subdivisions for the species in California [54] and a buffer around it.	Contractor's project boundary excludes a significant portion of the area of occupied geographic subdivisions for the species in California [54].	Contractor's project boundary appears to include most of the area of occupied geographic subdivisions for the species in California [54].
	Resolution of model output			
Model selection and thresholds	Model selection			
	Selection of threshold for mapping suitable habitat			

Fig A18_*Erigeron parishii*.

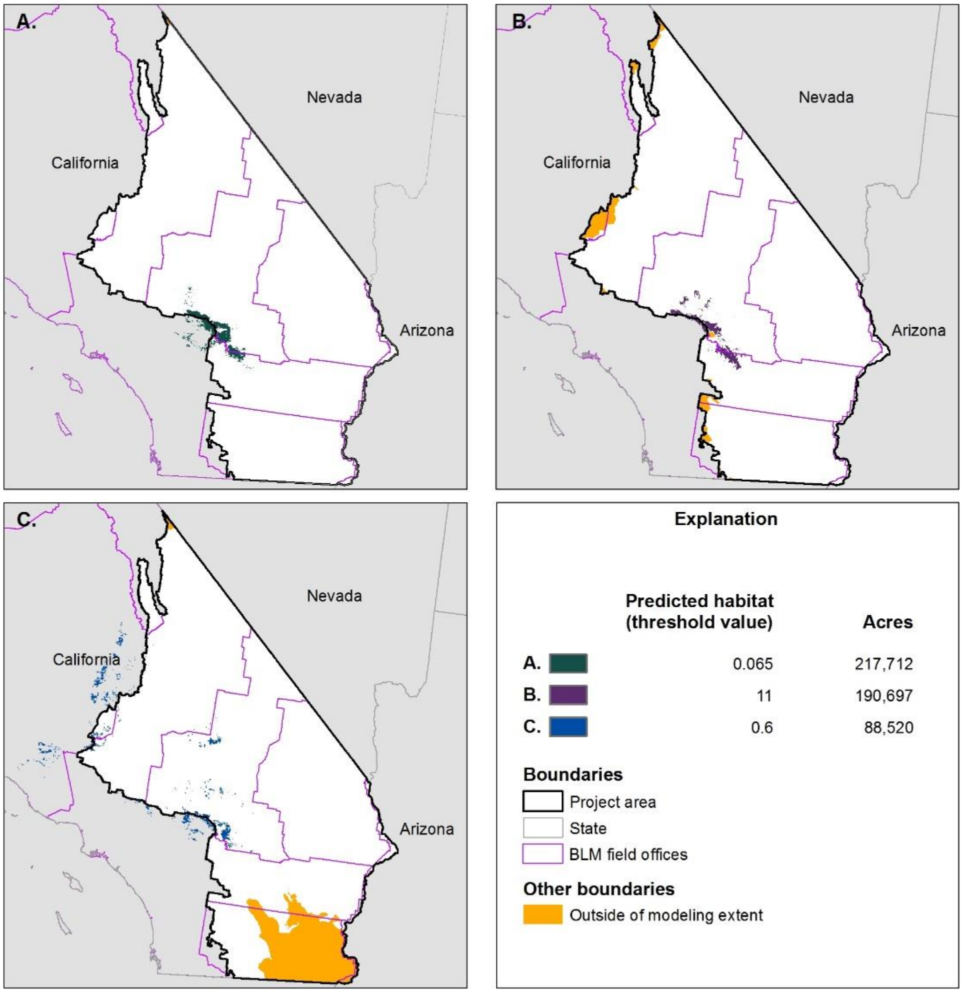

Fig B18_*Erigeron parishii*.

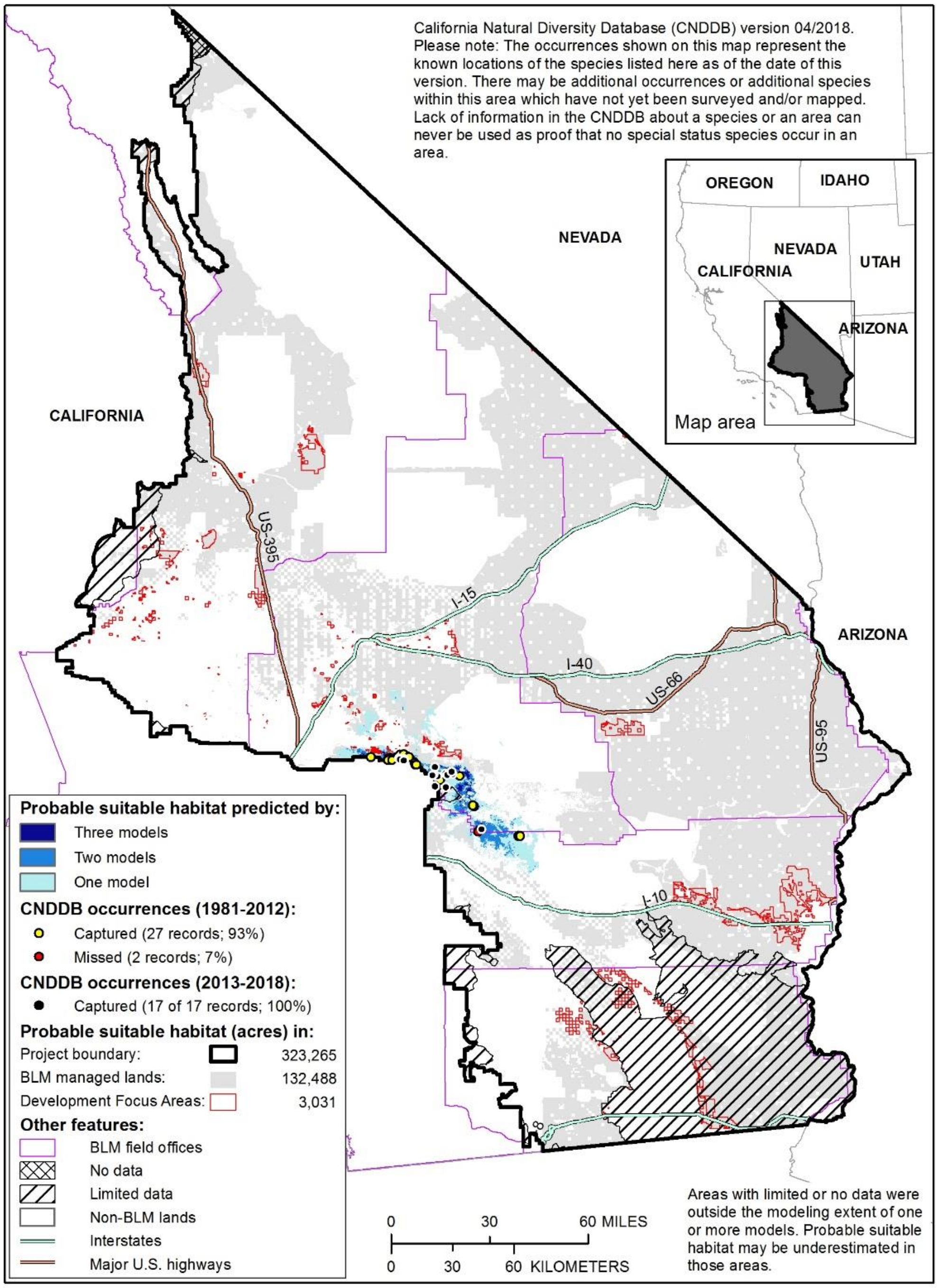

Fig C18_*Erigeron parishii*.

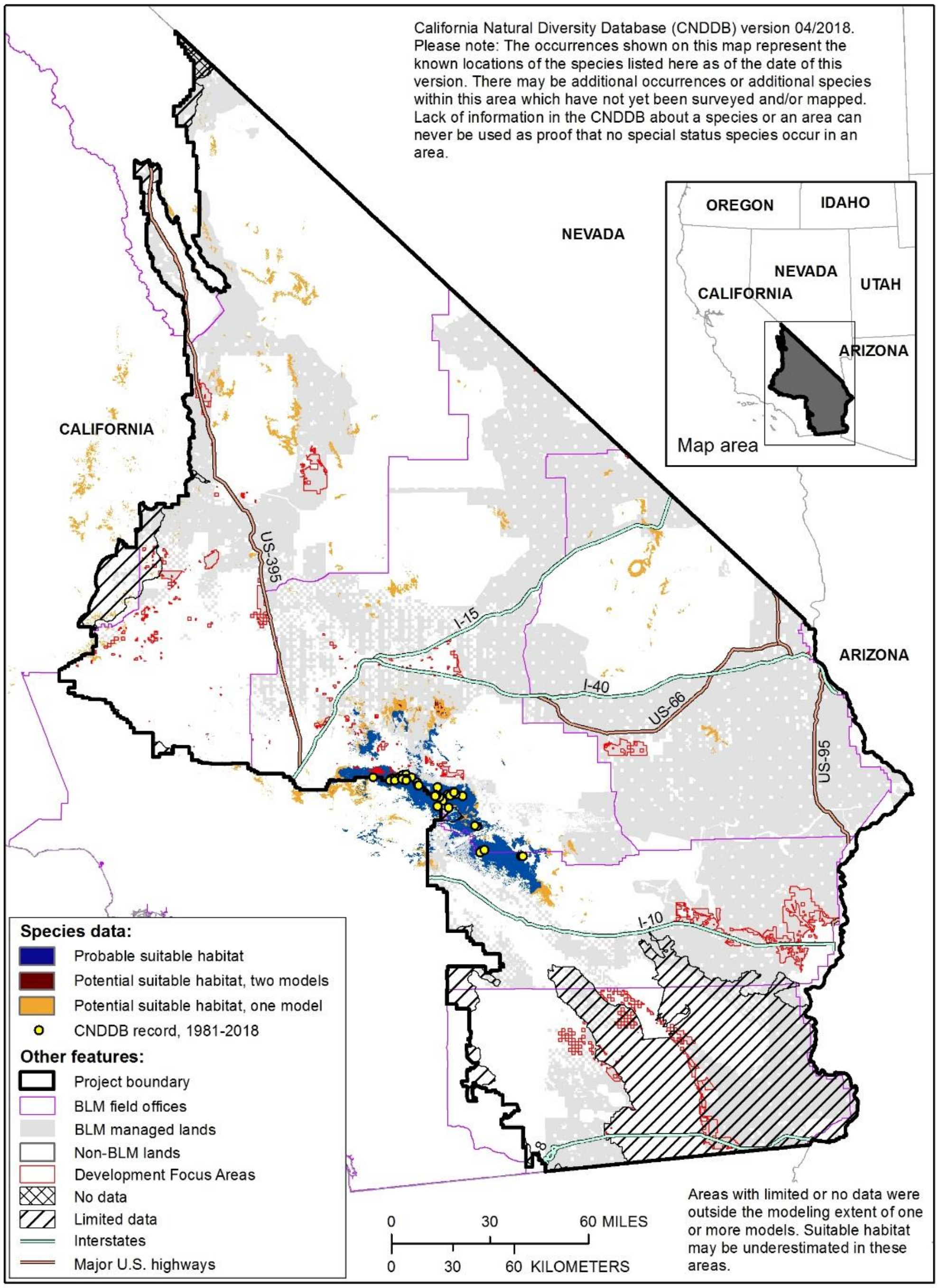

Table B19_*Eriogonum bifurcatum.*

Category	Topic		Contractor B	Contractor C
Occurrence data used to develop the model	Number of occurrences*		Report/data indicate that model was built from 40 occurrences. Currently available CNDDB data indicate 39 occurrences were available for use by this contractor for model development.	Report/data indicate that model was built from 72 occurrences. Currently available CNDDB data indicate 39 occurrences were available for use by this contractor for model development.
	Age of occurrences*		1 of 39 (2%) currently available CNDDB occurrences are from prior to 1981.	1 of 39 (2%) currently available CNDDB occurrences are from prior to 1981.
	Spatial accuracy of occurrences*		1 of 39 (2%) currently available CNDDB occurrences have imprecise spatial accuracy.	1 of 39 (2%) currently available CNDDB occurrences have imprecise spatial accuracy.
	Status of occurrences*		3 of 39 (8%) currently available CNDDB occurrences have Fair or Poor occurrence ranks.	3 of 39 (8%) currently available CNDDB occurrences have Fair or Poor occurrence ranks.
	Species identification of occurrences*			A substantial portion of records appear to be from sources other than CNDDB, for which the reliability of species identification is unknown.

Category	Topic		Contractor B	Contractor C
	Spatial bias of occurrences*			
	Spatial distribution of occurrences*		Currently available CNDDB records in the contractor's boundary are from a limited portion of the occupied geographic subdivisions for the species in California [54].	Currently available CNDDB records in the contractor's boundary are from a limited portion of the occupied geographic subdivisions for the species in California [54].
	Absence data			
Environ-mental covariates	Ecological relevance			
	Comprehensive			
	Resolution and scale			
	Accuracy			
	Number of covariates		Model includes 15 covariates and 40 occurrences.	Model includes 4 covariates and 72 occurrences; report stated that no more than one variable per 10 occurrences was allowed.
	Current covariate data			
	Covariate selection			
	Correlation			
Modeling algorithm	Use in the literature			
	Interactions			

Category	Topic		Contractor B	Contractor C
	Non-linear			
Modeling extent and resolution	Model extent		Contractor's project boundary includes the area of occupied geographic subdivisions for the species in California [54], but not a complete buffer around it. The species also occurs in Nevada.	Contractor's project boundary includes the area of occupied geographic subdivisions for the species in California [54], but not a complete buffer around it. The species also occurs in Nevada.
	Resolution of model output			
Model selection and thresholds	Model selection			
	Selection of threshold for mapping suitable habitat			

Fig A19_*Eriogonum bifurcatum.*

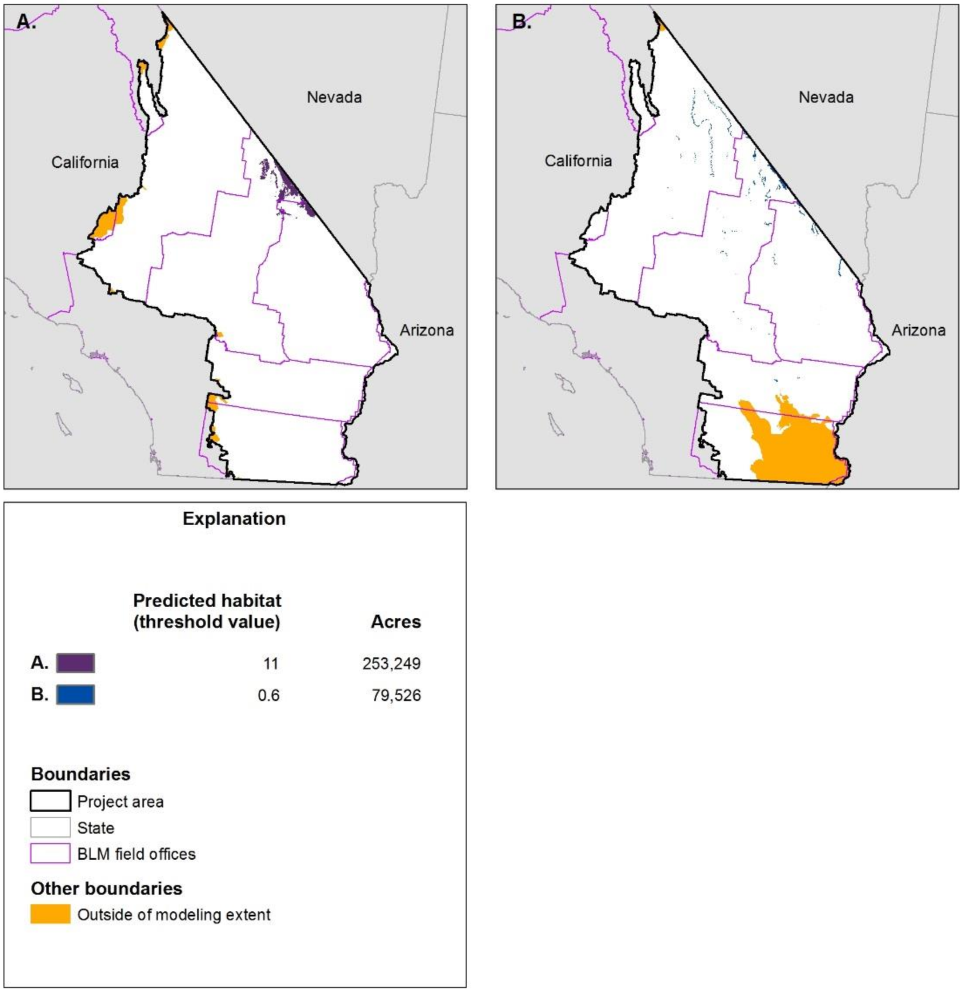

Fig B19_*Eriogonum bifurcatum.*

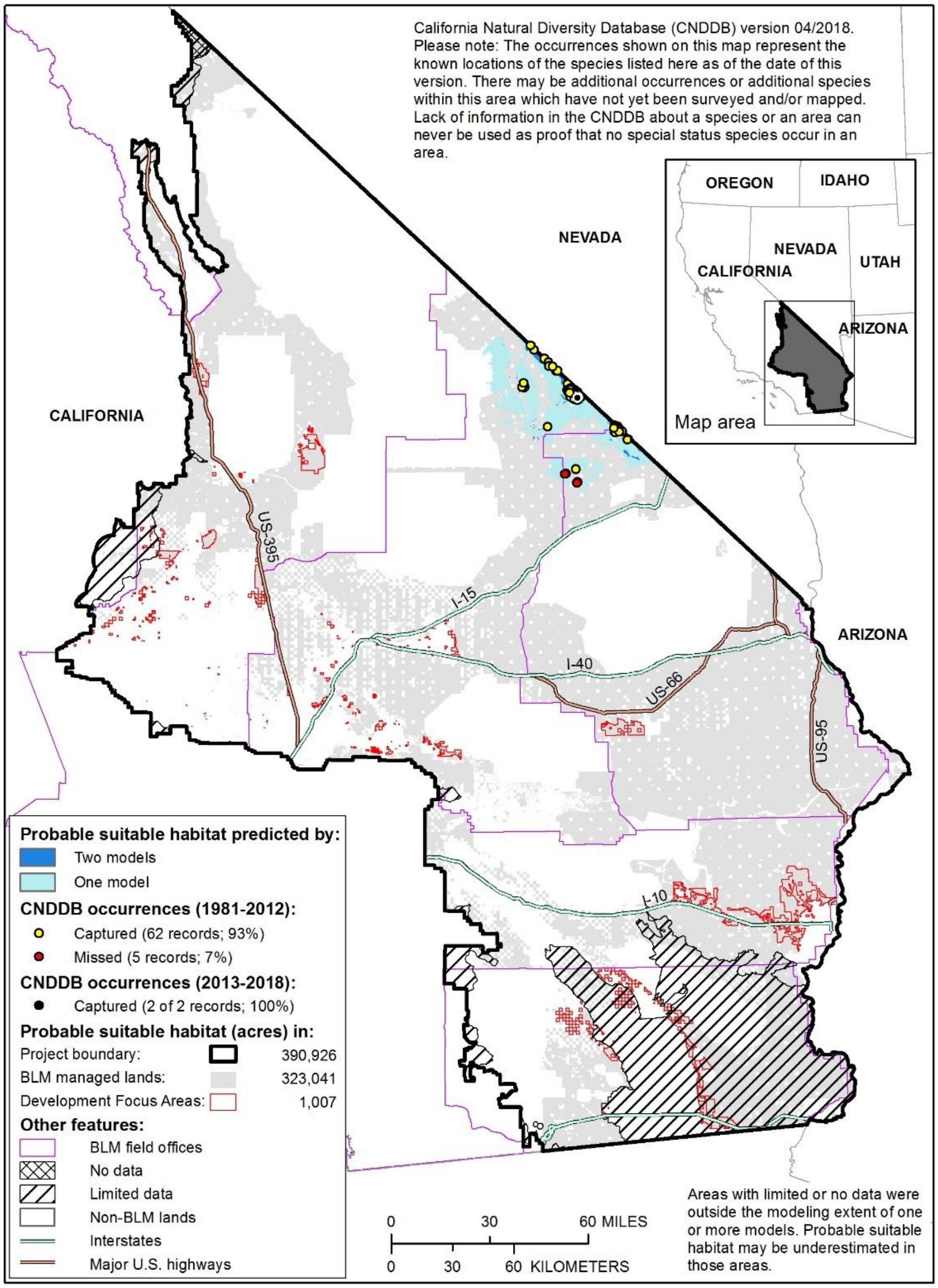

Fig C19_*Eriogonum bifurcatum.*

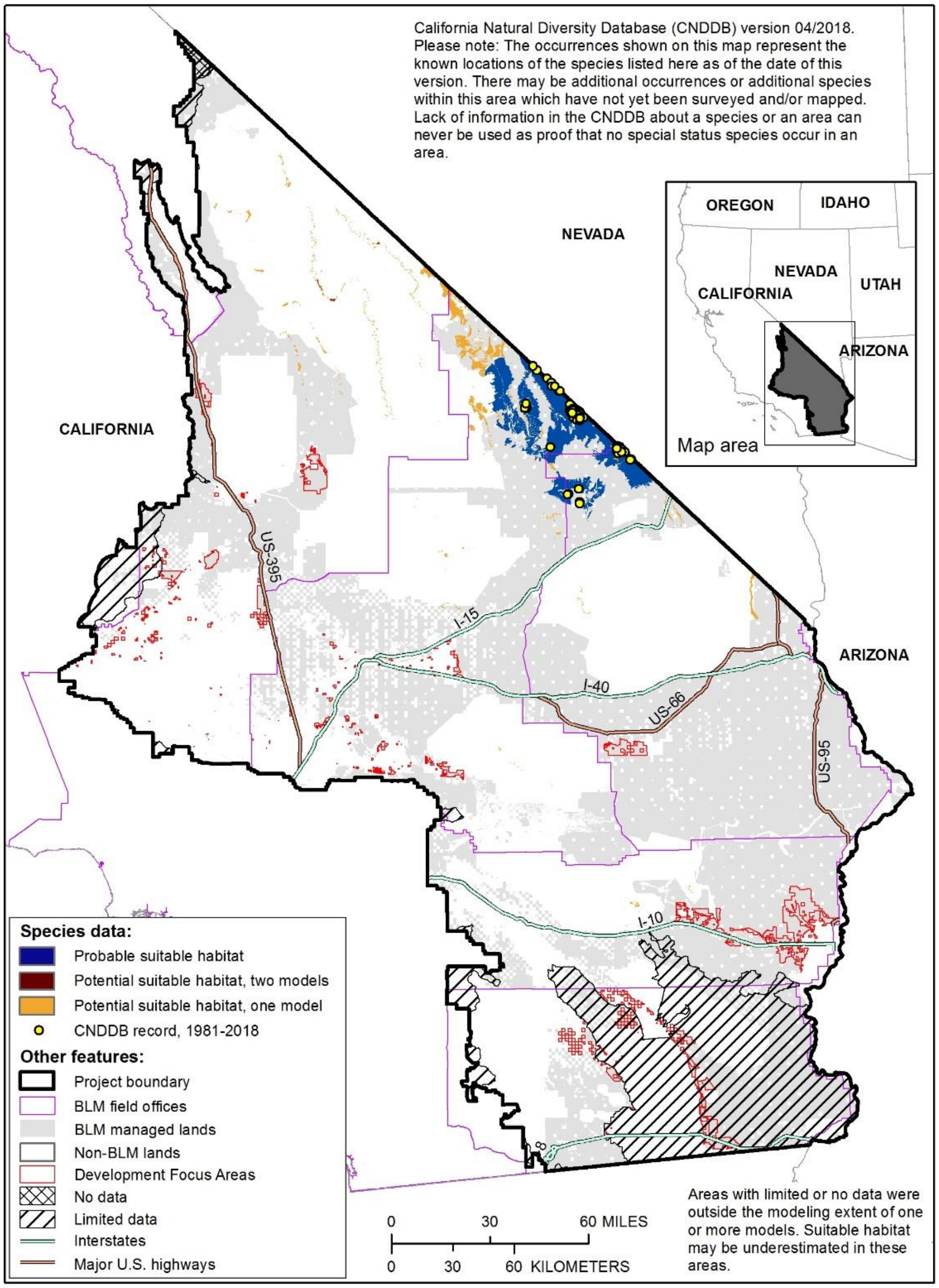

Table B20_*Eriogonum ovalifolium* var. *vineum.*

Category	Topic		Contractor B	Contractor C
Occurrence data used to develop the model	Number of occurrences*		Report/data indicate that model was built from 36 occurrences. Currently available CNDDB data indicate 27 occurrences were available for use by this contractor for model development, but many were outside of the contractor's project boundary.	Report/data indicate that model was built from 57 occurrences. Currently available CNDDB data indicate 27 occurrences were available for use by this contractor for model development.
	Age of occurrences*		3 of 27 (11%) currently available CNDDB occurrences are from prior to 1981.	3 of 27 (11%) currently available CNDDB occurrences are from prior to 1981.
	Spatial accuracy of occurrences*		1 of 27 (4%) currently available CNDDB occurrences have imprecise spatial accuracy.	1 of 27 (4%) currently available CNDDB occurrences have imprecise spatial accuracy.
	Status of occurrences*		6 of 27 (22%) currently available CNDDB occurrences have Fair occurrence ranks.	6 of 27 (22%) currently available CNDDB occurrences have Fair occurrence ranks.
	Species identification of occurrences*			A substantial portion of records appear to be from sources other than CNDDB, for which the reliability of

Category	Topic		Contractor B	Contractor C
				species identification is unknown.
	Spatial bias of occurrences*			
	Spatial distribution of occurrences*		Currently available CNDDB records in the contractor's boundary are from a limited portion of the occupied geographic subdivision for the species in California [54].	Currently available CNDDB records in the contractor's boundary are from a substantial portion of the occupied geographic subdivision for the species in California [54].
	Absence data			
Environ-mental covariates	Ecological relevance			
	Comprehensive			
	Resolution and scale			
	Accuracy			
	Number of covariates		Model includes 15 covariates and 36 occurrences.	Model includes 7 covariates and 57 occurrences; report stated that no more than one variable per 10 occurrences was allowed.
	Current covariate data			
	Covariate selection			
	Correlation			

Category	Topic		Contractor B	Contractor C
Modeling algorithm	Use in the literature			
	Interactions			
	Non-linear			
Modeling extent and resolution	Model extent		Contractor's project boundary excludes a substantial portion of the area of the occupied geographic subdivision for the species in California [54].	Contractor's project boundary includes the area of the occupied geographic subdivision for the species in California [54] and a buffer around it.
	Resolution of model output			
Model selection and thresholds	Model selection			
	Selection of threshold for mapping suitable habitat			

Fig A20_*Eriogonum ovalifolium* var. *vineum.*

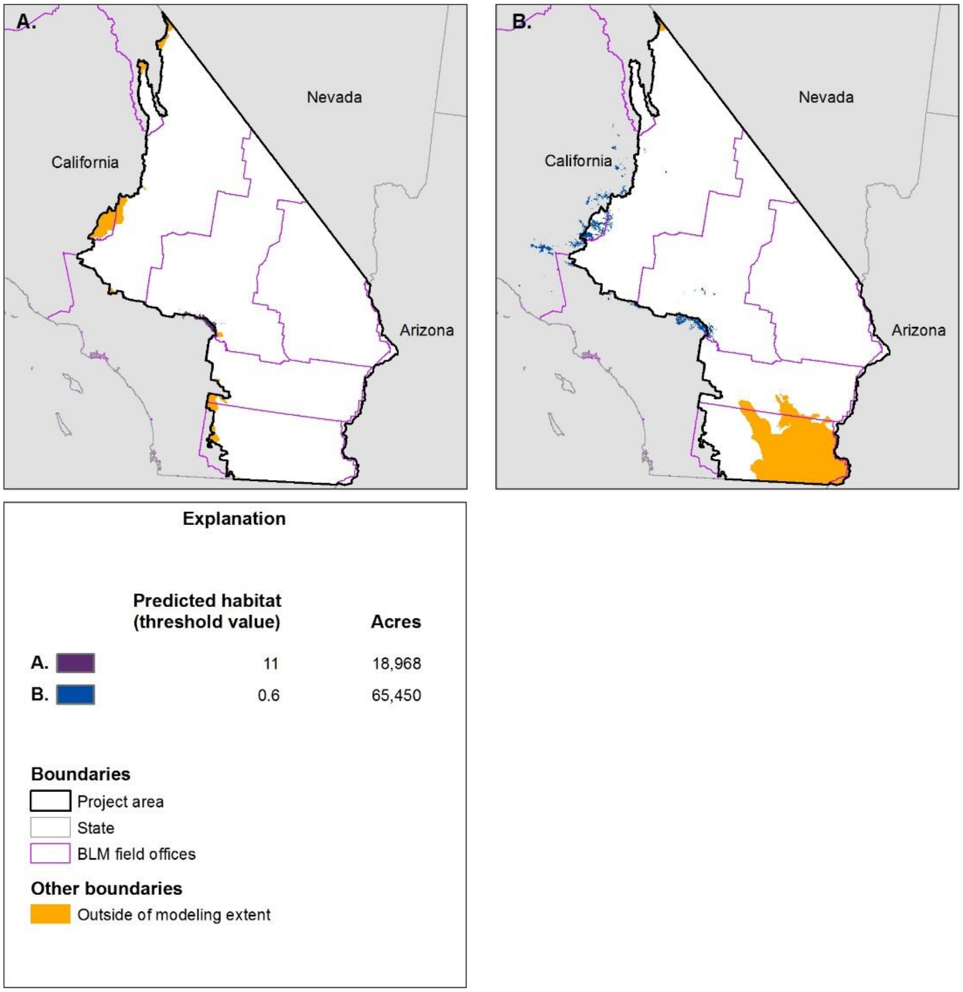

Fig B20_*Eriogonum ovalifolium* var. *vineum.*

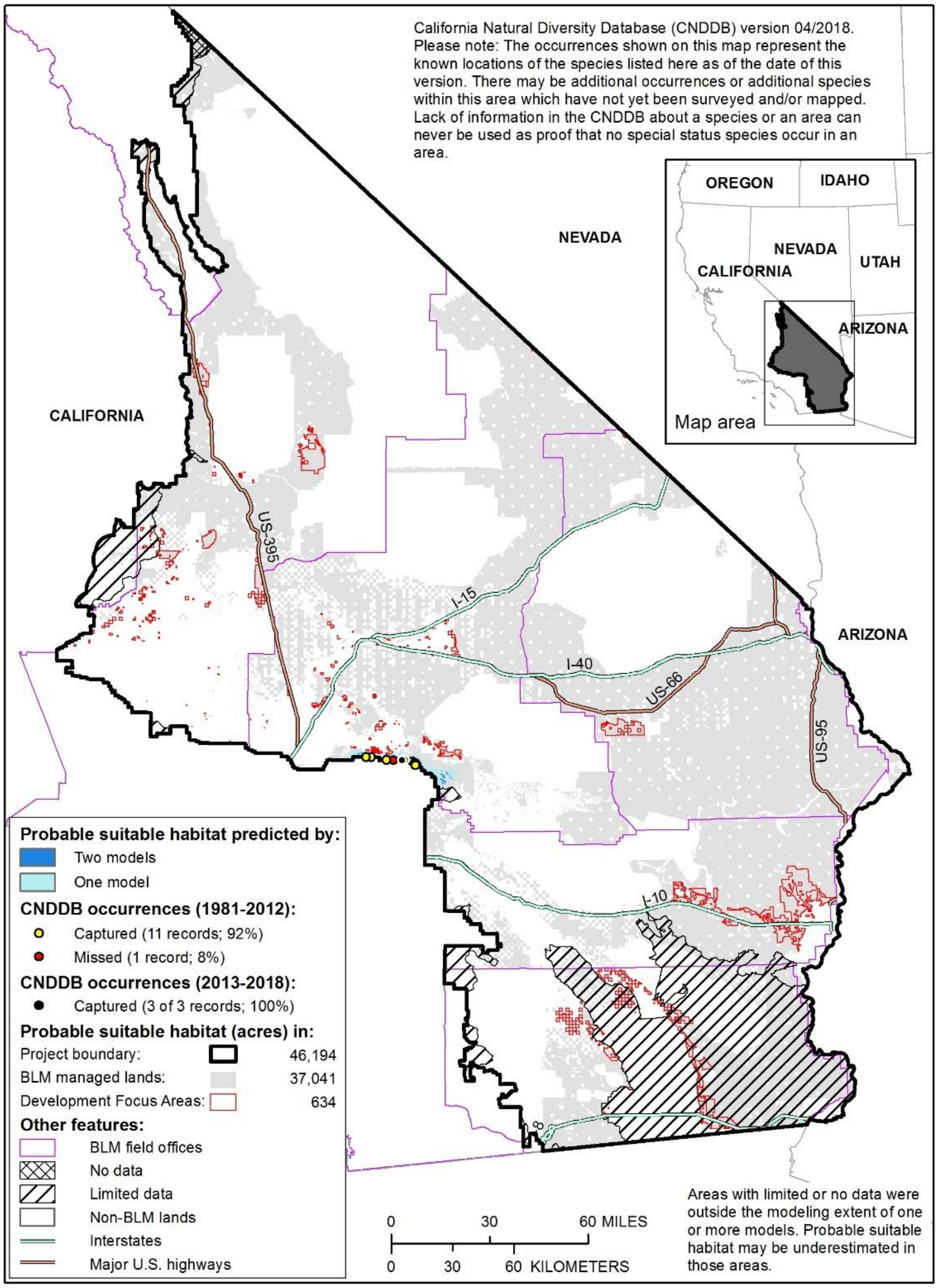

Fig C20_*Eriogonum ovalifolium* var. *vineum.*

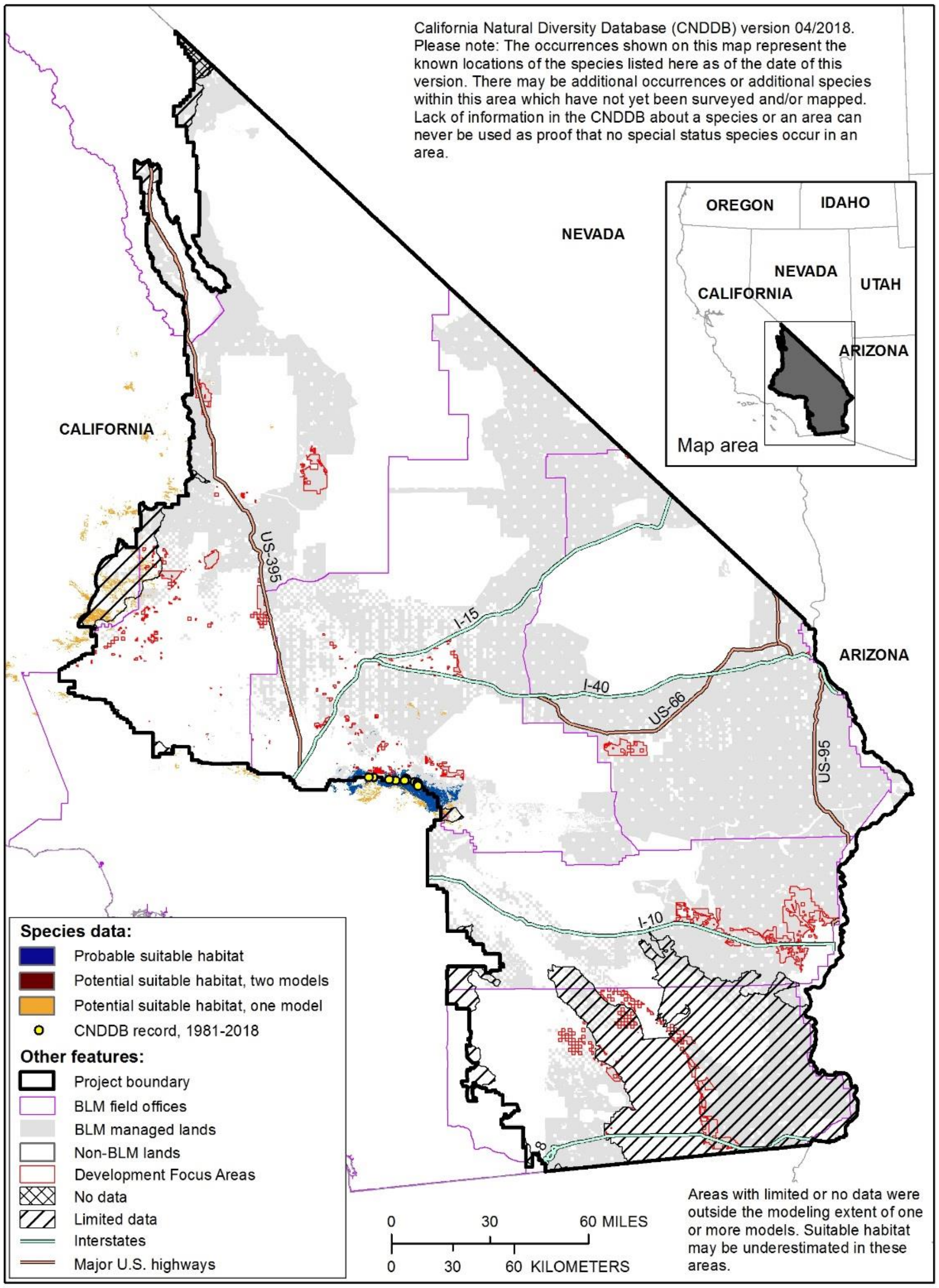

Table B21_*Eriophyllum mohavense.*

Category	Topic	Contractor A	Contractor B	Contractor C
Occurrence data used to develop the model	Number of occurrences*	Report/data indicate that model were built from 212 occurrences. Currently available CNDDB data indicate 61 occurrences were likely used by this contractor for model development.	Report/data indicate that model was built from 67 occurrences. Currently available CNDDB data indicate 64 occurrences were available for use by this contractor for model development.	Report/data indicate that model was built from 85 occurrences. Currently available CNDDB data indicate 64 occurrences were available for use by this contractor for model development.
	Age of occurrences*	Report indicates use of occurrence data from 1981-2012. Many records are from prior to 2000.	3 of 64 (4%) currently available CNDDB occurrences are from prior to 1981.	3 of 64 (4%) currently available CNDDB occurrences are from prior to 1981.
	Spatial accuracy of occurrences*	Report/data indicate occurrences with uncertainty >250-500 m were excluded.	9 of 64 (14%) currently available CNDDB occurrences have imprecise spatial accuracy.	9 of 64 (14%) currently available CNDDB occurrences have imprecise spatial accuracy.
	Status of occurrences*	12 of 61 (20%) currently available CNDDB occurrences have Fair occurrence ranks.	12 of 64 (19%) currently available CNDDB occurrences have Fair occurrence ranks.	12 of 64 (19%) currently available CNDDB occurrences have Fair occurrence ranks.
	Species identification of occurrences*			A substantial portion of records appear to be from sources other than CNDDB, for which the reliability of species

Category	Topic	Contractor A	Contractor B	Contractor C
				identification is unknown.
	Spatial bias of occurrences*			
	Spatial distribution of occurrences*	Currently available CNDDB records in the contractor's boundary are from a substantial portion of the occupied geographic subdivision for the species in California [54].	Currently available CNDDB records in the contractor's boundary are from a substantial portion of the occupied geographic subdivision for the species in California [54].	Currently available CNDDB records in the contractor's boundary are from a substantial portion of the occupied geographic subdivision for the species in California [54].
	Absence data			
Environ-mental covariates	Ecological relevance			
	Comprehensive			
	Resolution and scale			
	Accuracy			
	Number of covariates	It is not clear from the report/data how many covariates are in the model, but it is not more than 22; there are 212 occurrences.	Model includes 8 covariates and 67 occurrences.	Model includes 7 covariates and 85 occurrences; report stated that no more than one variable per 10 occurrences was allowed.
	Current covariate data			
	Covariate selection			
	Correlation			

Category	Topic	Contractor A	Contractor B	Contractor C
Modeling algorithm	Use in the literature			
	Interactions			
	Non-linear			
Modeling extent and resolution	Model extent	Contractor’s project boundary appears to include most or all of the occupied geographic subdivision for the species in California [54] but not a complete buffer around it.	Contractor’s project boundary appears to include most or all of the occupied geographic subdivision for the species in California [54] but not a complete buffer around it.	Contractor’s project boundary appears to include most or all of the occupied geographic subdivision for the species in California [54] but not a complete buffer around it.
	Resolution of model output			
Model selection and thresholds	Model selection			
	Selection of threshold for mapping suitable habitat			

Fig A21_*Eriophyllum mohavense*.

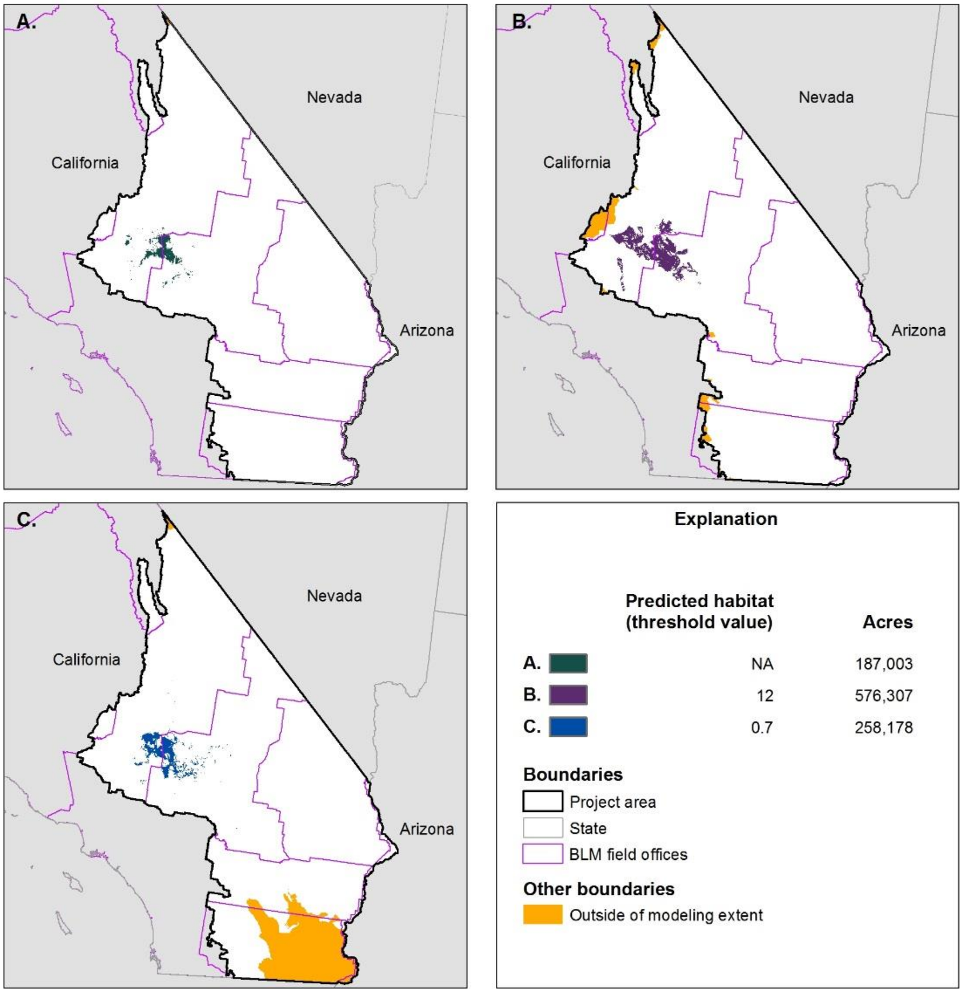

Fig B21_*Eriophyllum mohavense.*

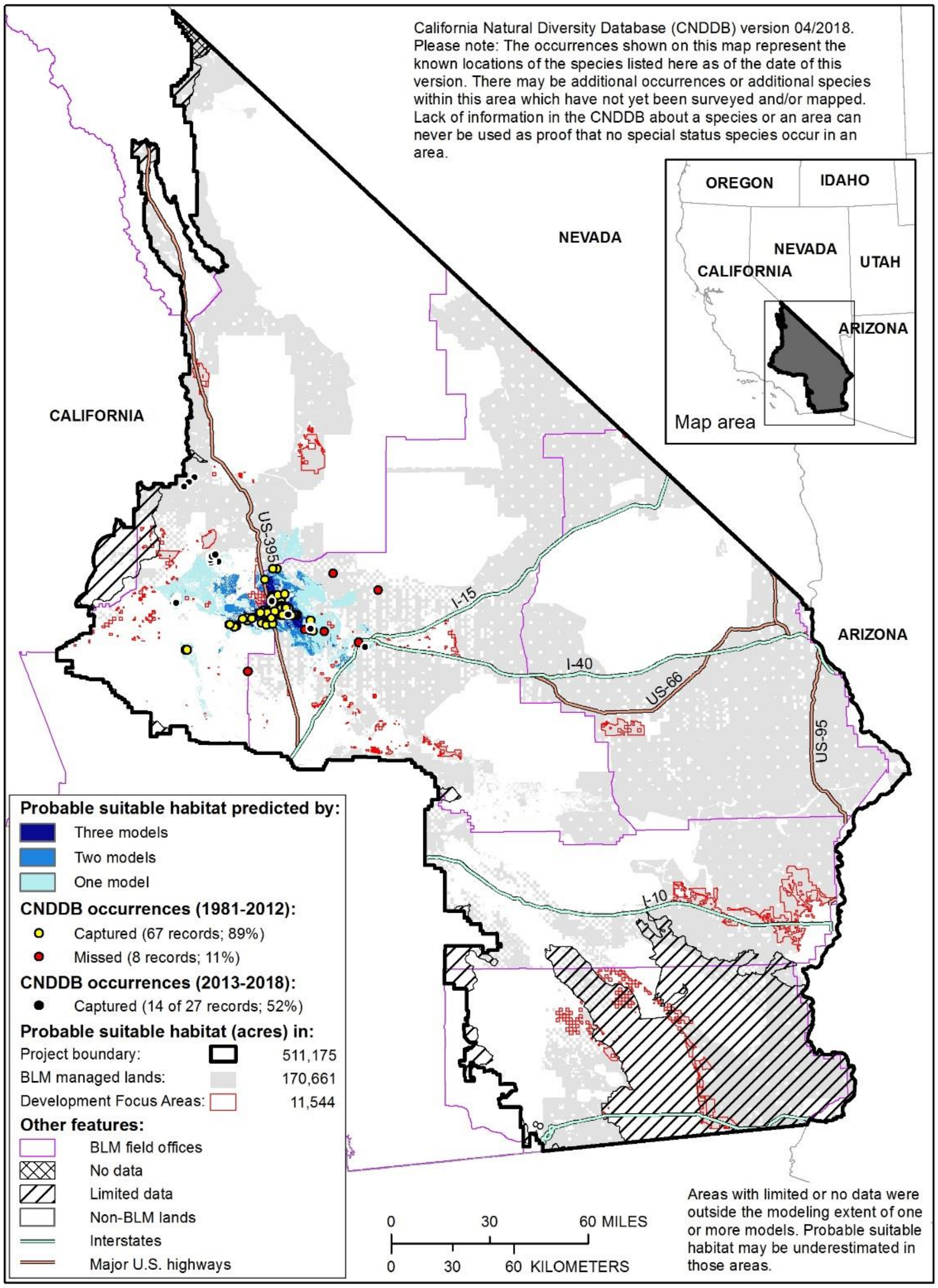

Fig C21_*Eriophyllum mohavense.*

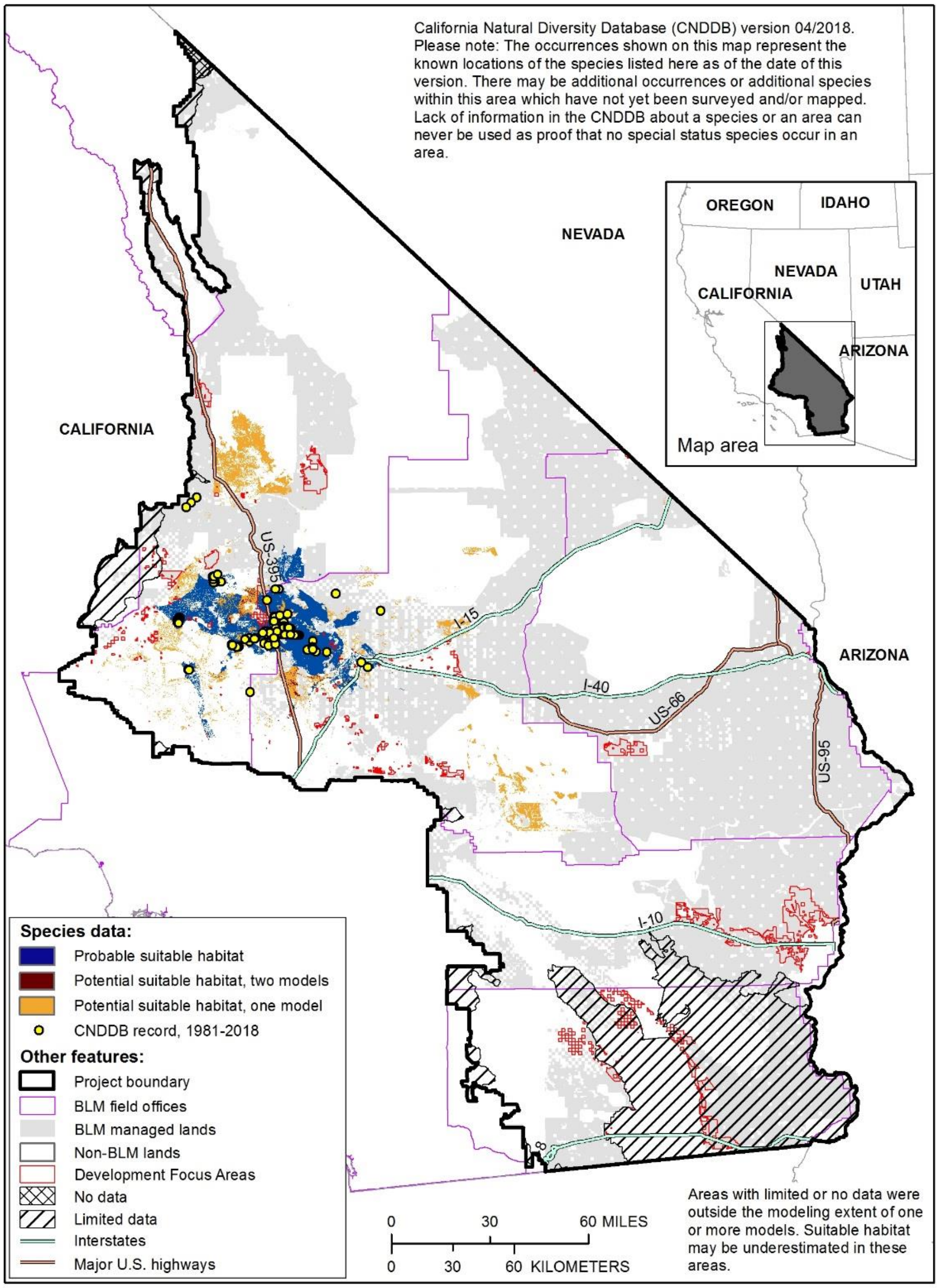

Table B22_*Erythranthe shevockii* (now *Mimulus shevockii*).

Category	Topic	Contractor A		
Occurrence data used to develop the model	Number of occurrences*	It is not clear from the report/data how many occurrences were used to build the model. Currently available CNDDB data indicate 11 occurrences were likely used by this contractor for model development.		
	Age of occurrences*	Report indicates use of occurrence data from 1981-2012. 2 of 11 records are from prior to 2000.		
	Spatial accuracy of occurrences*	Report/data indicate occurrences with uncertainty >250-500 m were excluded.		
	Status of occurrences*	1 of 11 (9%) currently available CNDDB occurrences has a Fair occurrence rank.		
	Species identification of occurrences*			
	Spatial bias of occurrences*			

Category	Topic	Contractor A		
	Spatial distribution of occurrences*	Currently available CNDDB records in the contractor's boundary are from a limited portion of the occupied geographic subdivisions for the species in California [54].		
	Absence data			
Environ-mental covariates	Ecological relevance			
	Comprehensive			
	Resolution and scale			
	Accuracy			
	Number of covariates	It is not clear from the report/data how many covariates or occurrences are in the model.		
	Current covariate data			
	Covariate selection			
	Correlation			
Modeling algorithm	Use in the literature			
	Interactions			
	Non-linear			

Category	Topic	Contractor A		
Modeling extent and resolution	Model extent	Contractor’s project boundary includes most of the occupied geographic subdivisions for the species in California [54].		
	Resolution of model output			
Model selection and thresholds	Model selection			
	Selection of threshold for mapping suitable habitat			

Fig A22_*Erythranthe shevockii* (now *Mimulus shevockii*).

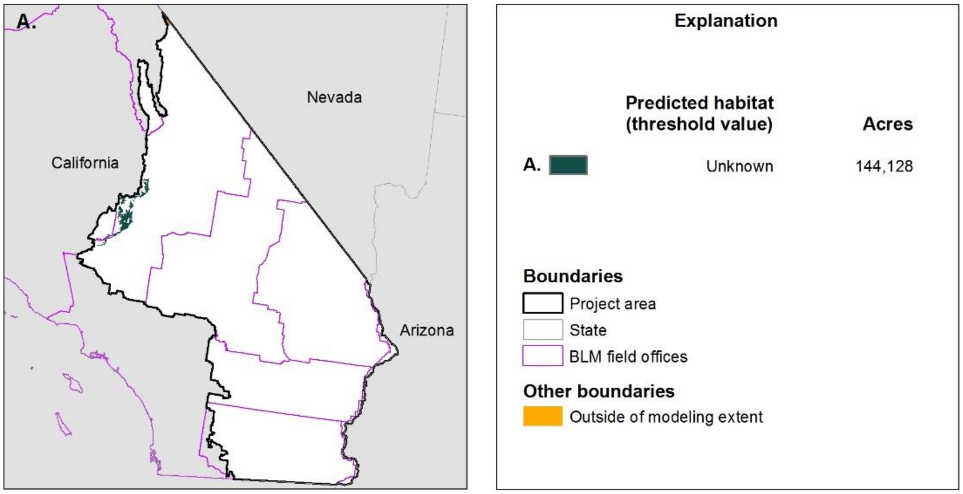

Fig C22_*Erythranthe shevockii* (now *Mimulus shevockii*).

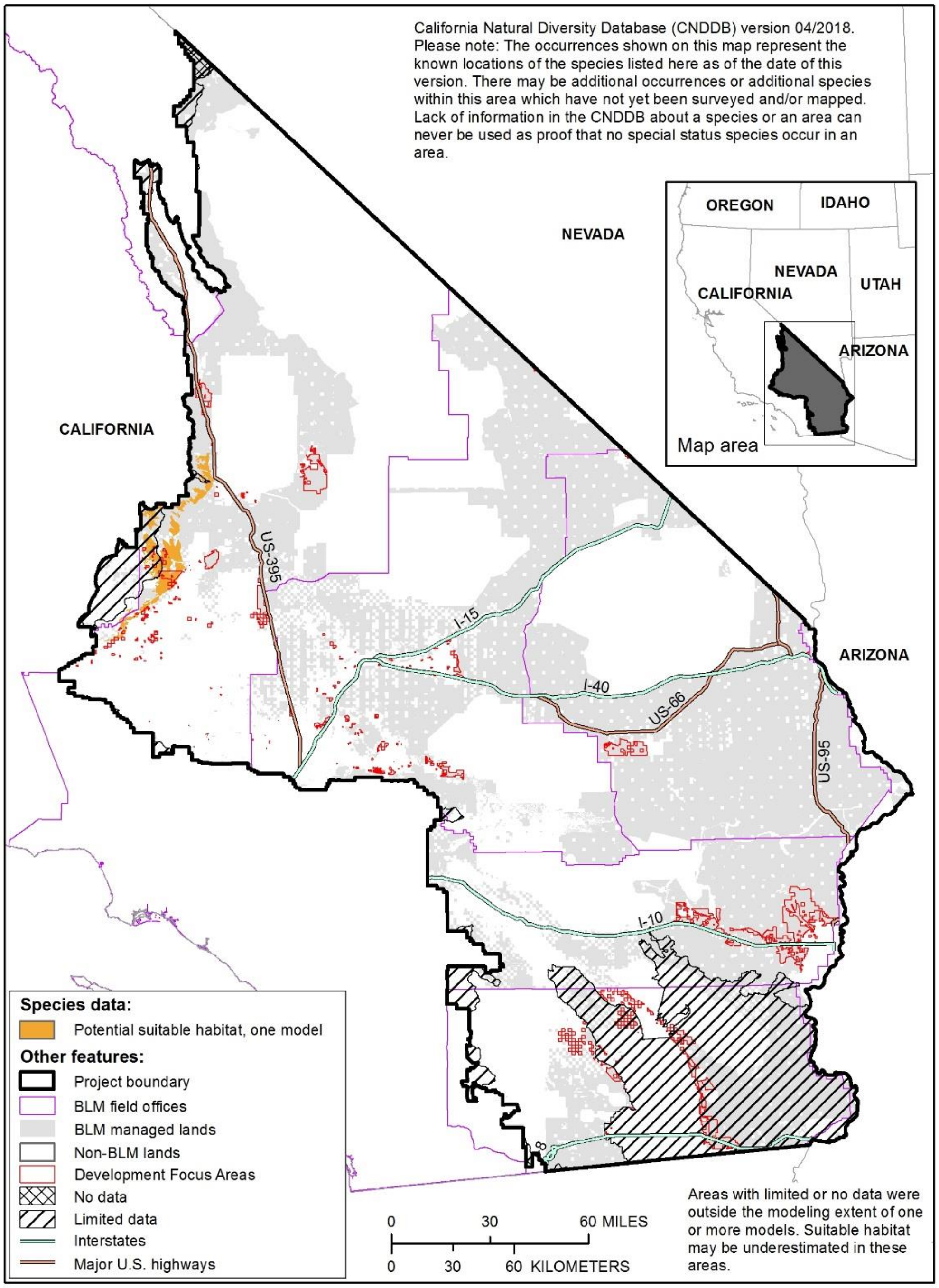

Table B23_*Eschscholzia minutiflora* ssp. *twisselmannii*.

Category	Topic	Contractor A	Contractor B	Contractor C
Occurrence data used to develop the model	Number of occurrences*	Report/data indicate that model were built from 146 occurrences. Currently available CNDDB data indicate 24 occurrences were likely used by this contractor for model development.	Report/data indicate that model was built from 26 occurrences. Currently available CNDDB data indicate 26 occurrences were available for use by this contractor for model development.	Report/data indicate that model was built from 34 occurrences. Currently available CNDDB data indicate 26 occurrences were available for use by this contractor for model development.
	Age of occurrences*	Report indicates use of occurrence data from 1981-2012. Many records are from prior to 2000.	2 of 26 (8%) currently available CNDDB occurrences are from prior to 1981.	2 of 26 (8%) currently available CNDDB occurrences are from prior to 1981.
	Spatial accuracy of occurrences*	Report/data indicate occurrences with uncertainty >250-500 m were excluded.	2 of 26 (8%) currently available CNDDB occurrences have imprecise spatial accuracy.	2 of 26 (8%) currently available CNDDB occurrences have imprecise spatial accuracy.
	Status of occurrences*	7 of 24 (29%) currently available CNDDB occurrences have Fair or Poor occurrence ranks.	7 of 26 (27%) currently available CNDDB occurrences have Fair or Poor occurrence ranks.	7 of 26 (27%) currently available CNDDB occurrences have Fair or Poor occurrence ranks.
	Species identification of occurrences*			A substantial portion of records appear to be from sources other than CNDDB, for which the reliability of species

Category	Topic	Contractor A	Contractor B	Contractor C
				identification is unknown.
	Spatial bias of occurrences*			
	Spatial distribution of occurrences*	Currently available CNDDB records in the contractor's boundary are from a limited portion of the area of the occupied geographic subdivisions for the species in California [54].	Currently available CNDDB records in the contractor's boundary are from a limited portion of the area of the occupied geographic subdivisions for the species in California [54].	Currently available CNDDB records in the contractor's boundary are from a limited portion of the area of the occupied geographic subdivisions for the species in California [54].
	Absence data			
Environ-mental covariates	Ecological relevance			
	Comprehensive			
	Resolution and scale			
	Accuracy			
	Number of covariates	Model includes 13 covariates and 146 occurrences.	Model includes 15 covariates and 26 occurrences.	Model includes 4 covariates and 34 occurrences; report stated that no more than one variable per 10 occurrences was allowed.
	Current covariate data			
	Covariate selection			
	Correlation			

Category	Topic	Contractor A	Contractor B	Contractor C
Modeling algorithm	Use in the literature			
	Interactions			
	Non-linear			
Modeling extent and resolution	Model extent	Contractor's project boundary excludes a significant portion of the occupied geographic subdivisions for the species in California [54].	Contractor's project boundary excludes a significant portion of the occupied geographic subdivisions for the species in California [54].	Contractor's project boundary excludes a significant portion of the occupied geographic subdivisions for the species in California [54].
	Resolution of model output			
Model selection and thresholds	Model selection			
	Selection of threshold for mapping suitable habitat			

Fig A23_ *Eschscholzia minutiflora* ssp. *twisselmannii.*

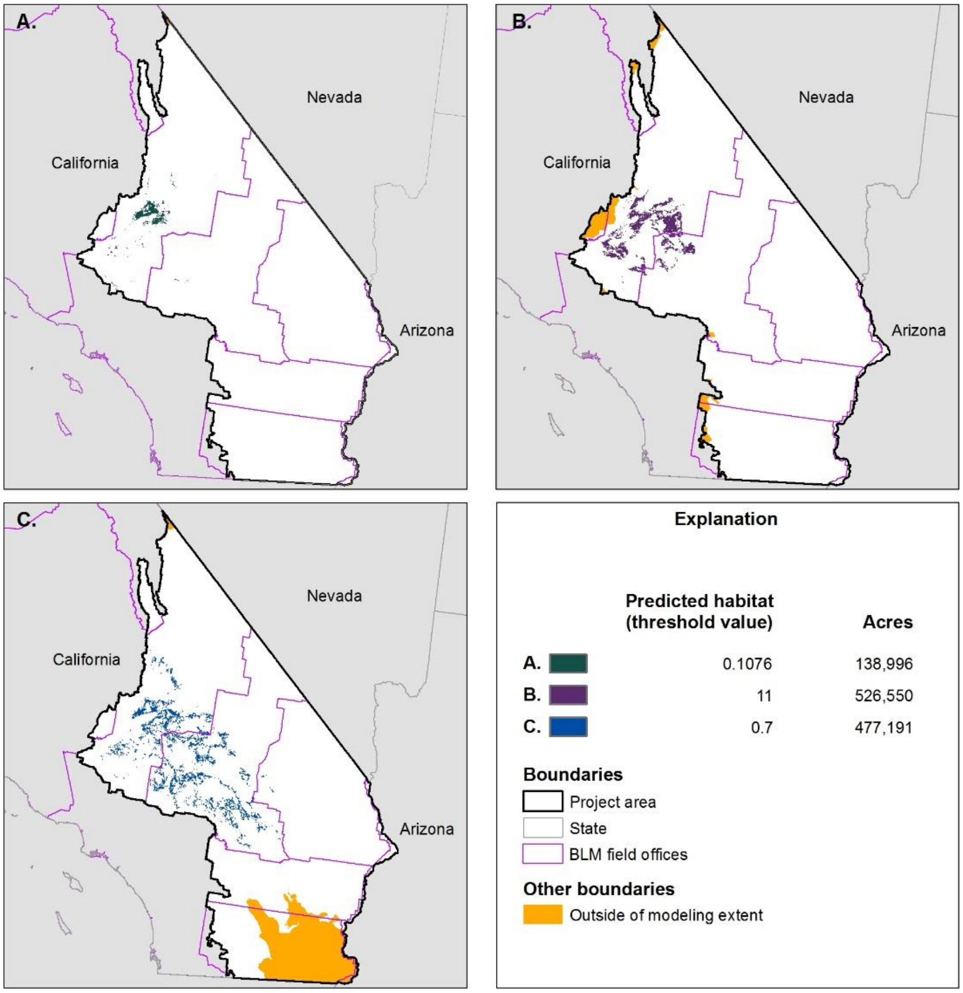

Fig B23_ *Eschscholzia minutiflora* ssp. *twisselmannii.*

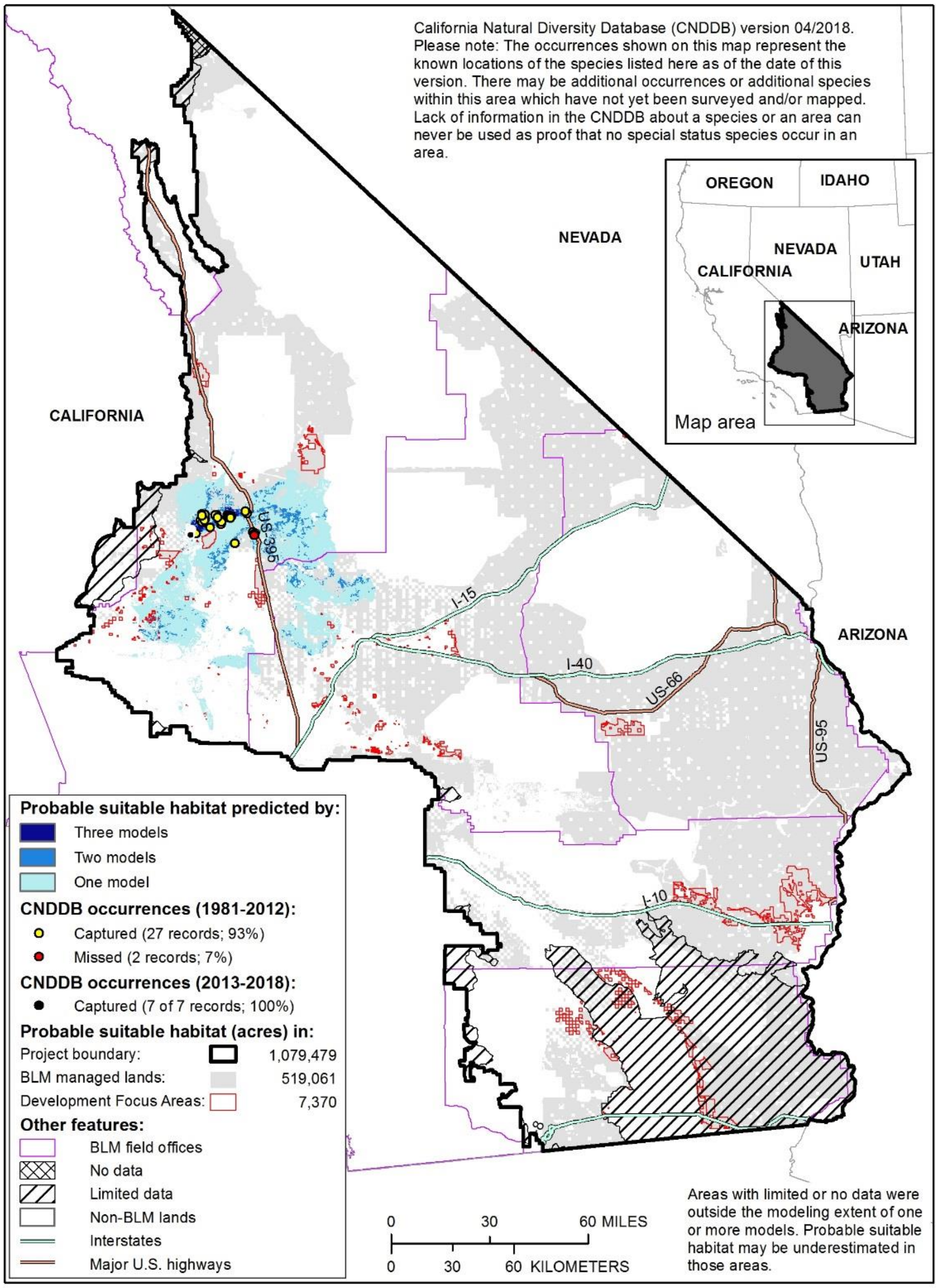

Fig C23_ *Eschscholzia minutiflora* ssp. *twisselmannii*.

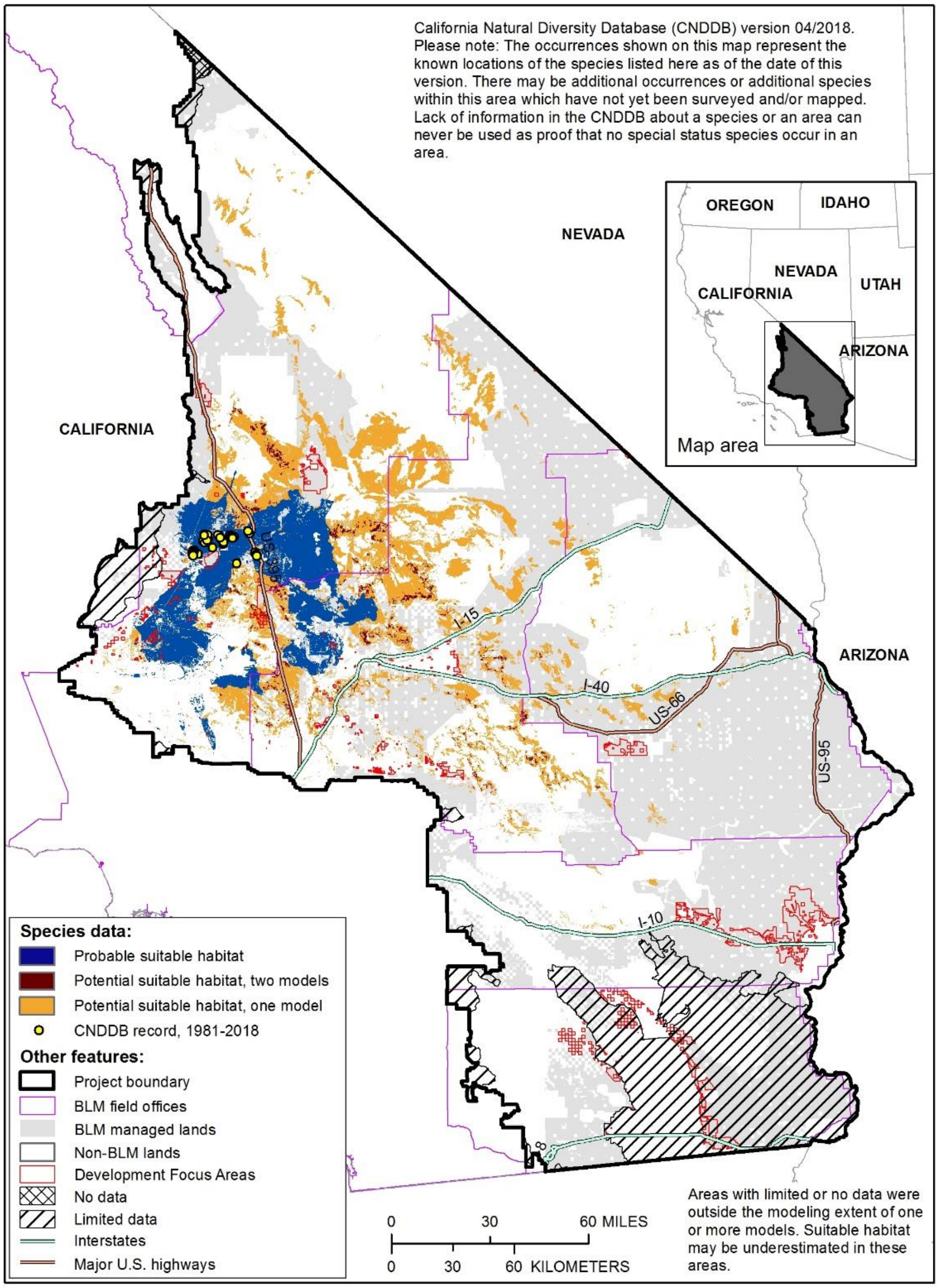

Table B24_*Grindelia fraxinipratensis.*

Category	Topic		Contractor B	
Occurrence data used to develop the model	Number of occurrences*		Report/data indicate that model was built from 3 occurrences. Currently available CNDDB data indicate 3 occurrences were available for use by this contractor for model development.	
	Age of occurrences*		0 of 3 (0%) currently available CNDDB occurrences are from prior to 1981.	
	Spatial accuracy of occurrences*		2 of 3 (67%) currently available CNDDB occurrences have imprecise spatial accuracy.	
	Status of occurrences*		0 of 3 (0%) currently available CNDDB occurrences have Fair or Poor occurrence ranks.	
	Species identification of occurrences*			
	Spatial bias of occurrences*			

Category	Topic		Contractor B	
	Spatial distribution of occurrences*		Currently available CNDDB records in the contractor's boundary are from a limited portion of the area of the occupied geographic subdivisions for the species in California [54].	
	Absence data			
Environ-mental covariates	Ecological relevance			
	Comprehensive			
	Resolution and scale			
	Accuracy			
	Number of covariates		Model includes 14 covariates and 3 occurrences.	
	Current covariate data			
	Covariate selection			
	Correlation			
Modeling algorithm	Use in the literature			
	Interactions			
	Non-linear			

Category	Topic		Contractor B	
Modeling extent and resolution	Model extent		Contractor's project boundary most of the occupied geographic subdivisions for the species in California [54], but not a complete buffer around them.	
	Resolution of model output			
Model selection and thresholds	Model selection			
	Selection of threshold for mapping suitable habitat			

Fig A24_*Grindelia fraxinipratensis*.

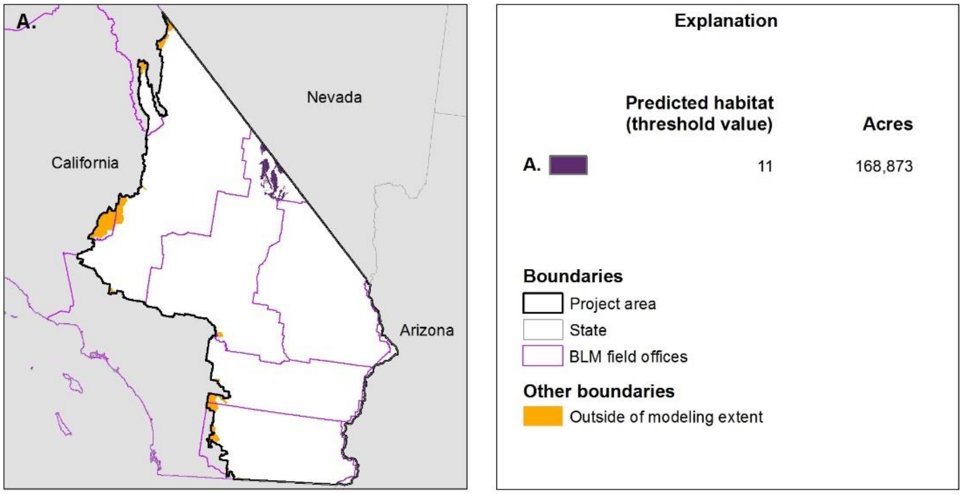

Fig C24_*Grindelia fraxinipratensis*.

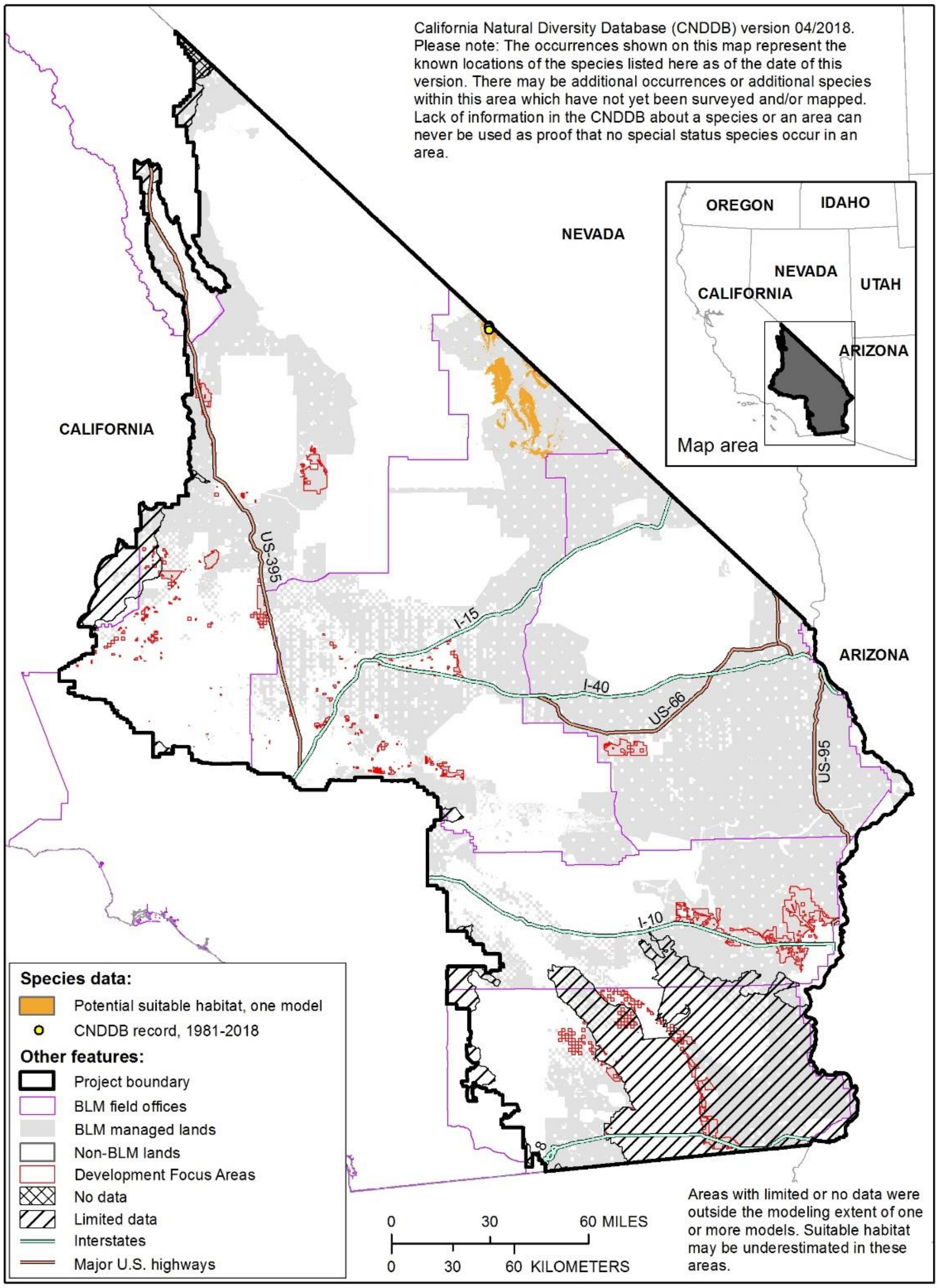

Table B25_*Heuchera brevistaminea*.

Category	Topic		Contractor B	
Occurrence data used to develop the model	Number of occurrences*		Report/data indicate that model was built from 3 occurrences. Currently available CNDDB data indicate 9 occurrences were available for use by this contractor for model development.	
	Age of occurrences*		2 of 9 (22%) currently available CNDDB occurrences are from prior to 1981.	
	Spatial accuracy of occurrences*		0 of 9 (0%) currently available CNDDB occurrences have imprecise spatial accuracy.	
	Status of occurrences*		0 of 9 (0%) currently available CNDDB occurrences have Fair or Poor occurrence ranks.	
	Species identification of occurrences*			
	Spatial bias of occurrences*			

Category	Topic		Contractor B	
	Spatial distribution of occurrences*		Currently available CNDDB records in the contractor’s boundary are from a limited portion of the area of the occupied geographic subdivisions for the species in California [54].	
	Absence data			
Environ-mental covariates	Ecological relevance			
	Comprehensive			
	Resolution and scale			
	Accuracy			
	Number of covariates		Model includes 9 covariates and 3 occurrences.	
	Current covariate data			
	Covariate selection			
	Correlation			
Modeling algorithm	Use in the literature			
	Interactions			
	Non-linear			

Category	Topic		Contractor B	
Modeling extent and resolution	Model extent		Contractor’s project boundary excludes most of the occupied geographic subdivision for the species in California [54].	
	Resolution of model output			
Model selection and thresholds	Model selection			
	Selection of threshold for mapping suitable habitat			

Fig A25_*Heuchera brevistaminea*.

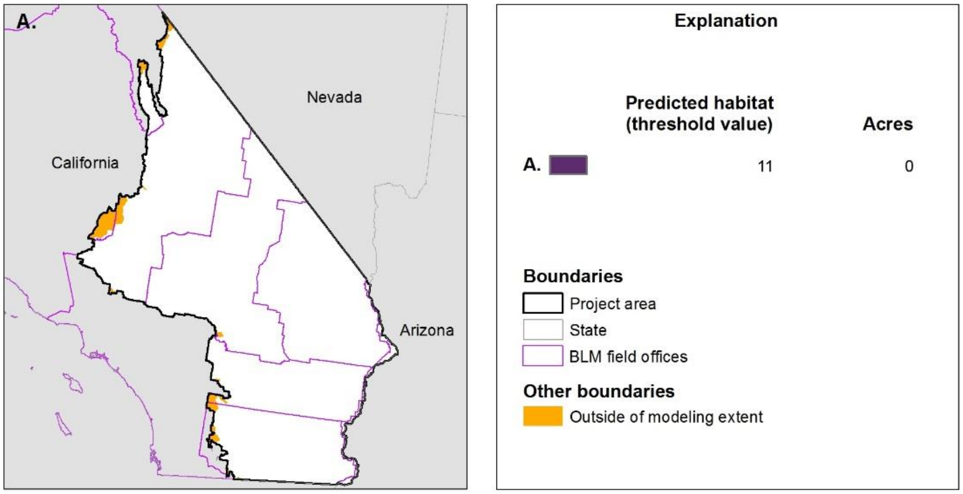

Fig C25_*Heuchera brevistaminea.*

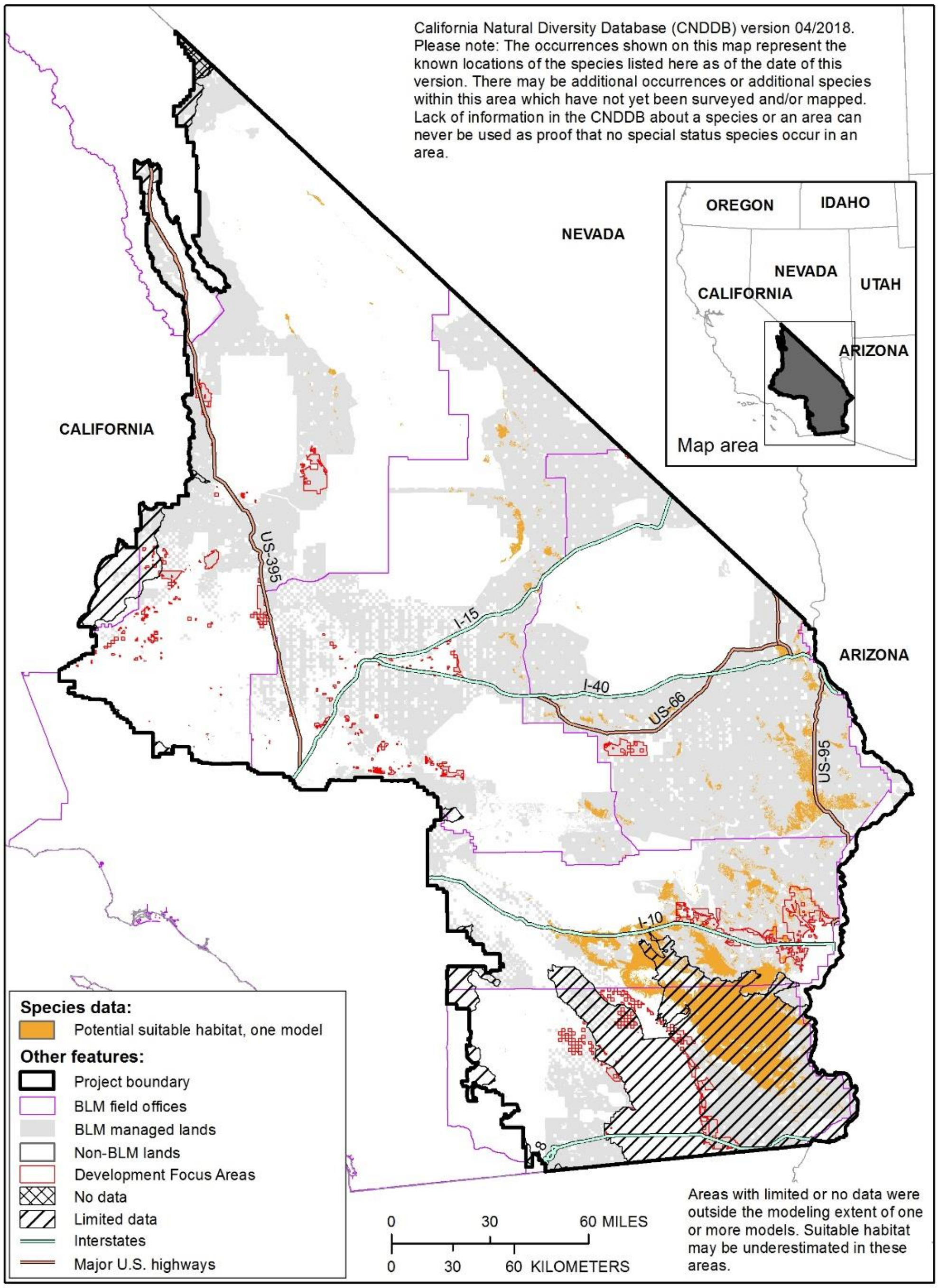

Table B26_*Layia heterotricha*.

Category	Topic	Contractor A		
Occurrence data used to develop the model	Number of occurrences*	Report/data indicate that the model was built from 30 occurrences. Currently available CNDDB data indicate 48 occurrences were likely used by this contractor for model development.		
	Age of occurrences*	Report indicates use of occurrence data from 1981-2012. Multiple records are from prior to 2000.		
	Spatial accuracy of occurrences*	Report/data indicate occurrences with uncertainty >250-500 m were excluded.		
	Status of occurrences*	5 of 48 (10%) currently available CNDDB occurrences have Fair or Poor occurrence ranks.		
	Species identification of occurrences*			
	Spatial bias of occurrences*			

Category	Topic	Contractor A		
	Spatial distribution of occurrences*	Currently available CNDDB records in the contractor's boundary are from a limited portion of the area of the occupied geographic subdivisions for the species in California [54].		
	Absence data			
Environ-mental covariates	Ecological relevance			
	Comprehensive			
	Resolution and scale			
	Accuracy			
	Number of covariates	Model includes 13 covariates and 30 occurrences.		
	Current covariate data			
	Covariate selection			
	Correlation			
Modeling algorithm	Use in the literature			
	Interactions			
	Non-linear			

Category	Topic	Contractor A		
Modeling extent and resolution	Model extent	Contractor's project boundary excludes a significant portion of the occupied geographic subdivisions for the species in California [54].		
	Resolution of model output			
Model selection and thresholds	Model selection			
	Selection of threshold for mapping suitable habitat			

Fig A26_*Layia heterotricha*.

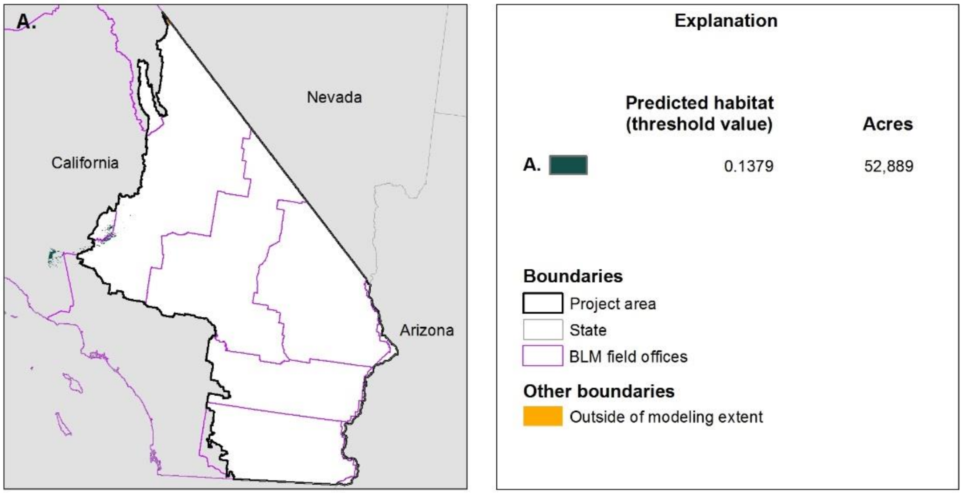

Fig B26_*Layia heterotricha*.

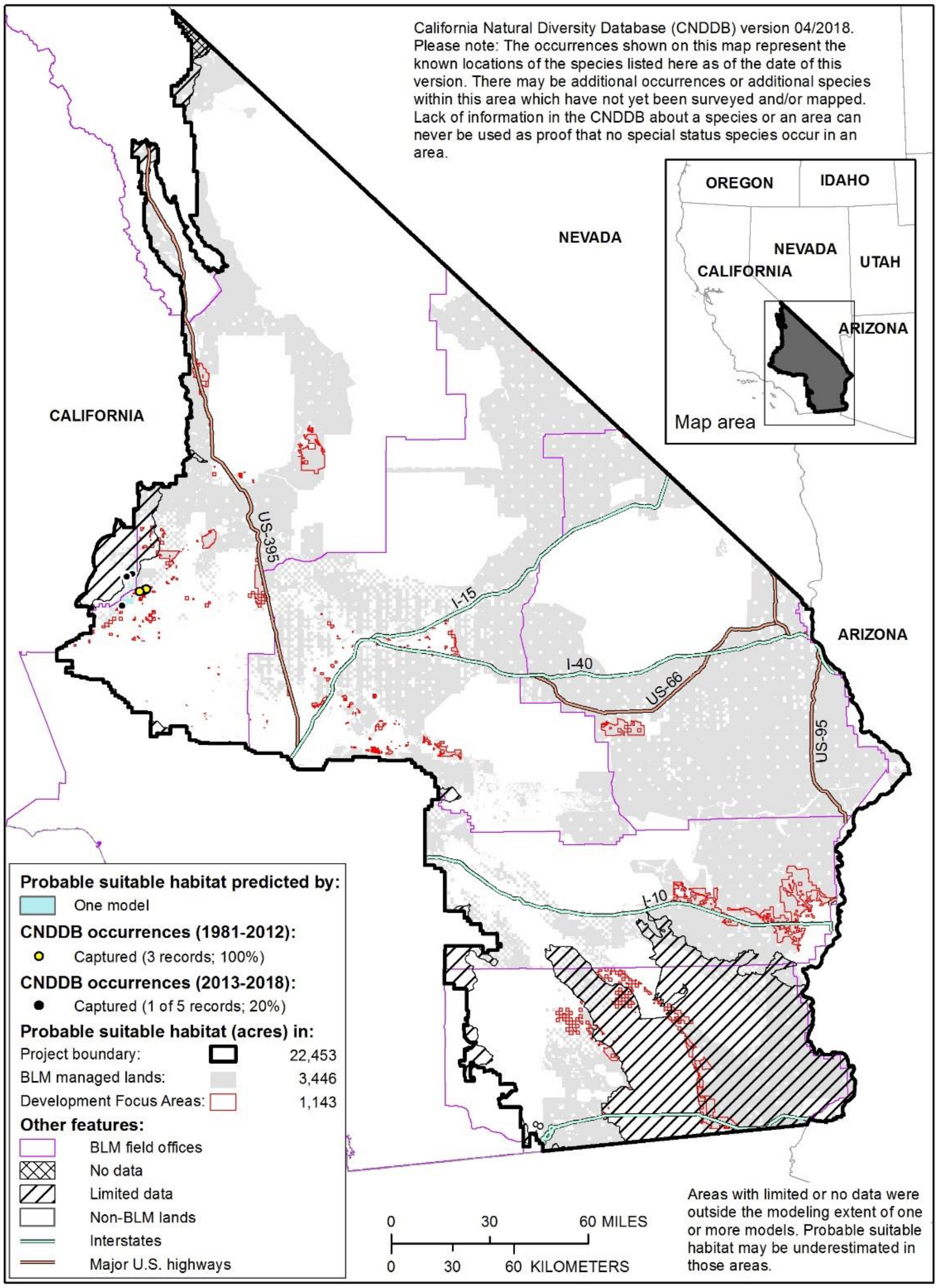

Fig C26_*Layia heterotricha*. No map produced; all existing model information contained in map of probable suitable habitat.

Table B27_*Linanthus maculatus*.

Category	Topic	Contractor A	Contractor B	Contractor C
Occurrence data used to develop the model	Number of occurrences*	Data do not indicate the number of occurrences used to build the model. Currently available CNDDB data indicate 32 occurrences were likely used by this contractor for model development.	Report/data indicate that model was built from 39 occurrences. Currently available CNDDB data indicate 37 occurrences were available for use by this contractor for model development.	Report/data indicate that model was built from 117 occurrences. Currently available CNDDB data indicate 37 occurrences were available for use by this contractor for model development.
	Age of occurrences*	Report indicates use of occurrence data from 1981-2012. Numerous records are from prior to 2000.	5 of 37 (14%) currently available CNDDB occurrences are from prior to 1981.	5 of 37 (14%) currently available CNDDB occurrences are from prior to 1981.
	Spatial accuracy of occurrences*	Report/data indicate occurrences with uncertainty >250-500 m were excluded.	14 of 37 (38%) currently available CNDDB occurrences have imprecise spatial accuracy.	14 of 37 (38%) currently available CNDDB occurrences have imprecise spatial accuracy.
	Status of occurrences*	4 of 32 (12%) currently available CNDDB occurrences have Fair or Poor occurrence ranks.	4 of 37 (11%) currently available CNDDB occurrences have Fair or Poor occurrence ranks.	4 of 37 (11%) currently available CNDDB occurrences have Fair or Poor occurrence ranks.
	Species identification of occurrences*			A substantial portion of records appear to be from sources other than CNDDB, for which the reliability of species

Category	Topic	Contractor A	Contractor B	Contractor C
				identification is unknown.
	Spatial bias of occurrences*			
	Spatial distribution of occurrences*	Currently available CNDDB records in the contractor's boundary are from a limited portion of the area of the occupied geographic subdivisions for the species in California [54].	Currently available CNDDB records in the contractor's boundary are from a limited portion of the area of the occupied geographic subdivisions for the species in California [54].	Currently available CNDDB records in the contractor's boundary are from a limited portion of the area of the occupied geographic subdivisions for the species in California [54].
	Absence data			
Environ-mental covariates	Ecological relevance			
	Comprehensive			
	Resolution and scale			
	Accuracy			
	Number of covariates	Model includes 7 covariates. The number of occurrences is unknown, but likely at least 32.	Model includes 18 covariates and 39 occurrences.	Model includes 7 covariates and 117 occurrences; report stated that no more than one variable per 10 occurrences was allowed.
	Current covariate data			
	Covariate selection			
	Correlation			

Category	Topic	Contractor A	Contractor B	Contractor C
Modeling algorithm	Use in the literature			
	Interactions			
	Non-linear			
Modeling extent and resolution	Model extent	Contractor's project boundary includes most or all of the occupied geographic subdivisions for the species in California [54], but not a full buffer around them.	Contractor's project boundary includes most of the occupied geographic subdivisions for the species in California [54], but not a full buffer around them.	Contractor's project boundary includes most or all of the occupied geographic subdivisions for the species in California [54], but not a full buffer around them.
	Resolution of model output			
Model selection and thresholds	Model selection			
	Selection of threshold for mapping suitable habitat			

Fig A27_*Linanthus maculatus*.

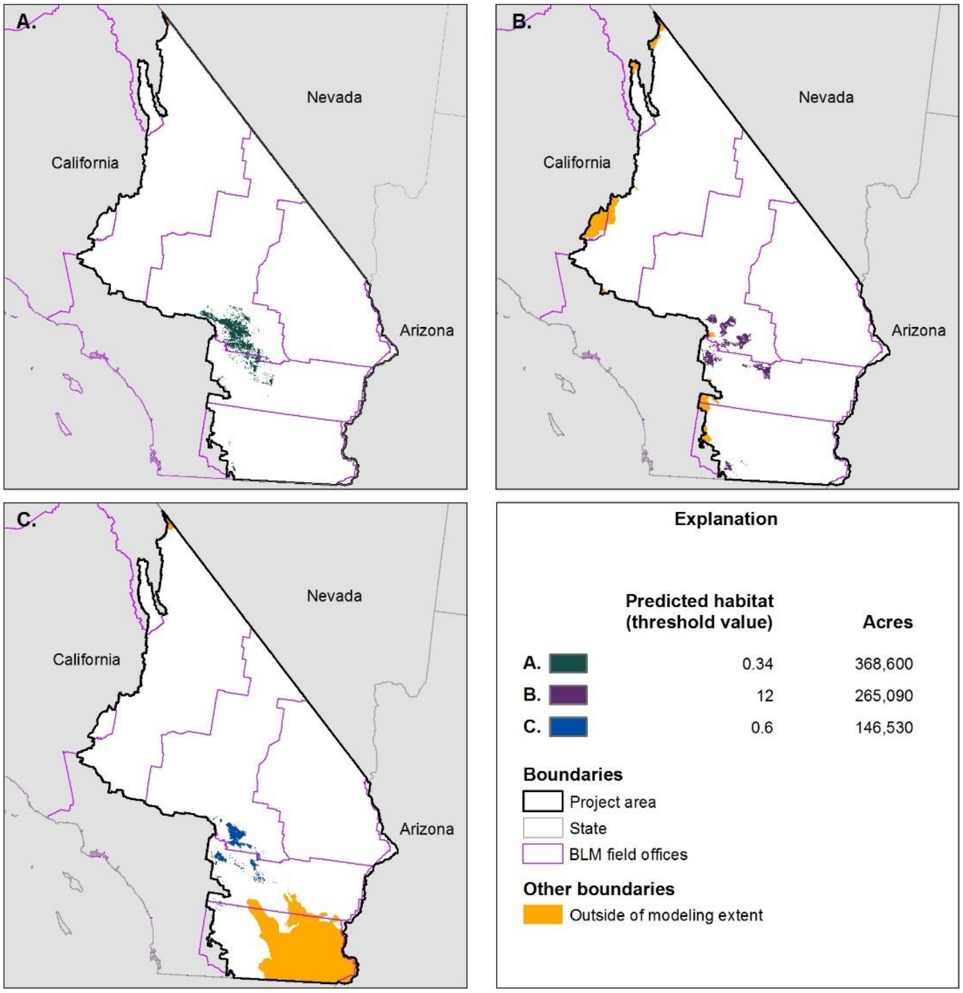

Fig B27_*Linanthus maculatus.*

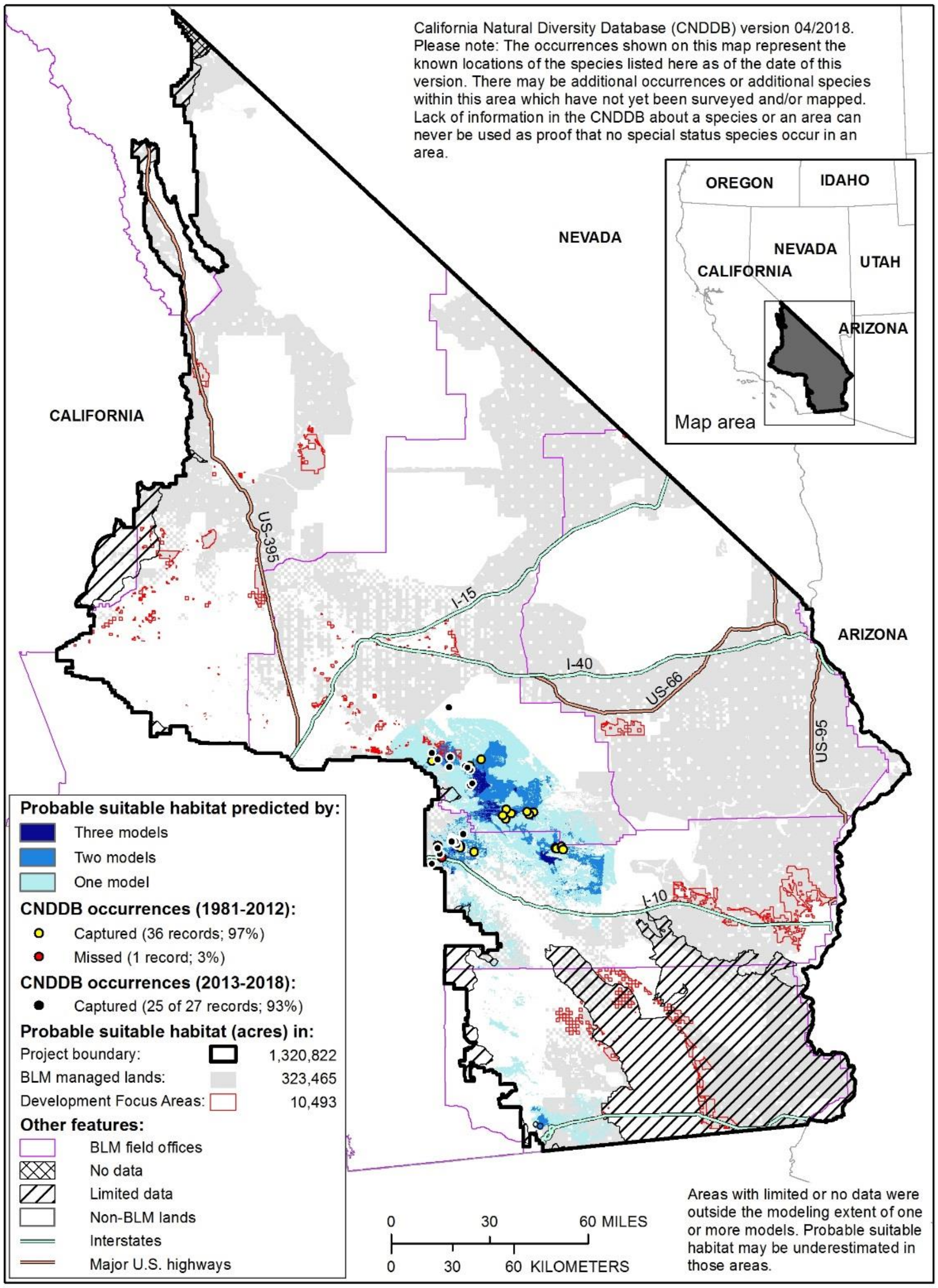

Fig C27_*Linanthus maculatus*.

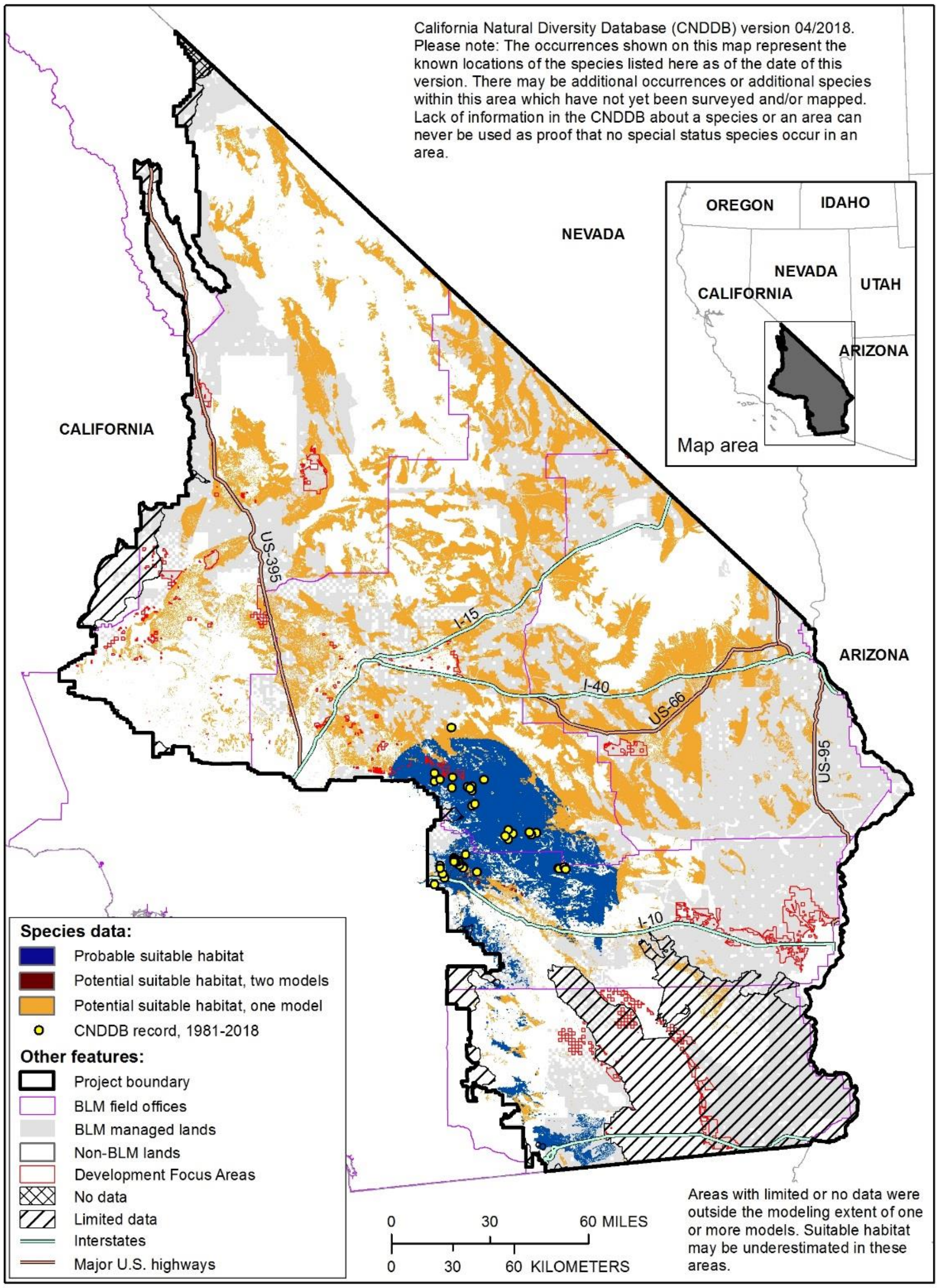

Table B28_*Lupinus excubitus* var. *medius.*

Category	Topic		Contractor B	Contractor C
Occurrence data used to develop the model	Number of occurrences*		Report/data indicate that model was built from 32 occurrences. Currently available CNDDB data indicate 42 occurrences were available for use by this contractor for model development.	Report/data indicate that model was built from 69 occurrences. Currently available CNDDB data indicate 42 occurrences were available for use by this contractor for model development.
	Age of occurrences*		24 of 42 (57%) currently available CNDDB occurrences are from prior to 1981.	24 of 42 (57%) currently available CNDDB occurrences are from prior to 1981.
	Spatial accuracy of occurrences*		32 of 42 (76%) currently available CNDDB occurrences have imprecise spatial accuracy.	32 of 42 (76%) currently available CNDDB occurrences have imprecise spatial accuracy.
	Status of occurrences*		3 of 42 (7%) currently available CNDDB occurrences have Fair or Poor occurrence ranks.	3 of 42 (7%) currently available CNDDB occurrences have Fair or Poor occurrence ranks.
	Species identification of occurrences*			A substantial portion of records appear to be from sources other than CNDDB, for which the reliability of species identification is unknown.

Category	Topic		Contractor B	Contractor C
	Spatial bias of occurrences*			
	Spatial distribution of occurrences*		Currently available CNDDB records in the contractor's boundary are from a limited portion of the area of the occupied geographic subdivisions for the species in California [54].	Currently available CNDDB records in the contractor's boundary are from a limited portion of the area of the occupied geographic subdivisions for the species in California [54].
	Absence data			
Environ-mental covariates	Ecological relevance			
	Comprehensive			
	Resolution and scale			
	Accuracy			
	Number of covariates		Model includes 15 covariates and 32 occurrences.	Model includes 7 covariates and 69 occurrences; report stated that no more than one variable per 10 occurrences was allowed.
	Current covariate data			
	Covariate selection			
	Correlation			
Modeling algorithm	Use in the literature			
	Interactions			

Category	Topic		Contractor B	Contractor C
	Non-linear			
Modeling extent and resolution	Model extent		Contractor's project boundary includes most of the occupied geographic subdivision for the species in California [54].	Contractor's project boundary includes all of the occupied geographic subdivision for the species in California [54], but not a full buffer around it.
	Resolution of model output			
Model selection and thresholds	Model selection			
	Selection of threshold for mapping suitable habitat			

Fig A28_*Lupinus excubitus* var. *medius.*

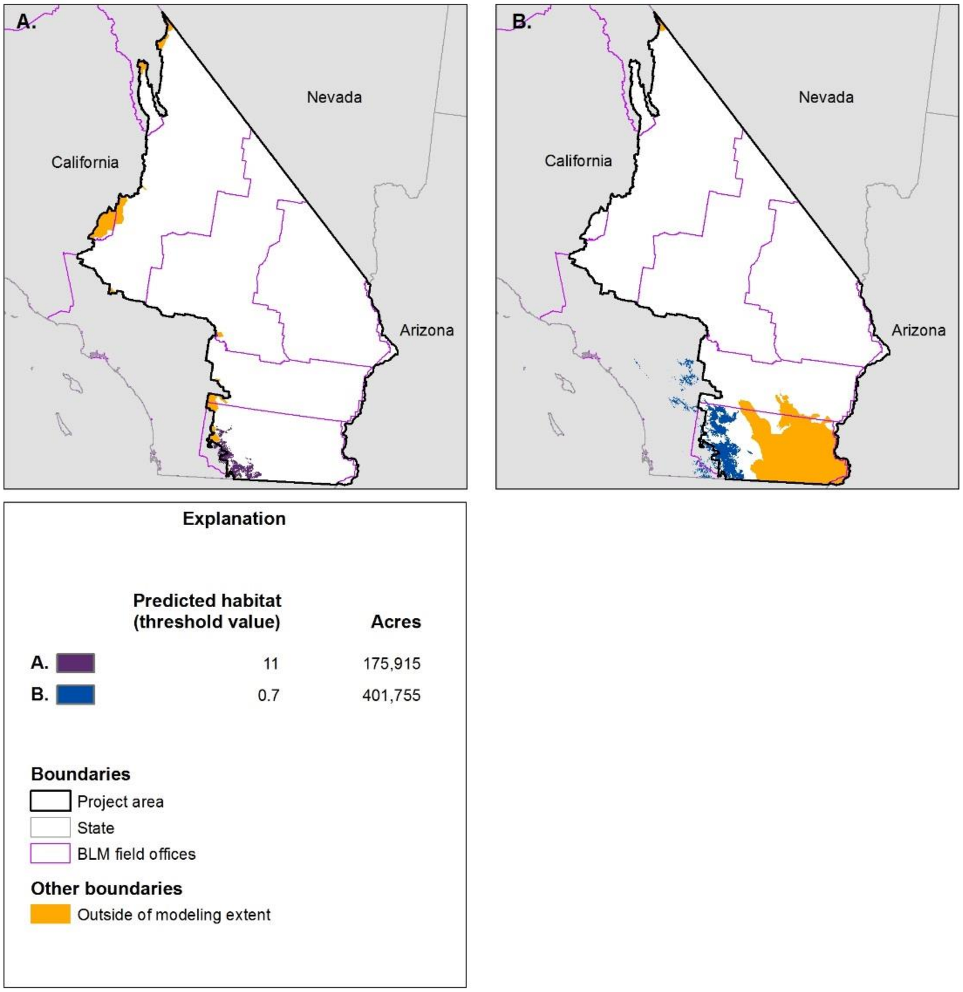

Fig C28_*Lupinus excubitus* var. *medius*.

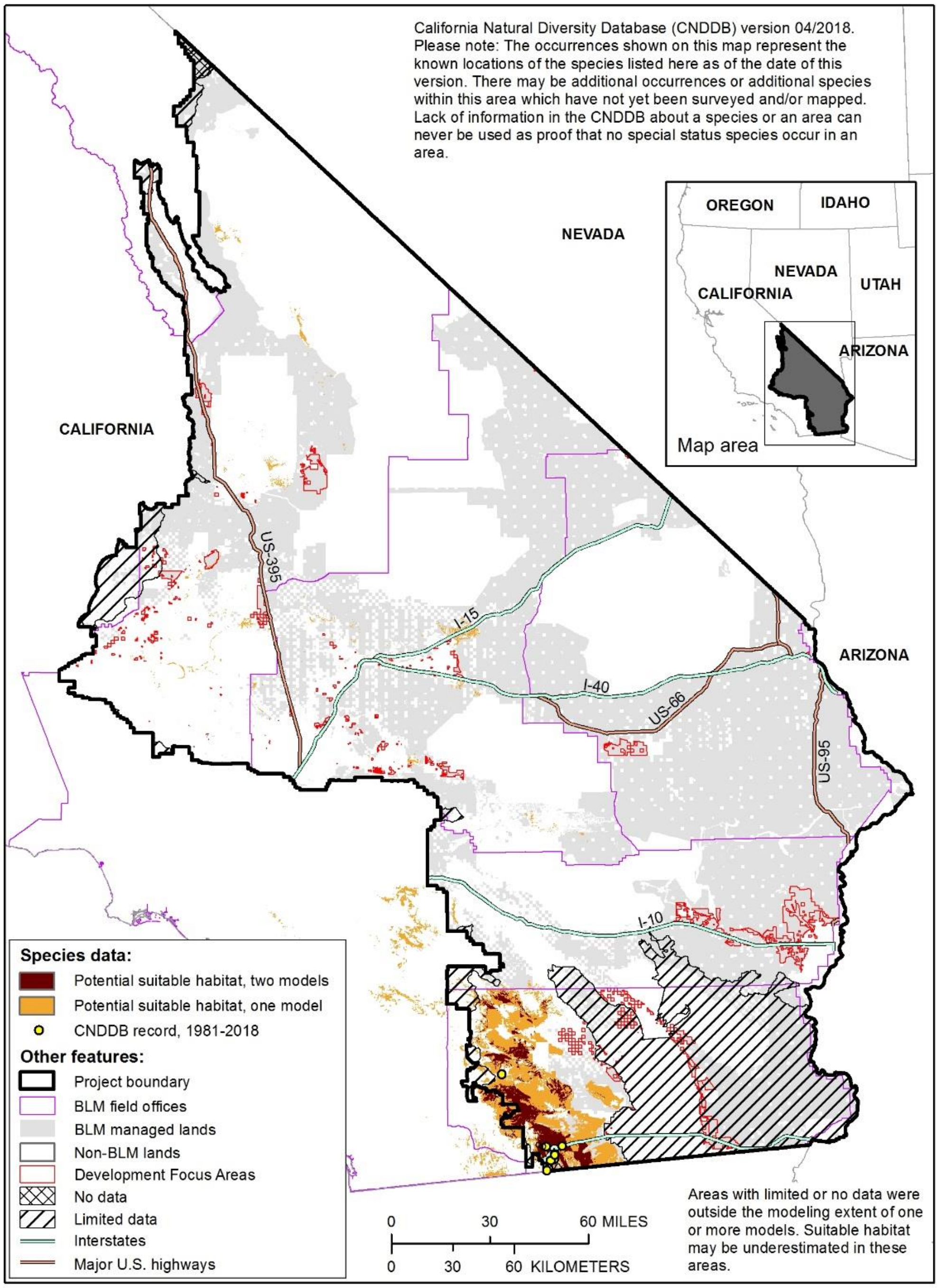

Table B29_*Menodora spinescens* var. *mohavensis.*

Category	Topic		Contractor B	
Occurrence data used to develop the model	Number of occurrences*		Report/data indicate that model was built from 13 occurrences. Currently available CNDDB data indicate 13 occurrences were available for use by this contractor for model development.	
	Age of occurrences*		2 of 13 (15%) currently available CNDDB occurrences are from prior to 1981.	
	Spatial accuracy of occurrences*		6 of 13 (46%) currently available CNDDB occurrences have imprecise spatial accuracy.	
	Status of occurrences*		3 of 13 (23%) currently available CNDDB occurrences have Fair occurrence ranks.	
	Species identification of occurrences*			
	Spatial bias of occurrences*			

Category	Topic		Contractor B	
	Spatial distribution of occurrences*		Currently available CNDDB records in the contractor's boundary are from a substantial portion of the area of the occupied geographic subdivisions for the species in California [54].	
	Absence data			
Environ-mental covariates	Ecological relevance			
	Comprehensive			
	Resolution and scale			
	Accuracy			
	Number of covariates		Model includes 17 covariates and 13 occurrences.	
	Current covariate data			
	Covariate selection			
	Correlation			
Modeling algorithm	Use in the literature			
	Interactions			
	Non-linear			

Category	Topic		Contractor B	
Modeling extent and resolution	Model extent		Contractor's project boundary excludes a substantial portion of the area of the occupied geographic subdivisions for the species in California [54].	
	Resolution of model output			
Model selection and thresholds	Model selection			
	Selection of threshold for mapping suitable habitat			

Fig A29_*Menodora spinescens* var. *mohavensis*.

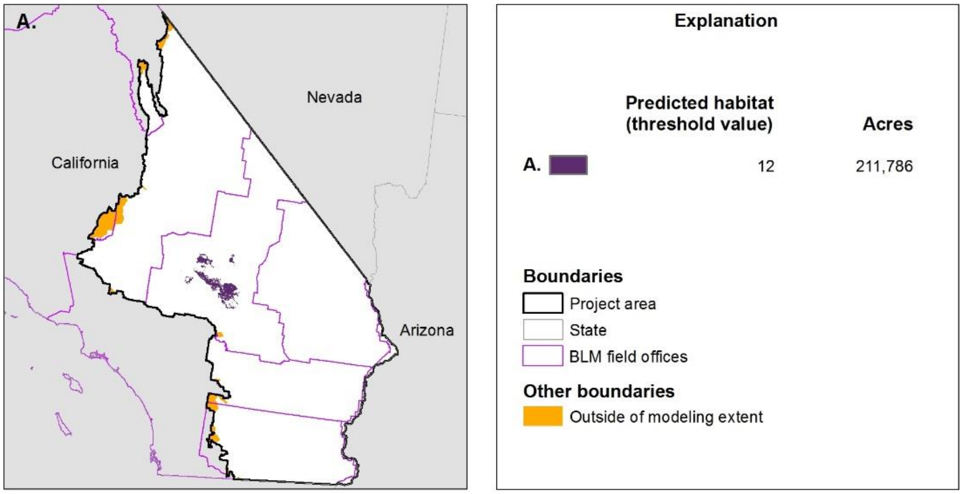

Fig C29_*Menodora spinescens* var. *mohavensis*.

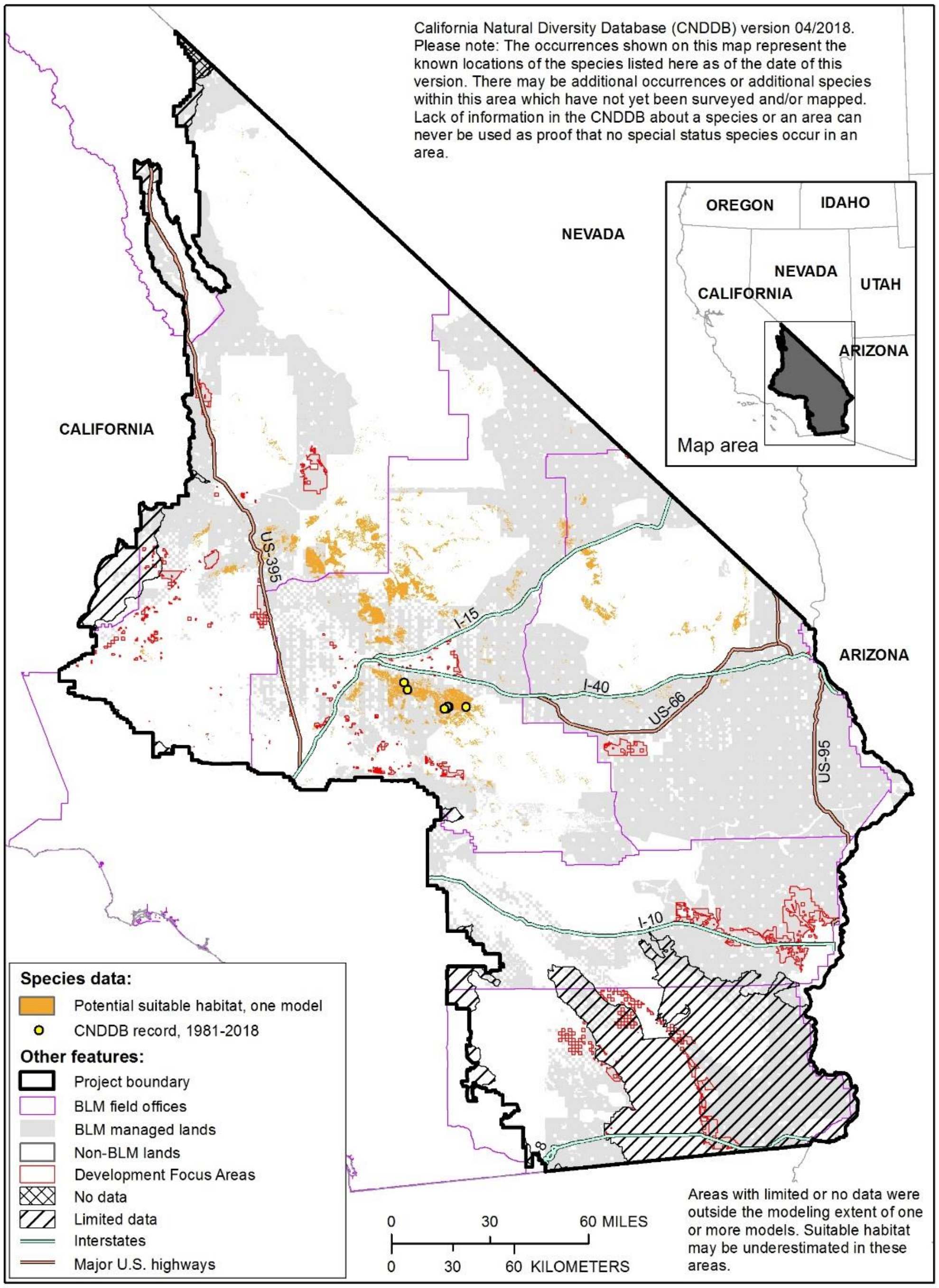

Table B30_*Mentzelia tridentata*.

Category	Topic		Contractor B	
Occurrence data used to develop the model	Number of occurrences*		Report/data indicate that model was built from 25 occurrences. Currently available CNDDB data indicate 28 occurrences were available for use by this contractor for model development.	
	Age of occurrences*		15 of 28 (54%) currently available CNDDB occurrences are from prior to 1981.	
	Spatial accuracy of occurrences*		18 of 28 (64%) currently available CNDDB occurrences have imprecise spatial accuracy.	
	Status of occurrences*		0 of 37 (0%) currently available CNDDB occurrences have Fair or Poor occurrence ranks.	
	Species identification of occurrences*			
	Spatial bias of occurrences*			

Category	Topic		Contractor B	
	Spatial distribution of occurrences*		Currently available CNDDB records in the contractor's boundary are from a substantial portion of the area of the occupied geographic subdivisions for the species in California [54].	
	Absence data			
Environ-mental covariates	Ecological relevance			
	Comprehensive			
	Resolution and scale			
	Accuracy			
	Number of covariates		Model includes 18 covariates and 25 occurrences.	
	Current covariate data			
	Covariate selection			
	Correlation			
Modeling algorithm	Use in the literature			
	Interactions			
	Non-linear			

Category	Topic		Contractor B	
Modeling extent and resolution	Model extent		Contractor's project boundary includes most of the occupied geographic subdivisions for the species in California [54], but not a full buffer around them.	
	Resolution of model output			
Model selection and thresholds	Model selection			
	Selection of threshold for mapping suitable habitat			

Fig A30_*Mentzelia tridentata*.

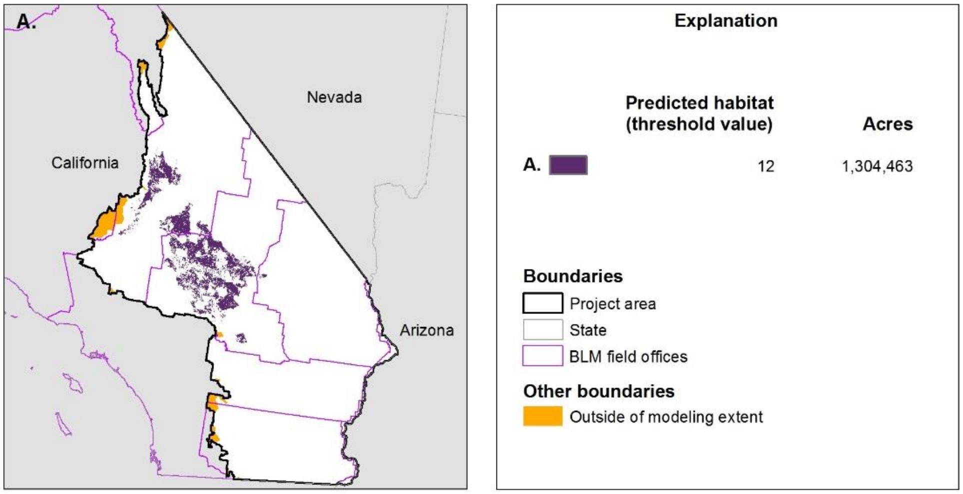

Fig B30_*Mentzelia tridentata*.

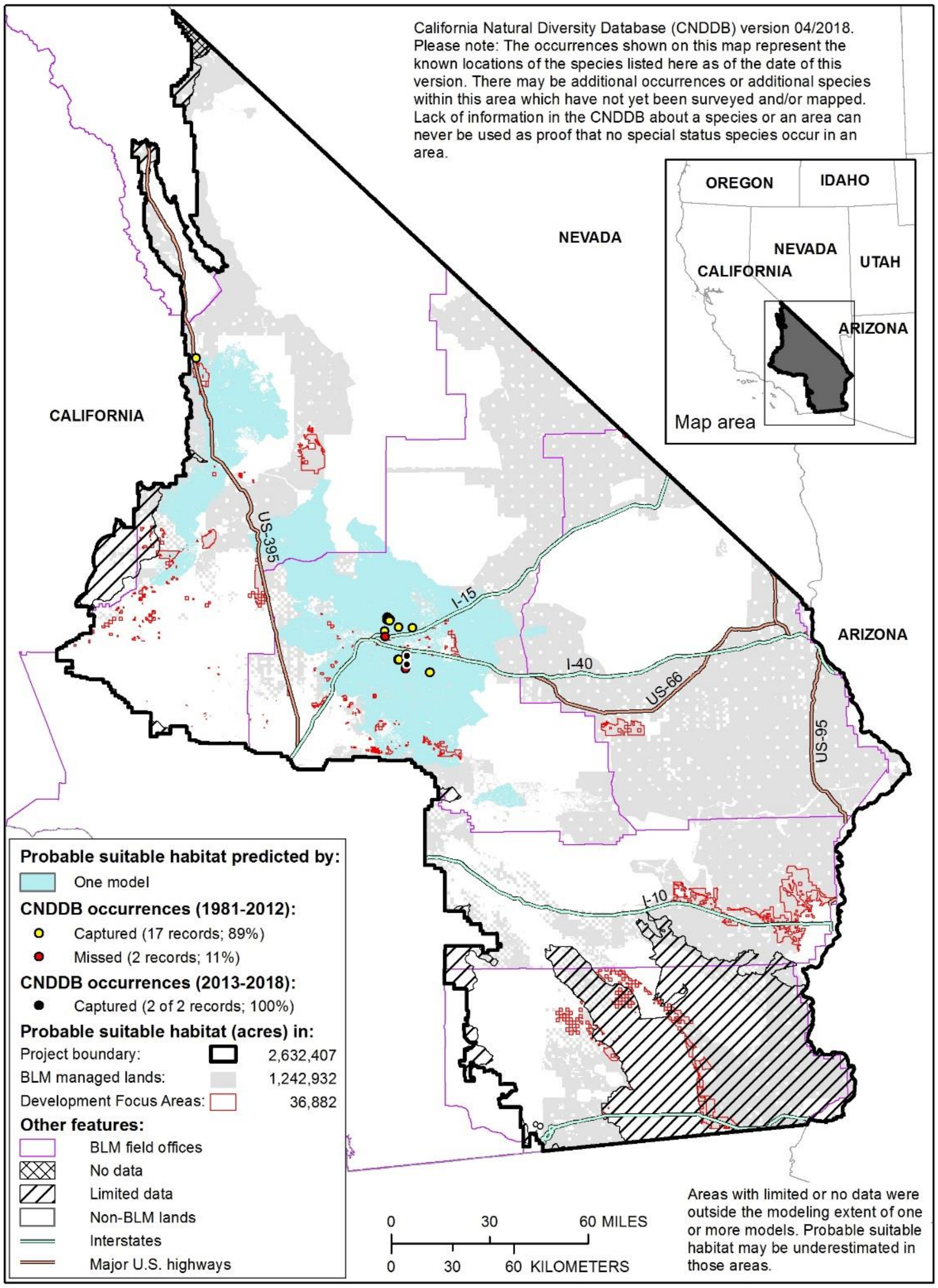

Fig C30_*Mentzelia tridentata*.

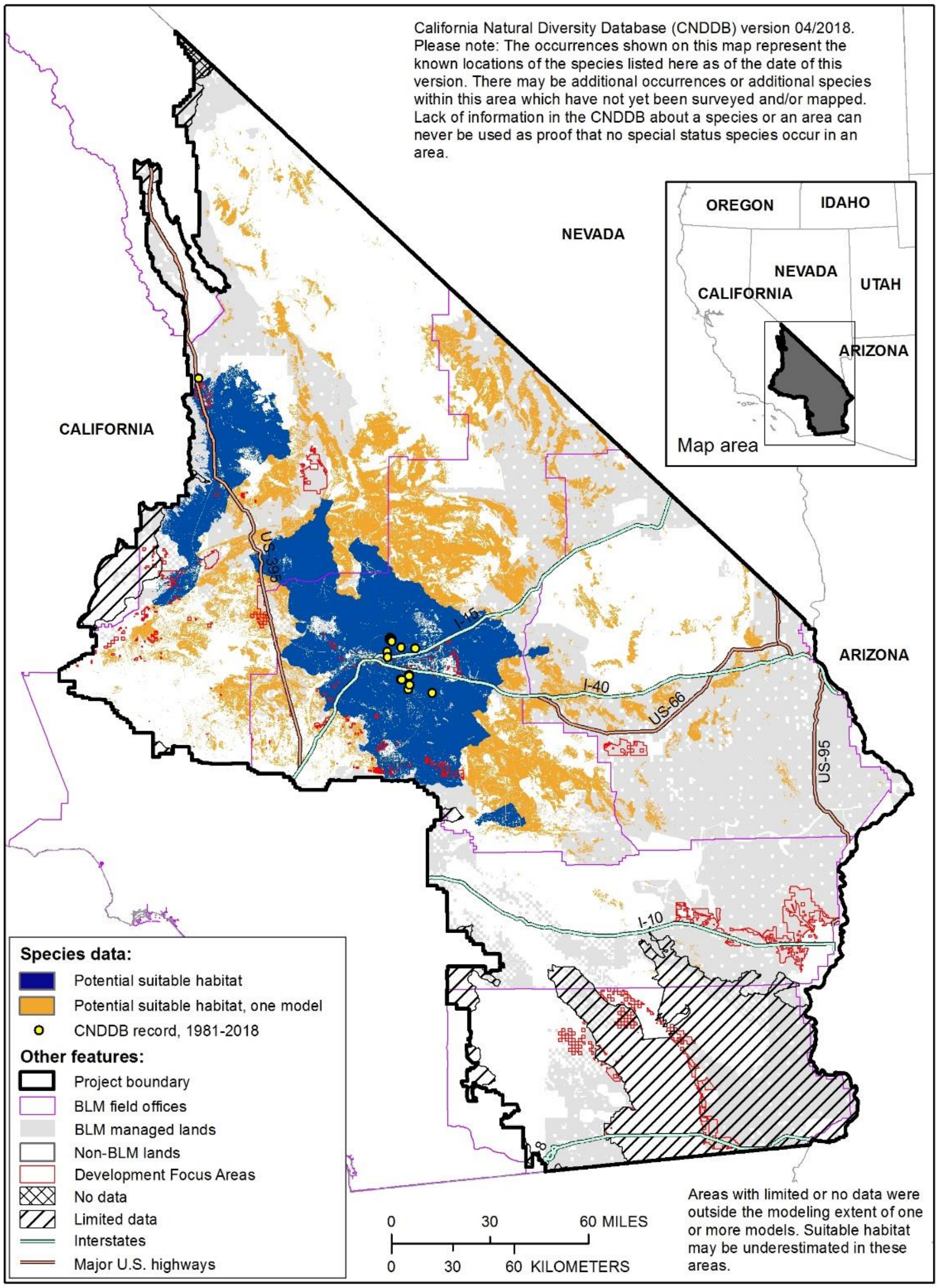

Table B31_*Mimulus mohavensis*.

Category	Topic	Contractor A	Contractor B	Contractor C
Occurrence data used to develop the model	Number of occurrences*	Report/data indicate that model was built from 175 occurrences. Currently available CNDDB data indicate 49 occurrences were likely used by this contractor for model development.	Report/data indicate that model was built from 56 occurrences. Currently available CNDDB data indicate 55 occurrences were available for use by this contractor for model development.	Report/data indicate that model was built from 65 occurrences. Currently available CNDDB data indicate 55 occurrences were available for use by this contractor for model development.
	Age of occurrences*	Report indicates use of occurrence data from 1981-2012. Many records are from prior to 2000.	6 of 55 (11%) currently available CNDDB occurrences are from prior to 1981.	6 of 55 (11%) currently available CNDDB occurrences are from prior to 1981.
	Spatial accuracy of occurrences*	Report/data indicate occurrences with uncertainty >250-500 m were excluded.	10 of 55 (18%) currently available CNDDB occurrences have imprecise spatial accuracy.	10 of 55 (18%) currently available CNDDB occurrences have imprecise spatial accuracy.
	Status of occurrences*	13 of 49 (26%) currently available CNDDB occurrences have Fair or Poor occurrence ranks.	13 of 55 (24%) currently available CNDDB occurrences have Fair or Poor occurrence ranks.	13 of 55 (24%) currently available CNDDB occurrences have Fair or Poor occurrence ranks.
	Species identification of occurrences*			Most records appear to be from CNDDB, for which species identification is reliable.
	Spatial bias of occurrences*			

Category	Topic	Contractor A	Contractor B	Contractor C
	Spatial distribution of occurrences*	Currently available CNDDB records in the contractor's boundary are from a limited portion of the area of the occupied geographic subdivision for the species in California [54].	Currently available CNDDB records in the contractor's boundary are from a limited portion of the area of the occupied geographic subdivision for the species in California [54].	Currently available CNDDB records in the contractor's boundary are from a limited portion of the area of the occupied geographic subdivision for the species in California [54].
	Absence data			
Environ-mental covariates	Ecological relevance			
	Comprehensive			
	Resolution and scale			
	Accuracy			
	Number of covariates	Model includes no more than 22 covariates (number is unclear from data provided) and 175 occurrences.	Model includes 17 covariates and 56 occurrences.	Model includes 7 covariates and 65 occurrences; report stated that no more than one variable per 10 occurrences was allowed.
	Current covariate data			
	Covariate selection			
	Correlation			
Modeling algorithm	Use in the literature			
	Interactions			
	Non-linear			

Category	Topic	Contractor A	Contractor B	Contractor C
Modeling extent and resolution	Model extent	Contractor's project boundary includes most or all of the occupied geographic subdivision for the species in California [54], but not a full buffer around it.	Contractor's project boundary includes most of the occupied geographic subdivision for the species in California [54], but not a full buffer around it.	Contractor's project boundary includes most or all of the occupied geographic subdivision for the species in California [54], but not a full buffer around it.
	Resolution of model output			
Model selection and thresholds	Model selection			
	Selection of threshold for mapping suitable habitat			

Fig A31_*Mimulus mohavensis*.

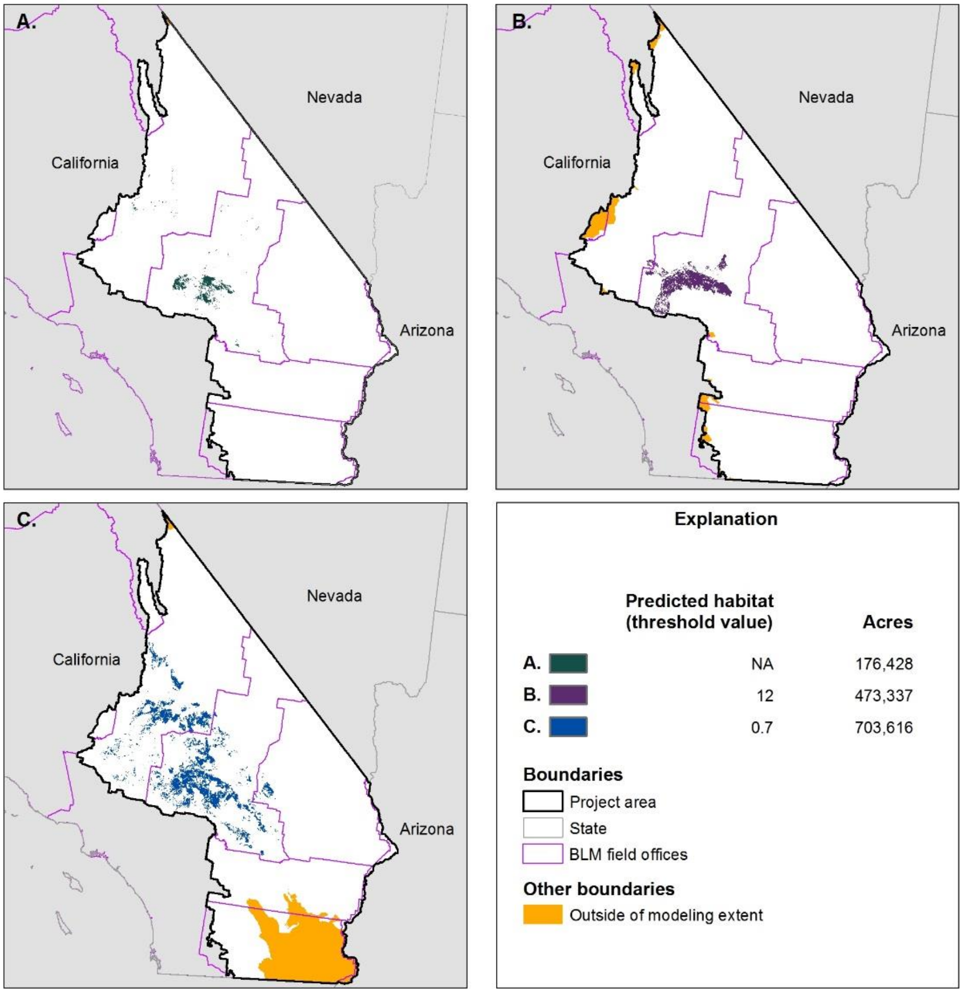

Fig B31_*Mimulus mohavensis*.

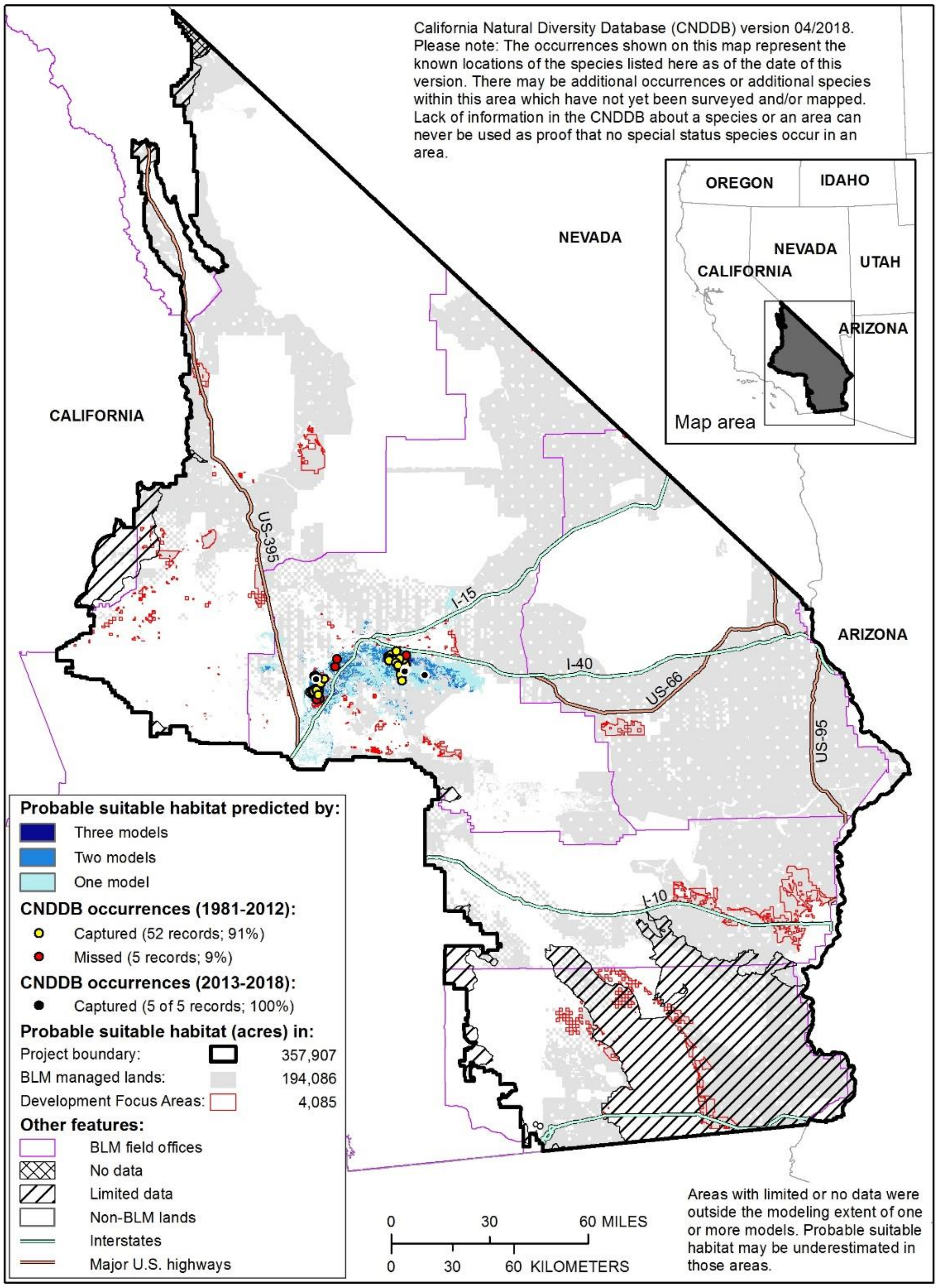

Fig C31_*Mimulus mohavensis*.

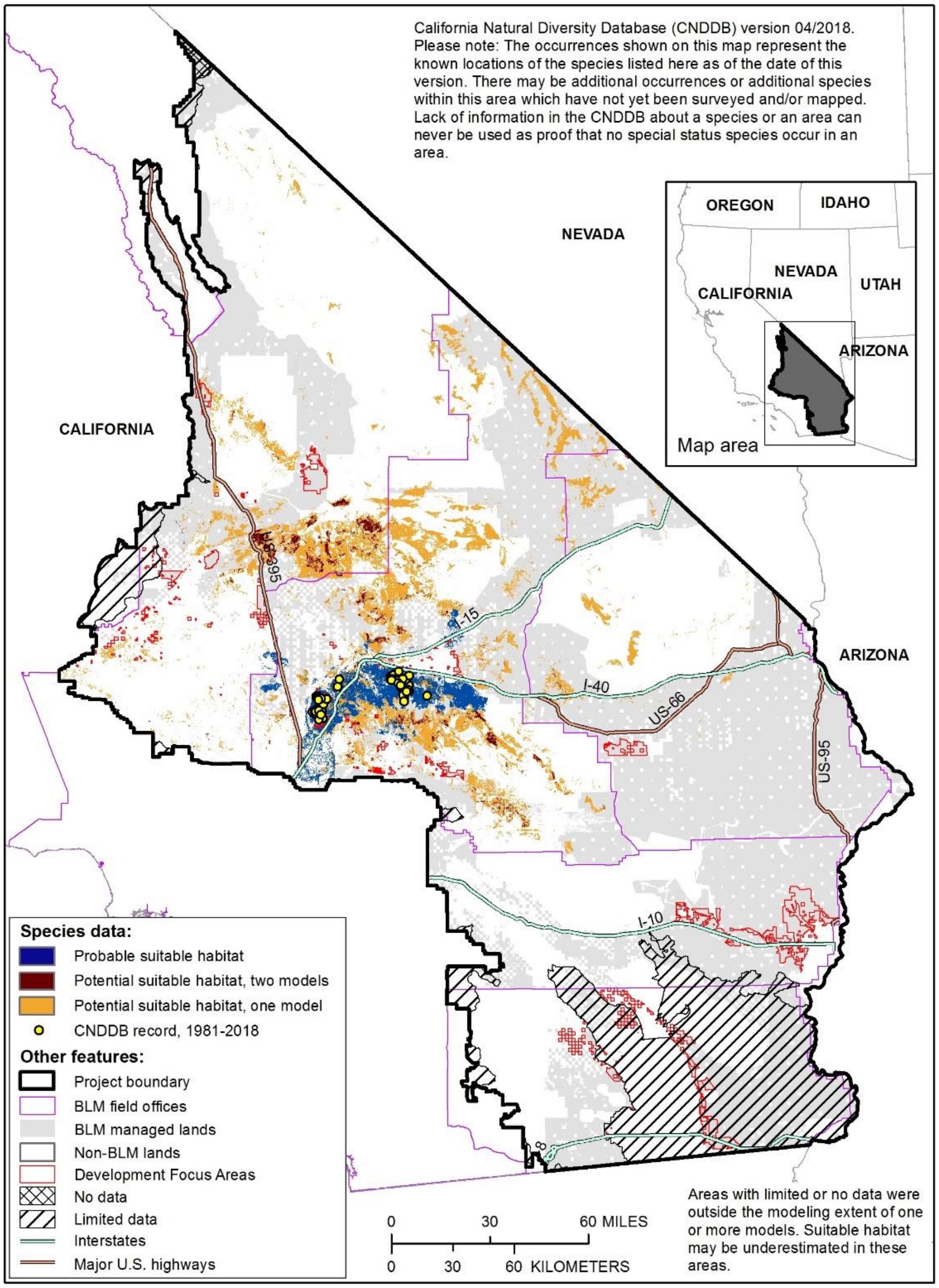

Table B32_*Monardella linoides* ssp. *oblonga*.

Category	Topic		Contractor B	Contractor C
Occurrence data used to develop the model	Number of occurrences*		Report/data indicate that model was built from 14 occurrences. Currently available CNDDB data indicate 57 occurrences were available for use by this contractor for model development, but most locations were outside the contractor's project boundary.	Report/data indicate that model was built from 63 occurrences. Currently available CNDDB data indicate 57 occurrences were available for use by this contractor for model development.
	Age of occurrences*		10 of 57 (18%) currently available CNDDB occurrences are from prior to 1981.	10 of 57 (18%) currently available CNDDB occurrences are from prior to 1981.
	Spatial accuracy of occurrences*		2 of 57 (4%) currently available CNDDB occurrences have imprecise spatial accuracy.	2 of 57 (4%) currently available CNDDB occurrences have imprecise spatial accuracy.
	Status of occurrences*		14 of 57 (24%) currently available CNDDB occurrences have Fair or Poor occurrence ranks.	14 of 57 (24%) currently available CNDDB occurrences have Fair or Poor occurrence ranks.
	Species identification of occurrences*			Most records appear to be from CNDDB, for which species identification is reliable.

Category	Topic		Contractor B	Contractor C
	Spatial bias of occurrences*			
	Spatial distribution of occurrences*		Currently available CNDDB records in the contractor’s boundary are from a limited portion of the area of the occupied geographic subdivision for the species in California [54].	Currently available CNDDB records in the contractor’s boundary are from a substantial portion of the area of the occupied geographic subdivision for the species in California [54].
	Absence data			
Environ-mental covariates	Ecological relevance			
	Comprehensive			
	Resolution and scale			
	Accuracy			
	Number of covariates		Model includes 13 covariates and 14 occurrences.	Model includes 7 covariates and 63 occurrences; report stated that no more than one variable per 10 occurrences was allowed.
	Current covariate data			
	Covariate selection			
	Correlation			
Modeling algorithm	Use in the literature			
	Interactions			

Category	Topic		Contractor B	Contractor C
	Non-linear			
Modeling extent and resolution	Model extent		Contractor’s project boundary excludes a significant portion of the occupied geographic subdivisions for the species in California [54].	Contractor’s project boundary includes most of the occupied geographic subdivisions for the species in California [54], but not a full buffer around it.
	Resolution of model output			
Model selection and thresholds	Model selection			
	Selection of threshold for mapping suitable habitat			

Fig A32_*Monardella linoides* ssp. *oblonga*.

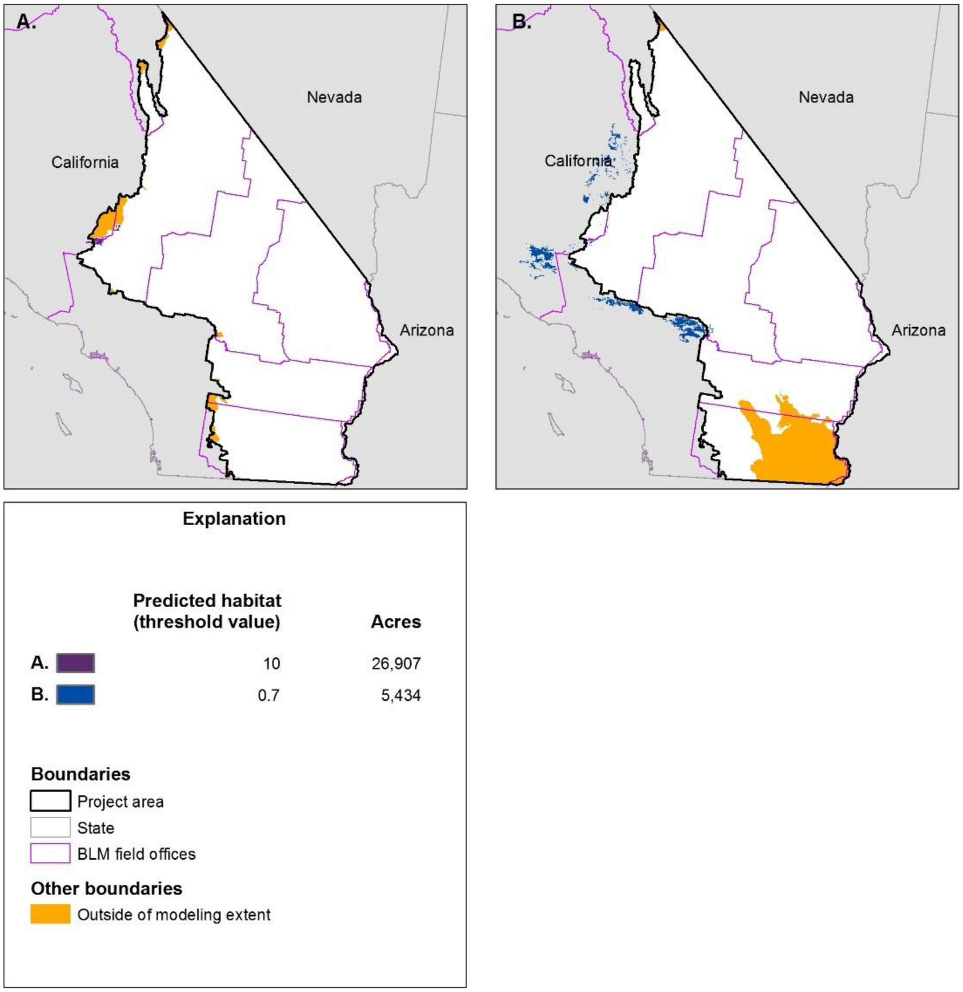

Fig B32_*Monardella linoides* ssp. *oblonga*.

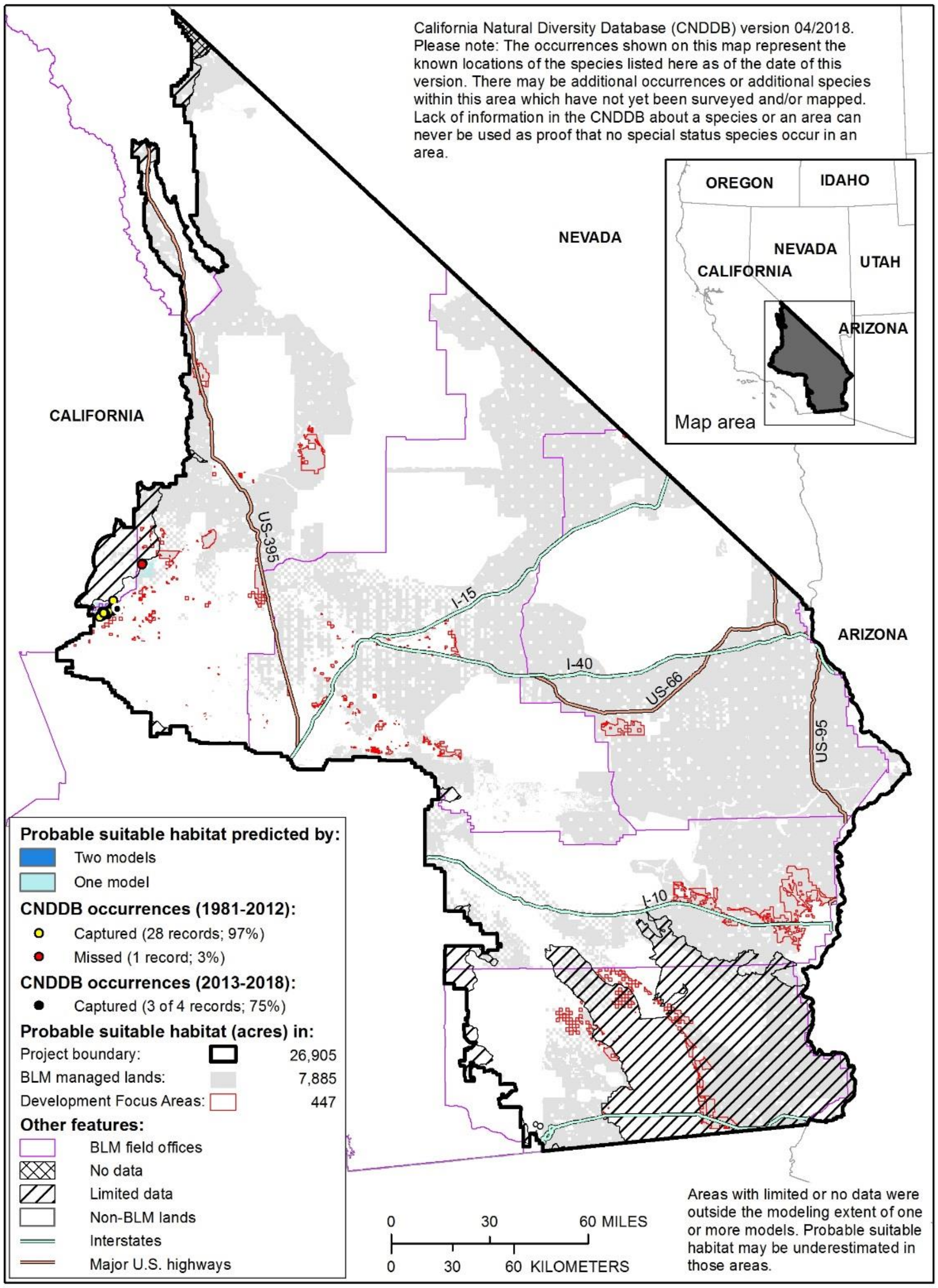

Fig C32_*Monardella linoides* ssp. *oblonga*.

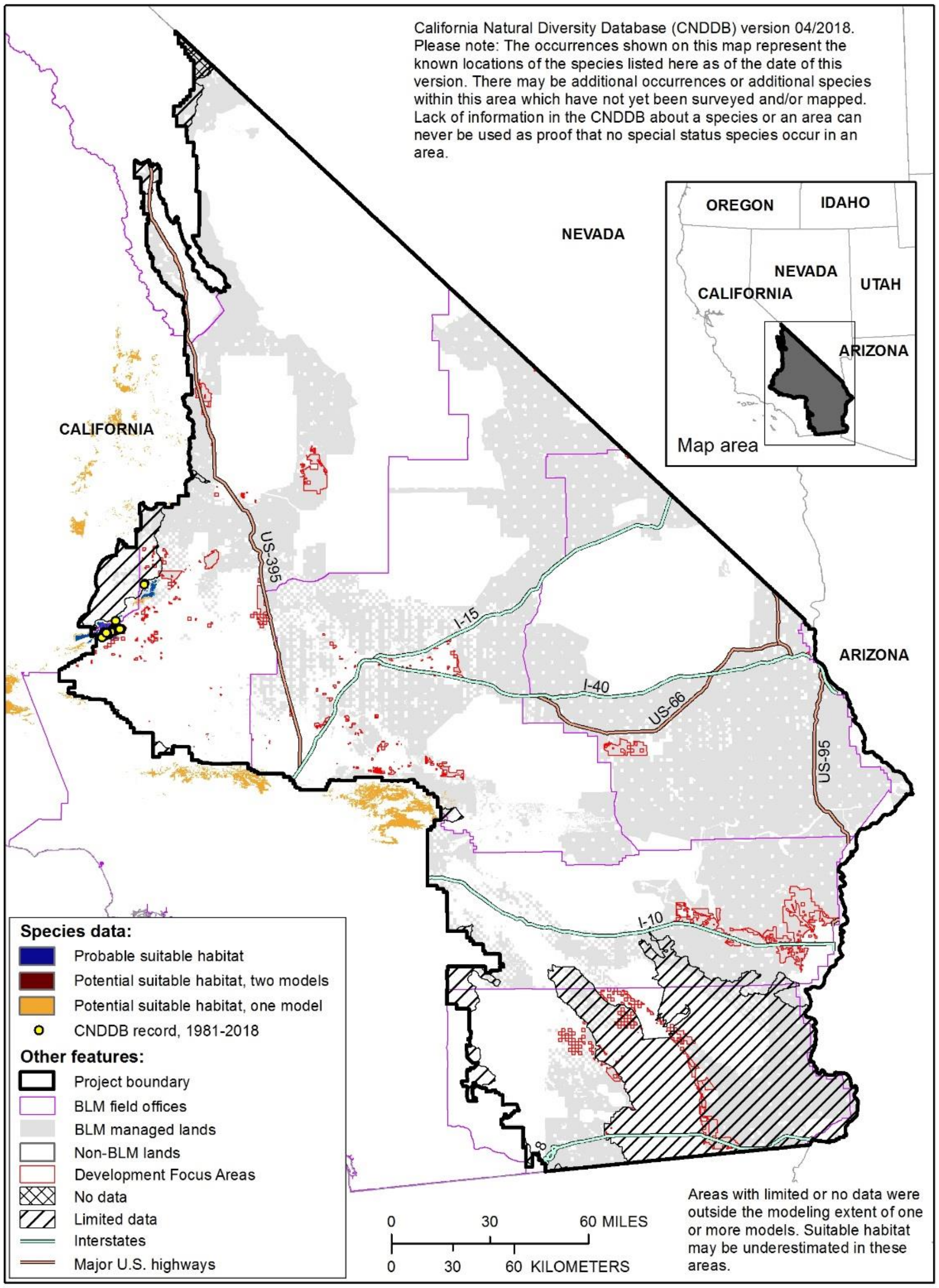

Table B33_*Nitrophila mohavensis.*

Category	Topic		Contractor B	
Occurrence data used to develop the model	Number of occurrences*		Report/data indicate that model was built from 2 occurrences. Currently available CNDDB data indicate 2 occurrences were available for use by this contractor for model development.	
	Age of occurrences*		0 of 2 (15%) currently available CNDDB occurrences are from prior to 1981. Both occurrences are more recent than 2000.	
	Spatial accuracy of occurrences*		1 of 2 (50%) currently available CNDDB occurrences have imprecise spatial accuracy.	
	Status of occurrences*		1 of 2 (50%) currently available CNDDB occurrences has a Fair occurrence rank.	
	Species identification of occurrences*			
	Spatial bias of occurrences*			

Category	Topic		Contractor B	
	Spatial distribution of occurrences*		Currently available CNDDB records in the contractor’s boundary are from a limited portion of the area of the occupied geographic subdivisions for the species in California [54].	
	Absence data			
Environ-mental covariates	Ecological relevance			
	Comprehensive			
	Resolution and scale			
	Accuracy			
	Number of covariates		Model includes 14 covariates and 2 occurrences.	
	Current covariate data			
	Covariate selection			
	Correlation			
Modeling algorithm	Use in the literature			
	Interactions			
	Non-linear			

Category	Topic		Contractor B	
Modeling extent and resolution	Model extent		Contractor’s project boundary includes most of the area of the occupied geographic subdivisions for the species in California [54], but not a complete boundary around them.	
	Resolution of model output			
Model selection and thresholds	Model selection			
	Selection of threshold for mapping suitable habitat			

Fig A33_*Nitrophila mohavensis*.

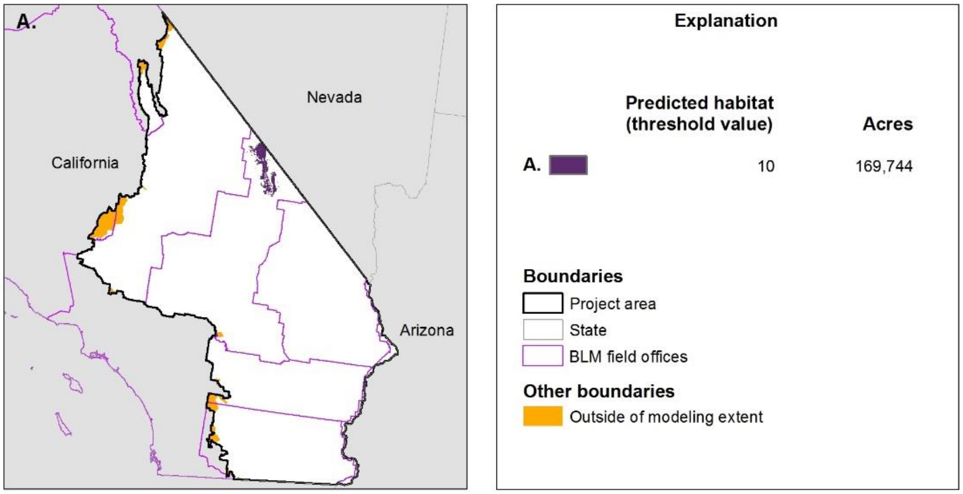

Fig C33_*Nitrophila mohavensis*.

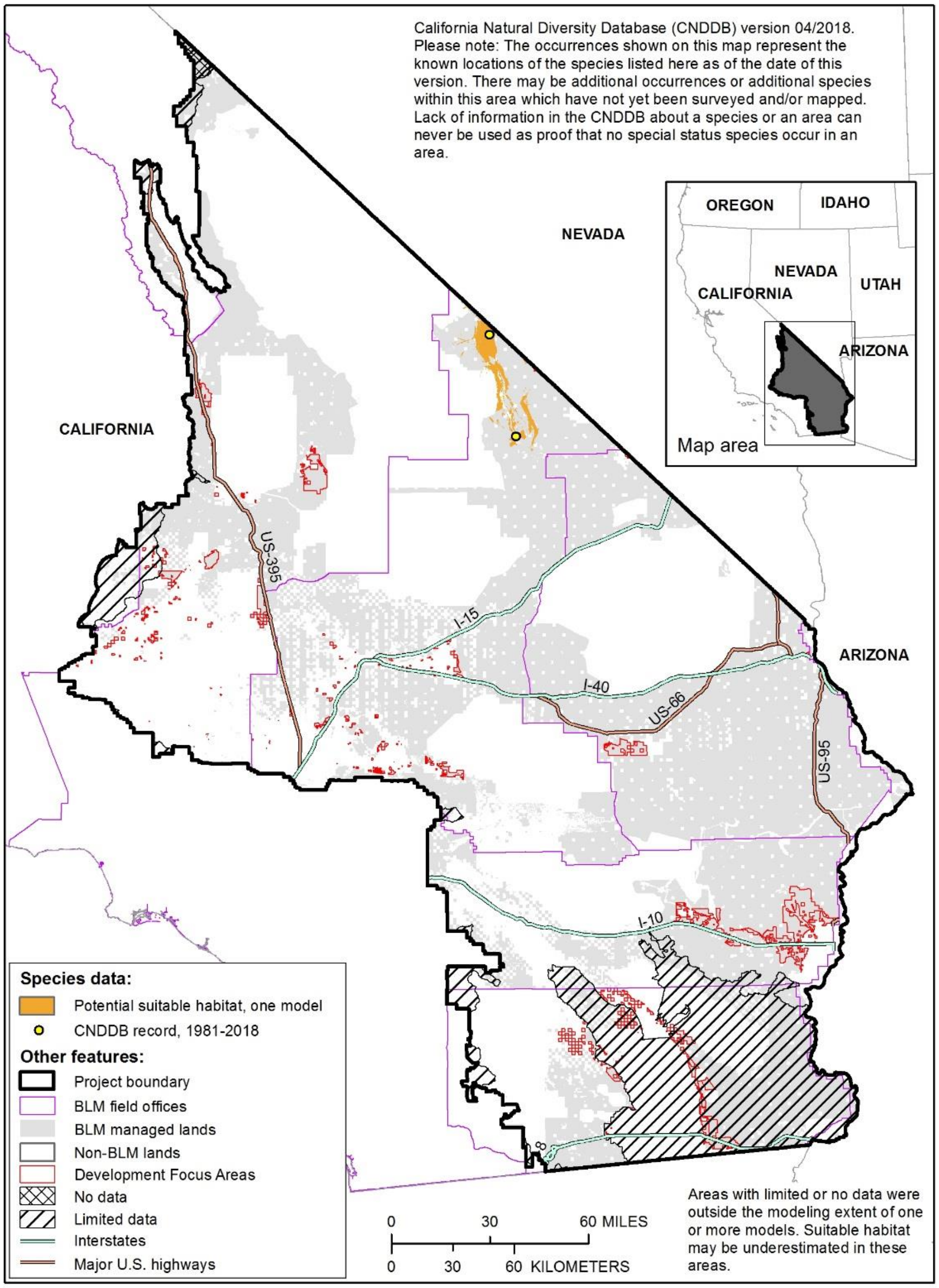

Table B34_*Pediomelum castoreum.*

Category	Topic		Contractor B	
Occurrence data used to develop the model	Number of occurrences*		Report/data indicate that model was built from 22 occurrences. Currently available CNDDB data indicate 20 occurrences were available for use by this contractor for model development.	
	Age of occurrences*		9 of 20 (45%) currently available CNDDB occurrences are from prior to 1981.	
	Spatial accuracy of occurrences*		18 of 20 (90%) currently available CNDDB occurrences have imprecise spatial accuracy.	
	Status of occurrences*		3 of 20 (15%) currently available CNDDB occurrences have Poor occurrence ranks.	
	Species identification of occurrences*			
	Spatial bias of occurrences*			

Category	Topic		Contractor B	
	Spatial distribution of occurrences*		Currently available CNDDB records in the contractor's boundary are from a substantial portion of the area of the occupied geographic subdivision for the species in California [54].	
	Absence data			
Environ-mental covariates	Ecological relevance			
	Comprehensive			
	Resolution and scale			
	Accuracy			
	Number of covariates		Model includes 17 covariates and 22 occurrences.	
	Current covariate data			
	Covariate selection			
	Correlation			
Modeling algorithm	Use in the literature			
	Interactions			
	Non-linear			

Category	Topic		Contractor B	
Modeling extent and resolution	Model extent		Contractor's project boundary includes most of the area of the occupied geographic subdivisions for the species in California [54], but not a complete buffer around them.	
	Resolution of model output			
Model selection and thresholds	Model selection			
	Selection of threshold for mapping suitable habitat			

Fig A34_*Pediomelum castoreum.*

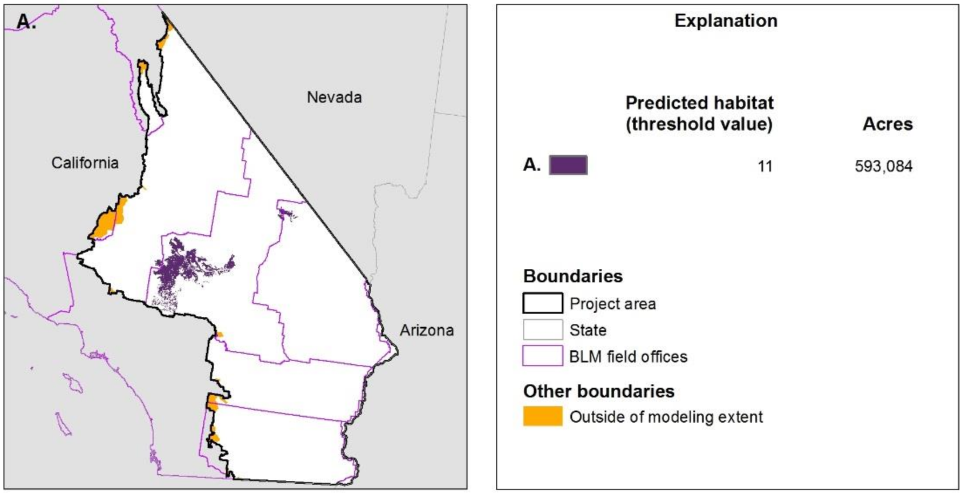

Fig B34_*Pediomelum castoreum.*

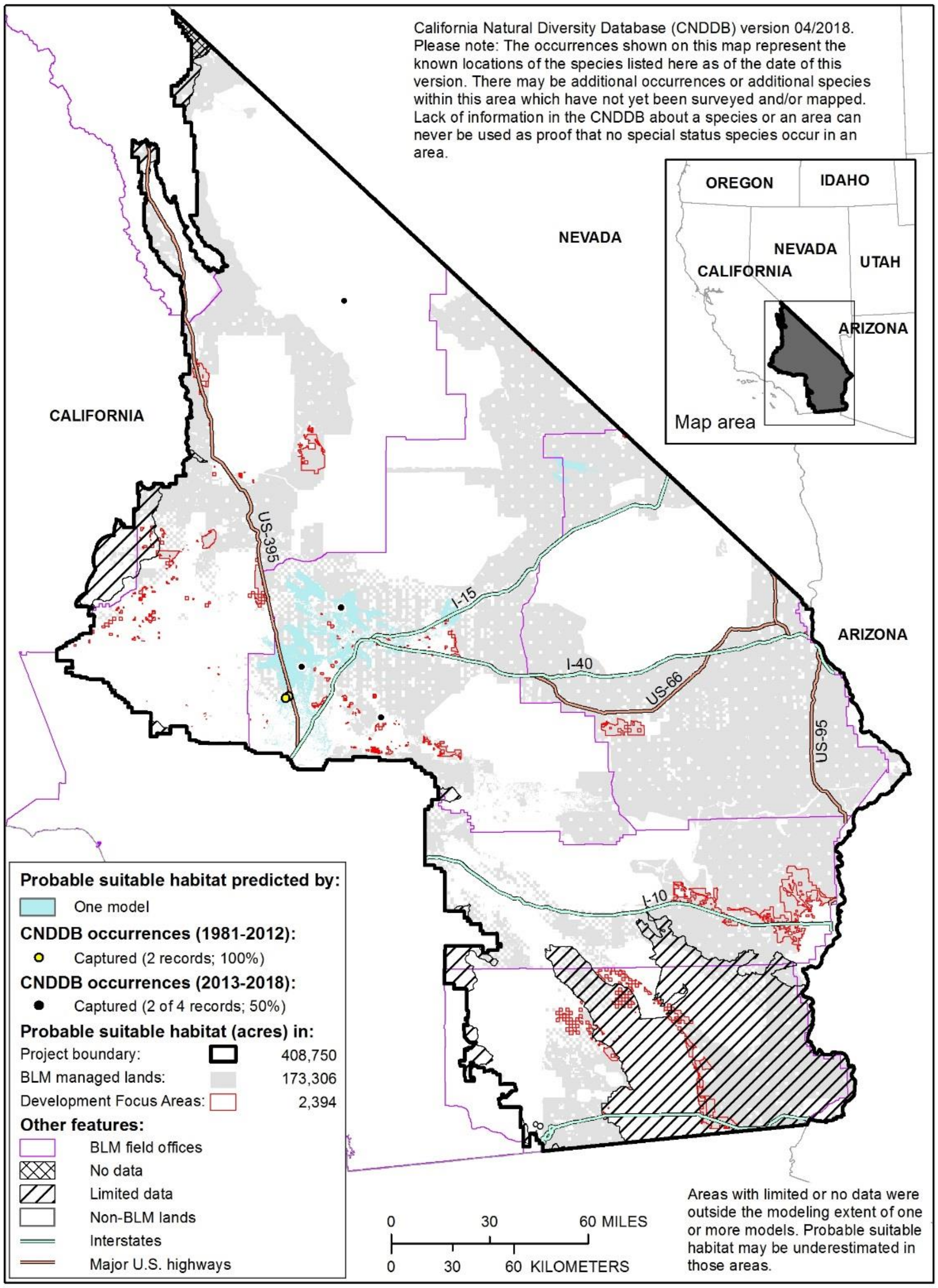

Fig C34_*Pediomelum castoreum.*

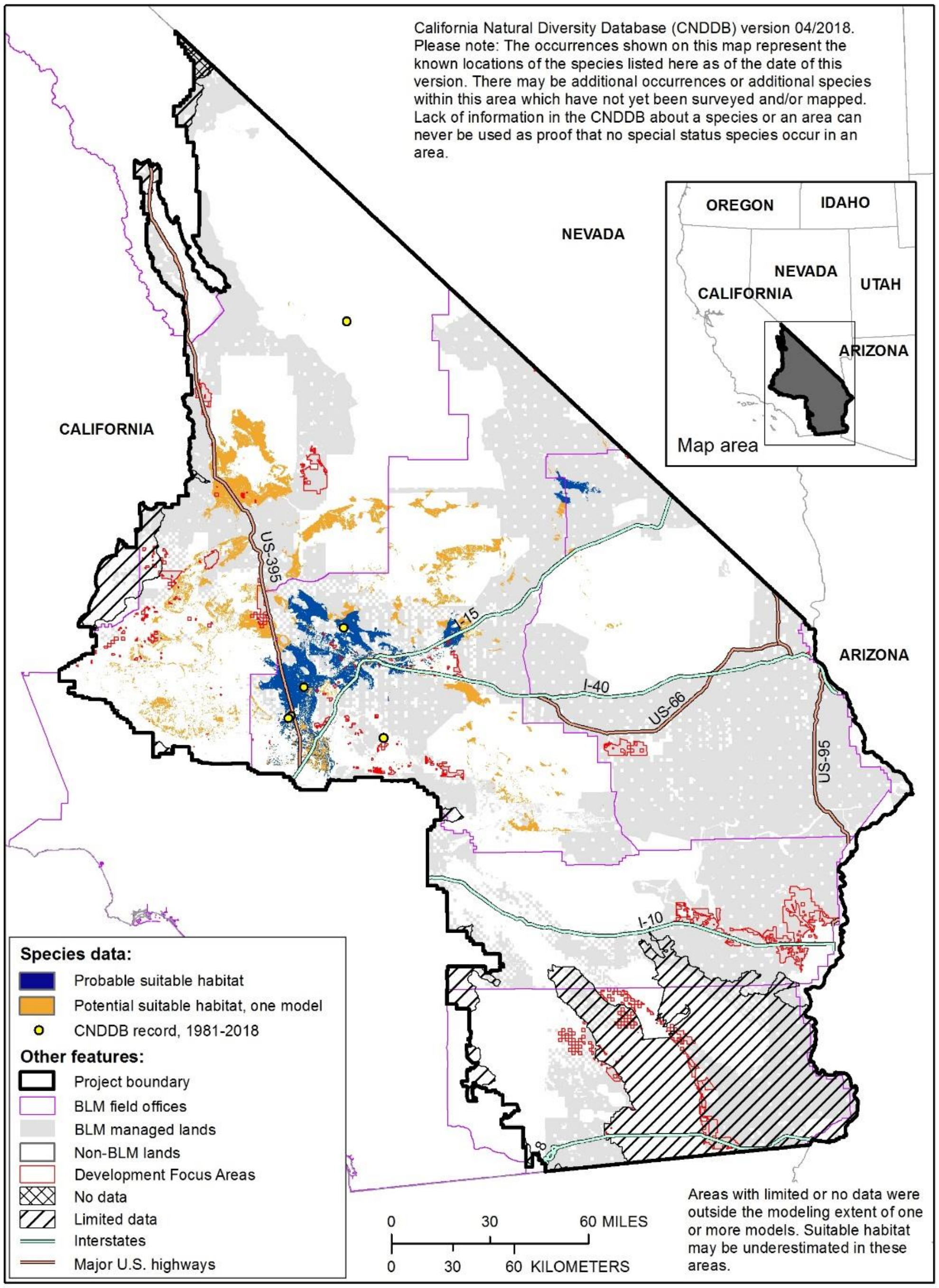

Table B35_*Penstemon albomarginatus.*

Category	Topic	Contractor A	Contractor B	Contractor C
Occurrence data used to develop the model	Number of occurrences*	Report/data indicate that model was built from 175 occurrences. Currently available CNDDB data indicate 20 occurrences were likely used by this contractor for model development.	Report/data indicate that model was built from 56 occurrences. Currently available CNDDB data indicate 23 occurrences were available for use by this contractor for model development.	Report/data indicate that model was built from 65 occurrences. Currently available CNDDB data indicate 23 occurrences were available for use by this contractor for model development.
	Age of occurrences*	Report indicates use of occurrence data from 1981-2012. 1 record is from prior to 2000.	3 of 23 (13%) currently available CNDDB occurrences are from prior to 1981.	3 of 23 (13%) currently available CNDDB occurrences are from prior to 1981.
	Spatial accuracy of occurrences*	Report/data indicate occurrences with uncertainty >250-500 m were excluded.	4 of 23 (17%) currently available CNDDB occurrences have imprecise spatial accuracy.	4 of 23 (17%) currently available CNDDB occurrences have imprecise spatial accuracy.
	Status of occurrences*	14 of 20 (70%) currently available CNDDB occurrences have Fair or Poor occurrence ranks.	14 of 23 (61%) currently available CNDDB occurrences have Fair or Poor occurrence ranks.	14 of 23 (61%) currently available CNDDB occurrences have Fair or Poor occurrence ranks.
	Species identification of occurrences*			A substantial portion of records appear to be from a source other than CNDDB, for which the reliability of species identification is unknown.

Category	Topic	Contractor A	Contractor B	Contractor C
	Spatial bias of occurrences*			
	Spatial distribution of occurrences*	Currently available CNDDB records in the contractor's boundary are from a limited portion of the area of the occupied geographic subdivisions for the species in California [54].	Currently available CNDDB records in the contractor's boundary are from a limited portion of the area of the occupied geographic subdivisions for the species in California [54].	Currently available CNDDB records in the contractor's boundary are from a limited portion of the area of the occupied geographic subdivisions for the species in California [54].
	Absence data			
Environ-mental covariates	Ecological relevance			
	Comprehensive			
	Resolution and scale			
	Accuracy			
	Number of covariates	Model includes 12 covariates and 73 occurrences.	Model includes 14 covariates and 23 occurrences.	Models include 5 and 5 covariates and, 36 and 58 occurrences, respectively; report stated that no more than one variable per 10 occurrences was allowed.
	Current covariate data			
	Covariate selection			
	Correlation			
Modeling algorithm	Use in the literature			

Category	Topic	Contractor A	Contractor B	Contractor C
	Interactions			
	Non-linear			
Modeling extent and resolution	Model extent	Contractor’s project boundary includes most or all of the occupied geographic subdivision for the species in California [54], but not a full buffer around it.	Contractor’s project boundary includes most of the occupied geographic subdivision for the species in California [54], but not a full buffer around it.	Contractor’s project boundary includes most or all of the occupied geographic subdivision for the species in California [54], but not a full buffer around it.
	Resolution of model output			
Model selection and thresholds	Model selection			
	Selection of threshold for mapping suitable habitat			

Fig A35_*Penstemon albomarginatus*.

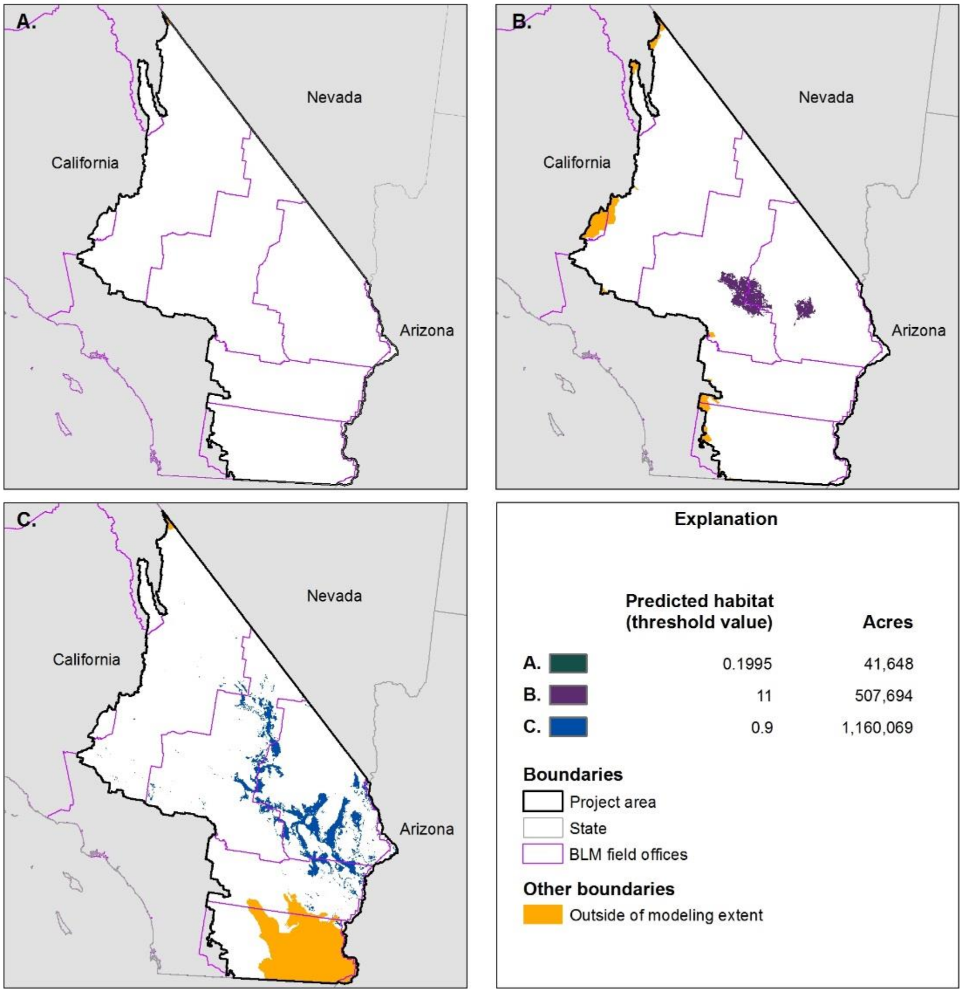

Fig B35_*Penstemon albomarginatus*.

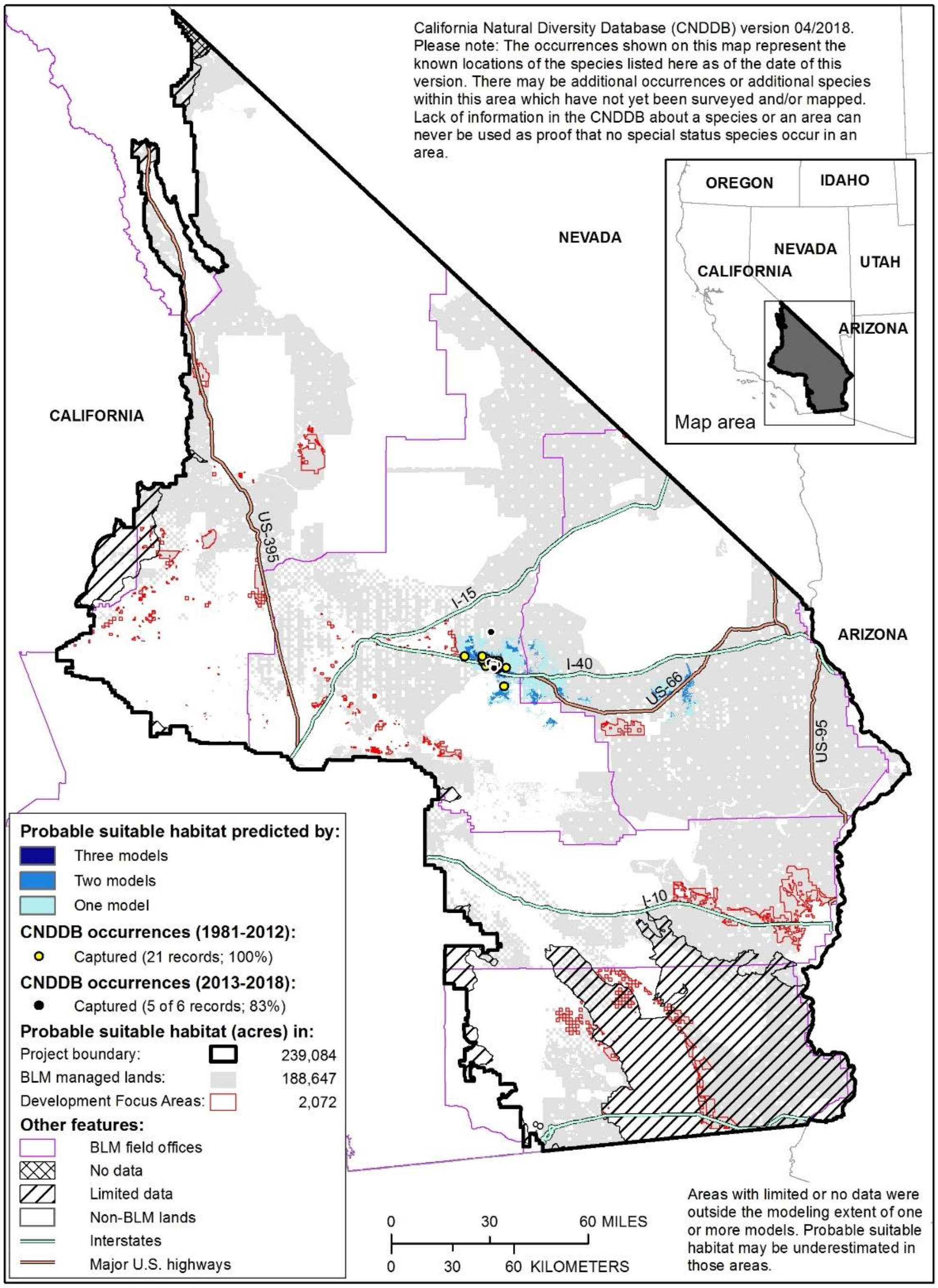

Fig C35_*Penstemon albomarginatus.*

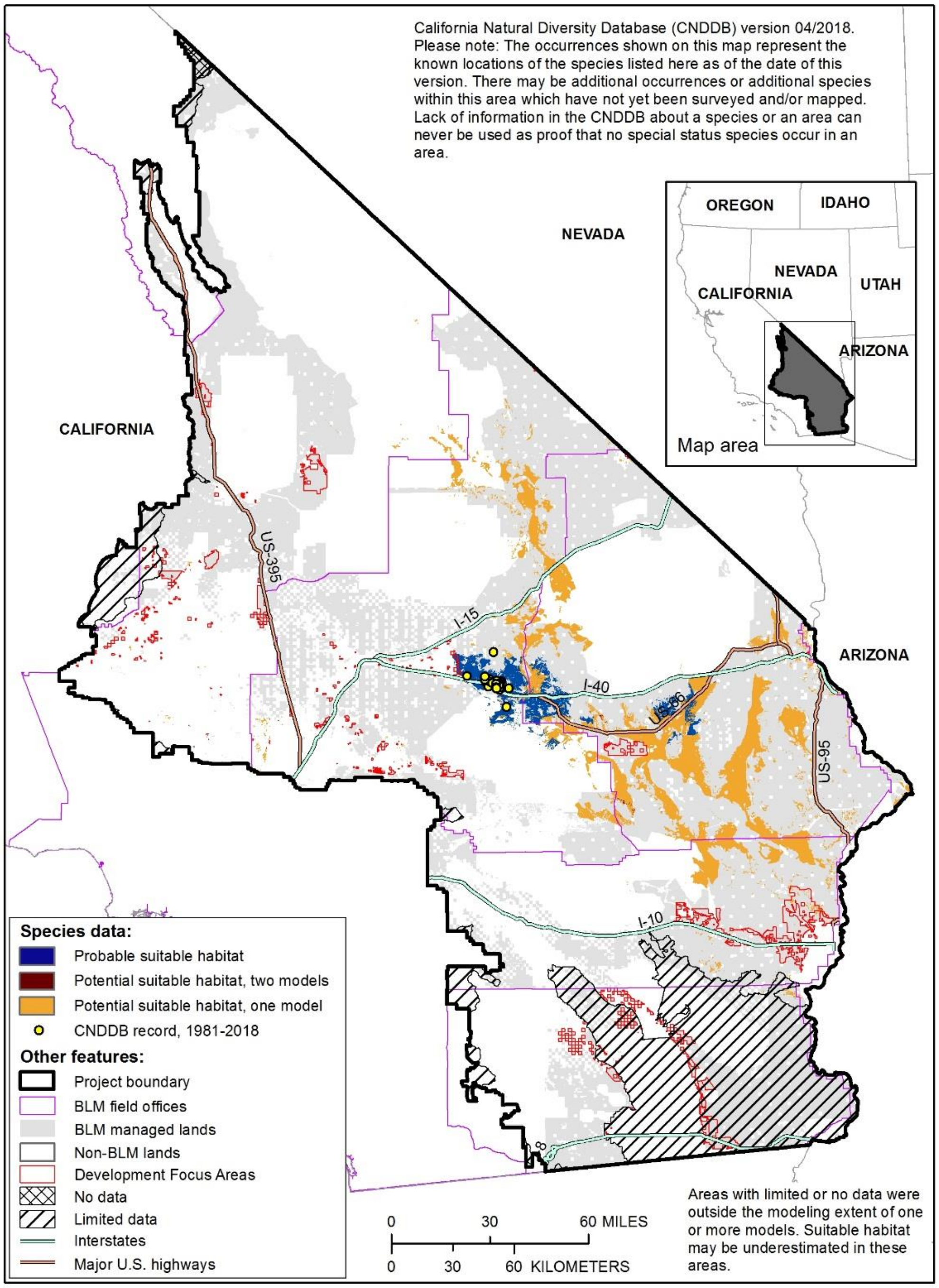

Table B36_*Penstemon bicolor* ssp. *roseus*.

Category	Topic		Contractor B	
Occurrence data used to develop the model	Number of occurrences*		Report/data indicate that model was built from 5 occurrences. Currently available CNDDB data indicate 7 occurrences were available for use by this contractor for model development.	
	Age of occurrences*		0 of 7 (0%) currently available CNDDB occurrences are from prior to 1981. 2 records are from prior to 2000.	
	Spatial accuracy of occurrences*		2 of 7 (28%) currently available CNDDB occurrences have imprecise spatial accuracy.	
	Status of occurrences*		2 of 7 (28%) currently available CNDDB occurrences have Fair or Poor occurrence ranks.	
	Species identification of occurrences*			
	Spatial bias of occurrences*			

Category	Topic		Contractor B	
	Spatial distribution of occurrences*		Currently available CNDDB records in the contractor's boundary are from a limited portion of the area shown for the species in California [70].	
	Absence data			
Environ-mental covariates	Ecological relevance			
	Comprehensive			
	Resolution and scale			
	Accuracy			
	Number of covariates		Model includes 11 covariates and 5 occurrences.	
	Current covariate data			
	Covariate selection			
	Correlation			
Modeling algorithm	Use in the literature			
	Interactions			
	Non-linear			
Modeling extent and resolution	Model extent		The species is not indicated as present in California by the Jepsen Herbarium [54]. Contractor's project boundary includes most of	

Category	Topic		Contractor B	
			the area shown for the species in California in the USDA PLANTS Database [70], but not a complete buffer around it. The species is also found in Nevada and Arizona.	
	Resolution of model output			
Model selection and thresholds	Model selection			
	Selection of threshold for mapping suitable habitat			

Fig A36_*Penstemon bicolor* ssp. *roseus*.

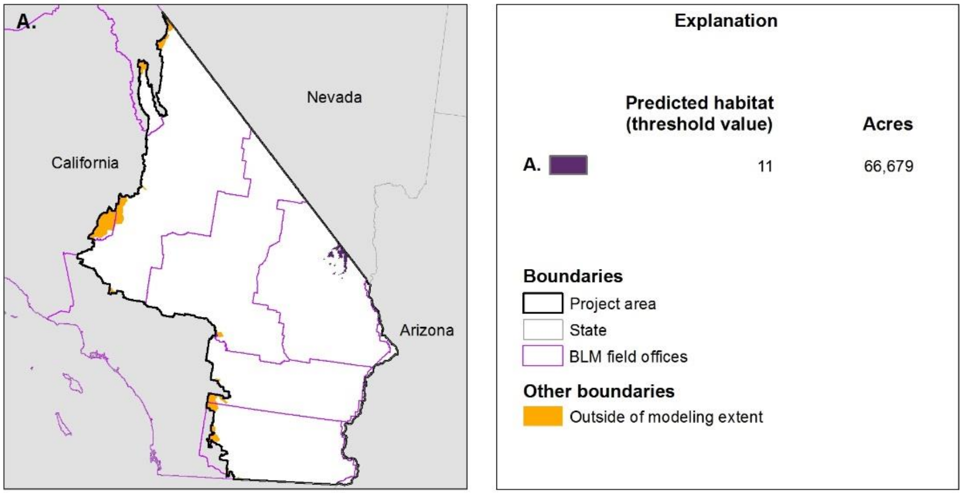

Fig C36_*Penstemon bicolor* ssp. *roseus.*

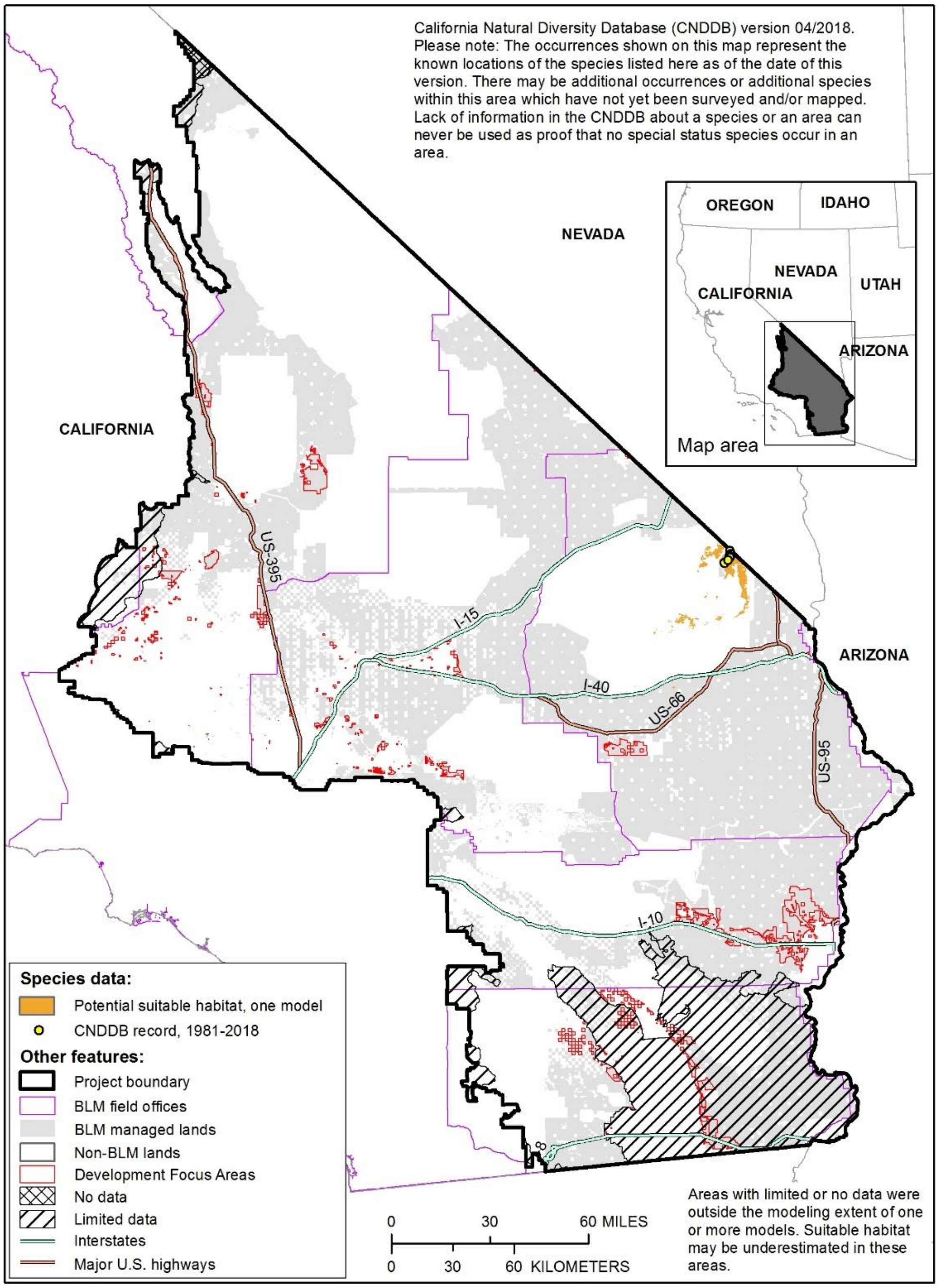

Table B37_*Perityle inyoensis*.

Category	Topic		Contractor B	
Occurrence data used to develop the model	Number of occurrences*		Report/data indicate that model was built from 8 occurrences. Currently available CNDDB data indicate 7 occurrences (with a valid date, 1 has no date) were available for use by this contractor for model development.	
	Age of occurrences*		1 of 7 (14%) currently available CNDDB occurrences are from prior to 1981.	
	Spatial accuracy of occurrences*		1 of 7 (14%) currently available CNDDB occurrences have imprecise spatial accuracy.	
	Status of occurrences*		0 of 7 (0%) currently available CNDDB occurrences have Fair or Poor occurrence ranks.	
	Species identification of occurrences*			
	Spatial bias of occurrences*			

Category	Topic		Contractor B	
	Spatial distribution of occurrences*		Currently available CNDDB records in the contractor’s boundary are from a limited portion of the area shown for the species in California [70].	
	Absence data			
Environ-mental covariates	Ecological relevance			
	Comprehensive			
	Resolution and scale			
	Accuracy			
	Number of covariates		Model includes 14 covariates and 8 occurrences.	
	Current covariate data			
	Covariate selection			
	Correlation			
Modeling algorithm	Use in the literature			
	Interactions			
	Non-linear			
Modeling extent and resolution	Model extent		The species is not indicated as present in California by the Jepsen Herbarium [54]. Contractor’s project boundary includes most of	

Category	Topic		Contractor B	
			the area shown for the species in California in the USDA PLANTS Database [70], but not a complete buffer around it. The species is also found in Nevada and Arizona.	
	Resolution of model output			
Model selection and thresholds	Model selection			
	Selection of threshold for mapping suitable habitat			

Fig A37_*Perityle inyoensis*.

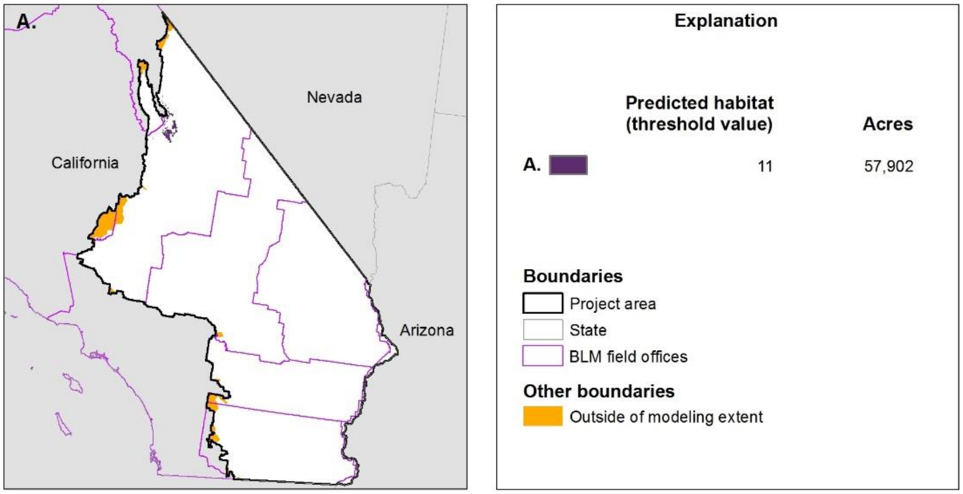

Fig C37_*Perityle inyoensis*.

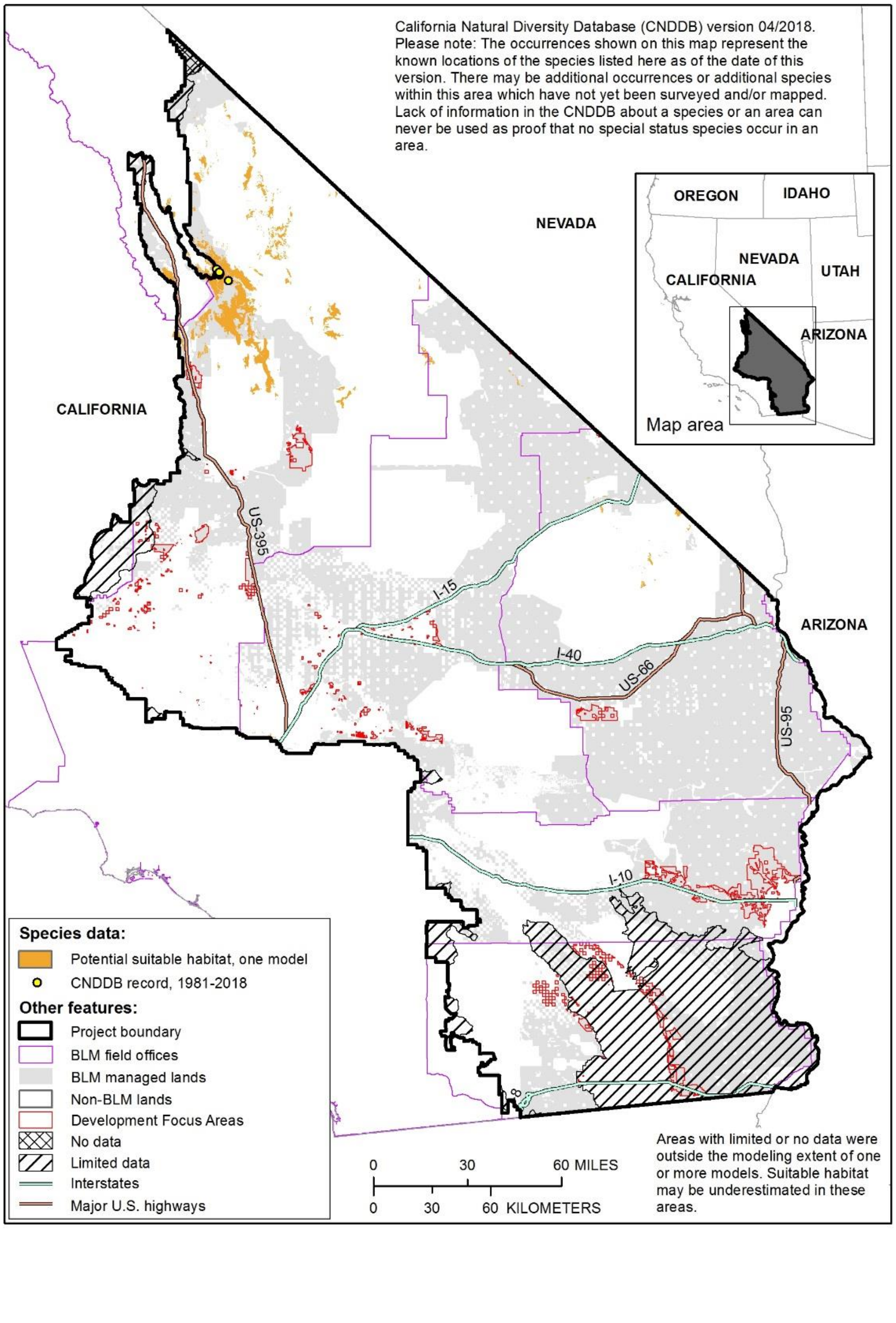

Table B38_*Phacelia nashiana*.

Category	Topic	Contractor A	Contractor B	Contractor C
Occurrence data used to develop the model	Number of occurrences*	Report/data indicate that model was built from 248 occurrences. Currently available CNDDB data indicate 66 occurrences were likely used by this contractor for model development.	Report/data indicate that model was built from 61 occurrences. Currently available CNDDB data indicate 67 occurrences were available for use by this contractor for model development.	Report/data indicate that model was built from 89 occurrences. Currently available CNDDB data indicate 67 occurrences were available for use by this contractor for model development.
	Age of occurrences*	Report indicates use of occurrence data from 1981-2012. Many records are from prior to 2000.	1 of 67 (1%) currently available CNDDB occurrences is from prior to 1981.	1 of 67 (1%) currently available CNDDB occurrences is from prior to 1981.
	Spatial accuracy of occurrences*	Report/data indicate occurrences with uncertainty >250-500 m were excluded.	7 of 67 (10%) currently available CNDDB occurrences have imprecise spatial accuracy.	7 of 67 (10%) currently available CNDDB occurrences have imprecise spatial accuracy.
	Status of occurrences*	12 of 66 (18%) currently available CNDDB occurrences have Fair or Poor occurrence ranks.	12 of 67 (18%) currently available CNDDB occurrences have Fair or Poor occurrence ranks.	12 of 67 (18%) currently available CNDDB occurrences have Fair or Poor occurrence ranks.
	Species identification of occurrences*			A substantial portion of records appear to be from a source other than CNDDB, for which the reliability of species

Category	Topic	Contractor A	Contractor B	Contractor C
				identification is unknown.
	Spatial bias of occurrences*			
	Spatial distribution of occurrences*	Currently available CNDDB records in the contractor's boundary are from a limited portion of the area of the occupied geographic subdivisions for the species in California [54].	Currently available CNDDB records in the contractor's boundary are from a limited portion of the area of the occupied geographic subdivisions for the species in California [54].	Currently available CNDDB records in the contractor's boundary are from a limited portion of the area of the occupied geographic subdivisions for the species in California [54].
	Absence data			
Environ-mental covariates	Ecological relevance			
	Comprehensive			
	Resolution and scale			
	Accuracy			
	Number of covariates	Model includes 12 covariates and 248 occurrences.	Model includes 18 covariates and 61 occurrences.	Model includes 9 covariates and 89 occurrences; report stated that no more than one variable per 10 occurrences was allowed.
	Current covariate data			
	Covariate selection			
	Correlation			

Category	Topic	Contractor A	Contractor B	Contractor C
Modeling algorithm	Use in the literature			
	Interactions			
	Non-linear			
Modeling extent and resolution	Model extent	Contractor's project boundary includes much but not all of the area of the occupied geographic subdivisions for the species in California [54].	Contractor's project boundary excludes a substantial area of the occupied geographic subdivisions for the species in California [54].	Contractor's project boundary includes much but not all of the area of the occupied geographic subdivisions for the species in California [54].
	Resolution of model output			
Model selection and thresholds	Model selection			
	Selection of threshold for mapping suitable habitat			

Fig A38_*Phacelia nashiana*.

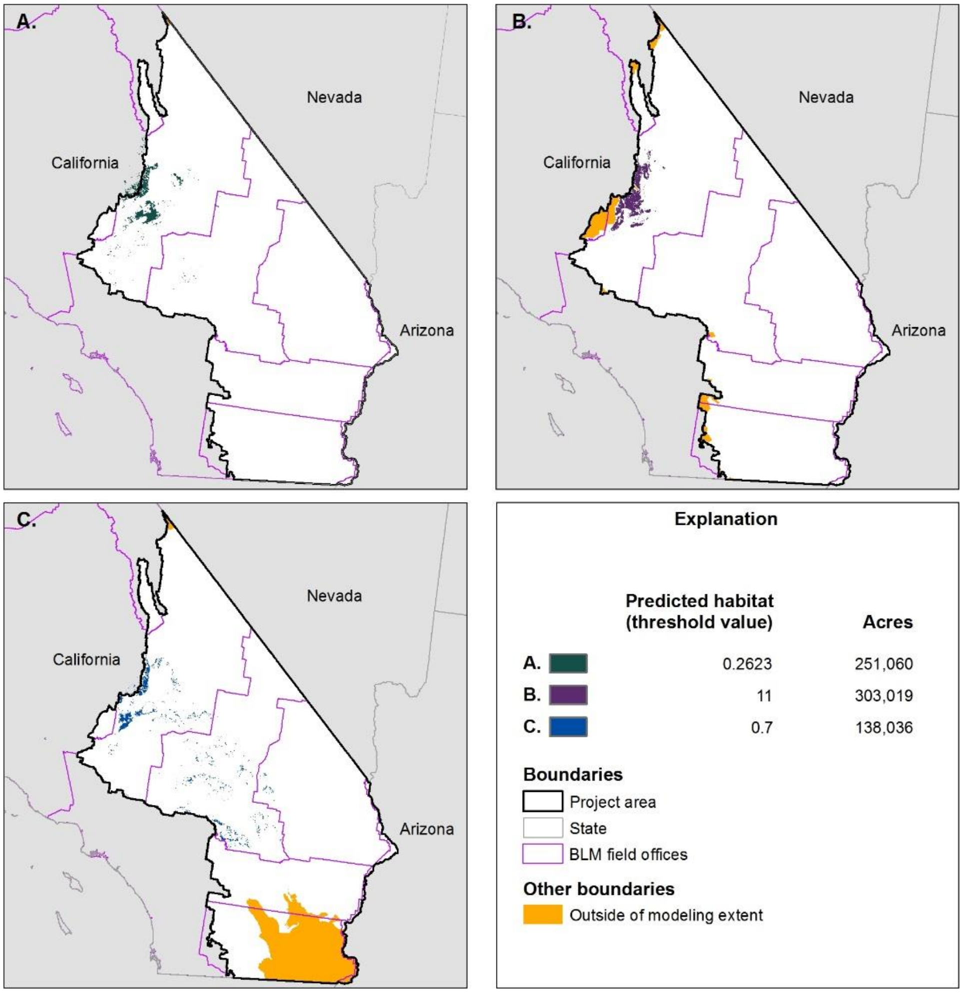

Fig B38_*Phacelia nashiana*.

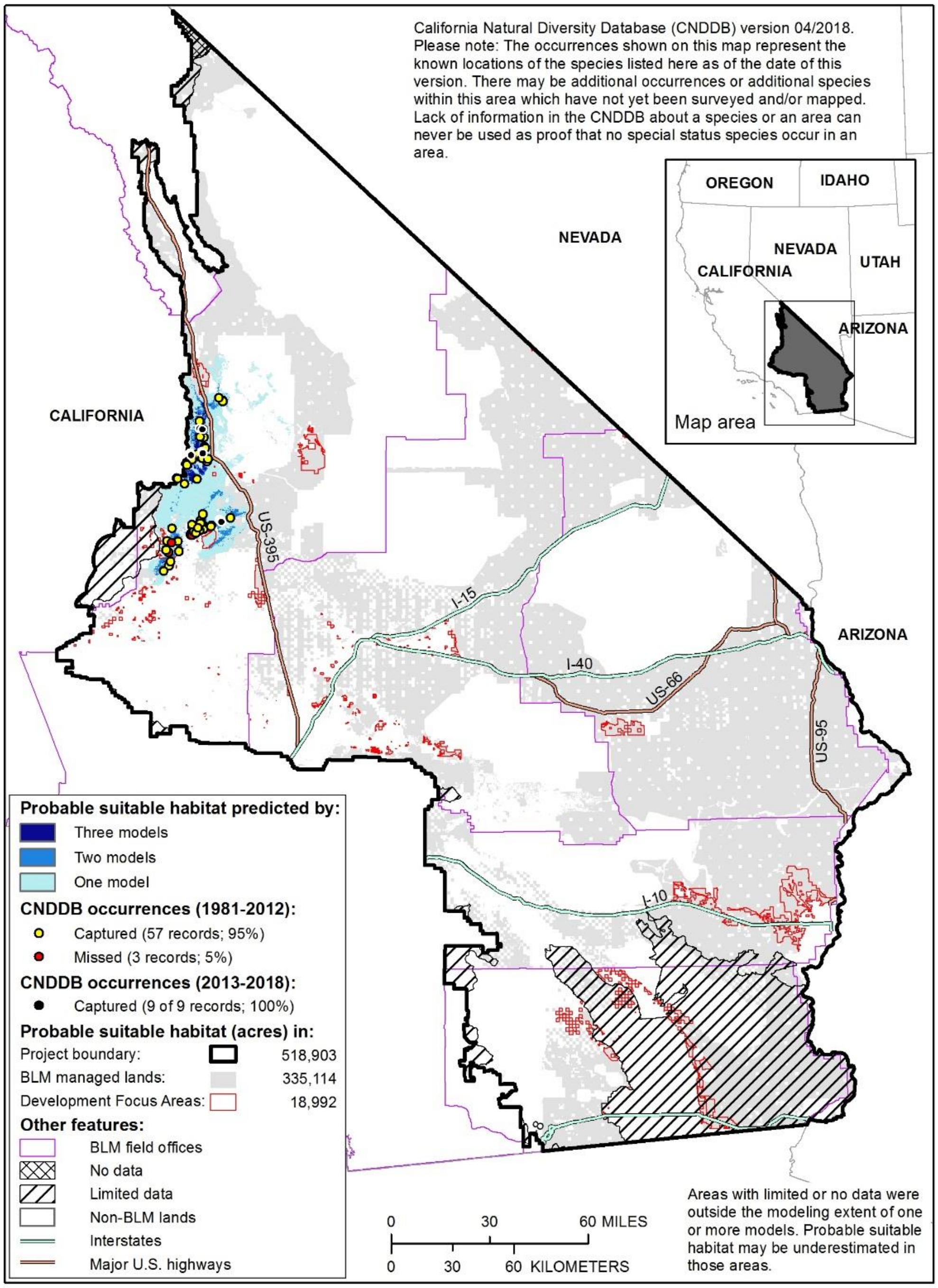

Fig C38_*Phacelia nashiana*.

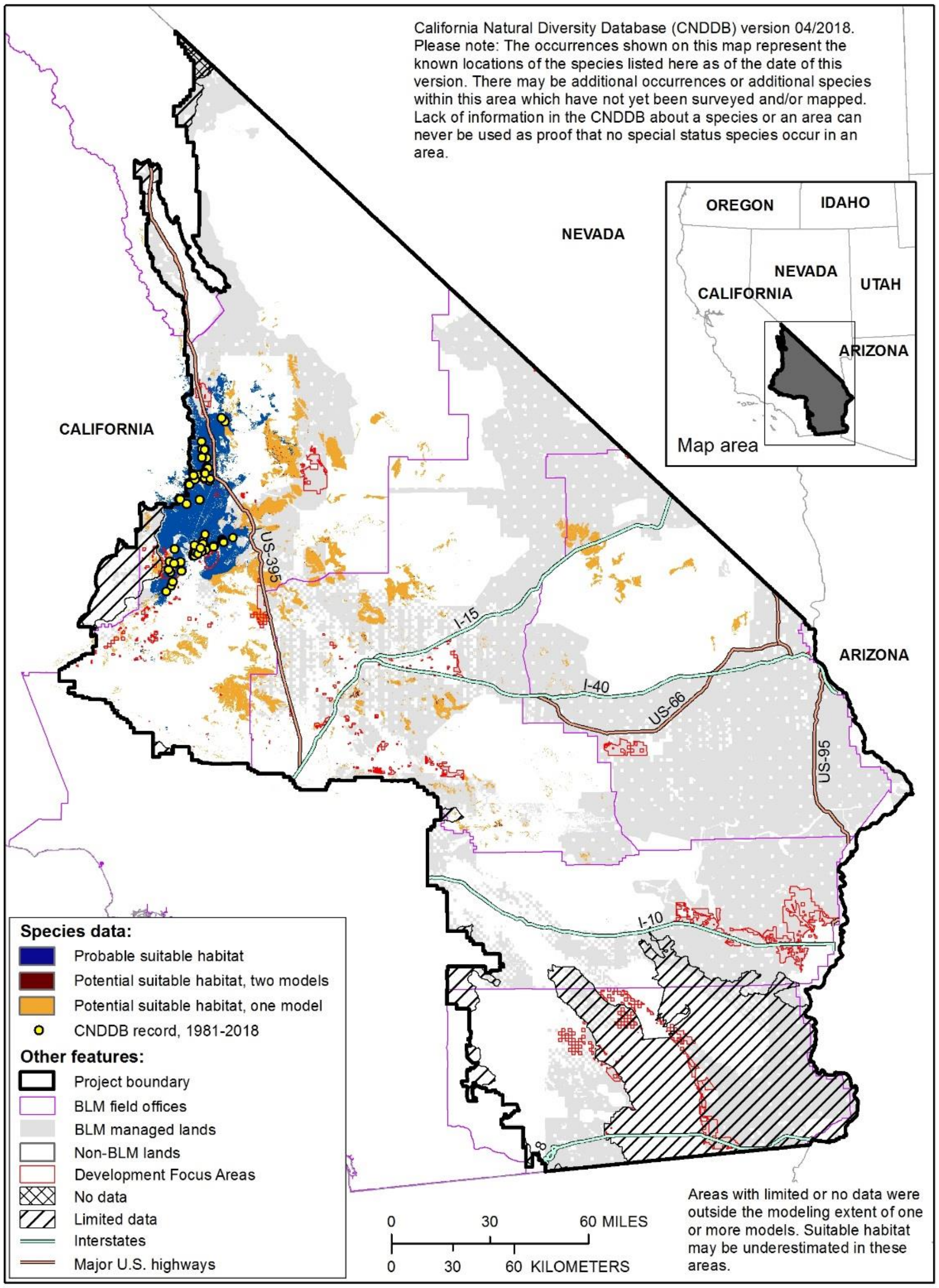

Table B39_*Phacelia parishii*.

Category	Topic		Contractor B	
Occurrence data used to develop the model	Number of occurrences*		Report/data indicate that model was built from 9 occurrences. Currently available CNDDB data indicate 9 occurrences were available for use by this contractor for model development.	
	Age of occurrences*		2 of 9 (22%) currently available CNDDB occurrences are from prior to 1981.	
	Spatial accuracy of occurrences*		3 of 9 (33%) currently available CNDDB occurrences have imprecise spatial accuracy.	
	Status of occurrences*		0 of 9 (0%) currently available CNDDB occurrences have Fair or Poor occurrence ranks.	
	Species identification of occurrences*			
	Spatial bias of occurrences*			

Category	Topic		Contractor B	
	Spatial distribution of occurrences*		Currently available CNDDB records in the contractor's boundary are from a substantial portion of the area shown for the species in California [70].	
	Absence data			
Environ-mental covariates	Ecological relevance			
	Comprehensive			
	Resolution and scale			
	Accuracy			
	Number of covariates		Model includes 17 covariates and 9 occurrences.	
	Current covariate data			
	Covariate selection			
	Correlation			
Modeling algorithm	Use in the literature			
	Interactions			
	Non-linear			
Modeling extent and resolution	Model extent		Contractor's project boundary includes much of the area of the occupied geographic	

Category	Topic		Contractor B	
			subdivisions for the species in California [54], but not a complete buffer around them.	
	Resolution of model output			
Model selection and thresholds	Model selection			
	Selection of threshold for mapping suitable habitat			

Fig A39_*Phacelia parishii*.

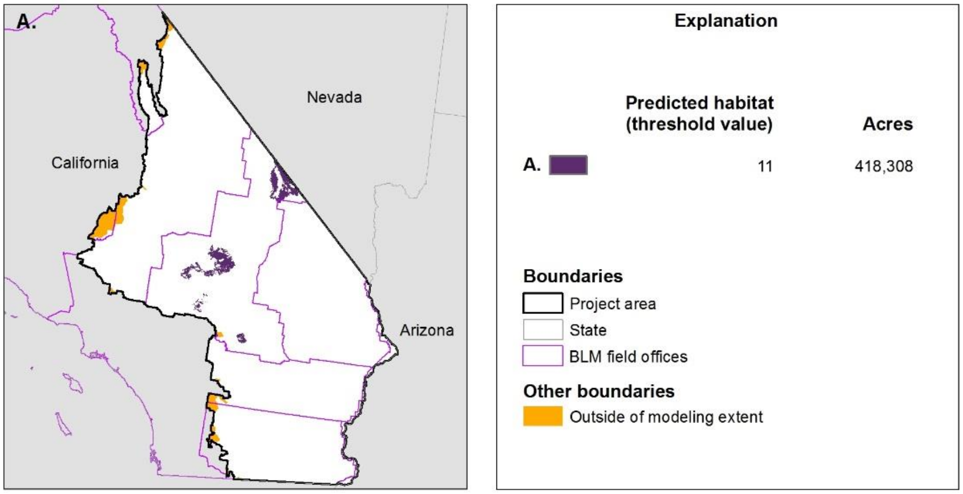

Fig C39_*Phacelia parishii*.

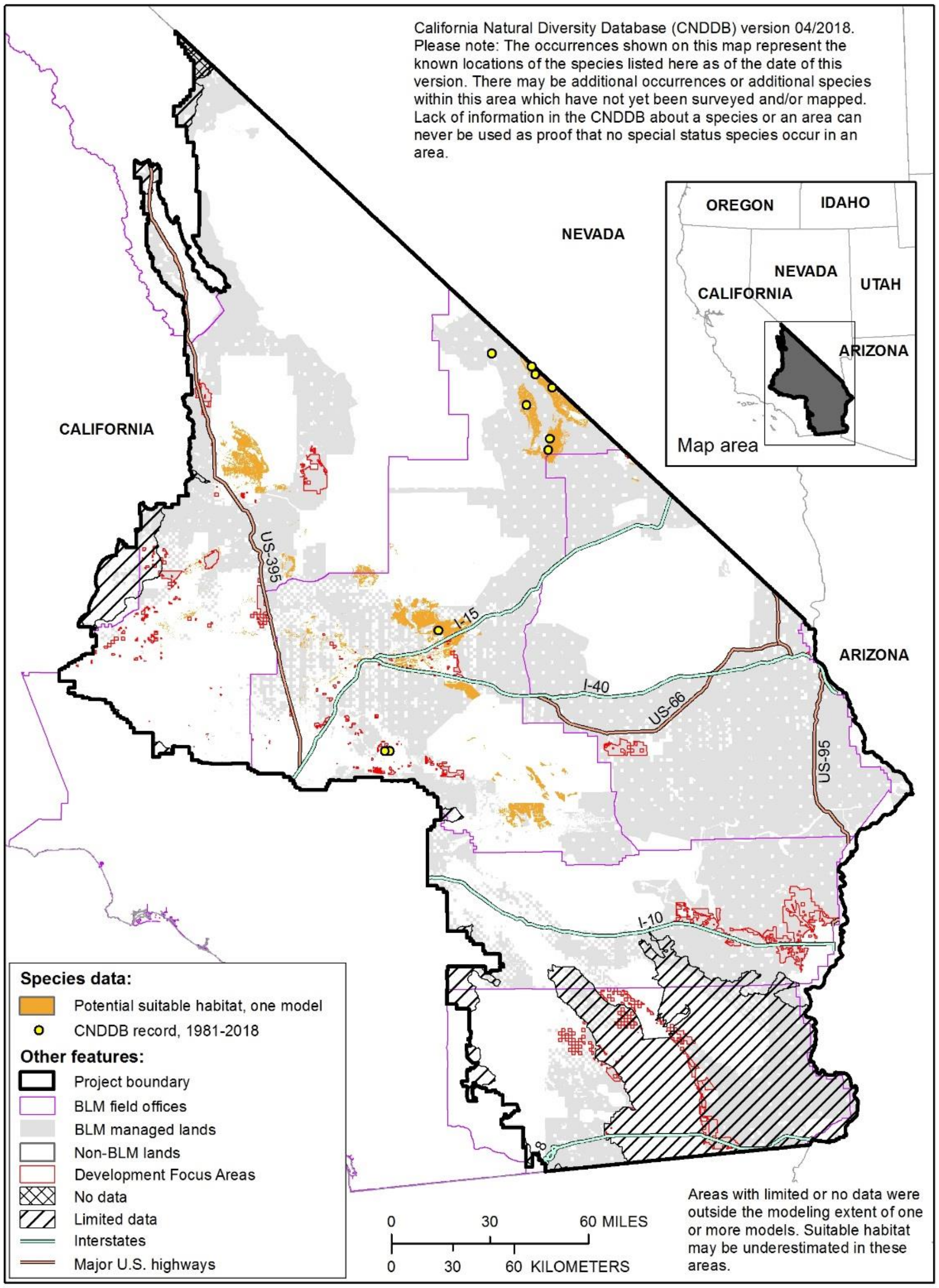

Table B40_*Saltugilia latimeri*.

Category	Topic		Contractor B	
Occurrence data used to develop the model	Number of occurrences*		Report/data indicate that model was built from 16 occurrences. Currently available CNDDB data indicate 29 occurrences were available for use by this contractor for model development.	
	Age of occurrences*		14 of 29 (48%) currently available CNDDB occurrences are from prior to 1981.	
	Spatial accuracy of occurrences*		15 of 29 (52%) currently available CNDDB occurrences have imprecise spatial accuracy.	
	Status of occurrences*		0 of 29 (0%) currently available CNDDB occurrences have Fair or Poor occurrence ranks.	
	Species identification of occurrences*			
	Spatial bias of occurrences*			

Category	Topic		Contractor B	
	Spatial distribution of occurrences*		Currently available CNDDB records in the contractor's boundary are from a substantial portion of the area shown for the species in California [70].	
	Absence data			
Environ-mental covariates	Ecological relevance			
	Comprehensive			
	Resolution and scale			
	Accuracy			
	Number of covariates		Model includes 16 covariates and 16 occurrences.	
	Current covariate data			
	Covariate selection			
	Correlation			
Modeling algorithm	Use in the literature			
	Interactions			
	Non-linear			
Modeling extent and resolution	Model extent		Contractor's project boundary excludes a substantial portion of the area of the occupied	

Category	Topic		Contractor B	
			geographic subdivisions for the species in California [54].	
	Resolution of model output			
Model selection and thresholds	Model selection			
	Selection of threshold for mapping suitable habitat			

Fig A40_*Saltugilia latimeri*.

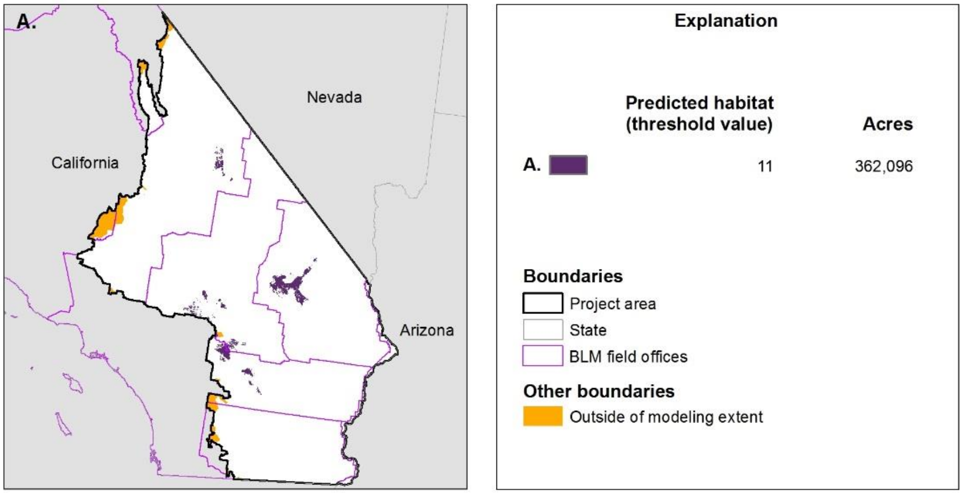

Fig C40_*Saltugilia latimeri*.

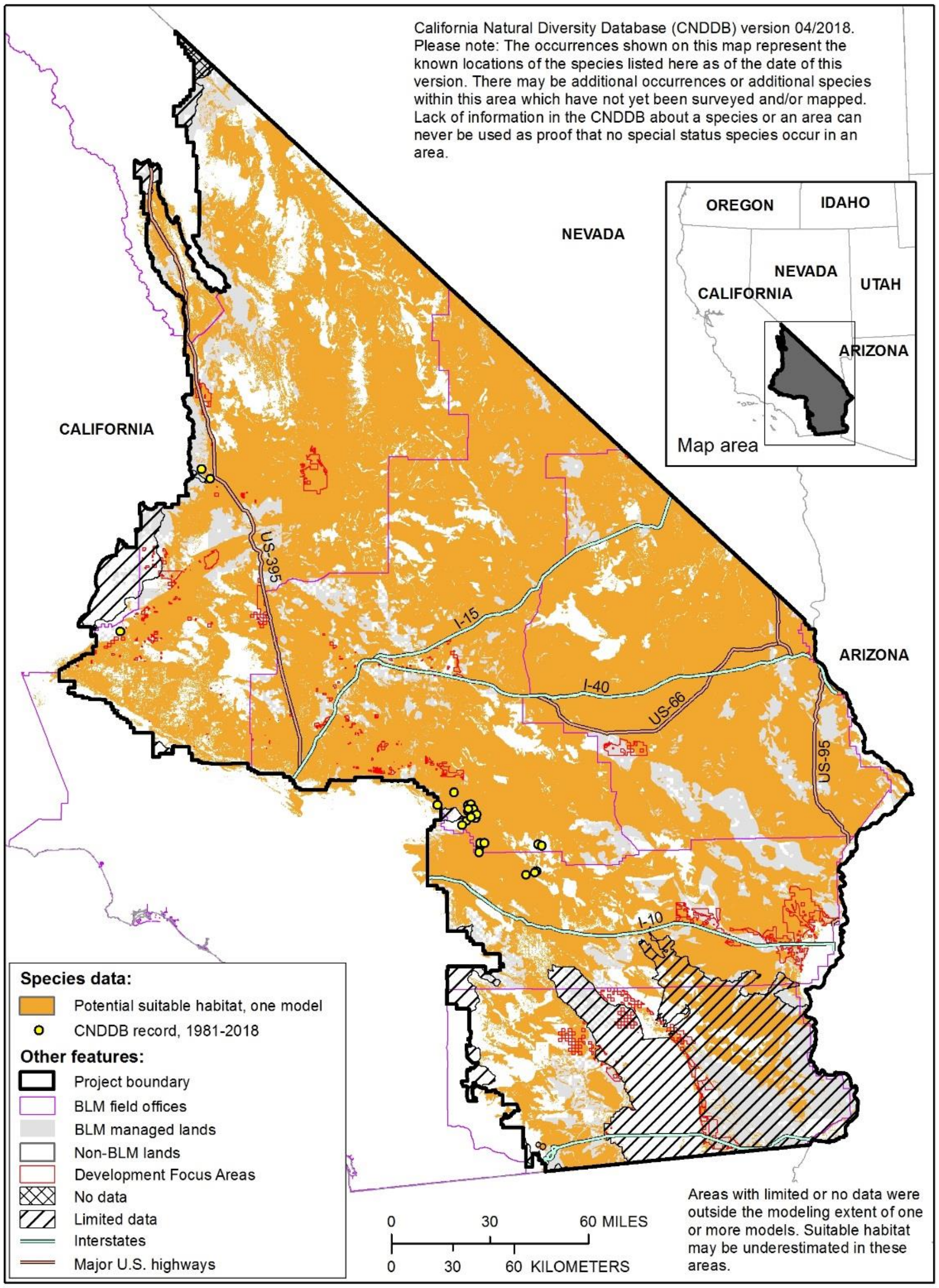

Table B41_*Sidalcea covillei*.

Category	Topic	Contractor A		
Occurrence data used to develop the model	Number of occurrences*	Report/data indicate that model was built from 73 occurrences. Currently available CNDDB data indicate 38 occurrences were likely used by this contractor for model development.		
	Age of occurrences*	Report indicates use of occurrence data from 1981-2012. Numerous records are from prior to 2000.		
	Spatial accuracy of occurrences*	Report/data indicate occurrences with uncertainty >250-500 m were excluded.		
	Status of occurrences*	13 of 38 (34%) currently available CNDDB occurrences have Fair or Poor occurrence ranks.		
	Species identification of occurrences*			
	Spatial bias of occurrences*			

Category	Topic	Contractor A		
	Spatial distribution of occurrences*	Currently available CNDDB records in the contractor's boundary are from a substantial portion of the area of the occupied geographic subdivisions for the species in California [54].		
	Absence data			
Environ-mental covariates	Ecological relevance			
	Comprehensive			
	Resolution and scale			
	Accuracy			
	Number of covariates	Model includes 9 covariates and 73 occurrences.		
	Current covariate data			
	Covariate selection			
	Correlation			
Modeling algorithm	Use in the literature			
	Interactions			
	Non-linear			

Category	Topic	Contractor A		
Modeling extent and resolution	Model extent	Contractor's project boundary excludes a significant portion of the area of the occupied geographic subdivisions for the species in California [54].		
	Resolution of model output			
Model selection and thresholds	Model selection			
	Selection of threshold for mapping suitable habitat			

Fig A41_*Sidalcea covillei*.

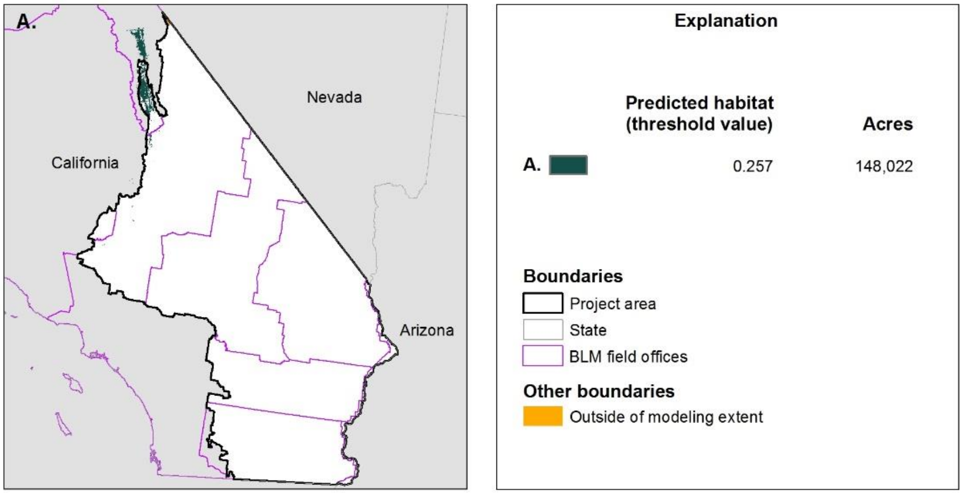

Fig B41_*Sidalcea covillei*.

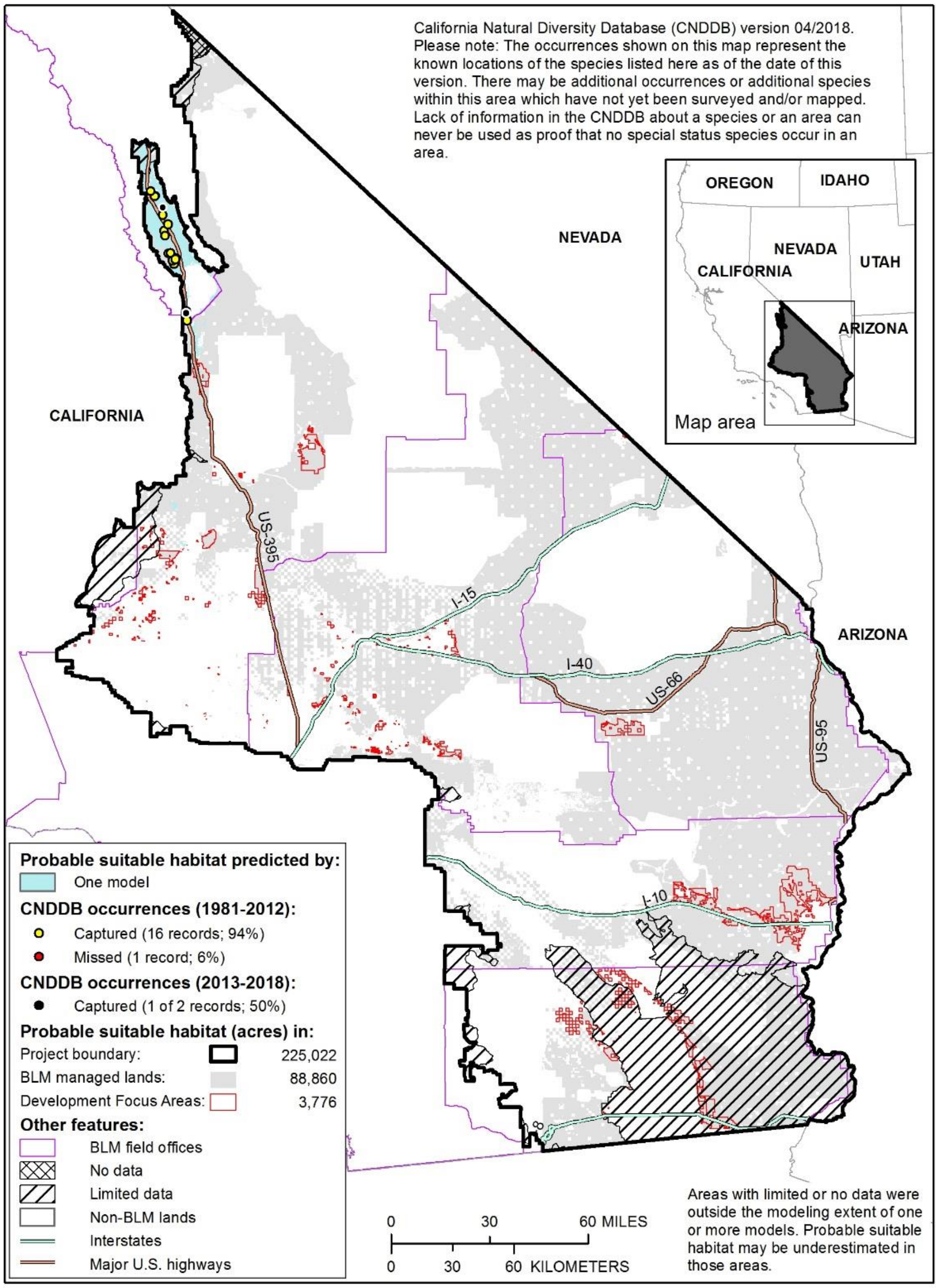

Fig C41_*Sidalcea covillei*. No map produced; all existing model information contained in map of probable suitable habitat.

Table B42_*Sphaeralcea rusbyi* var. *eremicola*.

Category	Topic	Contractor A	Contractor B	Contractor C
Occurrence data used to develop the model	Number of occurrences*	Report/data indicate that model was built from 123 occurrences. Currently available CNDDB data indicate 45 occurrences were likely used by this contractor for model development.	Report/data indicate that model was built from 49 occurrences. Currently available CNDDB data indicate 47 occurrences were available for use by this contractor for model development.	Report/data indicate that model was built from 75 occurrences. Currently available CNDDB data indicate 47 occurrences were available for use by this contractor for model development.
	Age of occurrences*	Report indicates use of occurrence data from 1981-2012. 5 records are from prior to 2000.	2 of 47 (4%) currently available CNDDB occurrences is from prior to 1981.	2 of 47 (4%) currently available CNDDB occurrences is from prior to 1981.
	Spatial accuracy of occurrences*	Report/data indicate occurrences with uncertainty >250-500 m were excluded.	8 of 47 (17%) currently available CNDDB occurrences have imprecise spatial accuracy.	8 of 47 (17%) currently available CNDDB occurrences have imprecise spatial accuracy.
	Status of occurrences*	4 of 45 (9%) currently available CNDDB occurrences have Fair or Poor occurrence ranks.	4 of 47 (8%) currently available CNDDB occurrences have Fair or Poor occurrence ranks.	4 of 47 (8%) currently available CNDDB occurrences have Fair or Poor occurrence ranks.
	Species identification of occurrences*			A substantial portion of records appear to be from a source other than CNDDB, for which the reliability of species identification is unknown.

Category	Topic	Contractor A	Contractor B	Contractor C
	Spatial bias of occurrences*			
	Spatial distribution of occurrences*	Currently available CNDDB records in the contractor's boundary are from a substantial portion of the area of the occupied geographic subdivisions for the species in California [54].	Currently available CNDDB records in the contractor's boundary are from a substantial portion of the area of the occupied geographic subdivisions for the species in California [54].	Currently available CNDDB records in the contractor's boundary are from a substantial portion of the area of the occupied geographic subdivisions for the species in California [54].
	Absence data			
Environ-mental covariates	Ecological relevance			
	Comprehensive			
	Resolution and scale			
	Accuracy			
	Number of covariates	Model includes 12 covariates and 123 occurrences.	Model includes 15 covariates and 49 occurrences.	Model includes 7 covariates and 57 occurrences; report stated that no more than one variable per 10 occurrences was allowed.
	Current covariate data			
	Covariate selection			
	Correlation			
Modeling algorithm	Use in the literature			
	Interactions			

Category	Topic	Contractor A	Contractor B	Contractor C
	Non-linear			
Modeling extent and resolution	Model extent	Contractor’s project boundary includes most or all of the area of the occupied geographic subdivisions for the species in California [54], but not a complete buffer around them.	Contractor’s project boundary includes most of the area of the occupied geographic subdivisions for the species in California [54], but not a complete buffer around them.	Contractor’s project boundary includes most or all of the area of the occupied geographic subdivisions for the species in California [54], but not a complete buffer around them.
	Resolution of model output			
Model selection and thresholds	Model selection			
	Selection of threshold for mapping suitable habitat			

Fig A42_*Sphaeralcea rusbyi* var. *eremicola*.

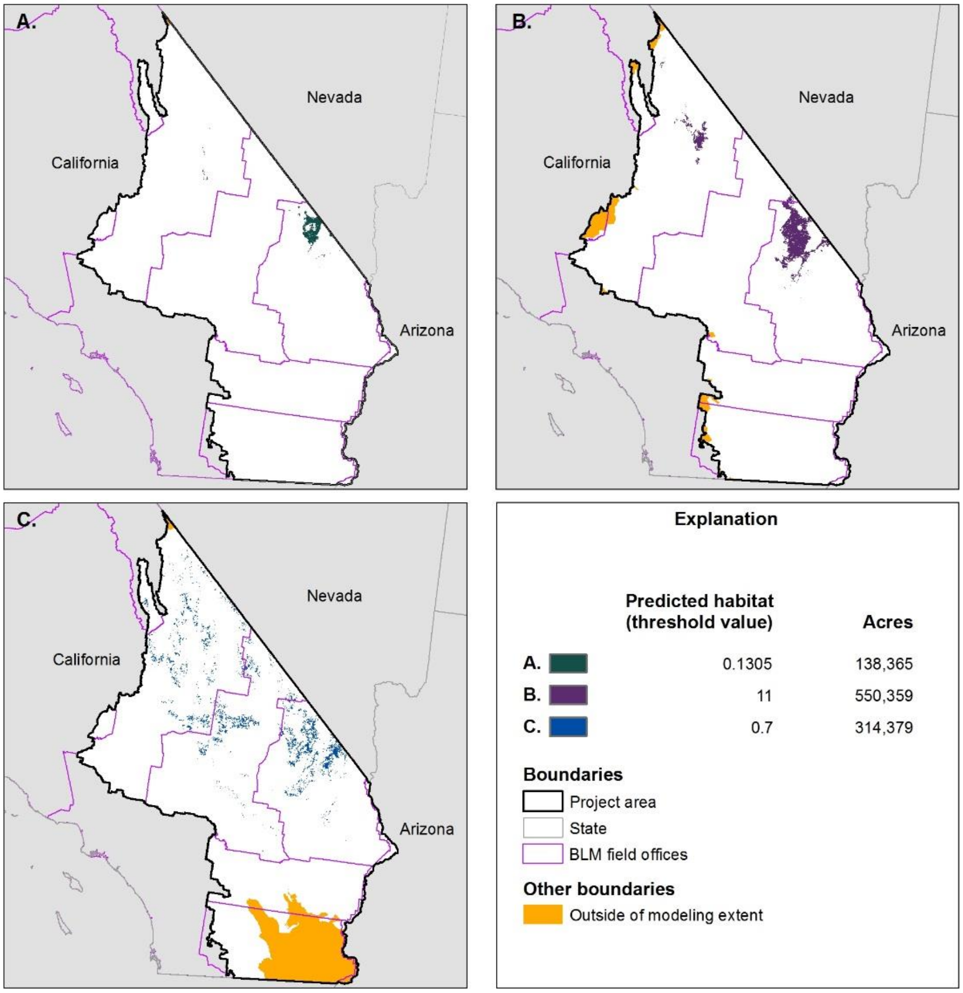

Fig B42_*Sphaeralcea rusbyi* var. *eremicola*.

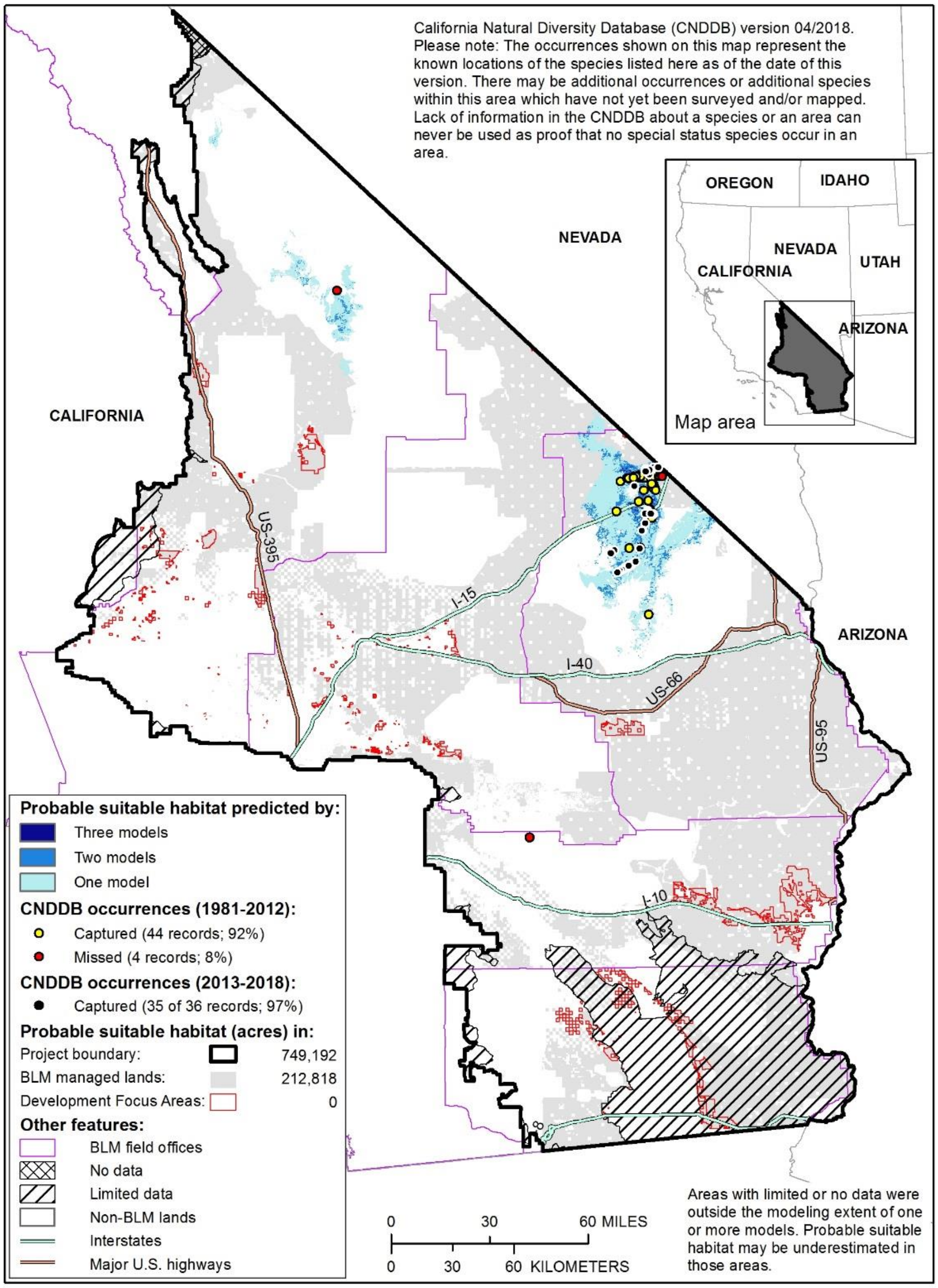

Fig C42_*Sphaeralcea rusbyi* var. *eremicola*.

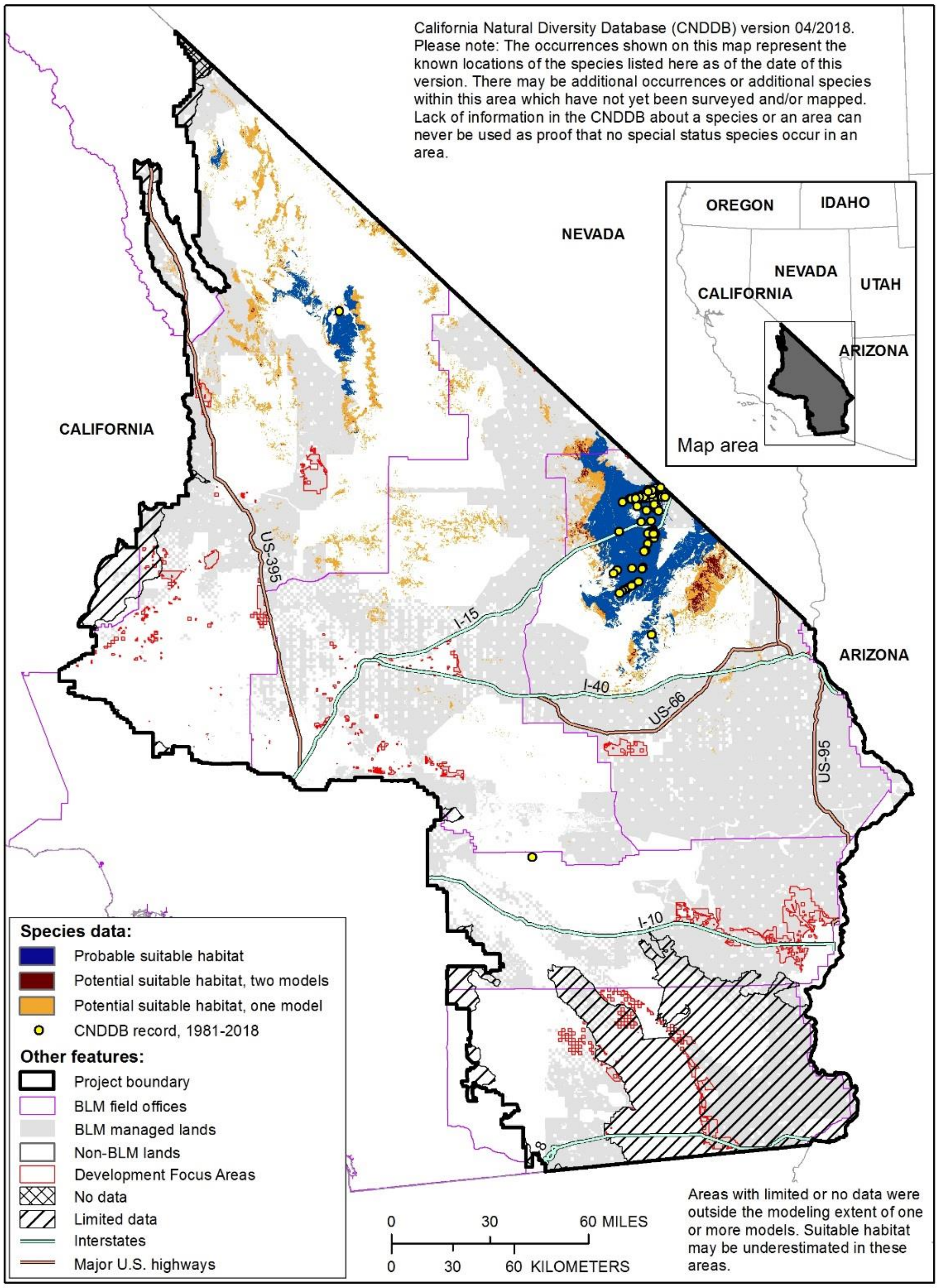

Table B43_*Xylorhiza orcuttii*.

Category	Topic		Contractor B	Contractor C
Occurrence data used to develop the model	Number of occurrences*		Report/data indicate that model was built from 61 occurrences. Currently available CNDDB data indicate 64 occurrences were available for use by this contractor for model development.	Report/data indicate that model was built from 109 occurrences. Currently available CNDDB data indicate 64 occurrences were available for use by this contractor for model development.
	Age of occurrences*		19 of 64 (30%) currently available CNDDB occurrences is from prior to 1981.	19 of 64 (30%) currently available CNDDB occurrences is from prior to 1981.
	Spatial accuracy of occurrences*		21 of 64 (33%) currently available CNDDB occurrences have imprecise spatial accuracy.	21 of 64 (33%) currently available CNDDB occurrences have imprecise spatial accuracy.
	Status of occurrences*		3 of 64 (5%) currently available CNDDB occurrences have Fair or Poor occurrence ranks.	3 of 64 (5%) currently available CNDDB occurrences have Fair or Poor occurrence ranks.
	Species identification of occurrences*			A substantial portion of records appear to be from a source other than CNDDB, for which the reliability of species identification is unknown.

Category	Topic		Contractor B	Contractor C
	Spatial bias of occurrences*			
	Spatial distribution of occurrences*		Currently available CNDDB records in the contractor’s boundary are from a limited portion of the area of the occupied geographic subdivision for the species in California [54].	Currently available CNDDB records in the contractor’s boundary are from a limited portion of the area of the occupied geographic subdivision for the species in California [54].
	Absence data			
Environ-mental covariates	Ecological relevance			
	Comprehensive			
	Resolution and scale			
	Accuracy			
	Number of covariates		Model includes 15 covariates and 61 occurrences.	Model includes 9 covariates and 109 occurrences; report stated that no more than one variable per 10 occurrences was allowed.
	Current covariate data			
	Covariate selection			
	Correlation			
Modeling algorithm	Use in the literature			
	Interactions			

Category	Topic		Contractor B	Contractor C
	Non-linear			
Modeling extent and resolution	Model extent		Contractor's project boundary includes most of the area of the occupied geographic subdivision for the species in California [54], but not a complete buffer around it.	Contractor's project boundary includes most or all of the area of the occupied geographic subdivision for the species in California [54], but not a complete buffer around it.
	Resolution of model output			
Model selection and thresholds	Model selection			
	Selection of threshold for mapping suitable habitat			

Fig A43_*Xylorhiza orcuttii*.

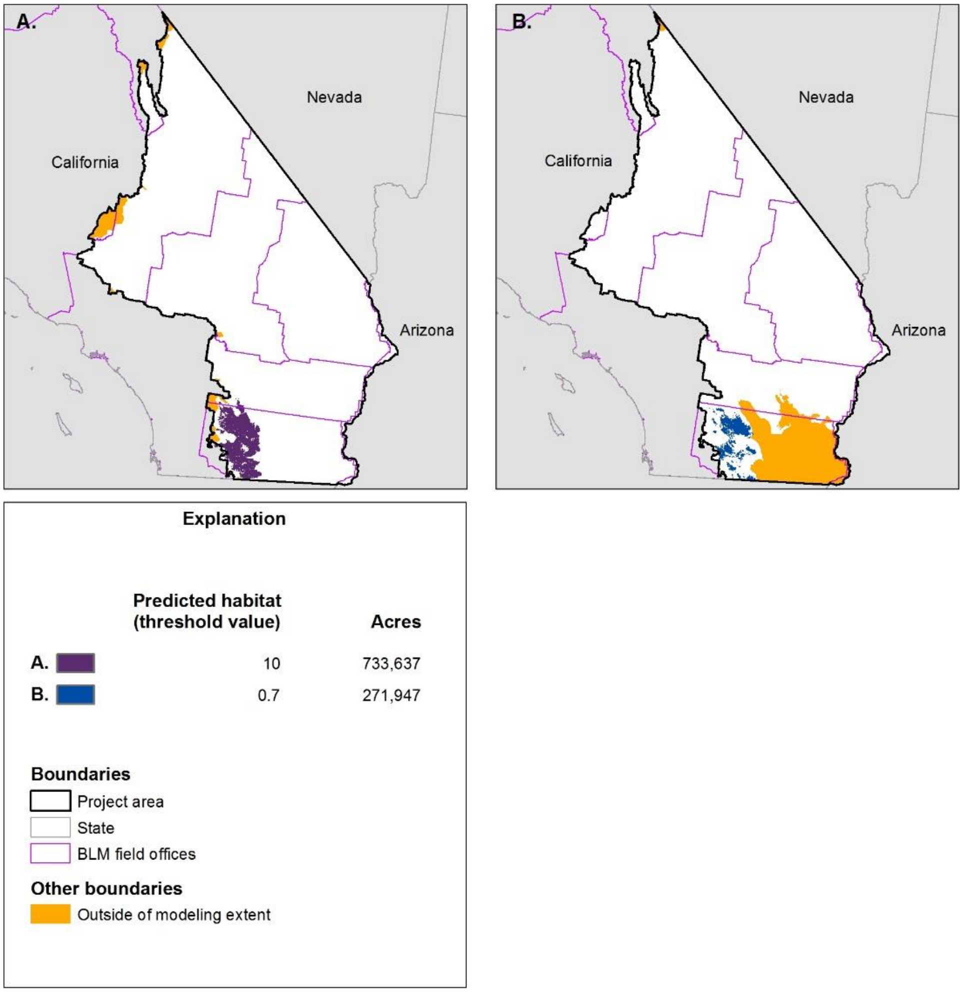

Fig B43_*Xylorhiza orcuttii*.

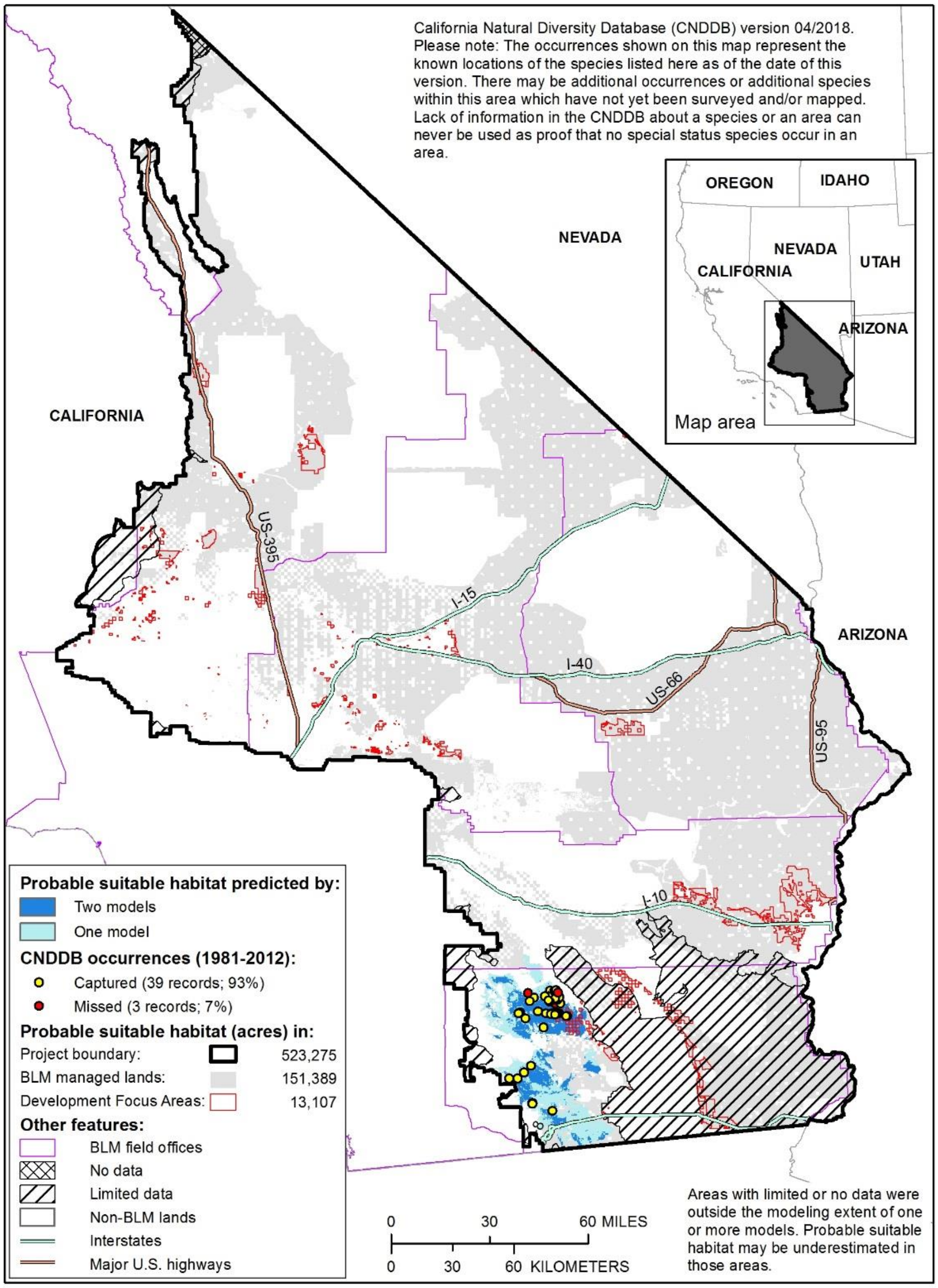

Fig C43_*Xylorhiza orcuttii*.

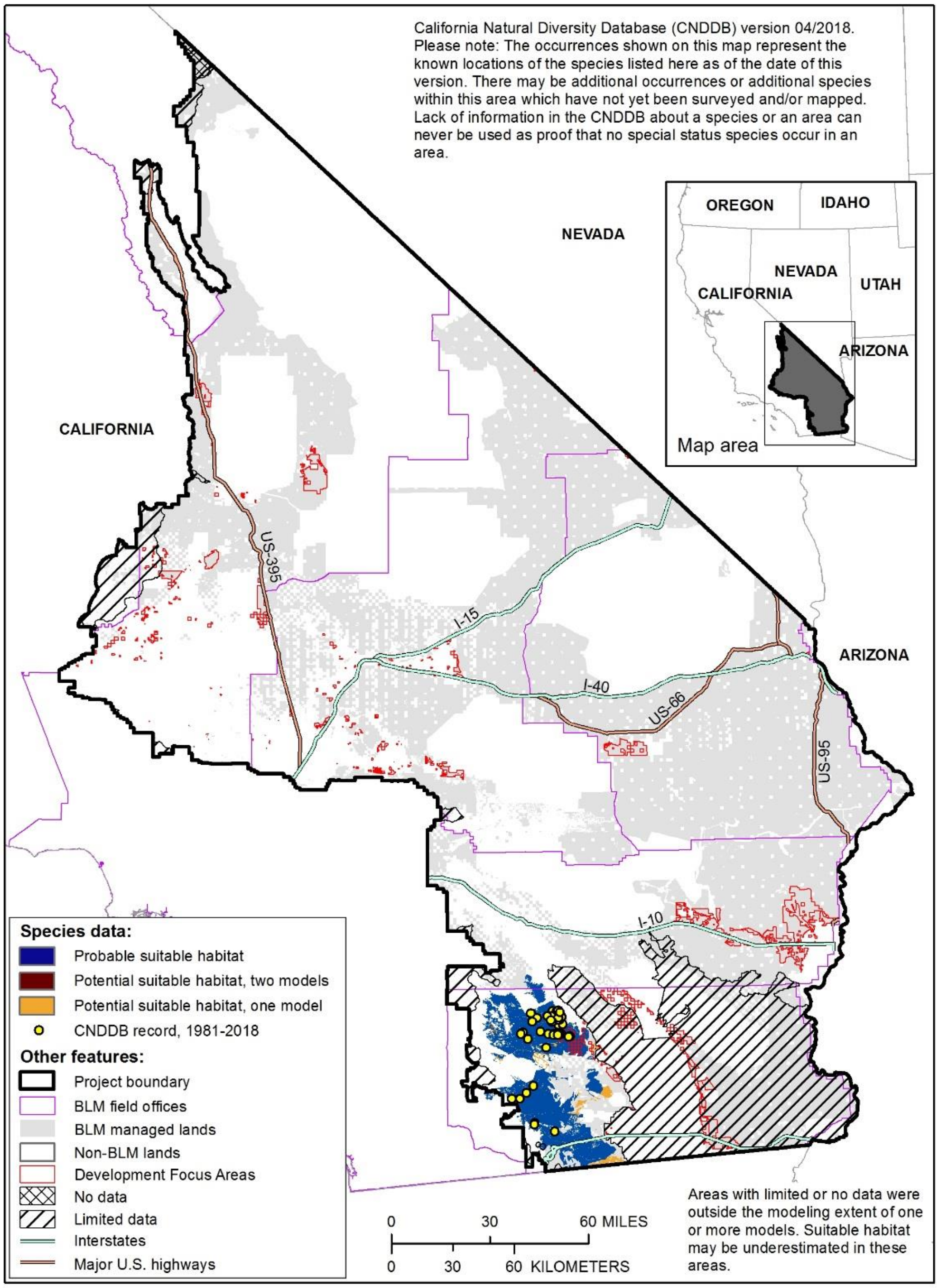

Supplement: S1 Supporting Information — Materials consist of a summary table, evaluations of the model construction for all species, maps of existing habitat models for all species, maps of probable suitable habitat for 26 species, and maps of potential suitable habitat for targeting future plant surveys for 41 species. (PDF) [file pone.0214099.s001.pdf]
